# Cedalion tutorial: a Python-based framework for comprehensive analysis of multimodal fNIRS and DOT from the lab to the everyday world

**DOI:** 10.1117/1.NPh.13.S3.S32602

**Published:** 2026-08-13

**Authors:** Eike Middell, Laura B. Carlton, Shakiba Moradi, Tomás Codina, Thomas Fischer, Josef Cutler, Shannon M. Kelley, Jacqueline Behrendt, Theekshana Dissanayake, Nils Harmening, Meryem A. Yücel, David A. Boas, Alexander von Lühmann

**Affiliations:** a Technische Universität Berlin, Intelligent Biomedical Sensing (IBS) Lab, Berlin, Germany; b BIFOLD – Berlin Institute for the Foundations of Learning and Data, Berlin, Germany; c Boston University, Neurophotonics Center, Biomedical Engineering, Boston, Massachusetts, United States

**Keywords:** functional near-infrared spectroscopy, diffuse optical tomography, multimodal, machine learning, data-driven, physiology, everyday neuroscience.

## Abstract

Functional near-infrared spectroscopy (fNIRS) and diffuse optical 1 tomography (DOT) are rapidly evolving toward wearable, multimodal, data-driven, and artificial-intelligence-supported neuroimaging in the everyday world. However, current analytical tools are fragmented across platforms, limiting reproducibility, interoperability, and integration with modern machine learning (ML) workflows. Cedalion is a Python-based open-source framework designed to unify advanced model-based and data-driven analysis of multimodal fNIRS and DOT data within a reproducible, extensible, and community-driven environment. Cedalion integrates forward modeling, photogrammetric optode coregistration, signal processing, general linear model (GLM) analysis, DOT image reconstruction, and ML-based data-driven methods within a single standardized architecture based on the Python ecosystem. It adheres to SNIRF and BIDS standards, supports cloud-executable Jupyter notebooks, and provides containerized workflows for scalable, fully reproducible analysis pipelines that can be provided alongside original research publications. Cedalion connects established optical-neuroimaging pipelines with ML frameworks such as scikit-learn and PyTorch, enabling seamless multimodal fusion with electroencephalography (EEG), magnetoencephalography (MEG), and physiological data. It implements validated algorithms for signal quality assessment, motion correction, GLM modeling, and DOT reconstruction, complemented by modules for simulation, data augmentation, and multimodal physiology analysis. Automated documentation links each method to its source publication, and continuous-integration testing ensures robustness. This tutorial paper provides seven fully executable notebooks that demonstrate core features. Cedalion offers an open, transparent, and community-extensible foundation that supports reproducible, scalable, and cloud- and ML-ready fNIRS/ DOT workflows for laboratory-based and real-world neuroimaging.

## 1 Introduction

### 1.1 Toward Data-Driven Functional Near-Infrared Spectroscopy (fNIRS)/Diffuse Optical Tomography (DOT)-Based Neuroimaging in the Everyday World

Human neuroscience and neurotechnology are in the process of significant transformation, moving from conventional laboratory settings toward embracing the complexity of natural environments.^1^^–^^5^ This shift to an ecologically valid depiction and decoding of human brain function will provide new scientific insights and breakthroughs in our understanding of neuronal development, health, and aging and drive translation in medicine, psychiatry, and brain–computer interfacing. However, substantial interdisciplinary challenges still need to be overcome. Naturalistic settings are difficult to study with conventional methods, sensors, and parametric experimental paradigms currently available. To better understand and deal with the complex interactions among the brain, the body, and the environment, we need unobtrusive and integrative multimodal platforms that can continuously monitor the embodied brain^6^^,^^7^ to discover and model the intertwined relationships among physiology, behavior, and cognition in everyday settings.

Thanks to the technological progress of the last years,^8^^,^^9^ wearable fNIRS and high-density diffuse optical tomography (HD-DOT) offer a pathway to continuous everyday brain monitoring. Recent HD-DOT studies have achieved the same spatial resolution of functional activity on the surface of the brain (cortex) as the latest fMRI-based Human Connectome Parcellation scheme,^10^^,^^11^ enabling connectivity analysis or decoding performance comparable with fMRI.^12^^–^^16^ However, natural behavior and motion create unique methodological challenges in fNIRS and DOT. It is non-trivial to distinguish evoked neuronal activity from complex systemic physiological activity^7^^,^^17^^–^^19^ and motion artifacts or to perform multimodal co-modulation analysis with electroencephalography (EEG), magnetoencephalography (MEG), optically pumped magnetometry magnetoenceophalography (OPM-MEG), or other multivariate signals of physiology or behavior.

One way of addressing these challenges is to leverage recent advances in classical and deep learning (ML/AI) to better isolate neuronal components from confounding physiological variance, reduce artifacts, and build generalizable and context-sensitive methods for brain imaging and decoding. ML/AI can explain variance and improve contrast by uncovering complex patterns and relationships among multimodal signals from the brain, physiology, and behavior, for which no straightforward analytical models exist. Although machine learning (ML) has transformed numerous scientific fields, its impact on fNIRS—let alone DOT—has only just begun.^20^ Consequently, there is both a growing need and great promise for analytical tools that converge the transformative potential of wearable fNIRS/DOT combined with multimodal ML-driven signal processing methods (see recent reviews on deep learning for fNIRS^20^^,^^21^ and ML for multimodal fNIRS-EEG sensor fusion^22^^,^^23^ for an overview of the recent progress and remaining limitations). In this tutorial, we introduce the Cedalion toolbox, a comprehensive suite for reproducible, state-of-the-art fNIRS analysis in channel and cortical image space (i.e., DOT) that supports multimodal signal analysis and facilitates the development and application of ML and AI tools.

### 1.2 Moving Beyond the State of the Art

**Fig. 1:**
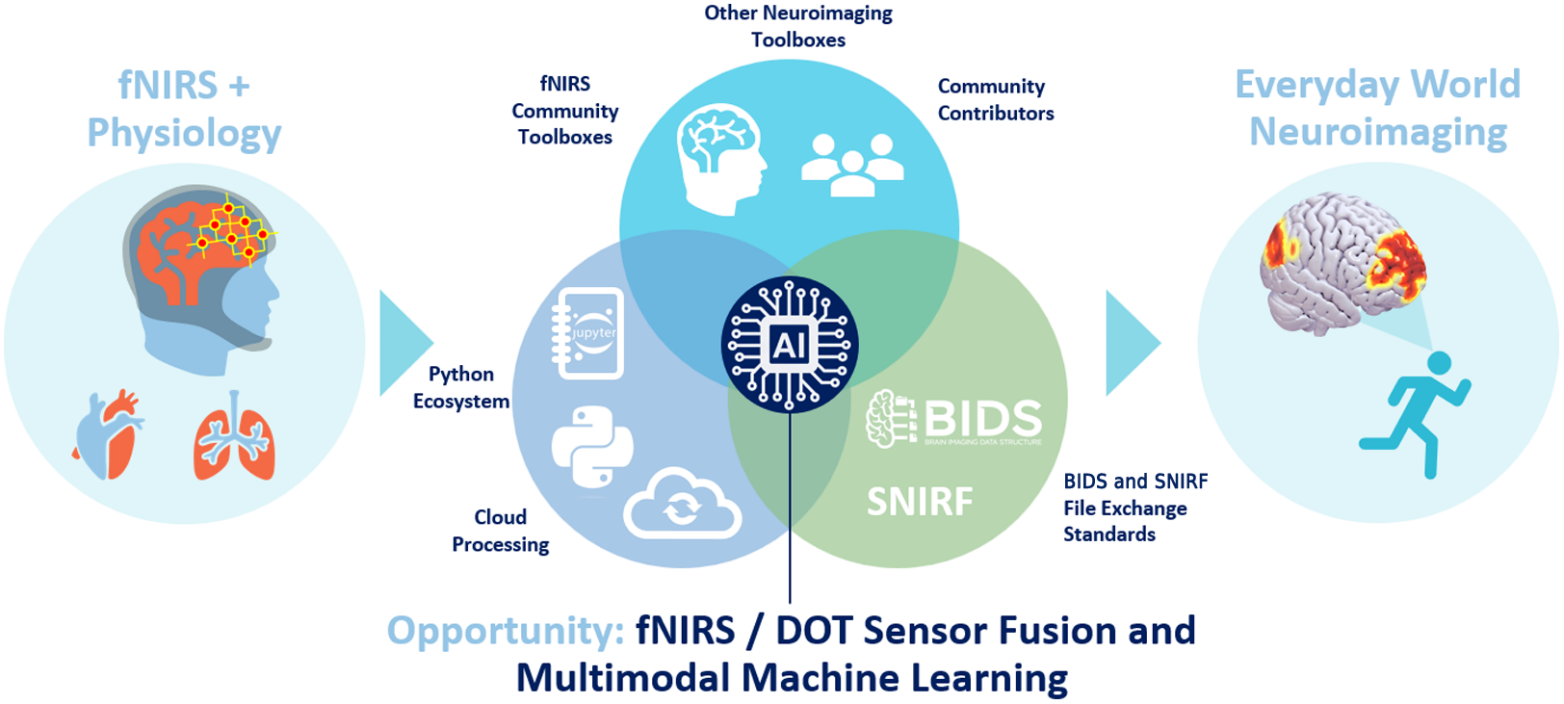
Key architectural considerations and motivation of the Cedalion toolbox. Cedalion brings together community contributions, file exchange standards, high performance computing (HPC), and cloud processing within a Python framework for advanced data-driven multimodal fNIRS/DOT analysis toward neuroimaging in the everyday world.

Current analytical toolkits for fNIRS and DOT—such as Homer2/3,^24^ AtlasViewer,^25^ Brain AnalyzIR,^26^ NeuroDOT,^27^ and NIRStorm^28^have mastered conventional signal processing of data from structured paradigms. Although powerful, these platforms are predominantly MATLAB-based and offer limited capabilities for multimodal neuroimaging with advanced ML. In recent years, there has been a clear trend toward Python-based development for ML applications.^29^^–^^31^ Python is open-source and presents unique advantages for toolbox extensibility and integration with multimodal and ML-driven analytical workflows. Beyond its individual libraries, Python functions as a common integration layer—a glue language whose shared ecosystem of data structures, interoperable packages, and interactive notebook environment allows otherwise independent components to be freely composed into end-to-end pipelines. Cedalion capitalizes on the Python ecosystem to provide (see also Fig. 1) the following:

• direct ML integration with widely used libraries such as scikit-learn and PyTorch, enabling cutting-edge fNIRS/DOT processing and Machine Learning within a unified pipeline
• compliance with standards such as SNIRF^32^ and BIDS,^33^ ensuring compatibility and interoperability with other neuroimaging modalities and community tools, including the existing fNIRS/DOT MATLAB toolboxes
• direct integration with established toolkits for neuroimaging and physiological signals, including MNE-Python (EEG/MEG),^31^ NeuroKit2 (EDA, ECG, and EMG),^34^ and Nipype (fMRI workflows),^35^ facilitating seamless multimodal analysis
• robust, intuitive data structures built on Xarray and Pandas, using multidimensional labeled arrays with physical units for easy data handling and minimizing indexing errors
• advanced visualization for direct inspection of results in brain- or parcel space, improving (neuro)physiological interpretability of high-dimensional data
• integration of Jupyter notebooks for combining code, documentation, and results in a single interactive environment. Hosted execution environments (e.g., Google Colab) allow entire analyses to run in the cloud without local installation to lower barriers to adoption and to greatly improve reproducibility of results by the scientific community.

Cedalion combines these general architectural considerations with established core fNIRS/DOT functionality from Homer3/Atlas Viewer, and significantly expanded the following features:

1. anatomical head models and image reconstruction: support for individual anatomies and template-based head models, functional atlases/parcellations, accurate photogrammetric sensor co-registration, forward-model photon simulation, and several variants of regularized DOT image reconstruction (see Secs. 2.1, 2.2, and 2.5)
2. signal processing and general linear model (GLM) analysis: state-of-the-art preprocessing methods for fNIRS and DOT data, including channel quality metrics, artifact rejection, filtering, detrending, resampling, modified Beer–Lambert law conversion, and fully customizable GLM analysis (see Secs. 2.3 and 2.4)
3. ML and data-driven analysis: functionality for multimodal data fusion, signal decomposition, and single-trial decoding; containerized execution on scientific computing clusters for performance-intensive model training and inference; and realistic data augmentation with synthetic hemodynamic response functions (HRF) and motion artifacts for method validation and model training (see Secs. 2.6 and 2.7).

Cedalion is developed with a community-centric philosophy that encourages broad participation and ensures contributor recognition by clear attribution mechanisms. Code contributions are credited in the function docstrings and on the documentation website. Implemented methods are linked to their source publications in both docstrings and code, giving the documentation a searchable and cross-linked bibliography. A full-text search for an author, a concept, or a BibTeX key surfaces places where the method is implemented or demonstrated, making it easy to move between a publication, its implementation, and a worked example. At runtime, every method invocation accumulates its associated citations. As demonstrated at the end of each example notebook, a consolidated reference table covering every publication relevant to the analysis can be easily generated. This makes it straightforward for researchers to properly credit all methods they used, rather than citing only the toolbox itself. This way, Cedalion aims to work as a shared framework that amplifies both the visibility and the accessibility of individual contributions.

Reproducibility and quality are maintained via version control and continuous integration pipelines that automatically test code for breaking changes and validate documentation builds. The toolbox is designed with a strong focus on reproducibility, open science principles, and reusability via a permissive MIT license. It comes with comprehensive documentation in the form of autogenerated application programming interface (API) descriptions and detailed examples in the form of Jupyter notebooks and downloadable example datasets. To maximize transparency and replicability as well as to lower the barrier to adoption, all examples and any user-published notebooks using Cedalion can be fully executed in the cloud (e.g., via Google Colab), enabling anyone to reproduce analyses without local setup. This provides the research community with a tool for publishing papers alongside fully replicable analysis pipelines run on the original data by anyone.

In this paper, we introduce the Cedalion toolbox and provide a comprehensive tutorial with seven detailed supplementary Jupyter notebook examples. Cedalion is freely available via Ref. 36. We invite the community to contribute to the project and to make it a lever for the convergence of wearable fNIRS/DOT and multimodal machine learning, toward everyday-world neuroscience and neurotechnology.

## 2 Toolbox

This section introduces the Cedalion toolbox, its architecture, data structures, and core functional packages and modules, organized into eight subsections. Each subsection is accompanied by a comprehensive tutorial that explains key elements and design choices through rendered Jupyter notebook examples (see the Supplementary Material). These notebooks can be explored in a static, rendered form, or executed interactively either locally, on a computing cluster or in the cloud https://codeocean.com/capsule/5432660/tree. By providing both code, automatic example data fetching and visualizations, the notebooks support hands-on testing and understanding of the toolbox. Readers unfamiliar with Python or its scientific computing ecosystem are referred to Appendix S0 in the Supplementary Material, which provides a self-contained introduction to the language, its core libraries, and the development tools used throughout this paper.

**Fig. 2:**
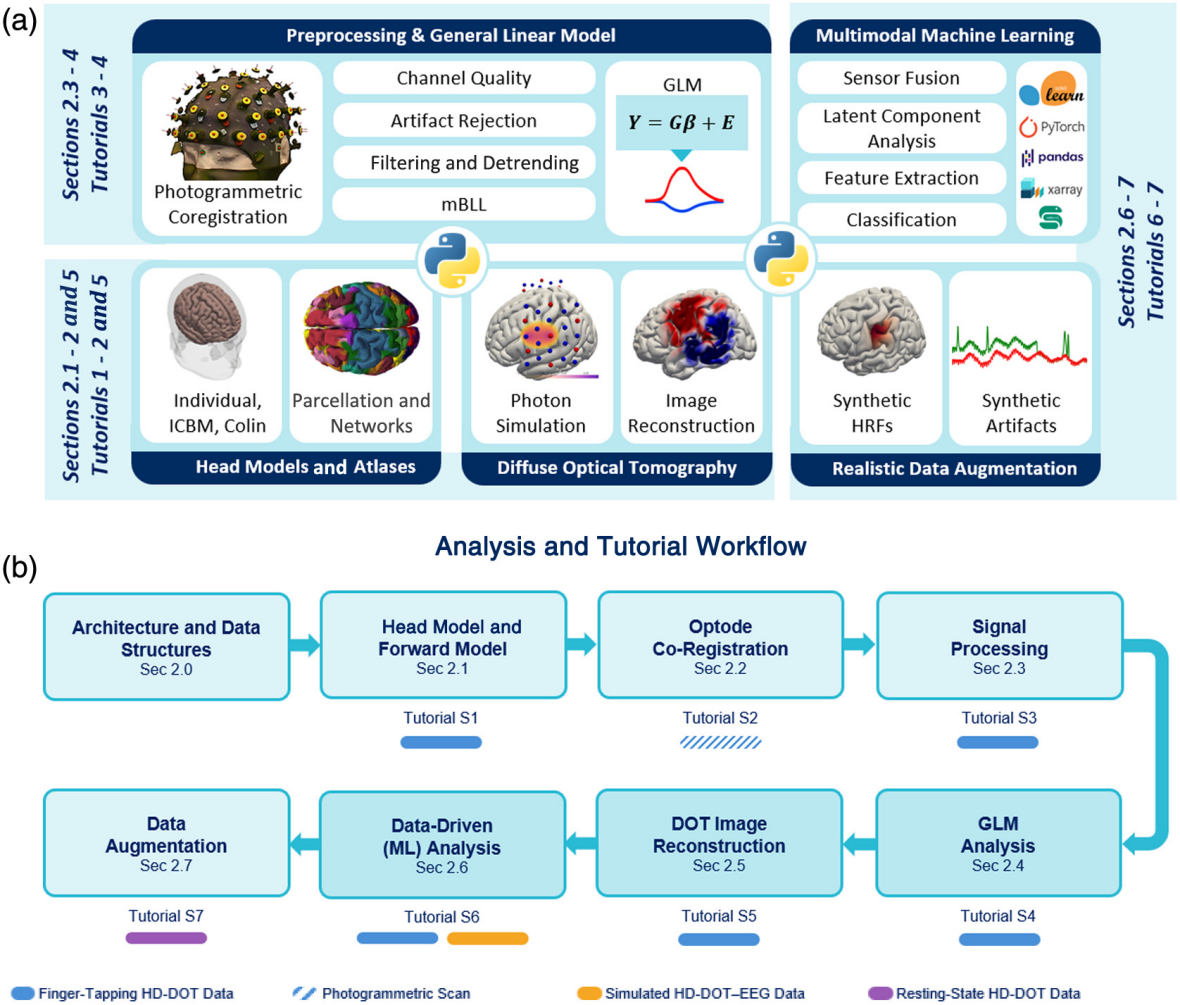
Cedalion toolbox overview and analysis workflow. (a) Functional overview of the toolbox modules and their associated manuscript sections and tutorial notebooks. Python logos symbolize the Python ecosystem serving as the integration layer connecting all modules. (b) Typical analysis trajectory, illustrating how the modules chain together in a standard fNIRS/DOT pipeline. The order of GLM analysis, DOT image reconstruction, and ML analysis is not prescriptive—the appropriate sequence depends on the research question. Colored bars indicate which dataset is used in each tutorial.

Cedalion’s functionality is organized into modules that can be used independently or combined into end-to-end analysis pipelines. Although this modular design allows users to adopt only the components relevant to their specific research question, the modules are designed to work together cohesively. To help the reader navigate the toolbox and this tutorial, here, we provide a brief overview of how the individual modules, manuscript sections, and supplementary tutorial notebooks relate to one another. Figure 2 offers a graphical overview of features and analysis workflow and serves as a visual table of contents for the paper and tutorials. The full API documentation and additional example notebooks are available at Ref. 36.

A typical fNIRS or DOT analysis in Cedalion proceeds along the following path that is representative of best practice in the community.^37^^,^^38^ Starting from reading raw recordings stored in the SNIRF or BIDS format to Cedalion’s internal data structures (Sec. 2), the user first sets up a head model and computes the forward model that relates optical measurements to underlying brain activity (Sec. 2.1, Tutorial Notebook S1 in the Supplementary Material). Optionally, accurate three-dimensional (3D) optode positions can be derived from photogrammetric head scans and co-registered to the head model to improve spatial accuracy (Sec. 2.2, Tutorial Notebook S2 in the Supplementary Material). Next, the raw data undergo signal quality assessment, artifact rejection, and preprocessing such as filtering and motion correction (Sec. 2.3, Tutorial Notebook S3 in the Supplementary Material). The preprocessed data can then be analyzed using model-driven approaches via the general linear model to estimate hemodynamic responses and identify significant activations (Sec. 2.4, Tutorial Notebook S4 in the Supplementary Material) or first projected into brain image and parcel space through DOT image reconstruction (Sec. 2.5, Tutorial Notebook S5 in the Supplementary Material). Alternatively or additionally, data-driven and machine learning methods—including independent component analysis, canonical correlation analysis, and single-trial classification—can be applied for multimodal signal decomposition and decoding (Sec. 2.6, Tutorial Notebook S6 in the Supplementary Material). Finally, Cedalion provides a data augmentation framework that allows users to inject synthetic hemodynamic responses and motion artifacts into real recordings, enabling systematic validation of any of the preceding analysis steps with known ground truth (Sec. 2.7, Tutorial Notebook S7 in the Supplementary Material).

To illustrate this workflow with concrete, reproducible examples, a shared finger-tapping motor task dataset recorded with a high-density DOT system serves as the primary example throughout Tutorials S1, S3, S4, S5, and parts of S6 in the Supplementary Material. This dataset is loaded with a single function call and is carried through from head model construction and forward modeling, through preprocessing and quality assessment, to GLM analysis, image reconstruction, and finally classification—demonstrating how the same recording progresses through each stage of the pipeline. Tutorials S2, S6 (in part), and S7 in the Supplementary Material complement this with additional datasets chosen to highlight specific capabilities: a photogrammetric scan for optode co-registration of the finger tapping dataset, simulated multimodal fNIRS–EEG finger tapping data for demonstrating cross-modal decomposition methods, and a resting-state recording for data augmentation. Together, the seven tutorials cover the full breadth of the toolbox.

### 2.1 Architecture and Data Structures

This section describes how Cedalion organizes fNIRS data in memory and how this representation simplifies writing and composing analysis code. The key idea is straightforward: the data structures are arrays with named axes, labeled entries, and physical units. Readers unfamiliar with the underlying Python libraries may find the primer in Appendix S0 in the Supplementary Material helpful for unfamiliar terms.

**Fig. 3:**
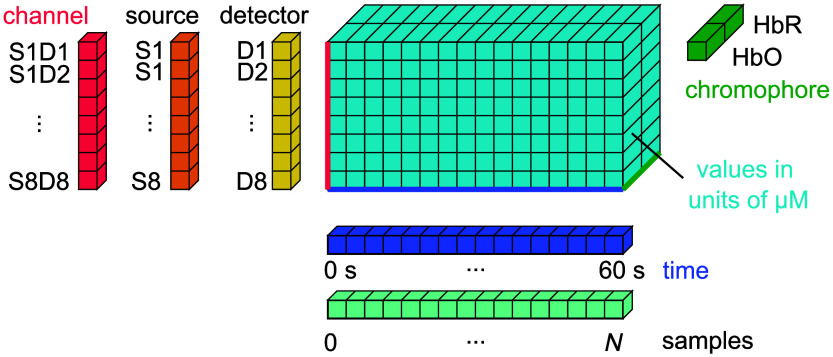
Visualization of the array layout of multidimensional time series with corresponding coordinates and units. The example has three dimensions with multiple coordinate axes per dimension (i.e., time and sample coordinates across the time dimension). Values carry physical units (i.e., micromolar) that allow automatic consistency checks and conversion.

The toolbox primarily processes sampled multivariate time series, i.e., data with multiple values per time point. Examples include wavelength-dependent NIRS measurements, chromophore concentrations across brain regions, or 3D accelerometer data. Such data fit naturally into multidimensional arrays, with axes representing quantities such as time, channel, and signal component (e.g., wavelength or chromophore). Cedalion builds on two established Python libraries for this purpose. NumPy^39^ provides the underlying numerical arrays and supports efficient array operations such as indexing, broadcasting, and reductions. Xarray^40^ extends NumPy arrays with named dimensions and labeled coordinates—for example, timestamps along the time axis, channel labels such as “S1D1” along the channel axis, and chromophore names such as “HbO” and “HbR” along the chromophore axis. Crucially, these labels stay aligned with the data through reshaping operations. Xarray refers to such labeled arrays as DataArrays, and Cedalion uses them as its primary container for time series and the associated metadata as shown in Fig. 3.

Using labels rather than numerical positions makes analysis code easier to read. In the following example, x_numpy and x_xarray denote two arrays storing amplitude time series. Time varies along the second dimension and each line calculates the time-averaged amplitude:

**Table:** 

avg_amplitude = x_numpy.mean(1)
avg_amplitude = x_xarray.mean(“time”)

Because DataArrays behave like NumPy arrays, they work directly with libraries that expect NumPy input. Common ML libraries (e.g., scikit-learn and PyTorch) operate on multidimensional array inputs, making integration straightforward.

The multidimensional layout with labeled dimensions aligns well with typical fNIRS transformations that modify some dimensions while leaving others intact. For instance, applying the Beer–Lambert law replaces the wavelength dimension with the chromophore, while image reconstruction swaps the channel dimension for a voxel, vertex, or parcel dimension. Cedalion uses canonical dimension and coordinate names, such as time, channel, and chromo, while allowing custom labels where useful. Additional flexibility comes from making toolbox functions dimension-agnostic. For instance, a bandpass filter operating along the time axis works equally well on a single-channel amplitude series or a multi-channel, multi-chromophore concentration series.

Cedalion uses the same labeled-array approach for non-time series data. For example, digitized 3D optode positions are stored alongside coordinates that specify optode labels and types. Information about different coordinate reference systems is encoded in dimension names to prevent errors related to mismatched reference systems (see S1 – Tutorial Notebook 1: Head Models and Forward Modeling in the Supplementary Material). In addition, Xarray’s support for physical units helps avoid common mistakes due to unit inconsistencies.

In the example below, the amplitudes array contains an amplitude time series and the array geo3d stores optode coordinates in centimeters. The function cedalion.nirs.channel_distances infers from amplitudes which optode pairs form channels and uses their coordinates from the geo3d array to calculate their distances also in centimeters. Element-wise comparisons with a quantity in different units (here millimeters) produce the boolean array is_short, which is then used to select only short-distance channels from the amplitudes time series. Unit conversion (mm ↔ cm) is automatically taken care of.

**Table:** 

distances = cedalion.nirs.channel_distances(amplitudes, geo3d)
is_short = distances <= 15 * cedalion.units.mm
short_channel_amplitudes = amplitudes.sel(channel=is_short)

In Cedalion, different categories of data (e.g., time series and coordinates) are represented as labeled multidimensional arrays, making them indistinguishable to Python’s type system. To compensate, Cedalion declares the expected layout of each array (its dimension names and coordinates) using type annotations and validates this layout at runtime. To make these layouts explicit in function signatures, Cedalion defines type aliases such as NDTimeSeries for time-series DataArrays and LabeledPoints for DataArrays holding optode and landmark positions. Xarray also allows attaching category-specific methods directly to a DataArray. Cedalion uses this feature selectively. For example, the attached function geo3d.points.apply_transform applies coordinate-system transformations on arrays of 3D positions. However, the overall architecture of the toolbox primarily follows a functional design. Most functionality is implemented as stand-alone functions organized into topical subpackages (see Tables 2–6). These functions take DataArray objects as input, with type hints indicating the category of data expected. Parameters representing a physical quantity must carry an explicit unit. Comprehensive docstrings accompany all functions to document their expected inputs, outputs, behavior, and scientific references and form one pillar of the toolbox’s documentation.

A typical analysis produces several related objects from different categories (e.g., time series, optode coordinates, and metadata). Cedalion groups these into a recording container, whose structure closely mirrors the SNIRF file format.^32^ When reading a SNIRF file, Cedalion populates a recording instance with data in the following attributes: .timeseries, .geo3d, .stim, .aux_ts, and .meta_data. Conversely, cedalion.io.write_snirf exports a recording container back into a SNIRF file. Continuous wave (CW), frequency-domain (FD), and time-domain (TD) fNIRS data are all supported according to the SNIRF specification.

Table 1 provides an overview of the common array layouts and their corresponding fields in the recording container.

**Table 1:** Common array layouts and coordinates used in Cedalion. For brevity, not all coordinates are always shown. Note that multiple coordinates per dimension are possible. Italic rows expand the SNIRF specification.

Data type	Layout	Coordinates	Units
Raw amplitudes or optical densities →rec[“amp”] →rec[“od”]	time × channel × wavelength	Time: [0 s, 0.1 s, 0.2 s,...]; channel: [S1D1, S1D2,…]; source (dim: channel): [S1, S2,...]; detector (dim: channel): [D1, D2,...]; wavelength: [760 nm, 850 nm]	V or unitless
Concentration changes in channel space →rec[“conc”]	time × channel × chromo	Time: [0s, 0.1s, 0.2s,...]; channel: [S1D1, S1D2, …]; chromo: [HbO, HbR]	µM
Stimuli →rec.stim	pandas.DataFrame		
Coordinates →rec.geo3d	label × 3D pos	Label: [S1, D1, Nz, Cz,..]	mm
Accelerometer →rec.aux_ts[“accel”]	time × axis	Time: [−10 s, …, 0 s, …, 30 s]	V or $m/s^{2}$
*Concentration changes in image space* →rec[“img”]	*time × (vertex|voxel) × chromo*	*Time: [0 s, 0.1 s, 0.2 s,...];* *vertex: [0,1,2,3,...];* *chromo: [HbO, HbR]*	μM
*Concentration changes in parcel space* →*rec[“parcel”]*	*time × parcel × chromo*	*Time: [0 s, 0.1 s, 0.2 s,...];* *parcel: [somatomotor A, visual B, …];* *chromo: [HbO, HbR]*	μM
*Epoched concentration changes* →*rec[“epochs”]*	*trial × reltime × (channel|vertex|parcel) × chromo*	*Reltime: [−10 s, …, 0 s, …, 30 s];* *channel: [S1D1, S1D2, …];* *chromo: [HbO, HbR];* *trial: [FingerTapping/Left, FingerTapping/Right, …];* *subject (dim: trial): [S001, S002]*	μM
*Boolean masks, e.g.*, *SNR or motion* →*rec.masks[“snr”]*			*Unitless (Boolean)*
*Headmodel* *rec.headmodel*	*cedalion.dot.TwoSurfaceHeadModel*		

By building its data structures around the SNIRF and BIDS standards, Cedalion stays compatible with other toolboxes and platforms across different stages of channel-space analysis. Users can process their data in a modular way, importing and exporting to SNIRF files in between steps, if needed. However, SNIRF was primarily designed for raw fNIRS time-series data in channel space with only a few provisions for derived data. Certain fields in recording, such as .head_model, .masks, ._aux_obj, or time series in image space, have no equivalent in the SNIRF specification. We are working with the SNIRF maintainers and the SfNIRS standardization committee on solutions to improve the standardized exchange of processed data in the future.

Table 2 shows a general overview of the data structure and in/out (I/O)-related packages in Cedalion. The cedalion.dataclasses package contains classes and functions for creating time series and geometric data. The cedalion.data package provides seamless access to example datasets for fNIRS and HD-DOT, along with head models and precomputed forward model results. These datasets are hosted online. They are versioned, get cached after download, and updated when the dataset changes. To the user, they are loadable with a single command, such as:

**Table:** 

rec = cedalion.data.get_fingertappingDOT()

Simple access to the required data lets users easily run example notebooks locally or in the cloud and quickly experiment with new implementations or analyses.

**Table 2:** Core functions for data structures and I/O. Not all subpackages are listed for brevity.

Path	Description	Ref.
cedalion	Top-level toolbox	
|—.**dataclasses**	Data classes used throughout Cedalion	
|—.**typing**	Type aliases, in particular, for arrays with data schemas	
|—.**physunits**	Physical units, built on pint_xarray’s unit registry	
|—.**xrutils**	Utility functions for Xarray objects	
|—.**data**	Cedalion data, example datasets, and utility functions	
|—.**io**	**Modules for data I/O**	
|—.snirf	Reading and writing SNIRF files	32
|—.bids	Reading BIDS data	33
|—.anatomy	Reading and processing anatomical data	
|—.forward_model	Reading and writing forward model computation results	
|—.photogrammetry	Reading photogrammetry output file formats	
|—.probe_geometry	Reading and writing probe geometry files	

#### 2.1.1 Pipelines

**Fig. 4:**
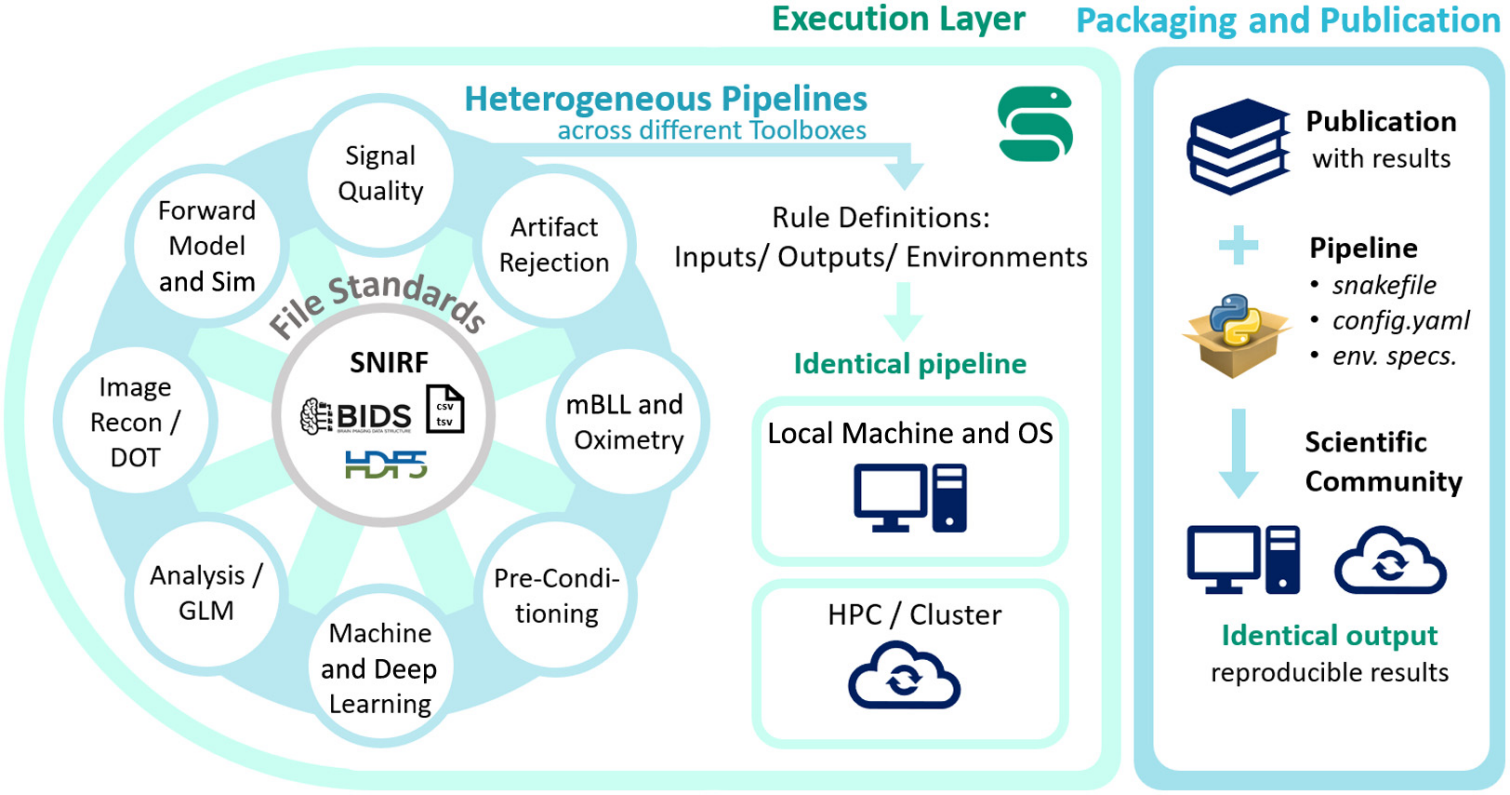
Pipelines in Cedalion. Through integration of environment specification with Snakemake- and SNIRF/BIDS-standardized file exchange, heterogeneous processing steps across community toolboxes can be executed identically on local machines and clusters via rule-based pipeline configuration and definition (snakefile, config.yaml).

There is substantial diversity in fNIRS analysis pipelines.^38^ With increasing complexity of a project, an analysis may involve multiple processing steps, draw on methods from several toolboxes in different scripting languages, and require substantial computing resources. One of our development goals is to help users assemble, run, and share complete analysis pipelines with as little friction as possible. To accomplish this, we are integrating with Snakemake,^47^ an established workflow management system, that coordinates the execution of multi-step analyses, tracks the dependencies among steps, and manages their software environment. In Snakemake, an analysis is described as a sequence of processing steps, each declaring its input and output files. From these declarations, the system automatically determines the order in which steps must run and which steps can run in parallel. Specified in this way, the complete processing pipeline can be executed from a single command. Iterative development becomes faster, because Snakemake re-executes only steps whose inputs have changed. Each step can declare its own runtime environment (e.g., specific Python, MATLAB, or library versions). Steps with conflicting dependencies can coexist in the same pipeline. Finally, the same workflow definition runs unchanged on a laptop or a high-performance computing cluster (see Fig. 4).

For Cedalion, this integration entails bundling existing functionality into larger, reusable pipeline building blocks. Each block encapsulates a coherent stage of an analysis while still exposing the flexibility users need. Relevant parameters can be modified by the user via text-based YAML configuration files. For example, in a preprocessing block, users need flexibility in selecting which artifact correction methods to apply, in what order, and with which parameter settings. By encapsulating complexity and offering a configuration–file interface that is simpler than Python code, these building blocks make it easy to express and adjust such choices for otherwise fixed pipelines and for users who are new to Python and its ecosystem. At the same time, advanced users can leverage Snakemake’s flexibility to define workflows that combine these building blocks with other tools, enabling them to design the complex processing schemes their projects require.

Finally, we believe that this design will make it easier to package and publish entire analysis pipelines and not just descriptions of them. We expect this to lower the barrier for others to reproduce, adapt, and extend published analyses. This, in turn, will help improve the overall reproducibility and transparency in fNIRS research.

### 2.2 Head Models and Forward Modeling

#### 2.2.1 Overview

Cedalion incorporates and expands established methods from AtlasViewer^25^ for head model generation, forward modeling, and image reconstruction/diffuse optical tomography. The main interface to this functionality is provided by classes in the cedalion.dot package, whereas assisting geometric calculations are provided in cedalion.geometry.

The cedalion.dot.TwoSurfaceHeadModel class supports the handling of segmented magnetic resonance imaging (MRI) head scans, the construction of triangulated brain and scalp surfaces, the derivation of voxel-vertex mappings, and the coordinate transformations between voxel and scanner spaces.

**Fig. 5:**
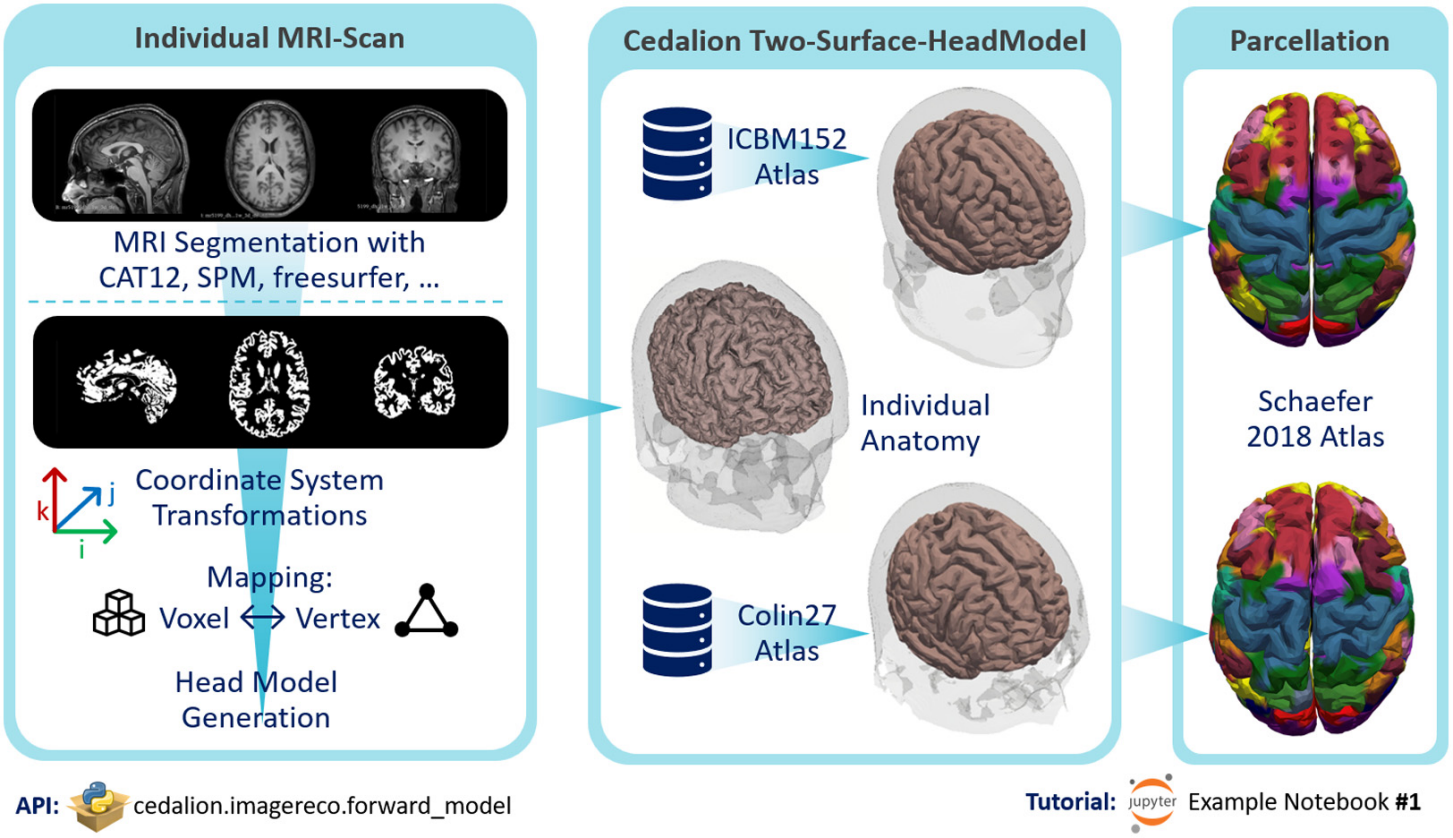
Head models in Cedalion. Segmentation masks from individual MRI scans as well as existing Atlases (ICBM152 and Colin27) can be used for head model generation, coordinate transformation, voxel–vertex mapping, and parcellation. Head models collapse volumetric tissue sensitivities on representative scalp and brain surfaces (see also Sec. 2.6).

Cedalion can process individual MRIs if available but also provides head anatomies based on the ICBM-152^48^ and Colin27^49^ atlases, including a brain parcellation scheme based on the Schaefer atlas^50^ (see Fig. 5).

Based on common fiducial points, the head model allows the registration of labeled points (i.e., existing probe geometries that comprise optodes, electrodes, or landmarks) onto the scalp surface. The reduction of the voxelized head anatomy to two surfaces simplifies the inverse problem of image reconstruction by reducing the number of unknowns and limiting reconstructed absorption changes to areas where hemodynamic changes are expected and measurable (see Sec. 2.5 for more details).

**Fig. 6:**
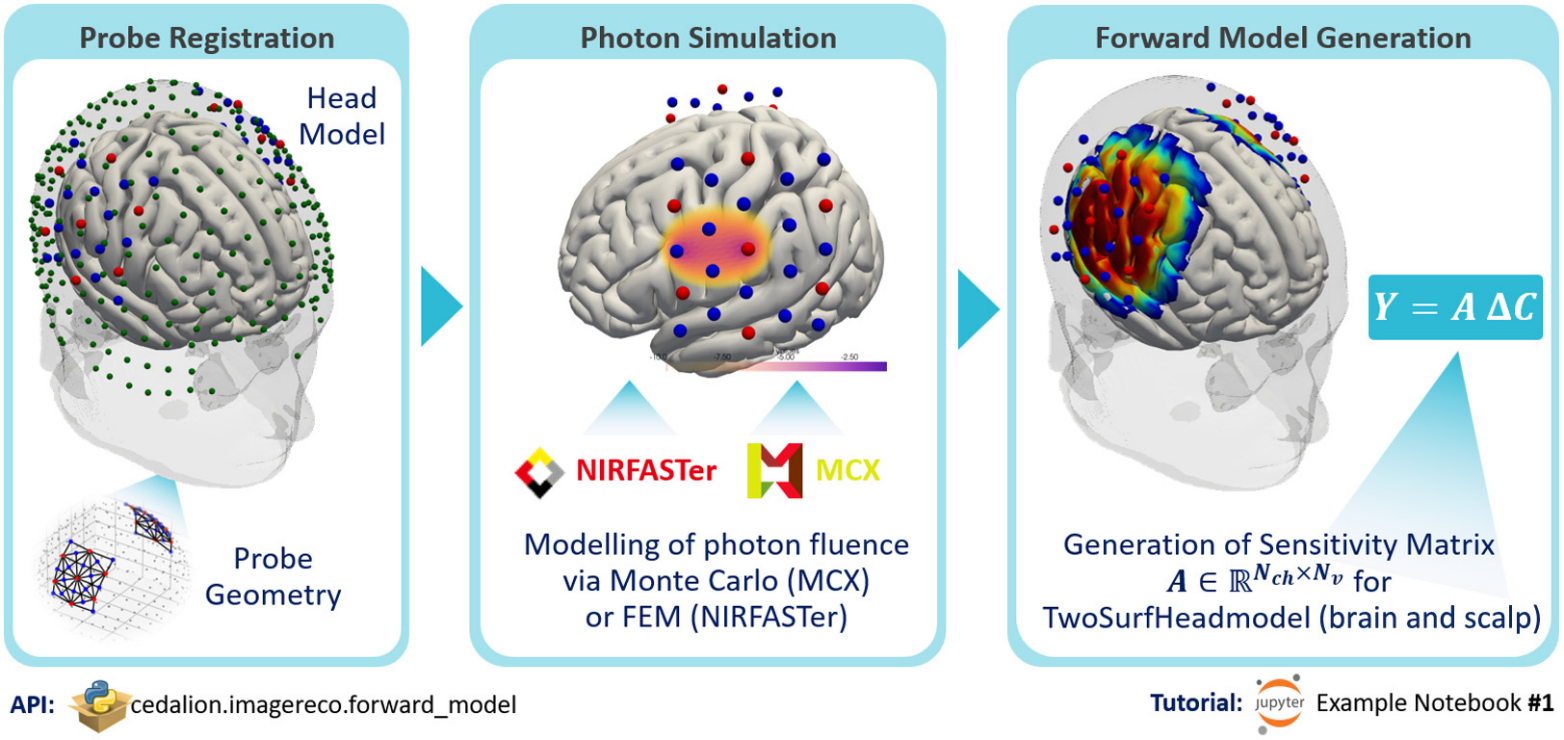
Forward modeling in Cedalion. Integration Cedalion’s head models and SNIRF probe geometries with MCX/MCX-CL and NIRFASTer enable both Monte Carlo and finite element method (FEM)-based photon propagation modeling for forward model generation.

The cedalion.dot.ForwardModel class provides methods for modeling photon migration in the voxelized and tissue-segmented head model both based on Monte Carlo simulations with MCX/MCX-CL^51^ and the finite-element method based on NIRFASTer.^52^ From the calculated photon fluences, it can then compute the sensitivity matrix A for forward modeling that maps absorption or corresponding concentration changes ΔC in brain/scalp to optical signal changes in channel space Y (see Fig. 6).

Table 3 provides an overview of the relevant packages and modules for the head and forward model.

**Table 3:** Core functions for head models and forward modeling. Not all subpackages are shown for brevity.

Path	Description	Ref.
cedalion	Top-level toolbox	
|—.**geometry**	**Modules for geometric calculations**	
|—.landmarks	Constructing the 10-10 system on the scalp surface	53
|—.registration	Registration of optodes to scalp surfaces	
|—.photogrammetry	Modules for photogrammetric sensor registration	
|—.segmentation	Functionality to work with segmented MRI scans	
|—.**dot**	**Modules for DOT image reconstruction**	
|—.TwoSurfaceHeadModel	Class to represent a segmented head	25
|—.get_standard_headmodel()	Access to Colin27 and ICBM-152 models	49 and 54
|—.ForwardModel	Class for simulating light transport in tissue	51 and 52
|—.tissue_properties	Optical properties for light transport simulation	25

#### 2.2.2 Tutorial 1

Tutorial notebook 1 (S1 – Tutorial Notebook 1: Head Models and Forward Modeling in the Supplementary Material) guides the user through the prerequisites for any image-reconstruction workflow in diffuse optical tomography: representing and transforming optode geometries, the construction of head models from segmented MRI scans, and simulating light propagation in tissue.

The tutorial notebook starts by loading optode coordinates from an example finger-tapping dataset and discusses how 3D probe geometry and channel definitions are represented. Geometric data arrives in several coordinate systems. For segmented MRI scans, voxel space (dimensionless, with axes aligned to the voxel grid) must be distinguished from scanner space (in physical units, with axes along the subject‘s right, anterior, and superior directions). Digitized optodes come in a separate coordinate system whose axis orientation is initially unknown and is recovered by an affine transformation fit to common landmark positions. Cedalion tracks coordinate reference systems explicitly to prevent accidental confusion.

Several routes for constructing a cedalion.dot.TwoSurfaceHeadModel are then demonstrated. The user has the choice to either load a standard atlas (Colin27, ICBM-152), to derive brain and scalp surfaces from a custom MRI segmentation, or to load high-resolution cortical surfaces produced by tools such as Freesurfer or CAT12. The notebook demonstrates several strategies for registering an existing probe geometry to a head model. The user can scale the head model to match measurements of the subject’s head or digitized landmarks. Alternatively, the probe geometry can be conformed to the scalp using a spring relaxation method that minimizes channel distance distortions.

Finally, the cedalion.dot.ForwardModel object is introduced. It wraps the MCX and NIRFASTer light simulations behind a common interface. Fluence distributions and the sensitivity matrix get computed. A 3D visualization of the cortical regions probed in this measurement concludes the first tutorial.

### 2.3 Photogrammetric Optode Co-Registration

#### 2.3.1 Overview

**Fig. 7:**
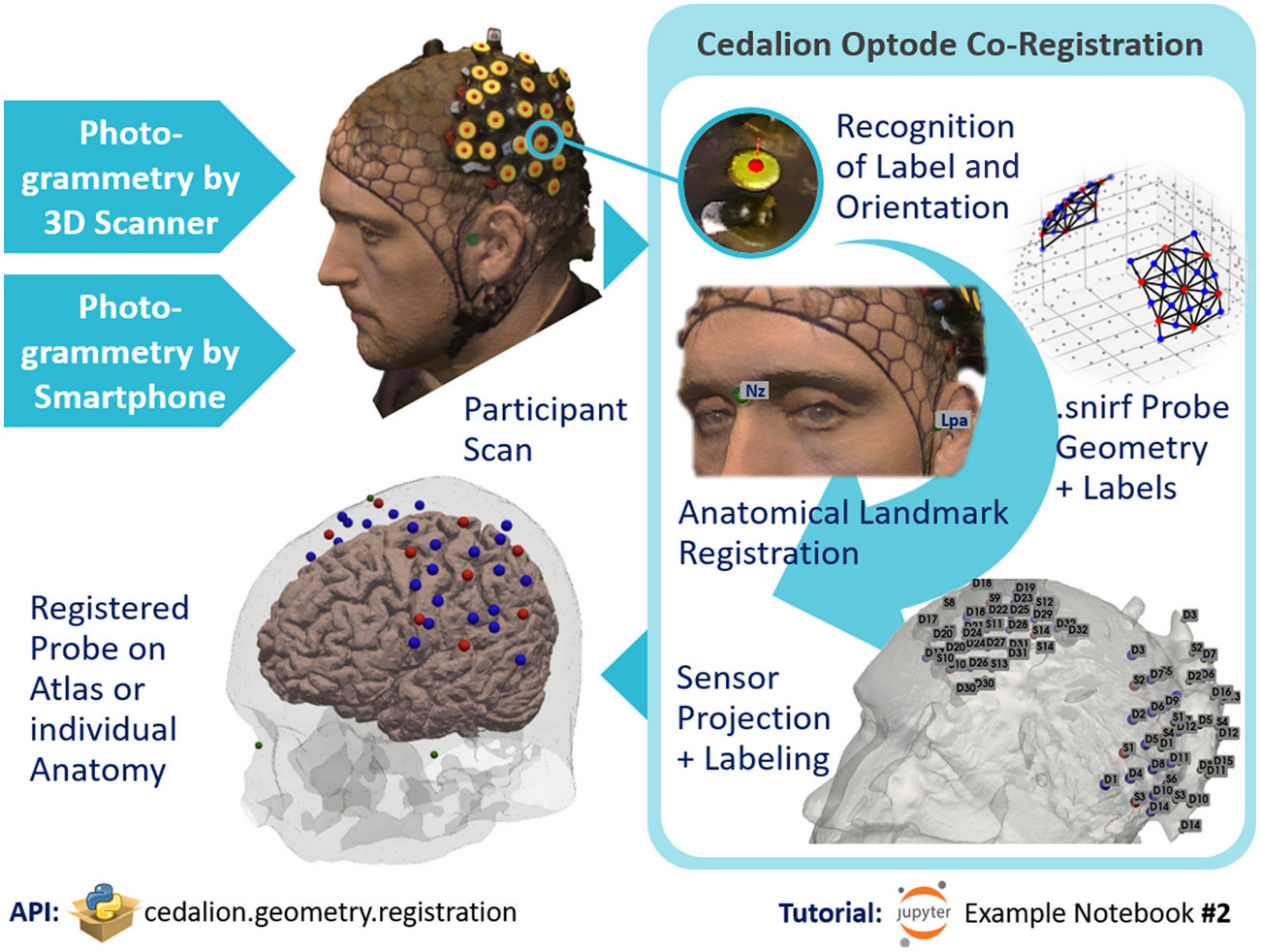
Photogrammetric optode co-registration in Cedalion. Photogrammetric scans of visually labeled optodes, either from a smartphone or a 3D scanner, can be automatically processed for optode detection, registration of orientation and position in 3D space relative to anatomical landmarks, and mapping pre-designed probe geometries with source and detector labels from SNIRF files. Enables precise co-registration of individual probe positions onto atlases or individual head geometries to reduce localization errors to anatomical brain regions within and across subjects.

The cedalion.geometry package also incorporates functionality for processing photogrammetric scans from a 3D scanner or smartphone for automatic optode digitization, labeling, and co-registration (see Fig. 7). This method has increasingly been established as a replacement for conventional manual or electromagnetic-field-based optode registration techniques. Photogrammetry provides accurate 3D positions of optodes on the subject’s scalp, which increases the spatial accuracy of fNIRS and DOT and reduces inter-subject variance.^55^^–^^–^^57^

#### 2.3.2 Tutorial 2

Tutorial notebook 2 (S2 – Tutorial Notebook 2: Photogrammetric Optode Co-Registration in the Supplementary Material) demonstrates a workflow for deriving optode coordinates from a photogrammetric head scan and mapping them onto a predefined montage. Circular colored stickers were attached to the spring tops of NIRx NIRSport2 optodes. A populated cap with such prepared optodes was scanned with a Structured-Light Photogrammetric 3D scanner (EINSTAR 3D Scanner, Shining 3D, Hangzhou, China), producing a textured triangle mesh. After loading the textured mesh, the pipeline identifies optode stickers by color and computes their surface normals. The sticker coordinates are shifted inward along the normal direction by the known optode length to find the points where the optodes touch the scalp surface. Finally, by comparing the found positions to known montage coordinates, optode labels are assigned.

The ColoredStickerProcessor class implements sticker detection by selecting vertices whose colors fall within configurable regions of the HSV color space. Furthermore, the class provides diagnostic visualizations to inspect and adjust the classification process. If automatic detection misses or misclassifies optodes, users can interactively add or remove points using the OptodeSelector class.

Furthermore, the user must select the positions of five anatomical landmarks (Nz, Iz, Cz, LPA, and RPA) on the mesh. If these are difficult to identify, the user may identify three optodes instead.

Based on these fiducial points, the known montage is aligned. An iterative closest point algorithm matches optode positions from the scan with those from the known montage, thereby assigning labels to the optodes.

Finally, two methods for storing the found optode positions are demonstrated. The first method writes a BIDS-compliant tab-separated text file, whereas the second method shows how to store the positions in a SNIRF file.

### 2.4 Signal Processing

#### 2.4.1 Overview

A great number of signal processing methods exist in the fNIRS community for signal quality assessment, artifact detection/correction, and signal modeling. Although the choice of methods and pipelines is diverse (see Ref. 38 for a recent large study investigating the choice of pipelines across 35 international teams), some approaches are increasingly adopted within the community as they have shown particular robustness and reliability across studies.

**Fig. 8:**
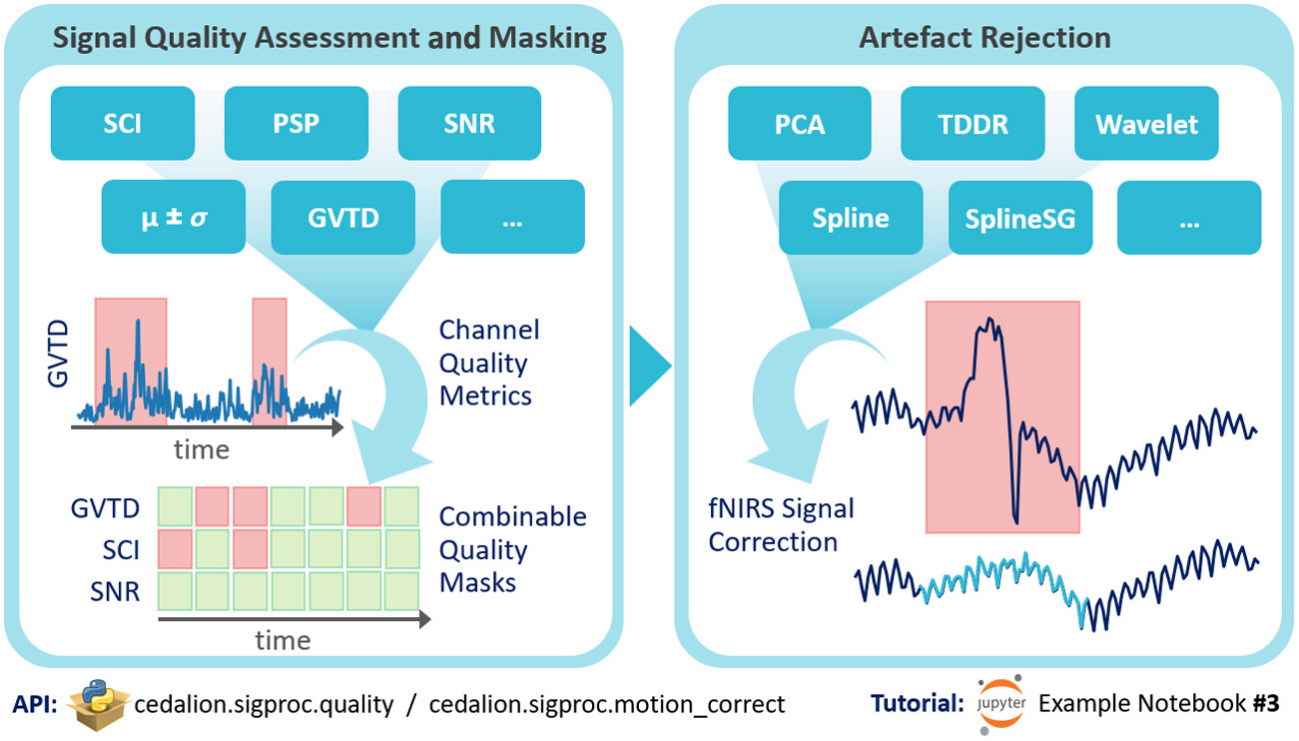
Quality assessment and artifact rejection. Established quality metrics such as SCI, PSP, and SNR can be used for the generation of multidimensional (Xarray-based) quality masks that can be flexibly combined or carried along. Widely used artifact rejection methods can clean data in an unsupervised way [e.g., temporal derivative distribution repair (TDDR), wavelet, and global variance of the time derivative (GVTD)] or via supervision from quality masks (e.g., Spline, SplineSG, and PCA).

Expanding Homer2/3’s functionality, Cedalion implements a range of these widely used methods (see Fig. 8 and Table 4) for the following:

1. signal quality assessment in cedalion.sigproc.quality, including GVTD,^58^ SNR, scalp-coupling index, and peak spectral power^59^
2. motion correction in cedalion.sigproc.motion, including TDDR,^63^ wavelet,^62^ spline,^60^ splineSG,^61^ and recursive PCA^25^
3. general linear model analysis (see Sec. 2.5).

Standard functionality for frequency filtering, epoching, or correcting data is complemented by a mechanism of masking bad segments in time series (e.g., time points or whole channels): Any quality or motion detection metric can be used to generate Boolean masks with the same array dimensions as the analyzed time series. These can be logically combined and collapsed across metrics or dimensions (e.g., time or wavelength) and carried along through the analysis pipeline in rec.masks. The following tutorial 3 provides more details on this concept.

**Table 4:** Core functions for signal processing and analysis.

Path	Description	Ref.
cedalion	Top-level toolbox	
|—.**sigproc**	**Module for processing and analyzing fNIRS signals**	
|—.**nirs**	**Module for fNIRS conversion and preprocessing** e.g., beer_lambert(), int2od(), and od2conc()	24
|—.**quality**	**Module for signal quality metrics and channel pruning**	
|—.snr()	Signal-to-noise ratio for each channel	24
|—.gvtd()	Global variance of the temporal derivative	58
|—.id_motion()	Identify motion artifacts in an fNIRS input data array	24
|—.sci()	Scalp-coupling index	59
|—.psp()	Peak spectral power	59
|—.prune_ch()	Prune channels from data using quality masks	
|—….	… and more	
|—**.motion**	**Module for motion correction of fNIRS data**	
|—.PCA_recurse()	… using recursive PCA method	24
|—.spline()	… using spline method	60
|—.splineSG()	… using splineSG method	61
|—.wavelet()	Wavelet-based motion correction	62
|—.tddr()	Temporal derivative distribution repair-based correction	63
|—….	… and more	
|—.**physio**	Methods for systemic physiology in fNIRS	
|—.ampd()	Automatic multiscale peak detection	64
|—….	… and more	
|—.**epochs**	**Module for epoching of time series based on events** e.g., to_epochs()	
|—.**frequency**	**Module for frequency-related signal processing** e.g., freq_filter() and sampling_rate()	
|—.**time**	**Module for time-related signal processing**	
|—.**models.glm**	**Module with general linear models for fNIRS data**	
|—.basis_functions	Temporal basis functions for the GLM, including gamma, GammaDeriv, Dirac, and Gaussian	65
|—.design_matrix	Functions to create the design matrix for the GLM	
|—.solve	GLM solver (including OLS and AR-IRLS)	24 and 66
|—.**math.ar_model**	**Module for autoregressive modeling**	
|—.ar_filter()	Computes and applies an AR filter on a time series	
|—….	… and more	

#### 2.4.2 Tutorial 3

Tutorial notebook 3 (S3 – Tutorial Notebook 3: Signal Processing in the Supplementary Material) walks through the workflow for loading, inspecting, and preprocessing a continuous-wave fNIRS recording of two motor tasks. It begins by examining the recording container in more detail, illustrating how it organizes fNIRS and auxiliary time series, probe geometry, and stimulus events.

Stimulus events are stored as a pandas.DataFrame to which additional functionality is attached through the custom accessor, .cd. The function rec.stim.cd.rename_events translates integer event codes into descriptive names. Users are free to choose any labels, but using a consistent naming scheme, such as “BallSqueezing/Left” and “BallSqueezing/Right,” makes later selection easier, because related events can be retrieved by matching shared parts of the label (e.g., select labels starting with “BallSqueezing” in this case).

Next, the notebook computes two channel-wise signal-quality metrics: the scalp coupling index (SCI) and the peak spectral power (PSP) ratio, both implemented in cedalion.sigproc.quality. These metrics are calculated in each channel over sliding, non-overlapping windows. For each channel and window, both the metric value and a Boolean indicating whether it surpasses a configurable threshold are computed. These Boolean masks represent periods when the signal quality, based on a single criterion, is deemed insufficient. More advanced signal quality selection rules can be formulated using logical combinations of these masks or by applying logical reduction operations across the array dimensions. A combined mask is generated, and the percentage of clean time per channel is calculated and plotted on the scalp to identify consistently low-quality channels.

The notebook then computes optical densities from raw amplitudes and applies two methods to correct for motion artifacts: TDDR and wavelet-based correction. The notebook concludes by demonstrating the effectiveness of these methods using the GVTD metric and by plotting individual time traces.

### 2.5 Model-Driven (GLM) Analysis

#### 2.5.1 Overview

The GLM^67^^,^^68^ for fNIRS explains the time series (Y) for each channel and chromophore individually as a linear superposition of regressors, some representing the hemodynamic response and others addressing nuisance effects such as superficial or physiological components and signal drifts. The regressors form the design matrix (G), and the fit determines the unknown coefficients (β) for each regressor in the presence of unmodeled noise (E).

**Fig. 9:**
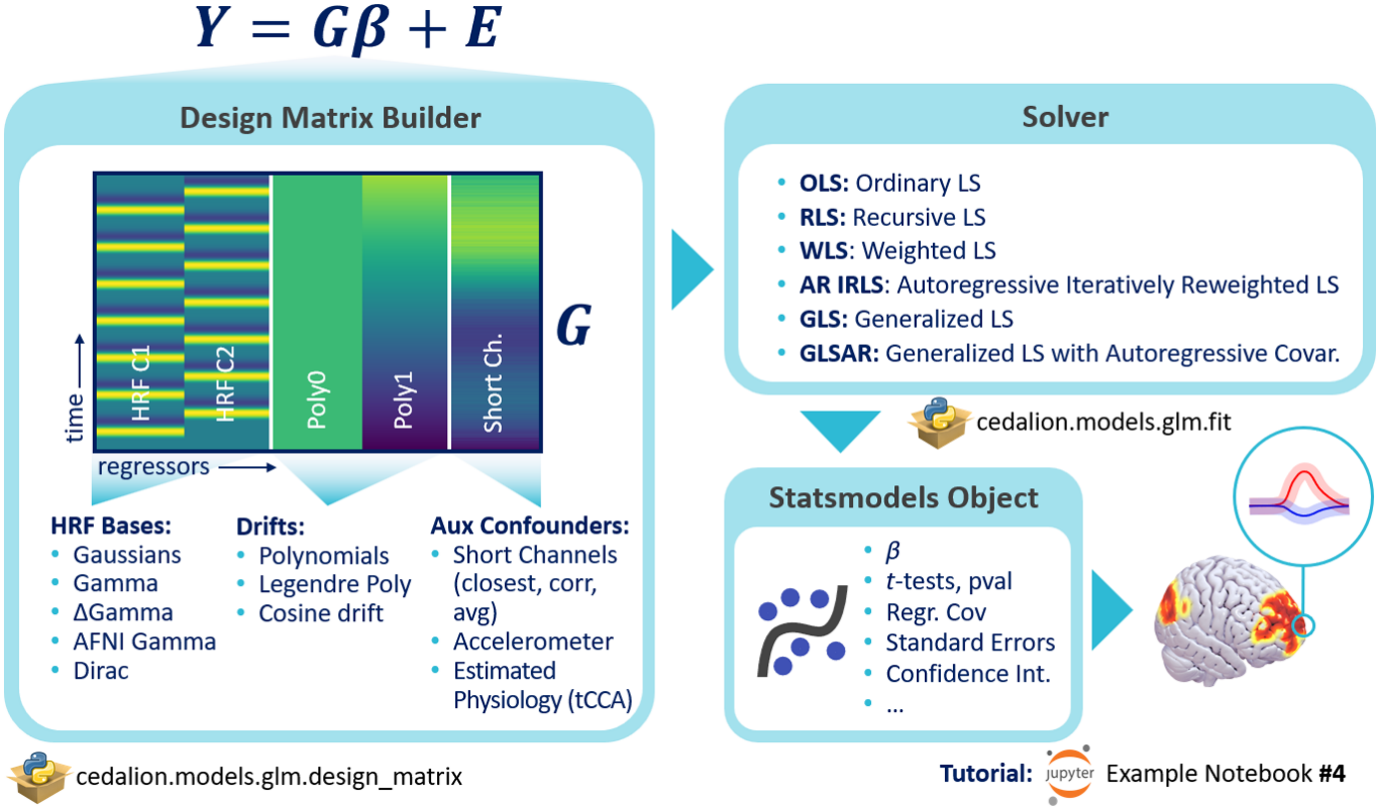
GLM in Cedalion. The design matrix builder permits flexible combination of HRF kernels, drift, and physiology regressors. A variety of solvers (from OLS to AR-IRLS) can be used to estimate the fits, provided as a statsmodels object, leveraging the statsmodels Python package for advanced statistical analysis.

Regressors can vary across channels, such as when using the nearest short channel for short-channel regression techniques. Therefore, regressors are managed by the DesignMatrix class, which distinguishes between common and channel-wise regressors, building each channel’s design matrix for the model fit. Multiple functions for creating DesignMatrix objects are available. Available nuisance regressors range from different polynomials to Fourier (cosine) basis regressors, to short-channel regressors, and to auxiliary data-driven physiology regressors, such as those derived via tCCA.^69^ Hemodynamic response regressors can be generated from paradigm-encoded basis functions, for which Cedalion offers several options including gamma functions (with or without derivatives) as well as Gaussian basis functions. By combining DesignMatrix objects, users can build models of any complexity needed to describe their data. To fit the GLM, the statsmodels library^70^ is used, giving users numerous options for robust coefficient estimation, uncertainty assessment, and statistical testing. The AR-IRLS algorithm is also available to address serially correlated noise (see Fig. 9 and Table 4).

#### 2.5.2 Tutorial 4

Tutorial notebook 4 (S4 – Tutorial Notebook 4: Model-Driven (GLM) Analysis in the Supplementary Material) illustrates a workflow for modeling the motor task dataset using a GLM. Building on the preprocessing steps from the previous notebook, optical densities are converted to concentration changes via the modified Beer–Lambert law, and a low-pass filter removes the cardiac component. Source–detector distances are computed to separate long from short channels. Only long channels are modeled, while short channels are averaged to form a nuisance regressor. The hemodynamic response is modeled as a combination of Gaussian kernels, and the model is fitted using the AR-IRLS method. The notebook illustrates how string labels for regressors and coefficients help to identify and select specific components of the fitted model. After illustrating goodness-of-fit metrics, a hypothesis test for the HRF coefficients is performed, and channels with significant activations are plotted on the scalp. The notebook concludes by extracting and visualizing the HRF along with its uncertainty.

### 2.6 DOT—Image Reconstruction

#### 2.6.1 Overview

**Fig. 10:**
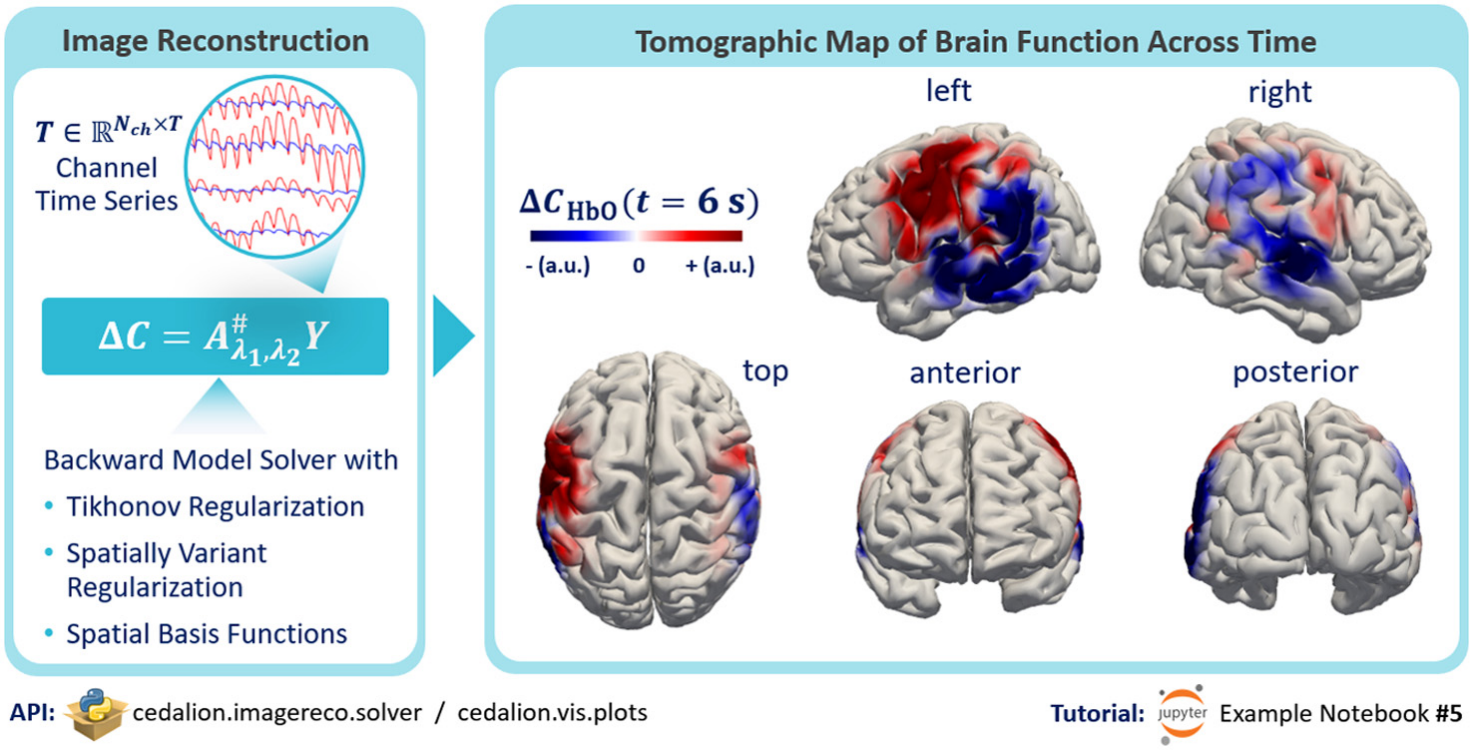
Image reconstruction in Cedalion. Forward model inversion includes options for Tikhonov (measurement noise) regularization and spatially variant regularization and use of spatial basis functions such as Gaussian kernels. Results can be visualized in still images and as GIFs across time.

Cedalion implements AtlasViewer’s surface-based image reconstruction functionality for DOT and extends it by incorporating additional regularization strategies and spatial bases.^71^ DOT using a two-surface head model reduces the dimensionality of the image space from 100 to 500k voxels to ∼10 to 50k surface vertices, thereby simplifying the inversion of the forward model. The approach maps volumetric tissue sensitivity to vertices of two representative surfaces—scalp and cortex—by attributing contributions from the scalp to the superficial surface and by pooling contributions from gray and white matter to the cortical surface. This two-surface representation is a simplification justified by the depth-dependent photon sensitivity in diffuse optical tomography: the optical sensitivity decays exponentially with depth and volumetric contributions within superficial and cortical compartments are highly correlated. The two-surface reduction is well matched to CW and typical FD HD-DOT settings with limited depth discrimination, whereas TD measurements can retain more depth information and may warrant time-resolved/volumetric reconstruction rather than a two-surface model. As shown by Boas et al.,^72^ the limited depth resolution of DOT causes partial volume effects that can be mitigated by incorporating anatomical priors. The two-surface head model used in AtlasViewer^25^ capitalizes on this property by effectively representing the main tissue classes that contribute to the measured signal while maintaining computational efficiency and anatomical interpretability. The currently implemented reconstruction methods (see Fig. 10) were designed for CW-NIRS measurements and target chromophore concentration changes. The toolbox does, however, also handle frequency-domain and time-domain NIRS data, and we hope that this existing infrastructure will support the development of reconstruction methods for absolute chromophore concentrations in the future. Table 5 provides an overview of relevant packages and modules.

**Table 5:** Core functions for DOT image reconstruction.

Path	Description	Ref.
cedalion	Top-level toolbox	
|—.**dot**	**Modules for DOT image reconstruction**	
|—.TwoSurfaceHeadModel	Class to represent a segmented head	25
|—.get_standard_headmodel()	Access to Colin27 and ICBM-152 models	49 and 54
|—.ForwardModel	Class for simulating light transport in tissue	51 and 52
|—.ImageRecon	Image reconstruction methods	25
|—.GaussianSpatialBasisFunctions	Basis functions for regularization and dimensionality reduction	71
|—.tissue_properties	Optical properties for light transport simulation	25
|—.utils	Utility functions for image reconstruction	

#### 2.6.2 Tutorial 5

Tutorial notebook 5 (S5 – Tutorial Notebook 5: Image Reconstruction in the Supplementary Material) demonstrates Cedalion’s image reconstruction capabilities to explain measured optical density changes through hemoglobin concentration changes in the brain and scalp, thereby shifting the analysis from channel space to image space and then parcel space.

In the motor task dataset used in the previous tutorials, the finger-tapping trials are epoched and block-averaged, demonstrating the flexibility of the time series data structures and yielding an HRF estimate to be reconstructed. The head model is set up, and a precomputed sensitivity matrix is loaded (computing the sensitivity is described in tutorial notebook 1).

The image reconstruction functionality is implemented in the class cedalion.dot.ImageRecon, which during construction takes the sensitivity matrix as well as measurement and spatial regularization parameters. With these options, the user can control the balance between image noise and spatial resolution and rescale sensitivities to suppress scalp-reconstructed activity.^71^ Instead of solving the inverse problem for each vertex, activations can be modeled as a combination of Gaussian spatial basis functions defined on the brain and scalp surfaces by passing an instance of cedalion.dot.GaussianSpatialBasisFunctions to ImageRecon.

Furthermore, the user can choose how to reconstruct concentration changes from optical densities: either directly, by incorporating extinction coefficients into the sensitivity matrix, or indirectly, by first reconstructing absorption changes for each wavelength and then converting these to concentration changes. The indirect approach is preferred, as it reduces cross-talk among chromophores.^71^

The function ImageRecon.reconstruct solves the inverse problem, and several ways of visualizing the results are demonstrated. The transition from channel space to image space is reflected in the returned time series array, where the channel dimension is replaced by a vertex dimension. When using a standard head model, the vertex dimension has parcel coordinates which map each brain surface vertex to its corresponding label in the Schaefer 2018 atlas with 17 networks and 600 regions of interest. Grouping vertices by shared labels makes it straightforward to average activations within each parcel, yielding a time series array in which the vertex dimension is replaced by a parcel dimension. This demonstration of shifting the analysis to parcel space concludes the notebook.

### 2.7 Data-Driven (ML) Analysis

#### 2.7.1 Overview

Being built within the Python ecosystem, Cedalion is designed for integration of advanced fNIRS/DOT analysis with state-of-the-art machine learning and deep learning libraries such as scikit-learn^73^ and PyTorch.^74^ The Xarray-based data structures map directly onto the input and output tensors used by these libraries, ensuring seamless interoperability. This enables preprocessing and feature extraction within Cedalion before passing the prepared data to machine-learning libraries for model training and inference. Cedalion can extract a range of features from fNIRS data, including statistical features, GLM-derived coefficients, and frequency-domain features based on Fourier and wavelet transforms.^75^^,^^76^

Beyond classical ML pipelines, the Xarray representation integrates also into PyTorch-based deep learning workflows, where for example, parcel/channel-informed normalization supports model development.

Looking ahead, the rapid emergence of deep learning architectures for tasks such as artifact correction and latent component analysis motivates the development of convenient APIs for deep learning model inference in future Cedalion releases. For example, the community will be able to leverage deep latent component models to obtain data-driven features for network analysis or classical ML pipelines, as well as generalized artifact-correction/detection models similar to Ref. 77.

**Fig. 11:**
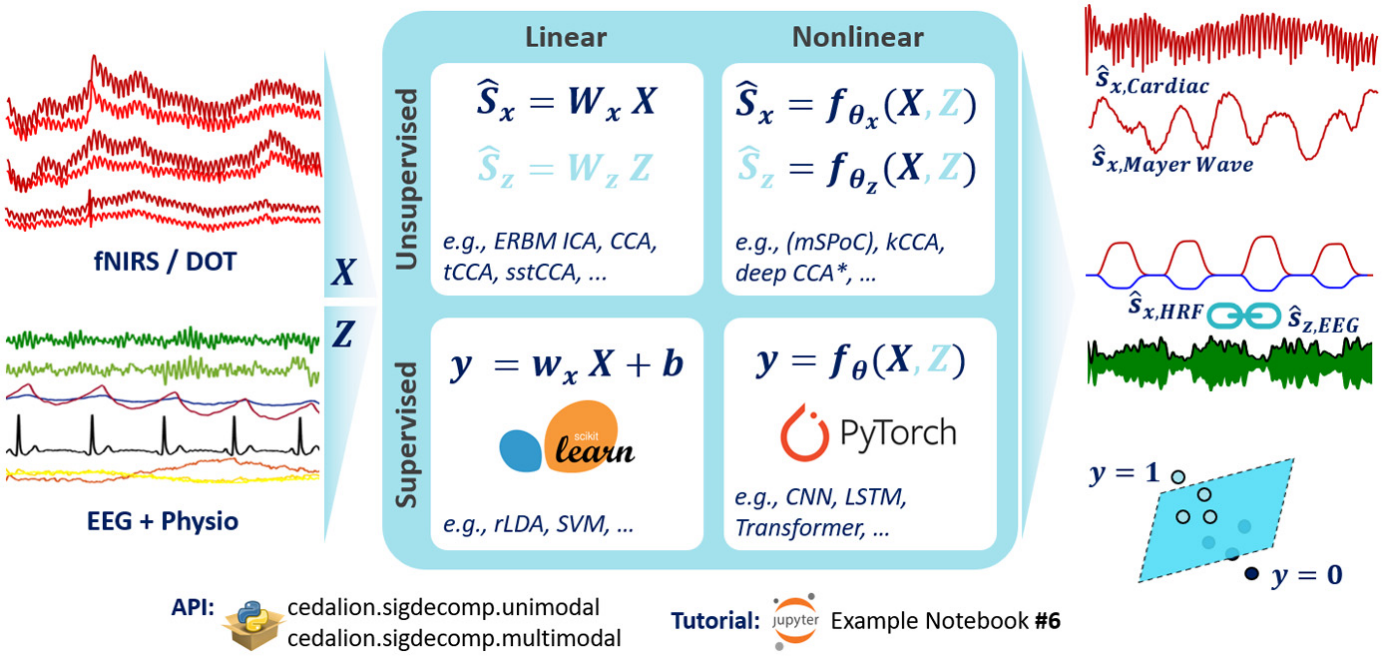
Data-driven analysis in Cedalion. The 2×2 grid organizes Cedalion’s signal decomposition and decoding methods along two axes. The vertical axis distinguishes unsupervised methods—which identify latent signal components without class labels, e.g., to separate neuronal from physiological sources—from supervised methods that learn a mapping to an explicit outcome label y (e.g., motor task versus rest). The horizontal axis distinguishes linear methods, where the mapping is a weight matrix W applied directly to the data, from nonlinear methods, where the mapping is a parametric function f_θ (e.g., a neural network). In all cases, X denotes the fNIRS/DOT signal and Z denotes a second modality (e.g., EEG or physiological signals); S^ denotes the estimated source components. Aside from direct interfaces to scikit-learn and PyTorch, Cedalion provides tailored implementations for unimodal and multimodal linear and nonlinear unsupervised decomposition, including the CCA family and (constrained) ICA.

Figure 11 maps the full landscape of data-driven methods available in and through Cedalion along two conceptual axes: whether learning is supervised (a target label y is available) or unsupervised (latent components are identified without labels), and whether the signal mapping is linear or nonlinear. The supervised quadrants are covered by Cedalion’s interfaces to scikit-learn and PyTorch described above; the unsupervised quadrants are populated by dedicated implementations in the cedalion.sigdecomp package (Table 6). This package contains several families of unimodal and multimodal methods in the domain of sensor fusion-based signal decomposition for unsupervised fNIRS/DOT analysis, see Ref. 23 for a recent review. Among them are independent component analysis (ICA)-based methods such as EBM and ERBM ICA^42^^,^^78^ and their constrained variants; canonical correlation analysis (CCA) with a range of regularization options, priors, and temporal embedding, e.g., for tCCA GLM;^69^ and (multimodal) “source power co-modulation” (SPoC) methods^43^^,^^45^ optimized for fNIRS-EEG band power analysis (see tutorial 6 in Sec. 2.7.2 for more details.

**Table 6:** Core functions for multimodal fusion, data-driven analysis, and ML.

Path	Description	Ref.
cedalion	Top-level toolbox	
|—.**sigdecomp**	**Package for signal/source decomposition**	
|—.**unimodal**	**Module for unimodal signal decomposition**	
|—.ICA_EBM()	ICA by entropy bound minimization	41
|—.ICA_ERBM()	ICA by entropy rate bound minimization (ICA-ERBM)	42
|—.spoc	SPoC algorithm	43
|—.**multimodal**	**Module for multimodal signal decomposition**	
|—.cca	Canonical correlation analysis variants (CCA, ssCCA, ElasticNetCCA, and PLS)	23,44
|—.tcca	CCA using temporal embedding (tCCA, tssCCA, and tElasticNetCCA)	23, 44
|—.mspoc	Multimodal source power co-modulation analysis	45
|—.**mlutils**	**Package with machine learning utility functions**	
|—.cv	Cross-validation techniques	46
|—.features	Functionality to extract features from fNIRS time series	

By interfacing these packages with DOT image reconstruction to a brain parcellation atlas, Cedalion enables machine learning from a structured image space based on shared coordinates across heterogeneous datasets from various subjects and studies.^79^^,^^80^

#### 2.7.2 Tutorial 6

Tutorial notebook 6 (S6 – Tutorial Notebook 6: Data-Driven (ML) Analysis in the Supplementary Material) first demonstrates how Cedalion’s multimodal source-decomposition methods recover shared neural sources from simulated fNIRS–EEG data in sim.datasets.synthetic_fnirs_eeg. A bimodal toy dataset with a controllable signal-to-noise ratio is generated, standardized, and split into train/test sets. The tutorial applies CCA to maximize the correlation of latent components from EEG bandpower and fNIRS signals to enable, for instance, neurovascular coupling analysis. It then demonstrates regularized variants—ElasticNet, Sparse, and Ridge CCA—and shows how hyperparameter choices affect performance. Temporally embedded CCA (tCCA) is then introduced to model realistic time lags caused by neurovascular coupling, followed by the multimodal source power co-modulation (mSPoC) algorithm, which computes EEG bandpower after inversion of the generative model to further improve latent component analysis performance. Correlations with known simulated sources and visual comparisons are used to evaluate how well each method recovers the underlying multimodal brain signals. Please see Ref. 23 for more details on methods and multimodal data simulation.

The second part illustrates Cedalion’s implementation of independent component analysis by entropy rate bound minimization (ICA-ERBM) for extracting physiological components from fNIRS recordings. After preprocessing a segment of the motor-task dataset, the cedalion.sigdecomp.unimodal.ICA_ERBM function decomposes 30 channels into the same number of independent sources. Ranking these sources by spectral power around 1 and 0.1 Hz reveals cardiac and Mayer-wave components, demonstrating ICA-ERBM’s ability to isolate meaningful physiological signals across different bands of the physiological power spectrum in an unsupervised way.

The third part demonstrates how Cedalion can be interfaced with scikit-learn to train a single-trial classifier. A cross-validation scheme is used and combined with short-channel regression to account for physiological noise.^46^ Here, special care must be taken to ensure that the GLM is not fitted on any data from the respective test set. After subtracting nuisance components, the time series is split into epochs from which features are extracted. The notebook demonstrates how the layout of the data changes from Cedalion’s representation of time series to samples expected by scikit-learn. In this conversion, coordinate data are preserved which allows to trace each feature back to its origin. At the end of the notebook, an LDA classifier is trained, and the output performance and feature importances are assessed.

### 2.8 Data Augmentation

#### 2.8.1 Overview

**Fig. 12:**
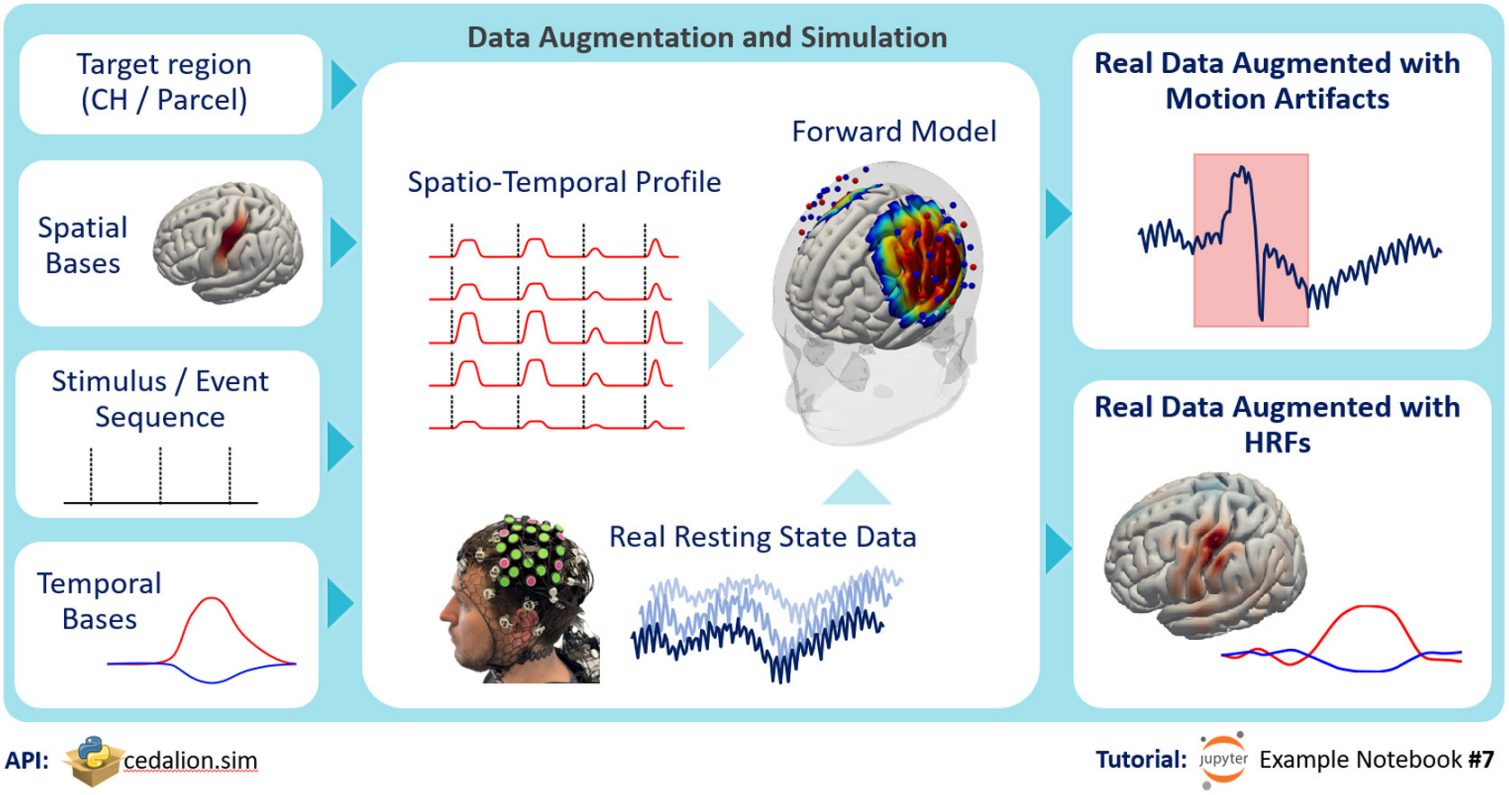
Modules for synthetic data augmentation. Realistic hemodynamic response functions on the cortex can be generated from customizable spatio-temporal bases and event sequences. Forward modeling enables projection to and addition of HRFs to real resting state background data in channel space.

Development of conventional and data-driven processing methods often requires ground truth activation or artifacts for method validation or to increase the diversity of data for training of larger ML/DL models. The cedalion.sim simulation package (see Fig. 12 and Table 7) provides structured functionality to add an expandable range of synthetic motion artifacts and synthetic hemodynamic responses to real fNIRS/DOT data. Using the forward model, spatio-temporal hemodynamic response functions can be injected at user-defined brain coordinates and projected to channel space where the activation can then be added to real fNIRS recordings to test a method’s reconstruction or classification performance with known ground truth (see tutorial 7 in Sec. 2.8.2 and Ref. 80 for more details).

**Table 7:** Core functions for data simulation and augmentation.

Path	Description	Ref.
cedalion	Top-level toolbox	
|—.**sim**	**Package for data augmentation with synthetic ground truth**	
|—.synthetic_artifact	Functions for generating synthetic artifacts in fNIRS data	80
|—.synthetic_hrf	Functions for augmenting data with synthetic hemodyn. responses	
|—.datasets	Functions for generating entirely synthetic datasets	

#### 2.8.2 Tutorial 7

Tutorial notebook 7 (S7 – Tutorial Notebook 7: Data Augmentation in the Supplementary Material) explains how fNIRS datasets may be systematically augmented with synthetic activations or artifacts using pre-defined basis functions.

The package cedalion.sim.synthetic_hrf provides functionality for creating simulated hemodynamic responses. The notebook processes a resting state dataset recorded with a NinjaNIRS 2022 device covering the whole head from Ref. 71. The Colin27 head model is set up, and precomputed sensitivities are loaded. Using the build_spatial_activation function, concentration changes with a Gaussian spatial profile are generated. The scale and intensity parameters determine the concentration change at the center and how rapidly the activation decays with geodesic distance along the convoluted brain surface. Using the forward model results, the activation in image space is translated into channel space. The build_stim_df function generates stimulus events, with random onset times and durations. Combined with a chosen hemodynamic response function (using the same options available for the GLM), this defines the temporal profile of the activation. The produced time series contains, for each channel, the change in optical density caused by the synthetic activation. This synthetic component is then added to the resting-state dataset. By reconstructing the augmented time series and comparing the reconstruction result to the ground truth, the effectiveness of the image reconstruction can be evaluated.

To assess the impact of motion artifacts on an analysis or to evaluate the effectiveness of artifact-removal algorithms, functions from cedalion.sim.synthetic_hrf augment time series with simulated artifacts. Baseline shift and spike artifacts are inserted at random time points and channels, after which the TDDR + wavelet artifact-removal procedure from tutorial 3 is applied. Comparing the original, augmented, and cleaned time series illustrates both the effects of the simulated artifacts and the performance of the cleaning method.

## 3 Discussion and Outlook

The Cedalion toolbox is a community effort toward a solution that bridges model-based and data-driven analysis for fNIRS and DOT, with a particular emphasis on findable, accessible, interoperable, and reusable principles;^81^ multimodal integration; and ML readiness. By uniting core optical neuroimaging functionality with the flexibility of the Python ecosystem, Cedalion complements and extends existing MATLAB-based toolboxes such as AtlasViewer, Brain AnalyzIR, Homer2/3, and NeuroDOT. It addresses the need for interoperable, transparent, and scalable workflows capable of supporting both conventional neuroimaging analyses and emerging ML-based paradigms.

### 3.1 Advancing the fNIRS/DOT and Multimodal Ecosystem

Cedalion’s architecture facilitates seamless integration with multimodal and physiological data sources, providing a coherent analysis framework for fNIRS and DOT together with EEG, MEG, OPM-MEG, and other complementary biosignals such as blood pressure, PPG, EDA, ECG, or EMG. This interoperability opens avenues for combined analyses of neural and systemic signals that have historically been treated in isolation. Further, Cedalion’s SNIRF-centered, modality-agnostic architecture is not limited to conventional fNIRS and DOT but can support plugin-style extensions to other optical neuroimaging methods that can be represented within the same data abstractions. As an example, a dedicated speckle contrast optical spectroscopy (SCOS) package builds on Cedalion’s core infrastructure to support recent SCOS and high-density SCOS approaches^82^^,^^83^ that process and infer blood flow index time series, demonstrating how emerging modalities can be integrated into the core framework.

By adhering to community standards (SNIRF and BIDS) and incorporating cloud-executable environments (Jupyter/Colab), the toolbox directly responds to the challenges in reproducibility and transparency highlighted by the FRESH study,^38^ where analytical variability across fNIRS pipelines was shown to critically affect outcomes. Integrated helper functionality that can be run both locally and on the cloud, such as Cedalion’s BIDS conversion tool for the creation of BIDS-compliant datasets from an arbitrary collection of SNIRF files, aims to help lower the barrier to adoption of data standards in the community. Cedalion promotes consistency and reproducibility through structured data models, containerized workflows, and open documentation—key enablers for cross-laboratory comparability and collective scientific advancement.

Key architectural design choices provide a natural interface with ML frameworks such as scikit-learn and PyTorch to facilitate data-driven exploration of latent neural and physiological structures in the data. This way, the toolbox provides an infrastructure to translate methodological insights from the emerging fields of deep learning for fNIRS^20^^,^^21^ and multimodal fusion^23^^,^^69^^,^^84^^–^^86^ into practical, community-accessible research tools.

### 3.2 Limitations and Critical Considerations

Cedalion’s scope and architecture also come with limitations that merit transparent discussion. First, although Python facilitates open and reproducible science, it introduces a learning curve for researchers accustomed to MATLAB-based toolchains. To mitigate this, the toolbox documentation is primarily built on extensive tutorials based on Jupyter notebooks that can be executed with only marginal Python proficiency.

Second, certain features of Cedalion’s advanced functionality, particularly for photon simulation, DOT reconstruction, and ML training, may exceed the resources available to individuals or smaller labs. Although this is a general challenge not specific to the toolbox, we provide mitigation strategies that offer alternative computational pathways. For instance, both GPU- and CPU-based methods are supported for calculating the DOT forward model using Monte Carlo (MCX) or FEM-based (NIRFASTer) photon transport simulations. If local infrastructure cannot accommodate computationally intensive operations (e.g., deep learning model training), Cedalion’s containerized design enables seamless migration to scientific computing clusters or cloud-based environments.

Third, as machine learning becomes increasingly accessible for fNIRS analysis, essential methodological standards from computer science are easily overlooked. Although Cedalion enables the implementation and development of advanced models and architectures, the responsibility for maintaining methodological rigor rests with the researcher. Achieving generalizable and interpretable data-driven solutions requires strict attention to model validation, prevention of overfitting, and preservation of interpretability.

Fourth, the diversity of devices, file formats, and acquisition paradigms remains a major obstacle for standardization. Although Cedalion supports SNIRF and BIDS, continuous alignment with evolving standards and active collaboration with community initiatives will be essential to achieve the envisioned full interoperability across modalities and toolboxes. One major remaining gap is the definition of a standard beyond the SNIRF specification to enable exchanging analysis results, including DOT image-space data, corresponding head models, sensitivity profiles, or voxel/vertex/parcel mapping.

Finally, systematic benchmarking of community pipelines, including those built with Cedalion, against each other on standardized datasets remains an open and necessary effort, echoing the reproducibility initiatives exemplified by the FRESH consortium.

### 3.3 Outlook and Future Directions

With its third major release, Cedalion has reached a stable foundation for multimodal and machine-learning-ready fNIRS and DOT analysis. The focus now shifts from consolidation to expansion. Looking forward, Cedalion aims to support the ongoing transformation of neuroimaging from fragmented analyses toward integrated, continuous, and interpretable workflows that span laboratory and real-world settings.

A key focus of upcoming development lies in pipeline usability. Although the architectural foundations for modular processing chains are implemented, ease of use must approach that of established MATLAB toolboxes such as Homer, where complete pipelines can be executed from compact configuration files. Using Snakemake, Cedalion’s next releases will extend this accessibility to both developers and end users by providing high-level interfaces, preconfigured template (YAML) files, and executable examples for common workflows.

Machine learning integration remains an area of active expansion. Current functionality emphasizes interfacing with established frameworks and includes decomposition methods such as ICA, CCA, and SPoC. Future work will focus on providing the means to integrate lightweight pretrained models for generalizable data-driven applications such as signal quality assessment, artifact rejection, or physiology-aware analysis.

Standardization and data exchange are central to Cedalion’s long-term vision both to enable the training of more complex machine learning models with larger amounts of data and to aid the enhancement of general reproducibility in fNIRS/DOT-based neuroscience. Building on its SNIRF- and BIDS-based data structures, ongoing work addresses the previously mentioned absence of standardized formats for analysis results and DOT reconstructions. These efforts align with community-driven standardization initiatives toward fully interoperable data and tool ecosystems.

Ultimately, Cedalion is conceived as a toolbox from the community for the community, standing on the shoulders of the many who built the foundations of optical neuroimaging. In Greek myth, Cedalion stood upon the shoulders of the blinded giant Orion to guide him toward the rising Sun, where his sight was restored. Likewise, the toolbox exists to bundle and extend—not replace—the collective expertise on which it is built. Consequently, as its strength derives from that shared foundation, a major future focus will lie on continued education and integration of community contribution to aid the advancement of global fNIRS and DOT research.

## Supplementary Material

10.1117/1.NPh.13.S3.S32602.s01

## Data Availability

Version 26.5 of the Cedalion toolbox presented in this article, alongside documentation, example data, and Jupyter notebooks, is publicly available on www.cedalion.tools. The seven tutorials for this paper are available in rendered form in the Supplemental Material and provided in a versioned executable container on https://codeocean.com/capsule/5432660/tree.
