# Supplementary material for: Cedalion tutorial: a Python-based framework for comprehensive analysis of multimodal fNIRS and DOT from the lab to the everyday world
Source: Neurophotonics. 2026 Aug 13;13(Suppl 3):S32602. doi: 10.1117/1.NPh.13.S3.S32602 (PMC13471981; doi:10.1117/1.NPh.13.S3.S32602)
Supplement: Supplementary file 1 [file NPh_013_S32602_SD001.pdf]

Supplementary Material

The paper is accompanied by seven Jupyter tutorial notebooks, included here in rendered form, along with a short Python primer for researchers new to the language.

The notebooks can be explored interactively on Google Colab via the following links:

- S0: Python Primer for fNIRS/DOT Researchers starting with Cedalion
- S1: Head models and Forward Modelling
- S2: Photogrammetric Optode Co-Registration
- S3: Signal Processing
- S4: Model-driven (GLM)
- S5: DOT - Image Reconstruction
- S6: Data-Driven (ML) Analysis
- S7: Data Augmentation

Please use the PDF's document outline to navigate.

The notebooks are hosted on GitHub and will eventually be available on Code Ocean:

- **Code Ocean**: *Link and DOI pending*
- **GitHub**: `https://github.com/ibs-lab/cedalion/tree/dev/examples/tutorial`

To cite any of these notebooks please use the following:

`FIXME DOI issued by Code Ocean`

The notebooks are also part of the Cedalion's official documentation, available at

`http://cedalion.tools/docs`

Appendix S0: Python Primer for fNIRS/DOT Researchers starting with Cedalion

S0.1 Python and the Scientific Computing Ecosystem

Python is a general-purpose, open-source programming language that has become the dominant platform for scientific computing, data science, and machine learning. Unlike MATLAB, which bundles a language with a proprietary computing environment, Python relies on a modular ecosystem of freely available libraries (also called packages) that extend its functionality for specific domains. This modularity is a core reason why Cedalion is built on Python: it allows the toolbox to integrate directly with state-of-the-art machine learning frameworks, neuroimaging tools, and cloud computing platforms without requiring users to leave a single programming environment.

For researchers accustomed to MATLAB-based fNIRS toolboxes such as Homer2/3, AtlasViewer, or Brain AnalyzIR, the transition to Python involves learning new syntax and a different approach to organizing code and data — but the underlying scientific concepts remain identical. **Table S0.1** provides a concise overview of the core Python libraries used in Cedalion and their closest MATLAB equivalents. For users migrating from MATLAB, we also recommend the NumPy documentation's "NumPy for MATLAB Users" guide[1]., which provides a comprehensive side-by-side comparison of array operations between the two languages. For a broader introduction to Python for scientists with prior programming experience, VanderPlas's *A Whirlwind Tour of Python*[2] is a concise, freely available resource; the *SciPy Lecture Notes*[3] offer a practical, tutorial-style introduction to the core scientific Python stack (NumPy, SciPy, matplotlib) well-suited to researchers new to the ecosystem.

Table S0.1: Core Python libraries used in Cedalion.

Python Library	Purpose in Cedalion	MATLAB Equivalent
**NumPy**	N-dimensional arrays and numerical operations	Built-in matrices and array operations
**SciPy**	Signal processing, optimization, statistics	Signal Processing Toolbox, Statistics Toolbox
**Xarray**	Labeled multidimensional arrays with metadata (Cedalion's primary data structure)	No direct equivalent; closest is labeled tables or dataset objects
**pandas**	Tabular data and metadata (e.g., stimulus events)	table data type
**matplotlib**	Plotting and visualization	Built-in plot, imagesc, figure commands
**scikit-learn**	Classical machine learning (classification, cross-validation)	Statistics and Machine Learning Toolbox
**PyTorch**	Deep learning model training and inference	Deep Learning Toolbox
**MNE-Python**	EEG/MEG processing and multimodal integration	FieldTrip, EEGLAB
**pint**	Physical unit tracking and conversion	No equivalent (units are implicit in MATLAB)

The most important library to understand for Cedalion is **Xarray**. While NumPy arrays are indexed by integer position (like MATLAB matrices), Xarray arrays carry named dimensions and coordinate labels — for example, a time series array might have dimensions called *time*, *channel*, and *chromo*, with coordinates specifying timestamps in seconds, channel labels like "S1D1", and chromophore names like "HbO" and "HbR". This labeled structure prevents common indexing errors and makes code more readable. See Figure 3 in the main text for a visual overview of Cedalion's Xarray-based data layout.

A second key difference concerns how library functions are accessed. Python libraries are installed and managed through **package managers**. The two most common are `pip` (Python's built-in package manager) and **conda** (a cross-platform manager popular in scientific computing). In MATLAB, installing a toolbox makes its functions immediately available by name — calling `bandpass()` or `mean()` requires no preamble. In Python, each library must be explicitly *imported* before use, typically at the top of a script or notebook. By convention, libraries are imported under a short alias: `import numpy as np` means that NumPy's array function is subsequently called as `np.array(...)`, not `array(...)`. This explicit namespacing avoids conflicts between functions with the same name from different liblibraries makes it immediately clear — to both the reader and the interpreter — which library each function belongs to. In Cedalion code you will routinely encounter `import numpy as np`, `import xarray as xr`, `import matplotlib.pyplot as plt`, and `import cedalion` at the top of every notebook.

---

[1] NumPy Developers, "NumPy for MATLAB Users," numpy.org/doc/stable/user/numpy-for-matlab-users.html (accessed April 2026).

[2] J. VanderPlas, *A Whirlwind Tour of Python*. O'Reilly Media, 2016. Freely available at: jakevdp.github.io/WhirlwindTourOfPython

[3] SciPy Community, *SciPy Lecture Notes*, lectures.scientific-python.org (accessed April 2026).

S0.2 MATLAB-to-Python Quick Reference

Table S0.2 maps common MATLAB operations to their Python equivalents, focusing on patterns that appear frequently in the Cedalion tutorials. The most important difference to note is that **Python uses 0-based indexing** (the first element is at index 0), while MATLAB uses 1-based indexing (the first element is at index 1).

Table S0.2: Common operations — MATLAB vs. Python.

Operation	MATLAB	Python (NumPy / Cedalion)
**Create a row vector**	`a = [1 2 3]`	`a = np.array([1, 2, 3])`
**Create a matrix**	`A = [1 2; 3 4]`	`A = np.array([[1, 2], [3, 4]])`
**Access first element**	`a(1)`	`a[0]`
**Slice elements 2–4**	`a(2:4)`	`a[1:4]`
**Last element**	`a(end)`	`a[-1]`
**Element-wise multiply**	`A .* B`	`A * B`
**Matrix multiply**	`A * B`	`A @ B`
**Transpose**	`A'`	`A.T`
**Array dimensions**	`size(A)`	`A.shape`
**Select by label (Xarray)**	—	`data.sel(channel="S1D1")`
**Select by time (Xarray)**	—	`data.sel(time=slice(0, 10))`
**Mean along dimension**	`mean(A, 2)`	`A.mean("channel")` (Xarray)
**Load data file**	`load('data.mat')`	`rec = cedalion.io.read_snirf("data.snirf")`
**Plot a time series**	`plot(t, y)`	`plt.plot(t, y)`
**Define a function**	`function y = f(x)` … `end`	`def f(x):` … `return y`
**For loop**	`for i = 1:10` … `end`	`for i in range(10):`
**If statement**	`if x > 0` … `end`	`if x > 0:`
**Comments**	`% comment`	`# comment`
**Print to console**	`disp('hello')`	`print('hello')`
**Logical AND / OR**	& / |	& / | (arrays), and / or (scalars)

S0.3 Development Environment and Tools

Jupyter Notebooks

The Cedalion tutorials are provided as **Jupyter Notebooks** — interactive documents that interleave executable code cells, formatted text, equations, and visualizations in a single file (file extension .ipynb). They are conceptually equivalent to MATLAB Live Scripts (.mlx), with the key difference that Jupyter notebooks can be run in any web browser and are not tied to a specific IDE. Each tutorial notebook can be read as a static document, or executed interactively cell-by-cell, allowing readers to modify parameters and immediately see the effect on the analysis. See the notebooks in Supplements S1-7

Google Colab — Zero-Install Cloud Execution

All Cedalion tutorials can be executed in the cloud via **Google Colab** (colab.research.google.com), a free service that provides a Jupyter notebook environment with pre-installed Python libraries and optional GPU access. This allows users to run the tutorials without installing anything on their local machine — only a web browser and a Google account are required. Colab notebooks can also be shared via a link, making them a convenient way to distribute reproducible analysis pipelines with Cedalion. To open any Cedalion Tutorial in Colab, you can click on the "Open in Colab" button on the top right of any Jupyter notebook on the Cedalion documentation website.

YAML Configuration Files

```yaml
preprocessing:
  filter_type: bandpass
  low_cutoff: 0.01    # Hz
  high_cutoff: 0.5    # Hz
  motion_correction: TDDR
```

Several Cedalion tools use **YAML** files (.yaml or .yml) for configuration. YAML is a plain-text format for structured settings — human-readable, easy to version-control, and independent of any programming language. A YAML file consists of key–value pairs that can be nested into hierarchies, see the textbox example to the right. This is analogous to a MATLAB script that assigns parameter variables, but the YAML file contains no executable code — it is pure configuration, designed to be shared, modified, and reused across analyses. YAML files appear at several points in the Cedalion workflow: the conda environment specification (environment_dev.yml) lists all required dependencies for

installation; and pipeline configuration files supply input parameters — data paths, subject lists, preprocessing settings — to Snakemake workflows without modifying any pipeline code.

Snakemake — Reproducible Pipeline Execution

For complex multi-step analyses, Cedalion integrates with **Snakemake**[4], a workflow management system widely used in bioinformatics and neuroimaging. The central idea is simple: instead of writing a single long script that runs every step in sequence, a user defines the analysis as a set of modular *rules* — each rule declares what input files it needs, what output files it produces, and what processing step to run. Snakemake then automatically determines the correct order of execution by tracing the chain of inputs and outputs across rules. This dependency structure is referred to as a *directed acyclic graph* (DAG) in the computer science literature, but in practice it simply means Snakemake figures out what needs to run and in what order, without the user having to manage this manually. Crucially, if input data or parameters change, Snakemake re-runs only the affected steps — not the entire pipeline — making iterative development efficient. Pipelines can be executed identically on a local laptop, a computing cluster, or a cloud environment, and the full pipeline definition (a plain-text Snakefile together with a YAML configuration) serves as complete, shareable documentation of the analysis. Cedalion's Snakemake integration is under active development; see Section 3.3 of the main manuscript for the current roadmap.

S0.4 Installation and Getting Started

The fastest way to explore Cedalion requires no local installation: visit www.cedalion.tools and click on the book symbol to go to the Cedalion documentation site. Click the "Open in Colab" badge next to any tutorial, and select *Runtime → Run All* — only a web browser and a Google account are needed.

For a local installation, the process is described in detail on the Cedalion documentation site under "Getting started → Installation" and consists of the 5 main steps summarized below.

Step	Where	What
0	Cedalion www.cedalion.tools	Get to all resources (github, documentation, community forum) via the main Cedalion website
1	CONDA docs.anaconda.com/miniconda/install/	Download and install Conda or Miniconda, this provides the conda package manager used to create isolated Python environments. Refer to the Miniconda installation guide for details.
2	github.com/ibs-lab/cedalion	Download or clone the Cedalion git repository with the tool of your choice. For command line git:<br>`cd/path/to/install_directory`<br>`git clone git@github.com:ibs-lab/cedalion.git`
3	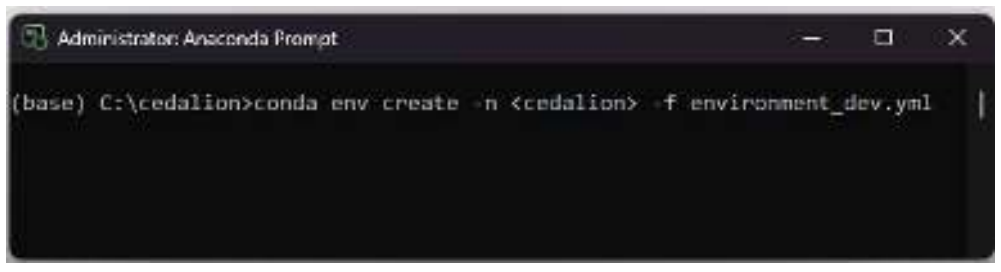	Open a terminal or Anaconda Prompt, move to your Cedalion folder and create the environment (here named "<cedalion>") from the provided YAML specification:<br>`conda env create -n <cedalion> -f environment_dev.yml`
4	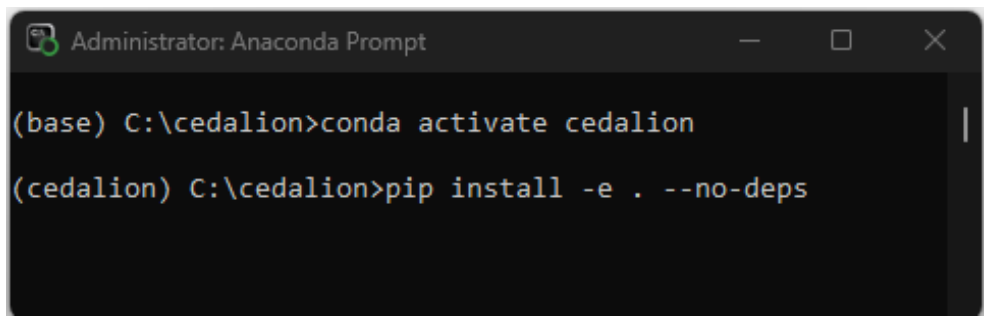	Activate the Cedalion environment and install Cedalion in *editable mode* — so that any updates in your code or pulled from GitHub take effect immediately without reinstallation:<br>`conda activate cedalion`<br>`pip install -e . --no-deps`
5	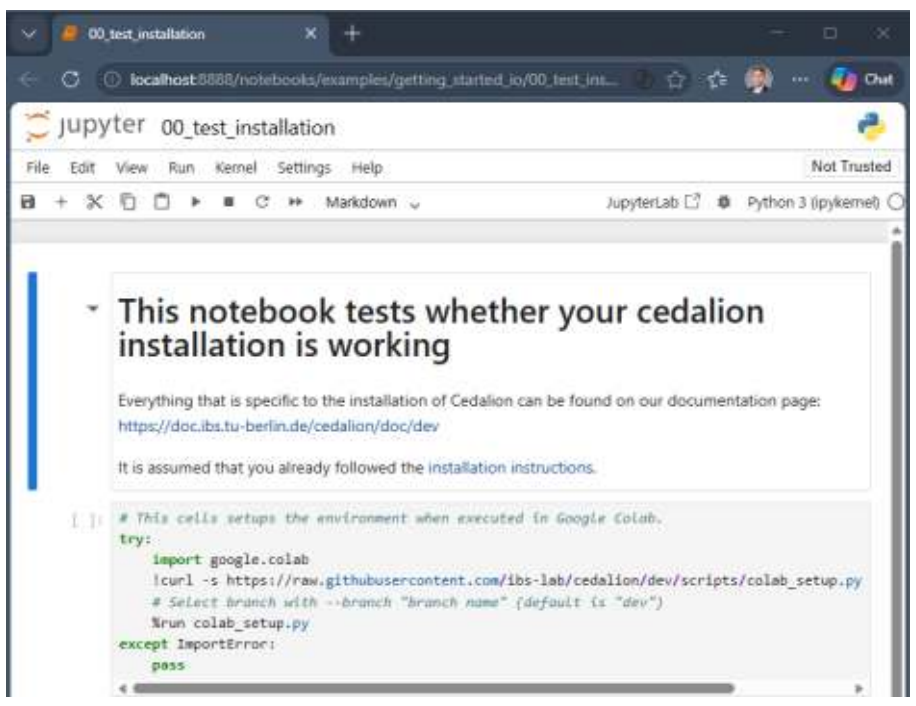	launch Jupyter Notebook in the terminal to access the tutorials:<br>`jupyter notebook`<br>Then follow the instructions in the terminal; copy and paste the given URL into your browser and select a notebook to edit and run in …/cedalion/examples/.<br>The image to the left shows the example "getting_started_io/00_test_installation.ipynb"

[4] F. Mölder et al., "Sustainable data analysis with Snakemake," *F1000Research*, vol. 10, p. 33, 2021, doi: 10.12688/f1000research.29032.2

S1: Head models and Forward Modelling

This notebook introduces how Cedalion handles head models and forward modelling for diffuse optical tomography.

Learning objectives

In this notebook you will learn to:

- Understand the `TwoSurfaceHeadModel` structure (segmentation, scalp mesh, brain mesh, landmarks)
- Navigate the coordinate reference systems used in Cedalion (digitized, ijk, ras/mni)
- Build a forward model: register optodes to the scalp, compute fluence, form the sensitivity matrix

[1]:
```
# This cells setups the environment when executed in Google Colab.
try:
    import google.colab
    !curl -s https://raw.githubusercontent.com/ibs-lab/cedalion/dev/scripts/
 ↪colab_setup.py -o colab_setup.py
    # Select branch with --branch "branch name" (default is "dev")
    %run colab_setup.py
except ImportError:
    pass
```

[2]:
```
from pathlib import Path
from tempfile import TemporaryDirectory

import cedalion
import cedalion.data
import cedalion.dataclasses as cdc
import cedalion.dot
import cedalion.io
import cedalion.nirs
import cedalion.vis.anatomy
import cedalion.vis.blocks as vbx
import matplotlib.pyplot as p
import numpy as np
import pyvista as pv
import xarray as xr
from cedalion import units
from matplotlib.colors import ListedColormap

# set pyvista's jupyter backend to 'server' for interactive 3D plots
pv.set_jupyter_backend("static")
# pv.set_jupyter_backend('server')

xr.set_options(display_expand_data=False);
```

Configuration

The constants in the next cell control the behavior of the notebook.

[3]:

```
# set this to either 'colin27', 'icbm152', 'custom_from_segmentation',␣
↪'custom_from_surfaces'
HEAD_MODEL = "colin27"

# set this to either 'mcx' or 'nirfaster'
FORWARD_MODEL = "mcx"

# set this to False to actually run the forward model
PRECOMPUTED_SENSITIVITY = True
```

Create a temporary directory as a working directory.

[4]:
```
working_directory = TemporaryDirectory()
tmp_dir_path = Path(working_directory.name)
```

Loading and inspecting an example dataset

This notebook loads one of Cedalion's example datasets.

Using a function from the `cedalion.data` package, the following cell will download a snirf file with a recording of a fingertapping experiment.

The content of the snirf file is returned as a `cedalion.dataclasses.Recording` object.

[5]:
```
rec = cedalion.data.get_fingertappingDOT()
```

The Recording container carries time series and related objects through the program in ordered dictionaries much like a segmented tray carrying items in separate compartments.

This notebook mainly needs the probe geometry and channel definitions, detailed below.

Probe Geometry

The snirf file contains the 3D probe geometry. When loaded in Cedalion, the probe geometry is represented in a `xarray.DataArray` with shape (number of labeled points x 3).

It is available as an attribute of the `Recording` container:

[6]:
```
rec.geo3d
```

[6]:
```
<xarray.DataArray (label: 346, digitized: 3)> Size: 8kB
[mm] -77.82 15.68 23.17 -61.91 21.23 56.49 … 14.23 -38.28 81.95 -0.678 -37.03
Coordinates:
    type     (label) object 3kB PointType.SOURCE … PointType.LANDMARK
  * label    (label) <U6 8kB 'S1' 'S2' 'S3' 'S4' … 'FFT10h' 'FT10h' 'FTT10h'
Dimensions without coordinates: digitized
```

This array stores 3D coordinates for 346 points. The first dimension, named `'label'`, indexes the points, while the second dimension holds the three coordinate values for each point. In Cedalion, the name of the second dimension serves as the label for the coordinate's reference system, which in this case is set to `'digitized'`.

Two coordinate arrays are linked to `'label'` dimension. The `'label'` coordinate assigns a string name to each 3D point (for example, `'S1'` for the first source or `'Nz'` for the nasion). Using values of `cedalion.dataclasses.PointType`, the `'type'` coordinate is used to distinguish between sources, detectors and landmarks.

The values stored in the geo3d array have physical units associated with them. In this case, the coordinates are given in millimeters.

This particular schema of representing geometry information in a `xarray.DataArray` is used throughout Cedalion. Arrays following this schema are called `cedalion.typing.LabeledPoints`.

Channel Definitions

This dataset contains one continuous-wave raw amplitudes time series. It is stored in the `Recording` container within the `timeseries` dictionary under the key `'amp'`.

[7]:
```
rec.timeseries.keys()
```

[7]:
```
odict_keys(['amp'])
```

Channels are defined by source-detector pairs. Cedalion uses two ways of representing channel definitions: measurement lists and time series coordinates.

In the SNIRF file, channels are specified in measurement lists, a tabular data structure that lists for each channel source, detector, wavelength and data type.

For each loaded time series, the measurement list is stored as a `pandas.DataFrame` in `rec._measurement_lists` under the same key.

[8]:
```
meas_list = rec._measurement_lists["amp"]
display(meas_list)
```

```
     sourceIndex  detectorIndex  wavelengthIndex wavelengthActual  \
0              1              1                1             None
1              1              2                1             None
2              1              4                1             None
3              1              5                1             None
4              1              6                1             None
..           …              …                …              …
195           14             27                2             None
196           14             28                2             None
197           14             29                2             None
198           14             31                2             None
199           14             32                2             None

    wavelengthEmissionActual  dataType dataUnit dataTypeLabel  dataTypeIndex  \
0                       None         1     None        raw-DC              1
1                       None         1     None        raw-DC              1
2                       None         1     None        raw-DC              1
3                       None         1     None        raw-DC              1
4                       None         1     None        raw-DC              1
..                       …       …      …           …              …
195                     None         1     None        raw-DC              1
196                     None         1     None        raw-DC              1
197                     None         1     None        raw-DC              1
198                     None         1     None        raw-DC              1
199                     None         1     None        raw-DC              1

    sourcePower detectorGain moduleIndex sourceModuleIndex  \
0          None         None        None              None
```

```
1           None         None        None             None
2           None         None        None             None
3           None         None        None             None
4           None         None        None             None
..           …          …         …               …
195         None         None        None             None
196         None         None        None             None
197         None         None        None             None
198         None         None        None             None
199         None         None        None             None

    detectorModuleIndex channel source detector  wavelength chromo
0                  None    S1D1     S1       D1       760.0   None
1                  None    S1D2     S1       D2       760.0   None
2                  None    S1D4     S1       D4       760.0   None
3                  None    S1D5     S1       D5       760.0   None
4                  None    S1D6     S1       D6       760.0   None
..                  …     …    …      …         …    …
195                None  S14D27    S14      D27       850.0   None
196                None  S14D28    S14      D28       850.0   None
197                None  S14D29    S14      D29       850.0   None
198                None  S14D31    S14      D31       850.0   None
199                None  S14D32    S14      D32       850.0   None

[200 rows x 19 columns]
```

The 'amp' time series is represented as a `xarray.DataArray` with dimensions 'channel', 'wavelength' and 'time'. Three coordinate arrays are linked to the 'channel' dimension, specifying for each channel a string label as well as the string label and the corresponding source and detector labels (e.g. the first channel 'S1D1' is between source 'S1' and detector 'D1').

[9]:
```
rec.timeseries["amp"]
```

[9]:
```
<xarray.DataArray (channel: 100, wavelength: 2, time: 8794)> Size: 14MB
[V] 0.0874 0.08735 0.08819 0.08887 0.0879 … 0.09108 0.09037 0.09043 0.08999
Coordinates:
  * time         (time) float64 70kB 0.0 0.2294 0.4588 … 2.017e+03 2.017e+03
    samples      (time) int64 70kB 0 1 2 3 4 5 … 8788 8789 8790 8791 8792 8793
  * channel      (channel) object 800B 'S1D1' 'S1D2' 'S1D4' … 'S14D31' 'S14D32'
    source       (channel) object 800B 'S1' 'S1' 'S1' 'S1' … 'S14' 'S14' 'S14'
    detector     (channel) object 800B 'D1' 'D2' 'D4' 'D5' … 'D29' 'D31' 'D32'
  * wavelength   (wavelength) float64 16B 760.0 850.0
Attributes:
    data_type_group:  unprocessed raw
```

The function `cedalion.vis.anatomy` visualizes the probe geometry. Optode locations are obtained from the geo3d array, while channel definitions are derived from the time series.

Sources, detectors and landmarks are represented as red, blue and grey dots, respectively. The nasion is highlighted in yellow.

[10]:
```
cedalion.vis.anatomy.plot_montage3D(rec["amp"], rec.geo3d)
```

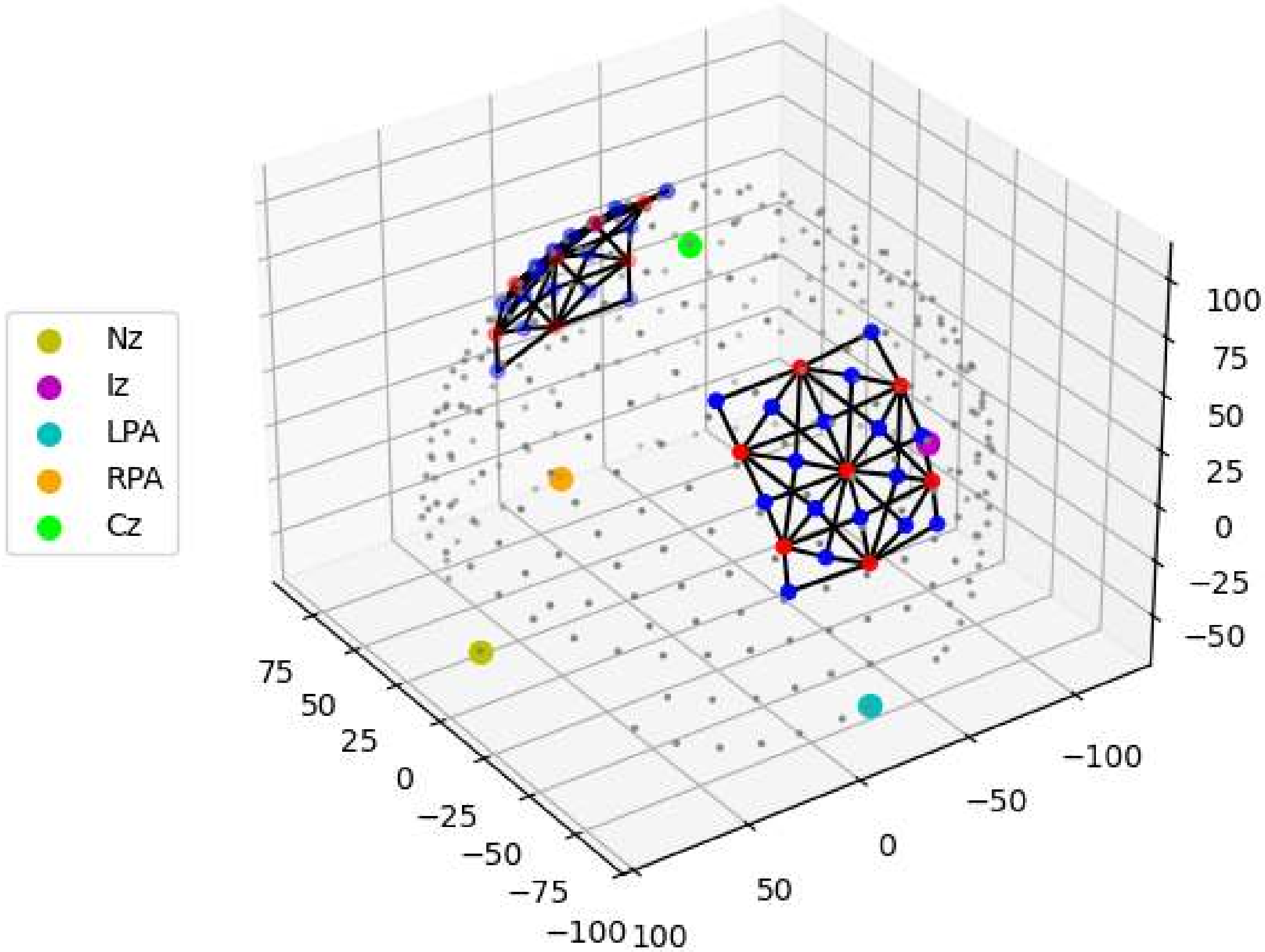

Coordinate systems

When working with probe and head model geometries, it is important to distinguish between coordinates from three different coordinate reference systems (CRS):

- **Digitization space** (`CRS='digitized'`)

  This is the coordinate space used by the digitization or montage planning tool. In this example, optode positions are given in millimeters with the origin located at the head center and the y-axis pointing in the superior direction. The CRS has the user-configurable name `'digitized'`. Other digitization tools may use different names, units or coordinate systems.

- **Voxel space** (`CRS='ijk`)

  Tissue masks from segmented MRI scans are in voxel space, where coordinate axes are aligned with voxel edges. Independent of the MRI scanner resolution or the physical voxel size, voxel space is defined such that each voxel has unit edge length and dimensionless coordinates.

  Axis-aligned grids are computationally efficient, which is why the photon propagation code MCX uses this coordinate system.

- **Scanner space / RAS space** (`CRS='ras'`)

  Scanner space differs from voxel space in that it uses physical units and its coordinate axes point in the **R**ight, **A**nterior, and **S**uperior directions. Therefore, scanner space is also referred to as RAS space. Both voxel and scanner spaces are related through affine transformations which are stored in the head model as `t_ijk2ras` and `t_ras2ijk`.

The scanner space of the standard Colin27 and ICBM-152 heads is widely used as a common coordinate reference system to share results. To highlight this particularly important CRS the head models returned by `get_standard_headmodel` use the label `'mni'` instead of `'ras'` for the scanner space.

To avoid confusion between different coordinate systems, Cedalion is explicit about the CRS associated with each point cloud or surface.

As shown above a dimension name of the `geo3d` array is used to track the CRS. Another way to inspect the CRS is by using the `.points` accessor:

[11]:
```
print(f"The coordinate reference system of the probe geometry is '{rec.geo3d.points.
↪crs}'")
```

```
The coordinate reference system of the probe geometry is 'digitized'
```

The TwoSurfaceHeadModel

The forward model simulates photon propagation through different tissue types. Starting from an anatomical MRI scan, tissue segmentation assigns each voxel to a specific tissue type and corresponding optical properties.

The image reconstruction method implemented in Cedalion does not estimate absorption changes for all voxels. Instead, the inverse problem is simplified by defining two surfaces representing the scalp and the brain and by restricting the reconstruction of absorption changes to voxels in their vicinitiy.

The class `cedalion.dot.TwoSurfaceHeadModel` groups together the segmentation mask, landmark positions and affine transformations as well as the scalp and brain surfaces.

Constructing the TwoSurfaceHeadModel

There are three ways how to construct a TwoSurfaceHeadModel: - using the function `cedalion.dot.get_standard_headmodel` - from tissue segmentation masks. Cedalion derives scalp and brain surfaces. - from tissue segmentation masks and triangulated surface meshes generated by an external tool

[12]:
```
if HEAD_MODEL in ["colin27", "icbm152"]:
    # use a factory method for the standard head models
    head_ijk = cedalion.dot.get_standard_headmodel(HEAD_MODEL)

elif HEAD_MODEL == "custom_from_segmentation":
    # point these to a directory with segmentation masks
    segm_datadir = Path("/path/to/dir/with/segmentation_masks")
    mask_files = {
        "csf": "mask_csf.nii",
        "gm": "mask_gray.nii",
        "scalp": "mask_skin.nii",
        "skull": "mask_bone.nii",
        "wm": "mask_white.nii",
    }
    # the landmarks must be in scanner (RAS) space.
    # For example Slicer3D can be used to pick the landmarks.
    landmarks_file = Path("/path/to/landmarks.mrk.json")

    # Construct a head model from segmentation mask.
    head_ijk = cedalion.dot.TwoSurfaceHeadModel.from_segmentation(
```

```python
        segmentation_dir=segm_datadir,
        mask_files=mask_files,
        landmarks_ras_file=landmarks_file,
        # adjust these to control mesh parameters
        brain_face_count=None,
        scalp_face_count=None,
        smoothing=0.0,
    )
elif HEAD_MODEL == "custom_from_surfaces":
    # point these to a directory with segmentation masks
    segm_datadir = Path("/path/to/dir/with/segmentation_masks")
    mask_files = {
        "csf": "mask_csf.nii",
        "gm": "mask_gray.nii",
        "scalp": "mask_skin.nii",
        "skull": "mask_bone.nii",
        "wm": "mask_white.nii",
    }
    # the landmarks must be in scanner (RAS) space.
    # For example Slicer3D can be used to pick the landmarks.
    landmarks_file = Path("/path/to/landmarks.mrk.json")

    # if available provide a mapping between vertices and labels
    coordinates_file = None

    # Better brain and scalp surfaces are achievable from
    # specialized segmentation tools.
    head_ijk = cedalion.dot.TwoSurfaceHeadModel.from_surfaces(
        segmentation_dir=segm_datadir,
        mask_files=mask_files,
        landmarks_ras_file=landmarks_file,
        coordinates_file=coordinates_file,
        brain_surface_file=segm_datadir / "mask_brain.obj",
        scalp_surface_file=segm_datadir / "mask_scalp.obj",
    )
else:
    raise ValueError("unknown head model")
```

Inspecting the Head Model

The created head model is in voxel space (CRS='ijk'). The string representation shows its different components: the segmentation masks for different tissue types, the brain and scalp surfaces as well as the landmarks.

[13]:
```python
head_ijk
```

[13]:
```
TwoSurfaceHeadModel(
  crs: ijk
  tissue_types: csf, gm, scalp, skull, wm
  brain faces: 49992 vertices: 25000 units: dimensionless
  scalp faces: 20096 vertices: 10050 units: dimensionless
  landmarks: 73
```

)

The segmentation masks are 3D image cubes (dimensions i,j,k) that are stacked along a fourth dimension 'segmentation_type'. Each tissue mask has a unique integer assigned which are used to mark voxels that belong to that tissue type. The string label of each tissue type (e.g. 'gm', 'csf') map to tabulated optical properties in `cedalion.dot.tissue_properties.TISSUE_LABELS`.

[14]:
```
head_ijk.segmentation_masks
```

[14]:
```
<xarray.DataArray (segmentation_type: 5, i: 181, j: 217, k: 181)> Size: 36MB
0 0 0 0 0 0 0 0 0 0 0 0 0 0 0 0 0 0 0 … 0 0 0 0 0 0 0 0 0 0 0 0 0 0 0 0 0 0 0
Coordinates:
  * segmentation_type  (segmentation_type) <U5 100B 'csf' 'gm' … 'skull' 'wm'
Dimensions without coordinates: i, j, k
```

Visualize segmentation masks

[15]:
```
collapsed_mask = head_ijk.segmentation_masks.sum("segmentation_type")

cmap = ListedColormap(["black", "cyan", "lightgray", "pink", "gray", "white"])
p.pcolormesh(collapsed_mask[:,:, collapsed_mask.sizes["k"]//2], cmap=cmap, vmin=-0.5,␣
↪vmax=5.5)
cb = p.colorbar()
cb.set_ticks([0,1,2,3,4,5])
cb.set_ticklabels(["air", "CSF", "gray matter", "skin", "skull", "white matter"])
p.xlabel("i")
p.ylabel("j");
```

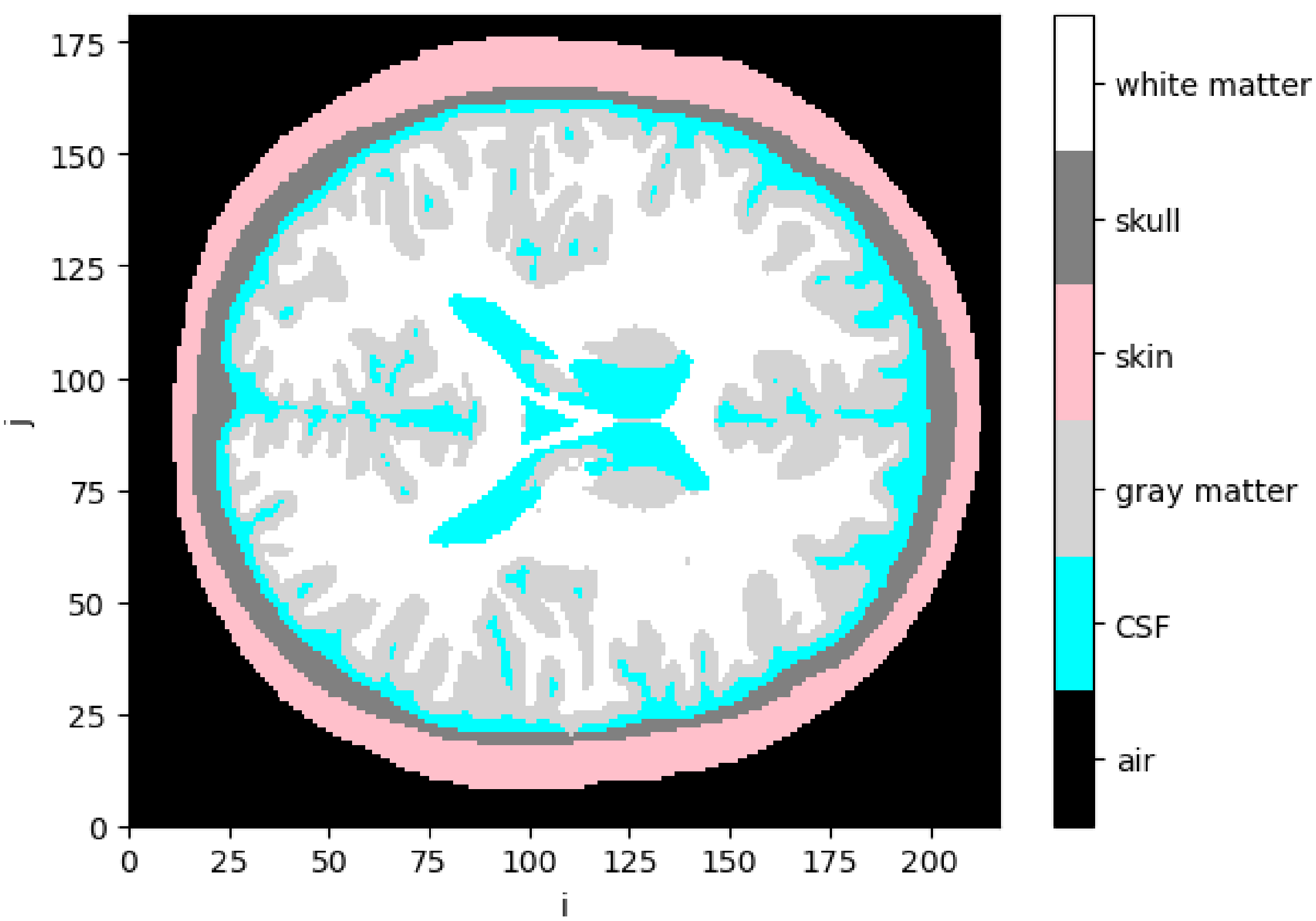

Landmarks are stored as `LabeledPoints`.

[16]:
```
head_ijk.landmarks
```

[16]:
```
<xarray.DataArray (label: 73, ijk: 3)> Size: 2kB
[] 90.0 7.435 48.92 3.924 106.0 24.01 … 127.4 25.63 109.0 139.8 26.66 93.47
Coordinates:
  * label    (label) <U3 876B 'Iz' 'LPA' 'RPA' 'Nz' … 'PO1' 'PO2' 'PO4' 'PO6'
    type     (label) object 584B PointType.LANDMARK … PointType.LANDMARK
Dimensions without coordinates: ijk
```

The brain and scalp surfaces are represented by triangulated meshes, which are represented by instances of `cedalion.dataclasses.TrimeshSurface`.

[17]:
```
head_ijk.brain
```

[17]:
```
TrimeshSurface(faces: 49992 vertices: 25000 crs: ijk units: dimensionless vertex_coords:
['fsaverage_vertex', 'parcel', 'mni152_r', 'mni152_a', 'mni152_s'])
```

[18]:
```
head_ijk.scalp
```

[18]:
```
TrimeshSurface(faces: 20096 vertices: 10050 crs: ijk units: dimensionless vertex_coords:
[])
```

The attribute `.crs` indicates that the head model and all its components is in voxel space.

[19]:
```
head_ijk.crs
```

[19]:
```
'ijk'
```

Transformations

The affine transformations between different coordinate reference systems are represented as 4x4 matrices which Cedalion also stores in `DataArrays`.

The dimension names are chosen such that applying the transformation through matrix multiplication transforms `LabeledPoints` from one CRS to the other.

The transformation matrix to translate from voxel to scanner space is available as:

[20]:
```
head_ijk.t_ijk2ras
```

[20]:
```
<xarray.DataArray (mni: 4, ijk: 4)> Size: 128B
[mm] 1.0 0.0 0.0 -90.0 0.0 1.0 0.0 -126.0 0.0 0.0 1.0 -72.0 0.0 0.0 0.0 1.0
Dimensions without coordinates: mni, ijk
```

And the inverse transformation matrix to translate from scanner to voxel space is available as:

[21]:
```
head_ijk.t_ras2ijk
```

[21]:
```
<xarray.DataArray (ijk: 4, mni: 4)> Size: 128B
[1/mm] 1.0 -1.616e-14 -8.192e-15 90.0 … 3.253e-16 -1.74e-16 -8.828e-17 1.0
Dimensions without coordinates: ijk, mni
```

The head model provides the function `.apply_transform` to change the coordinate systems for all components:

[22]:
```
head_ras = head_ijk.apply_transform(head_ijk.t_ijk2ras)
display(head_ras)
```

```
TwoSurfaceHeadModel(
  crs: mni
  tissue_types: csf, gm, scalp, skull, wm
  brain faces: 49992 vertices: 25000 units: millimeter
  scalp faces: 20096 vertices: 10050 units: millimeter
  landmarks: 73
)
```

Scaling head models or registering optode positions

- cedalion has functionality to scale the head model to given head sizes or probe geometries
- alternatively, the probe geometry can be scaled to match the head size, changing channel distances in doing so
- the scaling affects how large a head model is in scanner space. the result of the scaling is represented in the trafos that connect voxel and scanner space

[23]:
```
def compare_trafos(t_orig, t_scaled):
    """Compare the length of a unit vector after applying the transform."""
    t_orig = t_orig.pint.dequantify().values
    t_scaled = t_scaled.pint.dequantify().values

    for i, dim in enumerate("xyz"):
        unit_vector = np.zeros(4)
        unit_vector[i] = 1.0
        n1 = np.linalg.norm(t_orig @ unit_vector)
        n2 = np.linalg.norm(t_scaled @ unit_vector)

        print(
            f"After scaling, a unit vector along the {dim}-axis is {n2 / n1 * 100.0:.
↪1f}% "
            "of the original length."
        )
```

Scale head model using geodesic distances

Transform the head model to the subject's head size as defined by three measurements: - the head circumference - the distance from Nz through Cz to Iz - the distance from LPA through Cz to RPA

These distances define a ellipsoidal scalp surface to which the head model is fit.

The resulting head will be in CRS='ellipsoid'.

[24]:
```
head_scaled = head_ijk.scale_to_headsize(
    circumference=51*units.cm,
    nz_cz_iz=33*units.cm,
    lpa_cz_rpa=33*units.cm
)
display(head_scaled)
```

```
TwoSurfaceHeadModel(
  crs: ellipsoid
  tissue_types: csf, gm, scalp, skull, wm
  brain faces: 49992 vertices: 25000 units: millimeter
  scalp faces: 20096 vertices: 10050 units: millimeter
  landmarks: 73
)
```

Visualize original (green) and scaled (red) scalp surfaces and landmarks.

[25]:
```
plt = pv.Plotter()
vbx.plot_surface(plt, head_ras.scalp, color="#4fce64", opacity=.1)
vbx.plot_labeled_points(plt, head_ras.landmarks, color="g")
vbx.plot_surface(plt, head_scaled.scalp, color="#ce5c4f")
vbx.plot_labeled_points(plt, head_scaled.landmarks, color="r")
plt.show()
```

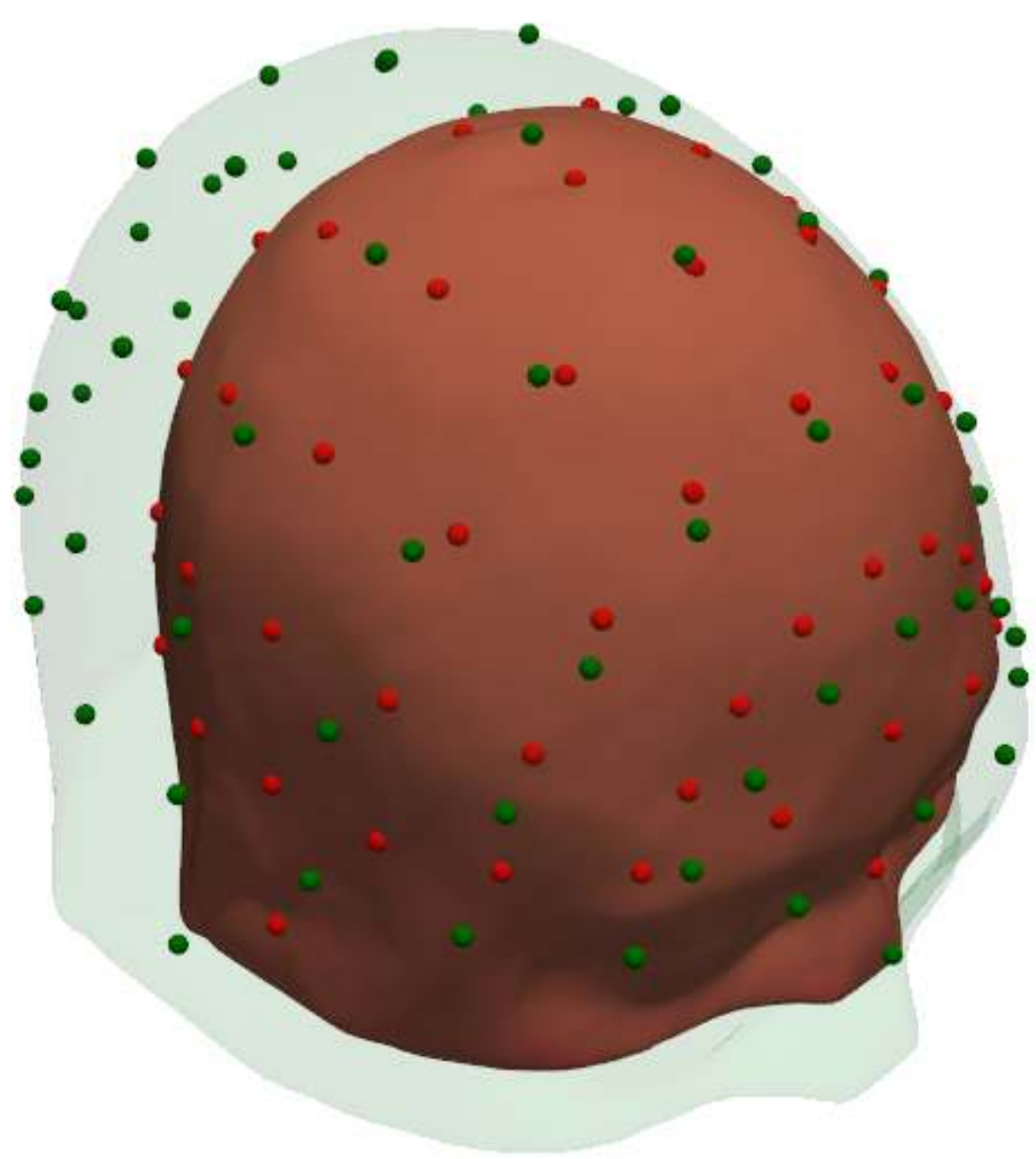

[26]:
```
compare_trafos(head_ijk.t_ijk2ras, head_scaled.t_ijk2ras)
```

```
After scaling, a unit vector along the x-axis is 89.8% of the original length.
After scaling, a unit vector along the y-axis is 76.4% of the original length.
After scaling, a unit vector along the z-axis is 87.3% of the original length.
```

Scale head model using digitized landmark positions

Transform the head model to the subject's head size by fitting landmarks of the head model to digitized landmarks.

The resulting head will be in the same CRS as the digitized landmarks. This also affects axis orientations.

[27]:
```
# Construct LabeledPoints with landmark positions.
# Use the digitized landmarks from 41_photogrammetric_optode_coregistration.ipynb
geo3d = cdc.build_labeled_points(
    [
        [14.00420712, -7.84856869, 449.77840004],
        [99.09920059, 29.72154755, 620.73876117],
        [161.63815139, -48.49738938, 494.91210993],
        [82.8771277, 79.79500128, 498.3338802],
        [15.17214095, -60.56186128, 563.29621021],
    ],
    crs="digitized",
    labels=["Nz", "Iz", "Cz", "LPA", "RPA"],
    types = [cdc.PointType.LANDMARK] * 5,
    units="mm"
)

# shift the center of the landmarks to plot original and
# scaled versions next to each other
geo3d = geo3d - geo3d.mean("label") + (0,200,0)*cedalion.units.mm

head_scaled = head_ijk.scale_to_landmarks(geo3d)
```

Visualize original (green) and scaled (red) scalp surfaces and landmarks.

The digitized landmarks are plotted in yellow.

[28]:
```
plt = pv.Plotter()
vbx.plot_surface(plt, head_ras.scalp, color="#4fce64", opacity=.1)
vbx.plot_labeled_points(plt, head_ras.landmarks, color="g")
vbx.plot_surface(plt, head_scaled.scalp, color="#ce5c4f", opacity=.8)
vbx.plot_labeled_points(plt, head_scaled.landmarks, color="r")
vbx.plot_labeled_points(plt, geo3d, color="y", )
vbx.camera_at_cog(plt, head_scaled.scalp, (600,0,0), fit_scene=True)
plt.show()
```

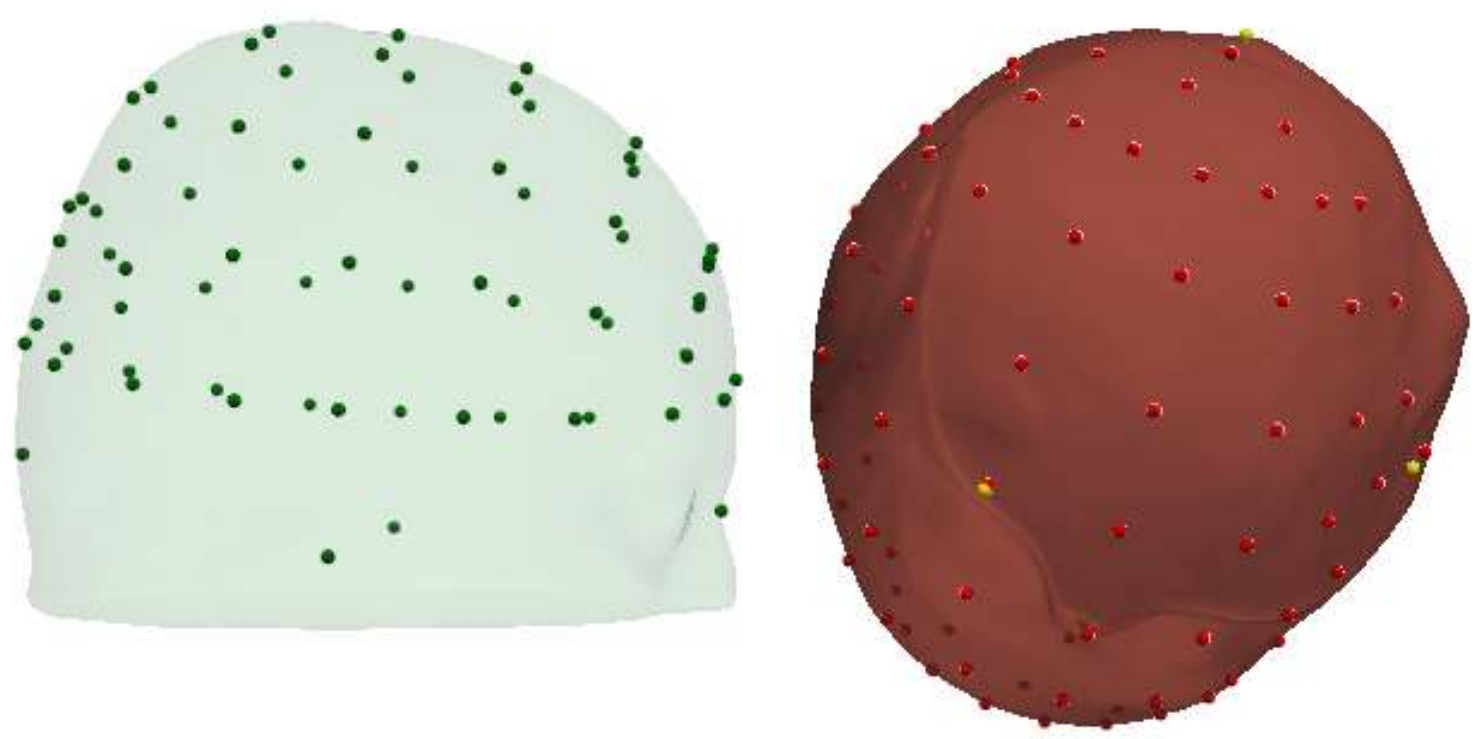

[29]:
```
compare_trafos(head_ijk.t_ijk2ras, head_scaled.t_ijk2ras)
```

```
After scaling, a unit vector along the x-axis is 98.2% of the original length.
After scaling, a unit vector along the y-axis is 95.9% of the original length.
After scaling, a unit vector along the z-axis is 93.0% of the original length.
```

Scale digitized optode positions to head model

Without changing the head model size, transform the optode positions to fit the head model's landmarks.

The function first aligns the landmarks via an unconstrained affine transformation, then uses spring relaxation to conform the montage to the scalp surface with minimal distortion of channel distances. To derive the unconstrained transformation the digitized coordinates and head model landmarks must contain at least the positions of "Nz", "Iz", "Cz", "LPA", "RPA".

If fewer landmarks are available `align_and_relax_to_scalp` can be called with `initial_align_mode='trans_rot_isoscale`, which restricts the affine to translations, rotations and isotropic scaling, which have fewer degrees of freedom.

[30]:
```
geo3d_snapped_ras, alignment_details = head_ras.align_and_relax_to_scalp(rec.geo3d,␣
    ↪rec["amp"])
```

The transformed digitized optode and landmark positions are plotted in red (sources), blue (detectors) and green (landmarks).

The fiducial landmarks of the head model are plotted in yellow.

[31]:
```
plt = pv.Plotter()
vbx.plot_surface(plt, head_ras.scalp, color="#4fce64", opacity=.1)
vbx.plot_labeled_points(plt, geo3d_snapped_ras)
vbx.plot_labeled_points(
    plt, head_ras.landmarks.sel(label=["Nz", "Iz", "Cz", "LPA", "RPA"]), color="y"
)
plt.show()
```

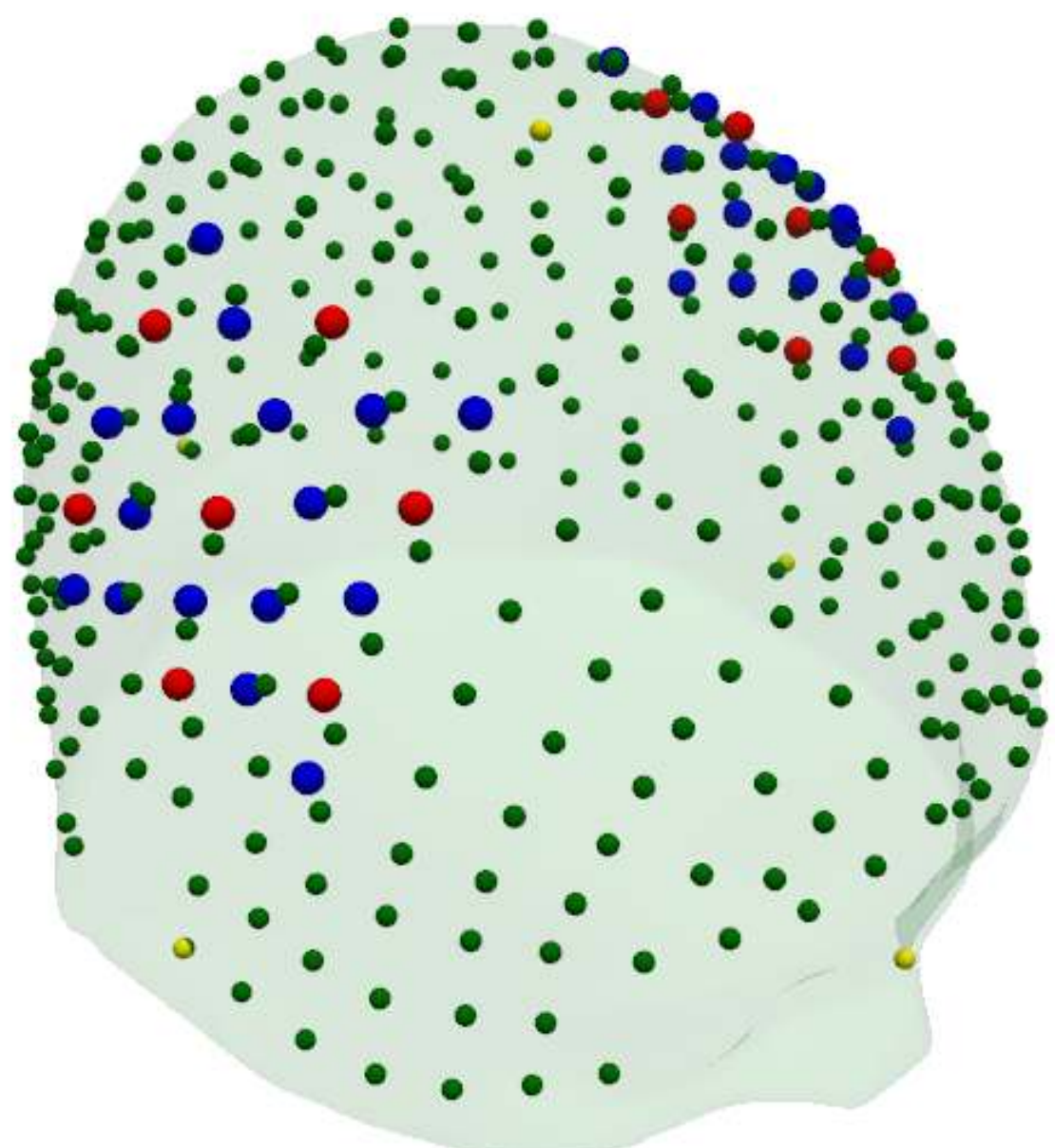

[32]:
```
bins = np.arange(0, 40.5, 1)
p.hist(cedalion.nirs.channel_distances(rec["amp"], rec.geo3d), bins, label="before␣
↪scaling", fc="g", alpha=.5)
p.hist(cedalion.nirs.channel_distances(rec["amp"], geo3d_snapped_ras), bins,␣
↪label="after scaling", fc="r", alpha=.5);
p.legend()
p.xlabel("channel distance / mm")
p.ylabel("# channel")
p.xlim(0,40);
```

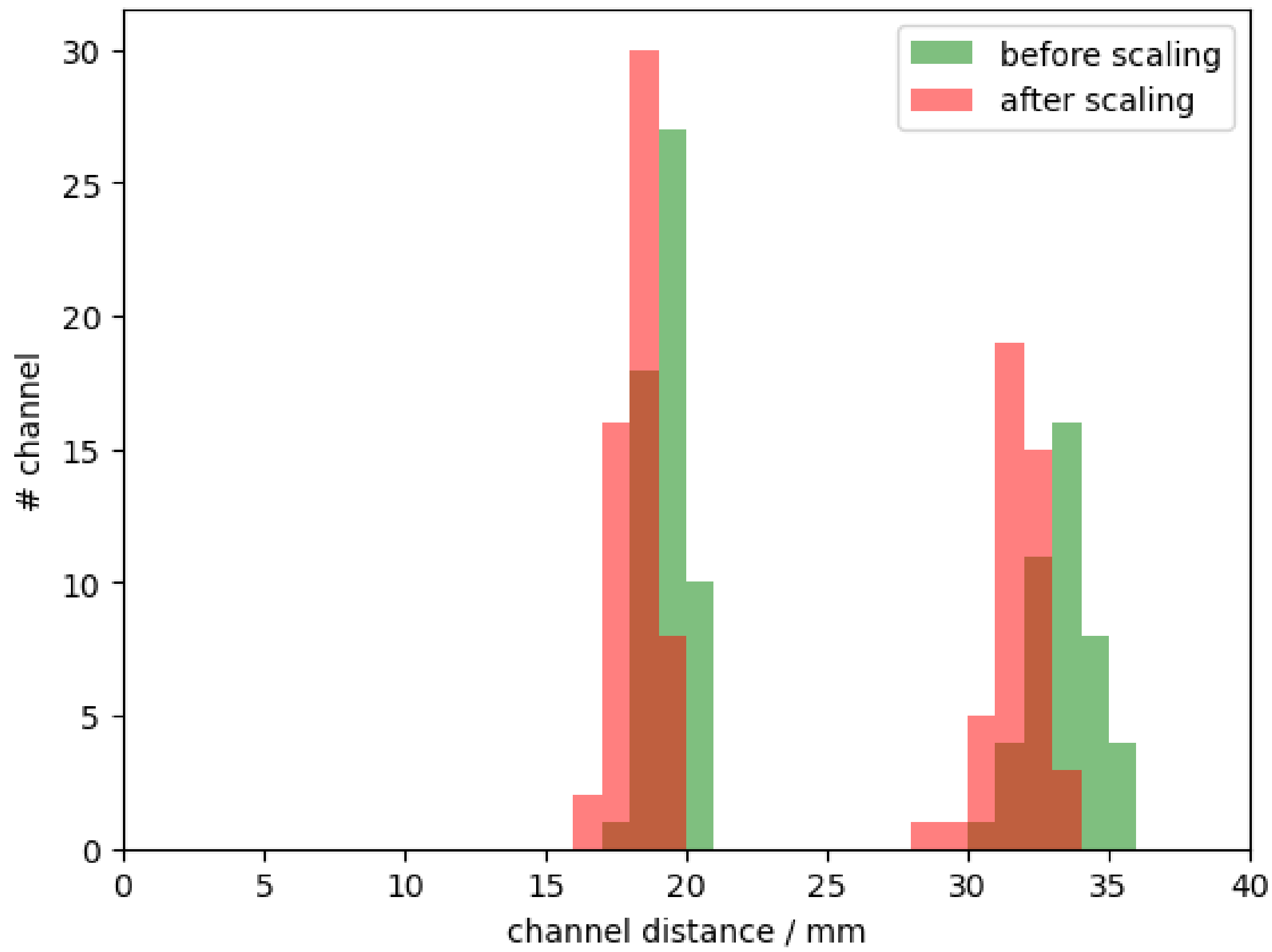

Simulate light propagation in tissue

`cedalion.dot.ForwardModel` is a wrapper around pmcx and NIRFASTER. It transforms the data in the head model to the input format expected by these photon propagators and offers functionality to calculate the sensitivity matrix.

The forward model operates in voxel space, so we transform the aligned optodes into this CRS.

[33]:
```
geo3d_snapped_ijk = geo3d_snapped_ras.points.apply_transform(head_ijk.t_ras2ijk)
```

[34]:
```
fwm = cedalion.dot.ForwardModel(head_ijk, geo3d_snapped_ijk, meas_list)
```

Run the simulation

The `compute_fluence_mcx` and `compute_fluence_nirfaster` methods simulate a light source at each optode position and calculate the fluence in each voxel. By setting `FORWARD_MODEL`, you can choose between the pmcx or NIRFASTer package to perform this simulation.

[35]:
```
if PRECOMPUTED_SENSITIVITY:
    Adot = cedalion.data.get_precomputed_sensitivity("fingertappingDOT", HEAD_MODEL)
else:
    fluence_fname = working_directory / "fluence.h5"
    sensitivity_fname = working_directory / "sensitivity.h5"

    # calculate fluence into fluence file
    if FORWARD_MODEL == "MCX":
```

```
        fwm.compute_fluence_mcx(fluence_fname)
    elif FORWARD_MODEL == "NIRFASTER":
        fwm.compute_fluence_nirfaster(fluence_fname)

    # calculate sensitivity from fluence into sensitivity file
    fwm.compute_sensitivity(fluence_fname, sensitivity_fname)

    # load sensitivity
    Adot = cedalion.io.load_Adot(sensitivity_fname)
```

The result is a sensitivity matrix for each wavelength that describes how changes in absorption at a given brain vertex affect the optical density in a given channel.

[36]:
```
Adot
```

[36]:
```
<xarray.DataArray (channel: 100, vertex: 35050, wavelength: 2)> Size: 28MB
3.656e-17 3.656e-17 1.758e-18 1.758e-18 … 8.413e-17 2.986e-15 2.986e-15
Coordinates:
    parcel      (vertex) object 280kB 'VisCent_ExStr_8_LH' … 'scalp'
    is_brain    (vertex) bool 35kB True True True True … False False False
  * channel     (channel) object 800B 'S1D1' 'S1D2' 'S1D4' … 'S14D31' 'S14D32'
    source      (channel) object 800B 'S1' 'S1' 'S1' 'S1' … 'S14' 'S14' 'S14'
    detector    (channel) object 800B 'D1' 'D2' 'D4' 'D5' … 'D29' 'D31' 'D32'
  * wavelength  (wavelength) float64 16B 760.0 850.0
Dimensions without coordinates: vertex
Attributes:
    units:    mm
```

Visualize the sensitivity

[37]:
```
# select only a subset of labeled points to plot
# Here we select sources and detectors via their labelsthat start with S or D
geo3d_plot_ijk = geo3d_snapped_ijk.sel(
    label=geo3d_snapped_ijk.label.str.contains("S|D")
)

plotter = cedalion.vis.anatomy.sensitivity_matrix.Main(
    sensitivity=Adot,
    brain_surface=head_ijk.brain,
    head_surface=head_ijk.scalp,
    labeled_points= geo3d_plot_ijk,
)
plotter.plot(high_th=0, low_th=-3)
plotter.plt.show()
```

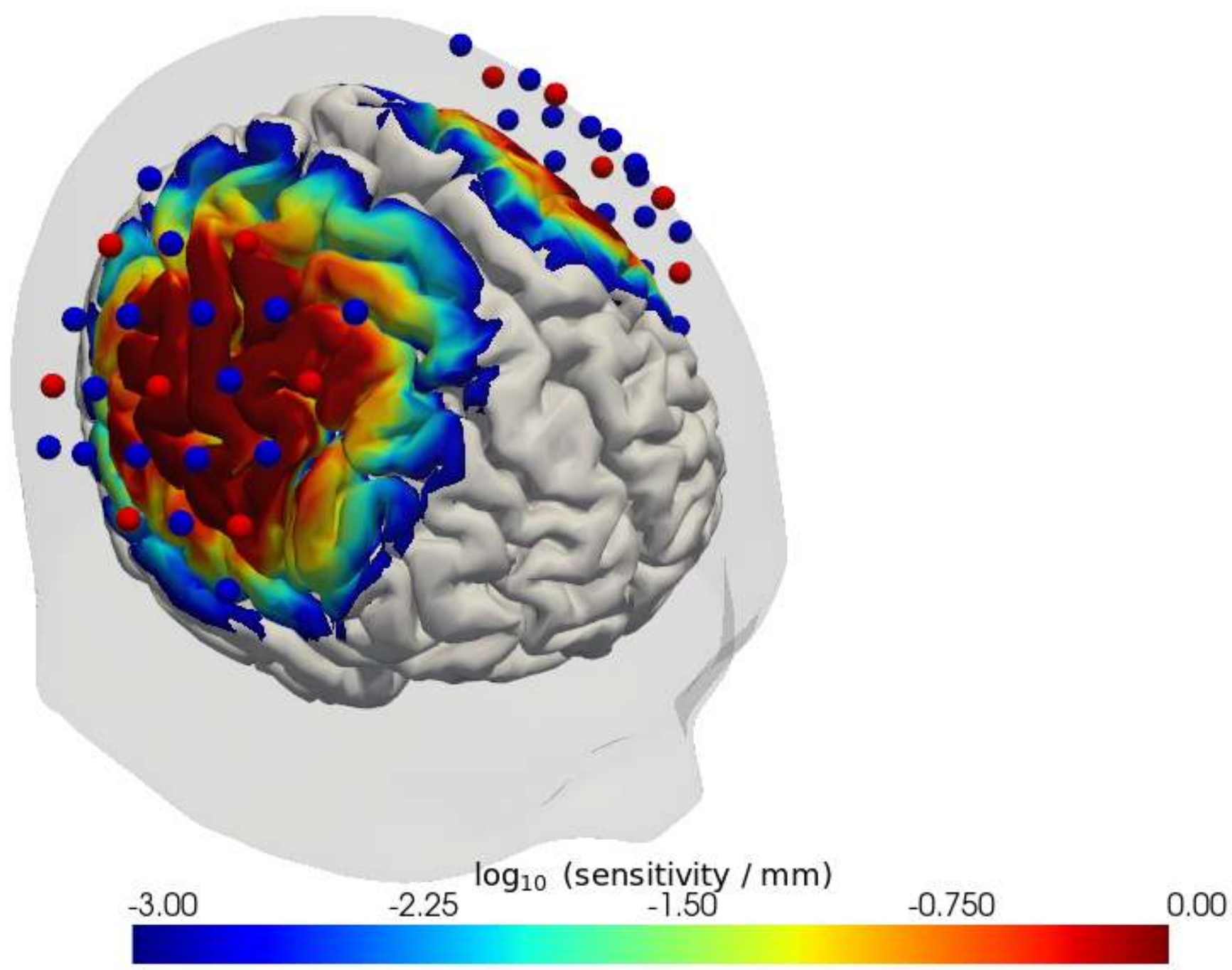

References

[38]:
```
cedalion.bib.dump_to_notebook()
```

```
<IPython.core.display.HTML object>
```

Next: 2 — Photogrammetry — registering optode positions from a photogrammetric scalp scan to the head model.

S2: Photogrammetric Optode Co-Registration

Photogrammetry offers a possibility to get subject-specific optode coordinates. This notebook illustrates the individual steps to obtain these coordinates from a textured triangle mesh and a predefined montage.

Learning objectives

In this notebook you will learn to:

- Understand what photogrammetry is and why it is used for fNIRS optode localisation
- Use Cedalion's photogrammetric registration to align a scalp scan to the head model
- Visualise the co-registration result and assess alignment quality

[1]:
```
# This cells setups the environment when executed in Google Colab.
try:
    import google.colab
    !curl -s https://raw.githubusercontent.com/ibs-lab/cedalion/dev/scripts/
↪colab_setup.py -o colab_setup.py
    # Select branch with --branch "branch name" (default is "dev")
    %run colab_setup.py
except ImportError:
    pass
```

[2]:
```
import matplotlib.image as mpimg
import matplotlib.pyplot as plt

import pyvista as pv
import xarray as xr

import cedalion
import cedalion.dataclasses as cdc
import cedalion.data
import cedalion.geometry.registration
import cedalion.io
import cedalion.vis
import cedalion.vis.blocks as vbx
from cedalion.vis.anatomy import OptodeSelector
from cedalion.geometry.photogrammetry.processors import (
    ColoredStickerProcessor,
    geo3d_from_scan,
)
from cedalion.geometry.registration import find_spread_points
from tempfile import TemporaryDirectory
from pathlib import Path


xr.set_options(display_expand_data=False)
```

[2]:
```
<xarray.core.options.set_options at 0x7387bf235310>
```

0. Choose between interactive and static mode

This example notebook provides two modes, controlled by the constant `INTERACTIVE`: - a static mode intended for rendering the documentation - an interactive mode, in which the 3D visualizations react to user input. The camera position can be changed. More importantly, the optode and landmark picking needs these interactive plots.

[3]:
```
INTERACTIVE = False

if INTERACTIVE:
    # option 1: render in the browser
    # pv.set_jupyter_backend("client")
    # option 2: offload rendering to a server process using trame
    pv.set_jupyter_backend("server")
else:
    pv.set_jupyter_backend("static")  # static rendering (for documentation page)
```

1. Loading the triangulated surface mesh

Use `cedalion.io.read_einstar_obj` to read the textured triangle mesh produced by the Einstar scanner. By default we use an example dataset. By setting the `fname_` variables the notebook can operate on another scan.

[4]:
```
# insert here your own files if you do not want to use the example
fname_scan = ""  # path to .obj scan file
fname_snirf = "" # path to .snirf file for montage information
fname_montage_img = "" # path to an image file of the montage

if not fname_scan:
    fname_scan, fname_montage_img = (
        cedalion.data.get_fingertappingDOT_photogrammetry_scan()
    )

surface_mesh = cedalion.io.read_einstar_obj(fname_scan)
display(surface_mesh)
```

```
TrimeshSurface(faces: 1170428 vertices: 628185 crs: digitized units: millimeter␣
↪vertex_coords: [])
```

2. Identifying sticker vertices

Processors are meant to analyze the textured mesh and extract positions. The `ColoredStickerProcessor` searches for colored vertices that form circular areas. We operate in HSV color space and the colors must be specified by their ranges in hue and value. These can be found by usig a color pipette tool on the texture file.

Multiple classes with different colors can be specified. In the following only yellow stickers for class "O(ptode)" are searched. But it could be extended to search also for differently colored sticker. (e.g. "L(andmark)").

For each sticker the center and the normal is derived. Labels are generated from the class name and a counter, e.g. "O-01, O-02, …"

[5]:
```
processor = ColoredStickerProcessor(
    colors={
        "O" : ((0.11, 0.21, 0.7, 1)), # (hue_min, hue_max, value_min, value_max)
        #"L" : ((0.25, 0.37, 0.35, 0.6))
    }
)

# Select the hue and value ranges based on this preview of the distribution of
# vertices in color space.
preview_details = processor.inspect_colors(surface_mesh)
preview_details.plot_vertex_colors()
```

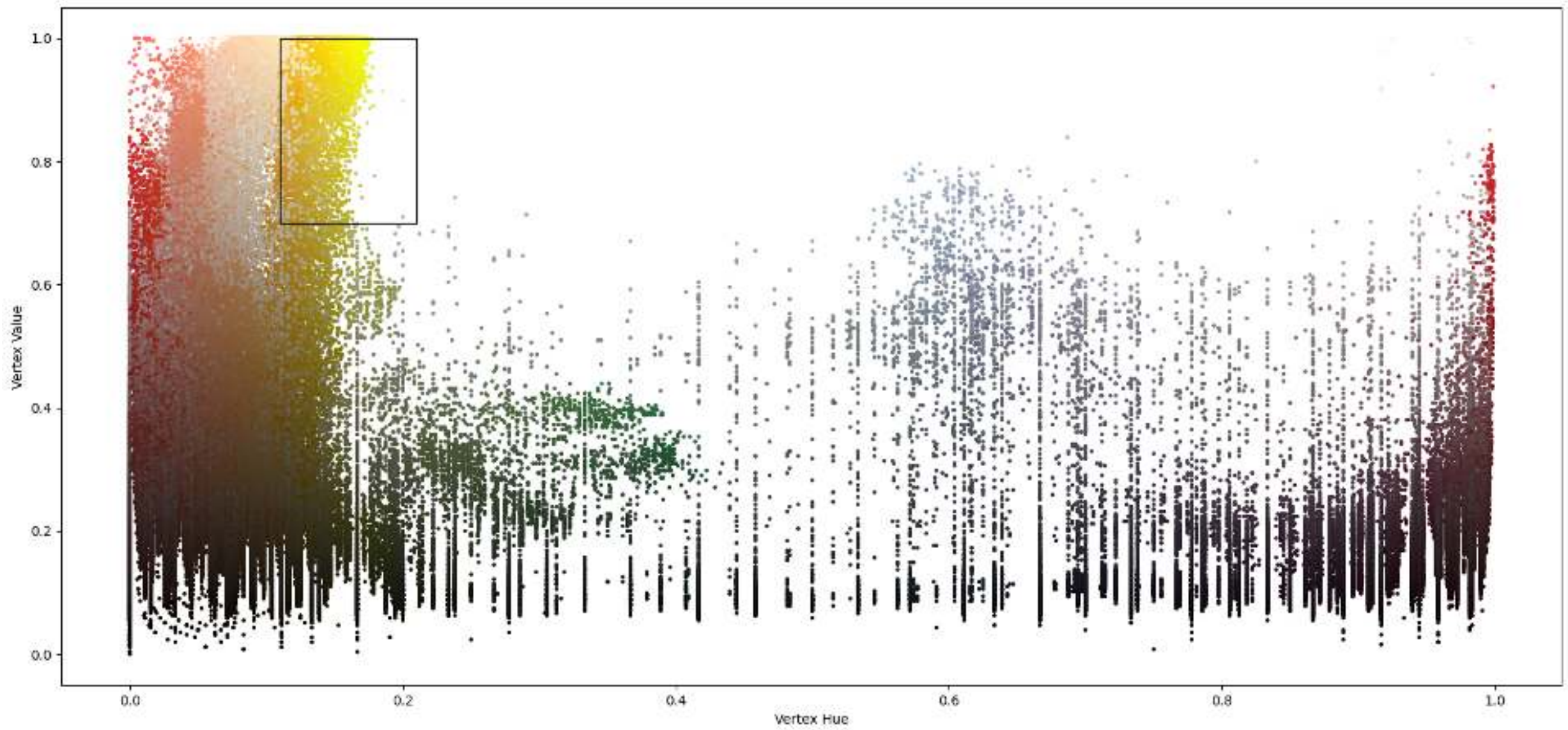

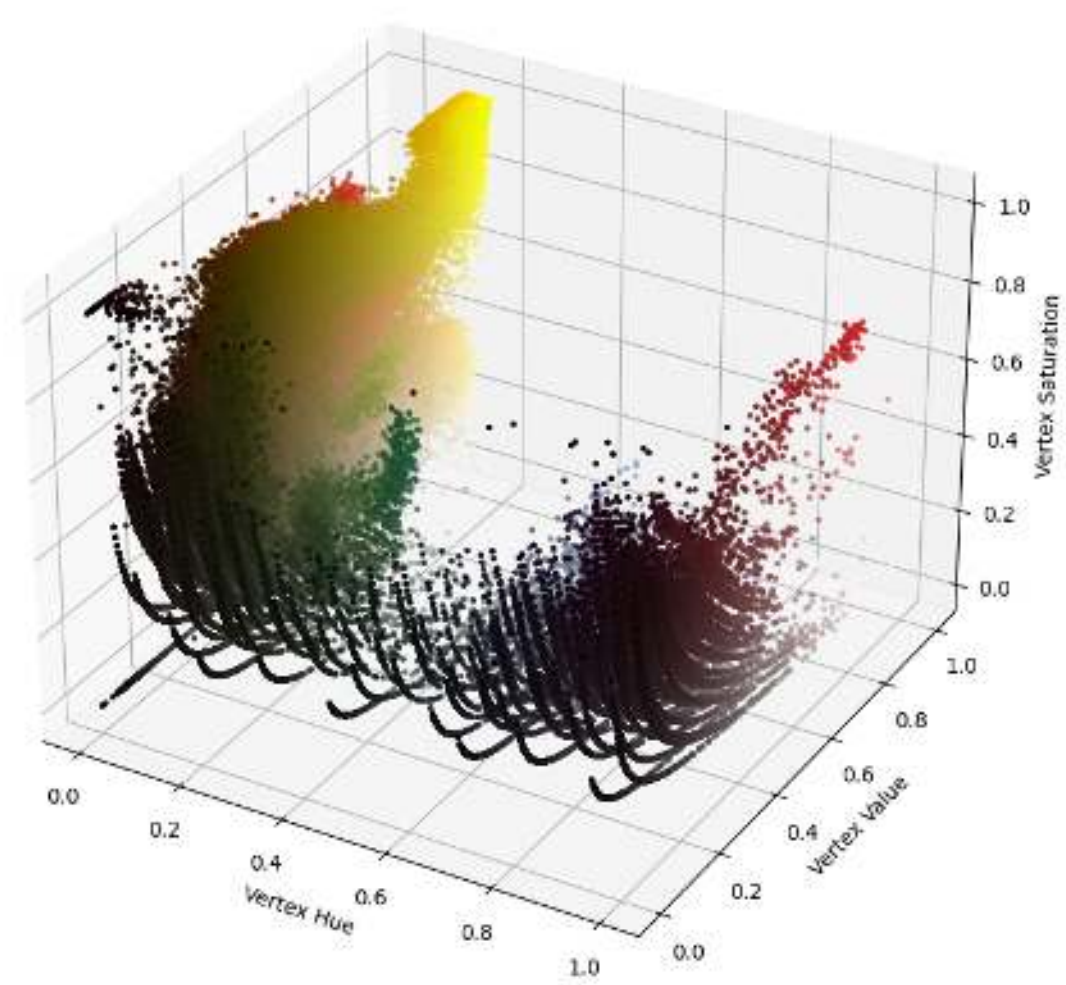

[6]:
```
# process the mesh
sticker_centers, normals, details = processor.process(surface_mesh, details=True)
display(sticker_centers)
```

```
[[172.506348 168.70253  570.348572]
 [150.027954  86.302536 570.348572]
 [172.497742 169.502548 570.348572]
 …
 [146.47406  217.239182 493.978607]
 [145.855286 217.494583 493.578003]
 [145.55806  217.509079 493.342957]]
[[153.826904 253.747421 570.348572]
 [152.226929 254.987106 570.348572]
 [151.426941 255.54277  570.348572]
 …
 [147.931213 179.410263 446.350281]
 [146.85408  180.071243 446.188019]
 [148.198898 179.035706 446.32309 ]]
O (0.11, 0.21, 0.7, 1)
```

```
[0.08044177 0.13082995 0.33646228 0.11412244 0.25736438 0.11922193
 0.4153435  0.03535901 0.07084033 0.05099133 0.02689893 0.01353992
 0.2012572  0.10884814 0.13261828 0.09033008 0.02105115 0.36269092
 0.46121203 0.18653857 0.08298083 0.08889661 0.1213103  0.32902637
 0.10617724 0.13214465 0.02324206 0.04296863 0.22714917 0.16556811
 0.07966138 0.51736194 0.22815784 0.04237109 0.3412102  1.30951184
 0.10917468 0.00800159 0.58517163 0.23383349 0.26694934 2.72721173
 0.0205321  0.67452466 0.4052373  0.312755   0.27754834]
5.791657838212683
```

```
surface.crs digitized
```

```
<xarray.DataArray (label: 47, digitized: 3)> Size: 1kB
[mm] 171.3 195.1 608.5 146.5 152.2 448.6 … 123.8 236.1 615.8 142.3 240.5 603.4
Coordinates:
  * label    (label) <U4 752B 'O-38' 'O-12' 'O-43' … 'O-44' 'O-36' 'O-42'
    type     (label) object 376B PointType.UNKNOWN … PointType.UNKNOWN
    group    (label) <U1 188B 'O' 'O' 'O' 'O' 'O' 'O' … 'O' 'O' 'O' 'O' 'O'
Dimensions without coordinates: digitized
```

Visualize the surface and extraced results.

[7]:
```
pvplt = pv.Plotter()
vbx.plot_surface(pvplt, surface_mesh, opacity=1.0)
vbx.plot_labeled_points(pvplt, sticker_centers, color="r")
vbx.plot_vector_field(pvplt, sticker_centers, normals)
vbx.camera_at_cog(pvplt, surface_mesh, (300,300,700),up=(1,0,0))
pvplt.show()
```

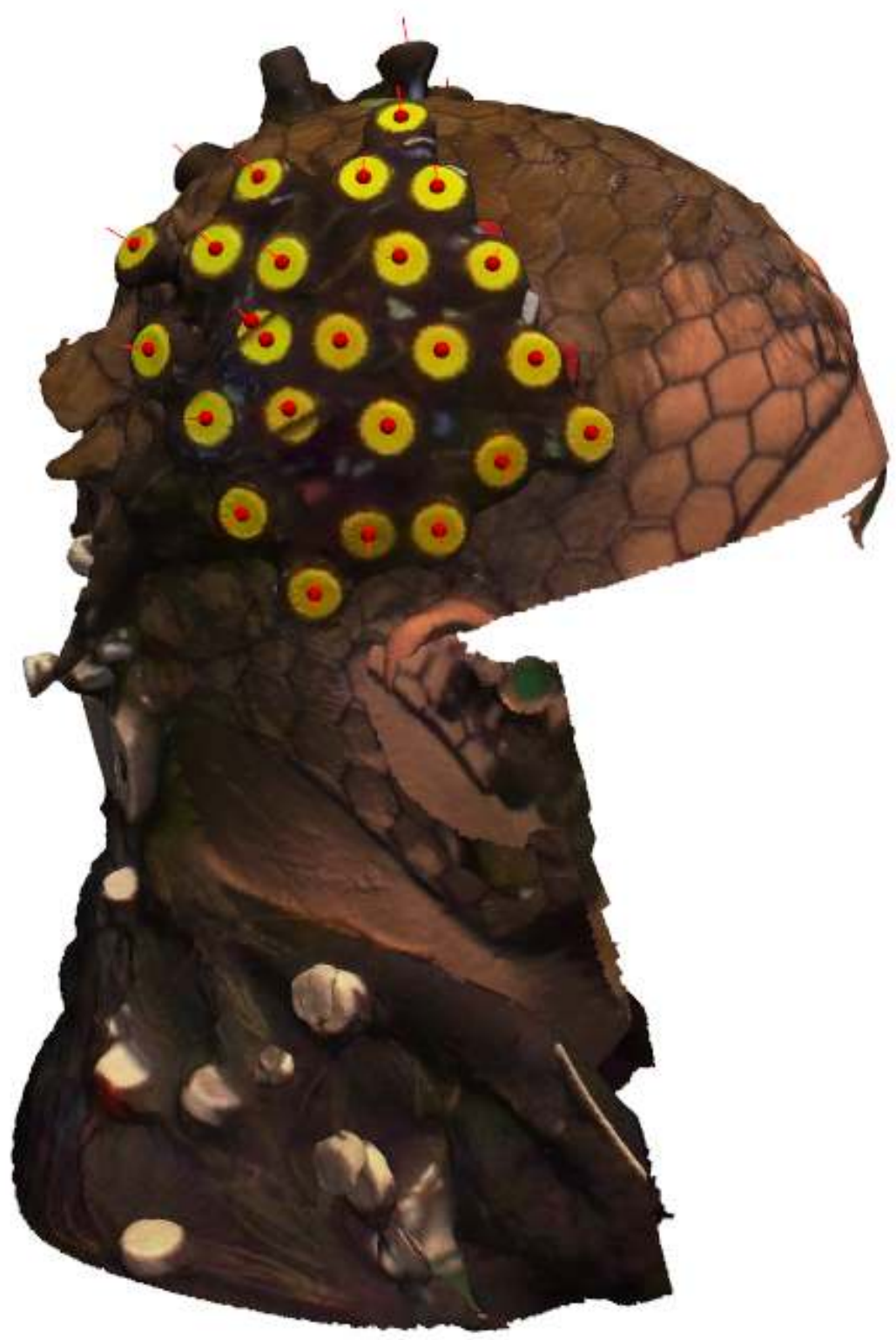

The details object is a container for debugging information and provides plotting functionality.

The following plots show for each cluster (tentative group of sticker vertices) The vertex positions perpendicular to the sticker normal as well as the minimum enclosing circle which is used to find the sticker's center.

[8]:
```
details.plot_cluster_circles()
```

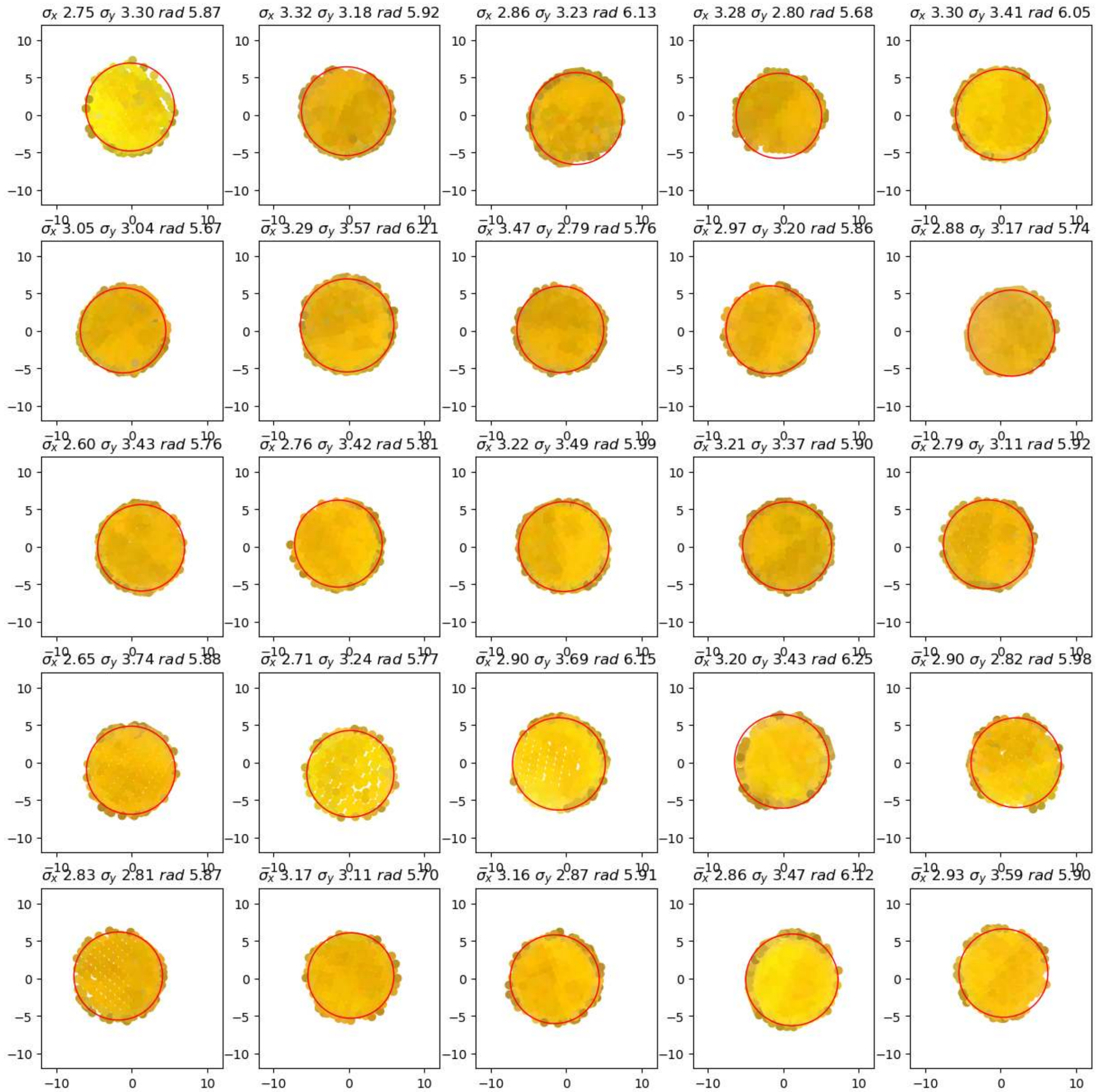
σx 2.75 σy 3.30 rad 5.87
σx 3.32 σy 3.18 rad 5.92
σx 2.86 σy 3.23 rad 6.13
σx 3.28 σy 2.80 rad 5.68
σx 3.30 σy 3.41 rad 6.05
σx 3.05 σy 3.04 rad 5.67
σx 3.29 σy 3.57 rad 6.21
σx 3.47 σy 2.79 rad 5.76
σx 2.97 σy 3.20 rad 5.86
σx 2.88 σy 3.17 rad 5.74
σx 2.60 σy 3.43 rad 5.76
σx 2.76 σy 3.42 rad 5.81
σx 3.22 σy 3.49 rad 5.99
σx 3.21 σy 3.37 rad 5.90
σx 2.79 σy 3.11 rad 5.92
σx 2.65 σy 3.74 rad 5.88
σx 2.71 σy 3.24 rad 5.77
σx 2.90 σy 3.69 rad 6.15
σx 3.20 σy 3.43 rad 6.25
σx 2.90 σy 2.82 rad 5.98
σx 2.83 σy 2.81 rad 5.87
σx 3.17 σy 3.11 rad 5.70
σx 3.16 σy 2.87 rad 5.91
σx 2.86 σy 3.47 rad 6.12
σx 2.93 σy 3.59 rad 5.90

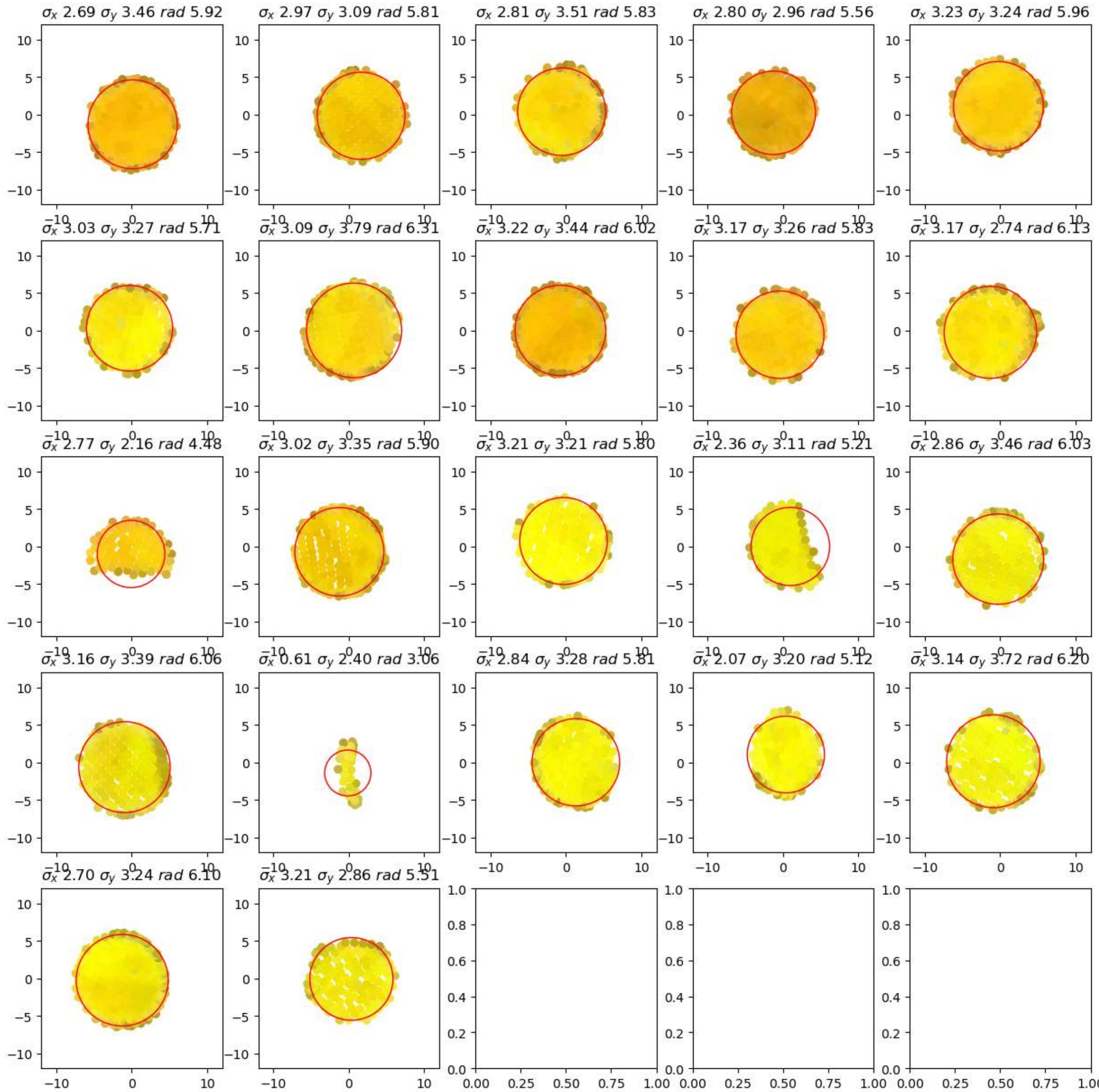

3. Manual corrections of sticker detection

If some optodes were misclassified or not all optodes were found automatically, there's way to remove or add them manually.

In this example, a cable crosses the sticker of optode "D28" on the right hemisphere. The `ColoredStickerProcessor` misclassified the single sticker as two.

The `OptodeSelect` class provides an interactive visualization of the head scan and the detected stickers (red spheres):

By clicking with the right mouse button on: - a sphere, a misidentified sticker can be removed. - somewhere on the surface, a new sticker position can be added.

Use this, to remove the erroneously placed red sphere.

[9]:
```
optode_selector = OptodeSelector(surface_mesh, sticker_centers, normals)
optode_selector.plot()
optode_selector.enable_picking()
vbx.plot_surface(optode_selector.plotter, surface_mesh, opacity=1.0)

optode_selector.plotter.show()
```

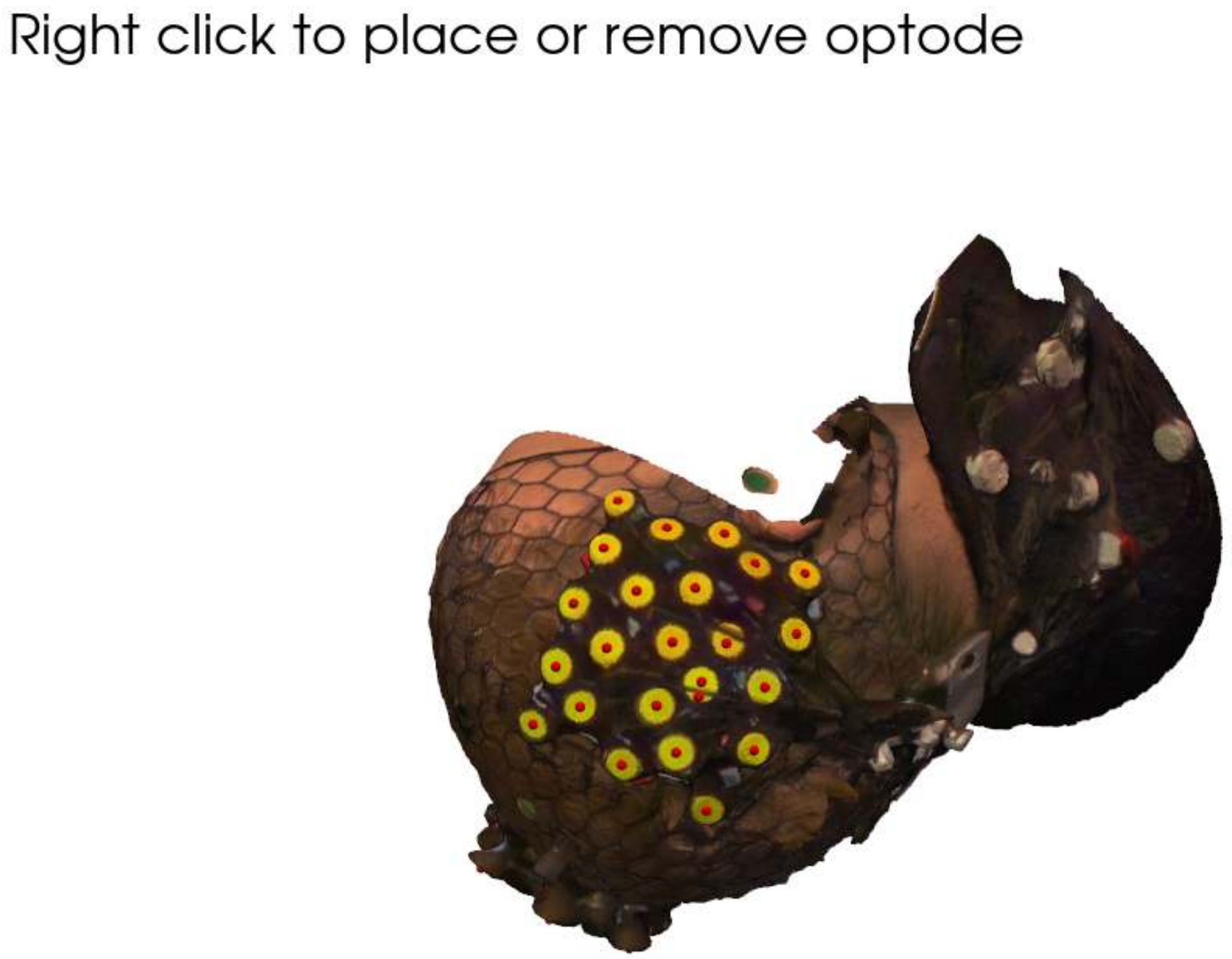

Interactions modify the `optode_selector.points` and `optode_selector.normals`. After selecting all optodes, update `sticker_centers` and `normals`:

[10]:
```
sticker_centers = optode_selector.points.copy()
normals = optode_selector.normals.copy()

if not INTERACTIVE:
    sticker_centers = sticker_centers.drop_sel(label="O-42")
    normals = normals.drop_sel(label="O-42")

display(sticker_centers)
```

```
<xarray.DataArray (label: 46, digitized: 3)> Size: 1kB
[mm] 171.3 195.1 608.5 146.5 152.2 448.6 … 184.3 195.8 589.3 123.8 236.1 615.8
Coordinates:
  * label     (label) <U4 736B 'O-38' 'O-12' 'O-43' … 'O-39' 'O-44' 'O-36'
    type      (label) object 368B PointType.UNKNOWN … PointType.UNKNOWN
```

```
    group    (label) <U1 184B 'O' 'O' 'O' 'O' 'O' 'O' … 'O' 'O' 'O' 'O' 'O'
Dimensions without coordinates: digitized
```

4. Project from sticker to scalp surface

Finally, to get from the sticker centers to the scalp coordinates we have to subtract the known lenght of the optodes in the direction of the normals:

[11]:
```
optode_length = 22.6 * cedalion.units.mm

scalp_coords = sticker_centers.copy()
mask_optodes = sticker_centers.group == "O"
scalp_coords[mask_optodes] = (
    sticker_centers[mask_optodes] - optode_length * normals[mask_optodes]
)
# we make a copy of this raw set of scalp coordinates to use later in the 2nd case of
# the coregistration example that showcases an alternative route if landmark-based
# coregistration fails
scalp_coords_altcase = scalp_coords.copy()

display(scalp_coords)
```

```
<xarray.DataArray (label: 46, digitized: 3)> Size: 1kB
[mm] 155.1 187.1 595.0 136.7 153.6 468.9 … 165.2 189.2 579.2 117.1 222.3 599.2
Coordinates:
  * label    (label) <U4 736B 'O-38' 'O-12' 'O-43' … 'O-39' 'O-44' 'O-36'
    type     (label) object 368B PointType.UNKNOWN … PointType.UNKNOWN
    group    (label) <U1 184B 'O' 'O' 'O' 'O' 'O' 'O' … 'O' 'O' 'O' 'O' 'O'
Dimensions without coordinates: digitized
```

Visualize sticker centers (red) and scalp coordinates (green).

[12]:
```
pvplt = pv.Plotter()
vbx.plot_surface(pvplt, surface_mesh, opacity=0.3)
vbx.plot_labeled_points(pvplt, sticker_centers, color="r")
vbx.plot_labeled_points(pvplt, scalp_coords, color="g")
vbx.plot_vector_field(pvplt, sticker_centers, normals)
pvplt.show()
```

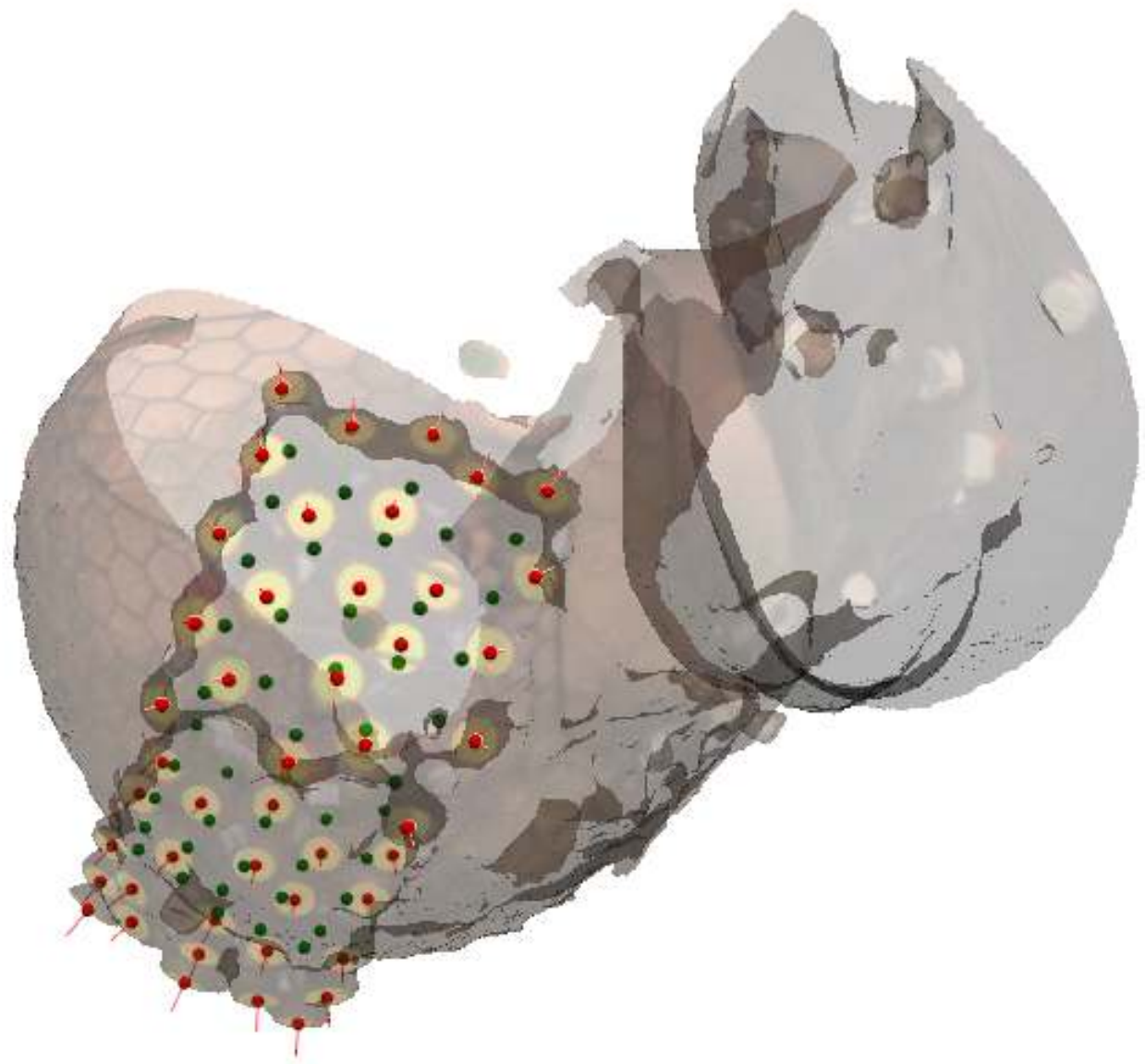

5. Specify landmarks on scanned head surface

5.1. Pick positions in interactive plot

When using the `plot_surface` function with parameter `pick_landmarks` set to *True*, the plot becomes interactive and allows to pick the positions of 5 landmarks. These are “Nz”, “Iz”, “Cz”, “Lpa”, “RpA”.

After clicking on the mesh, a green sphere marks the picked location. The sphere has a label attached. If this label is not visible, try to zoom further into the plot (mouse wheel). By clicking again with right mouse button on the sphere one can cycle through the different labels or remove a misplaced landmark.

It helps to add colored markers at the landmark positions when preparing the subject. Here green (Nz, Iz, Lpa, Rpa) and yellow (Cz) stickers where used. If the face is cropped from the scanned mesh for anonymization, make sure to preserve these stickers.

[13]:
```
pvplt = pv.Plotter()
get_landmarks = vbx.plot_surface(pvplt, surface_mesh, opacity=1.0, pick_landmarks=True)
pvplt.show()
```

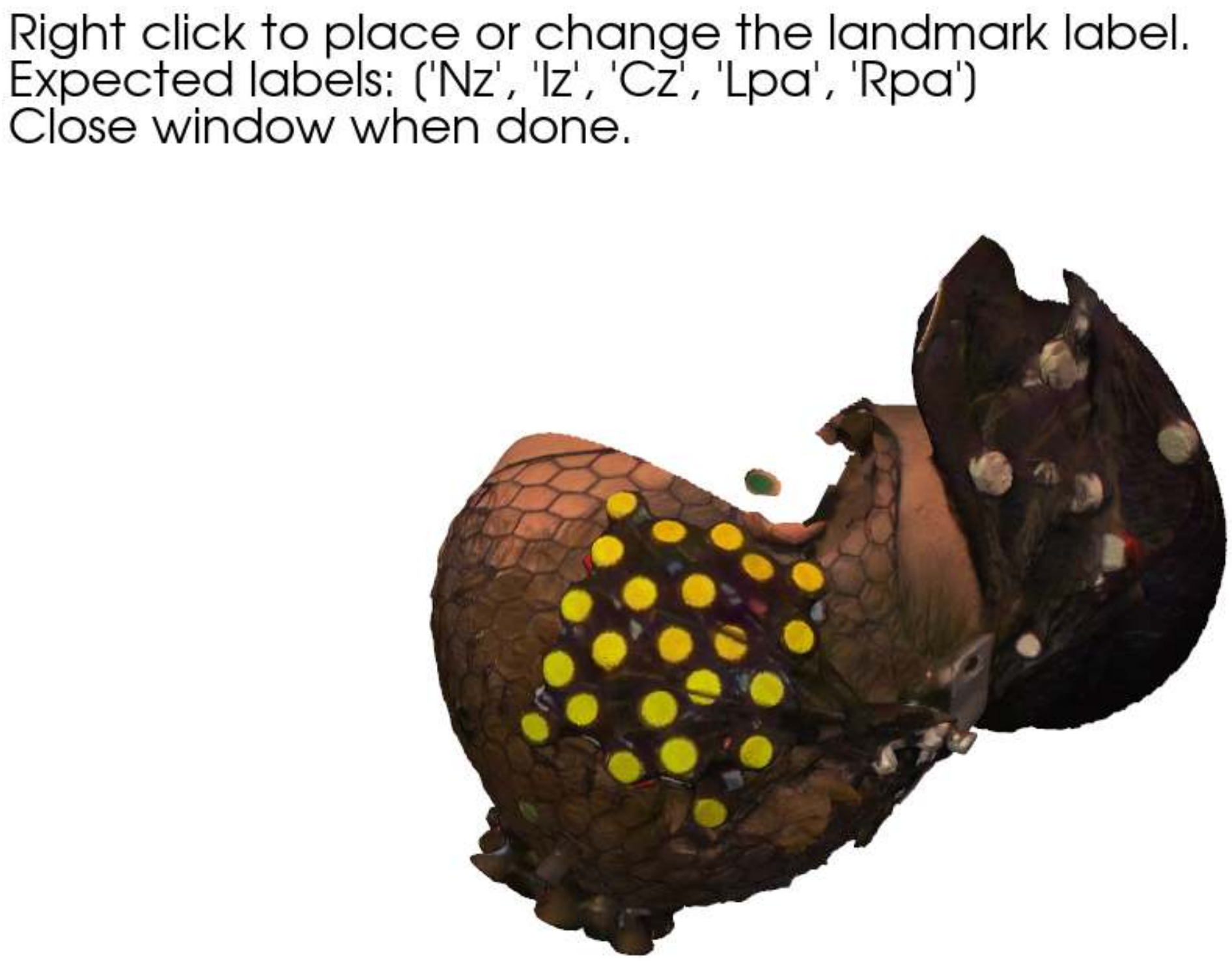

5.2. Retrieve picked positions from interactive plot

The `plot_surface` function returns a function `get_landmarks`. Call this function to obtain: * 1st value - coordinates of picked landmarks * 2nd - labels of corresponding landmarks

[14]:
```python
if INTERACTIVE:
    landmarks = get_landmarks()
else:
    # For documentation purposes and to enable automatically rendered example notebooks
    # we provide the hand-picked coordinates here, too.
    landmarks = xr.DataArray(
        [
            [ 68.64,  58.49, 594.20],
            [ 33.50, 249.85, 518.98],
            [176.69, 175.61, 530.85],
            [ 37.48, 107.10, 489.61],
            [ 48.39, 175.45, 624.98],
        ],
        dims=["label", "digitized"],
        coords={
            "label": ("label", ["Nz", "Iz", "Cz", "Lpa", "Rpa"]),
            "type": ("label", [cdc.PointType.LANDMARK] * 5),
            "group": ("label", ["L"] * 5),
```

```
        },
    ).pint.quantify("mm")

display(landmarks)

assert len(set(landmarks.label.values)) == 5, "please select 5 landmarks"
```

```
<xarray.DataArray (label: 5, digitized: 3)> Size: 120B
[mm] 68.64 58.49 594.2 33.5 249.8 519.0 … 37.48 107.1 489.6 48.39 175.4 625.0
Coordinates:
  * label    (label) <U3 60B 'Nz' 'Iz' 'Cz' 'Lpa' 'Rpa'
    type     (label) object 40B PointType.LANDMARK … PointType.LANDMARK
    group    (label) <U1 20B 'L' 'L' 'L' 'L' 'L'
Dimensions without coordinates: digitized
```

6. Mapping the scanned optode positions to a predefined montage.

So far the optode positions found in the photogrammetric head scan carry only generic labels. In oder to identify them, they must be matched with a definition of the actual montage.

Snirf files store next to the actual time series data also the probe geometry, i.e. 3D coordinates of each source and detector. To label the optodes found in the photogrammetric scan, we map each optode to its counterpart in the snirf file.

The snirf coordinates are written during the data acquisition and are typically obtained by arranging the montage on a template head like ICBM-152 or colin27. So despite their similarity, the probe geometries in the snirf file and those from the head scan have differences because of different head geometries and different coordinate systems.

6.1 Load the montage information from .snirf file

[15]:
```
# read the example snirf file. Specify a name for the coordinate reference system.
if not fname_snirf:
    rec = cedalion.data.get_fingertappingDOT(crs="montage")
else:
    rec = cedalion.io.read_snirf(fname_snirf, crs="montage")[0]

# read 3D coordinates of the optodes
montage_elements = rec.geo3d

# landmark labels must match exactly. Adjust case where they don't match.
montage_elements = montage_elements.points.rename({"LPA": "Lpa", "RPA": "Rpa"})
```

6.2 Find a transformation to align selected landmarks to montage coordinates

The coordinates in the snirf file and from the photogrammetric scan use different coordinate reference systems (CRS). In Cedalion the user needs to explicitly name different CRSs. Here the labels 'digitized' and 'montage' were used.

The following plot shows the probe geometry from the snirf file and the landmarks from the head scan. Two black lines Nz-Iz and Lpa-Rpa are added to guide the eye.

[16]:
```
f = plt.figure(figsize=(12,5))
ax1 = f.add_subplot(1,2,1, projection="3d")
```

```
ax2 = f.add_subplot(1,2,2, projection="3d")
colors = {cdc.PointType.SOURCE: "r", cdc.PointType.DETECTOR: "b"}
sizes = {cdc.PointType.SOURCE: 20, cdc.PointType.DETECTOR: 20}

for i, (type, x) in enumerate(montage_elements.groupby("type")):
    x = x.pint.to("mm").pint.dequantify()
    ax1.scatter(x[:, 0], x[:, 1], x[:, 2], c=colors.get(type, "g"), s=sizes.get(type,␣
 ↪2))

for i, (type, x) in enumerate(landmarks.groupby("type")):
    x = x.pint.to("mm").pint.dequantify()
    ax2.scatter(x[:, 0], x[:, 1], x[:, 2], c=colors.get(type, "g"), s=20)

for ax, points in [(ax1, montage_elements), (ax2, landmarks)]:
    points = points.pint.to("mm").pint.dequantify()
    ax.plot([points.loc["Nz",0], points.loc["Iz",0]],
            [points.loc["Nz",1], points.loc["Iz",1]],
            [points.loc["Nz",2], points.loc["Iz",2]],
            c="k"
            )
    ax.plot([points.loc["Lpa",0], points.loc["Rpa",0]],
            [points.loc["Lpa",1], points.loc["Rpa",1]],
            [points.loc["Lpa",2], points.loc["Rpa",2]],
            c="k"
            )

ax1.set_title(f"from snirf | crs: {montage_elements.points.crs}")
ax2.set_title(f"from scan | crs: {landmarks.points.crs}");
```

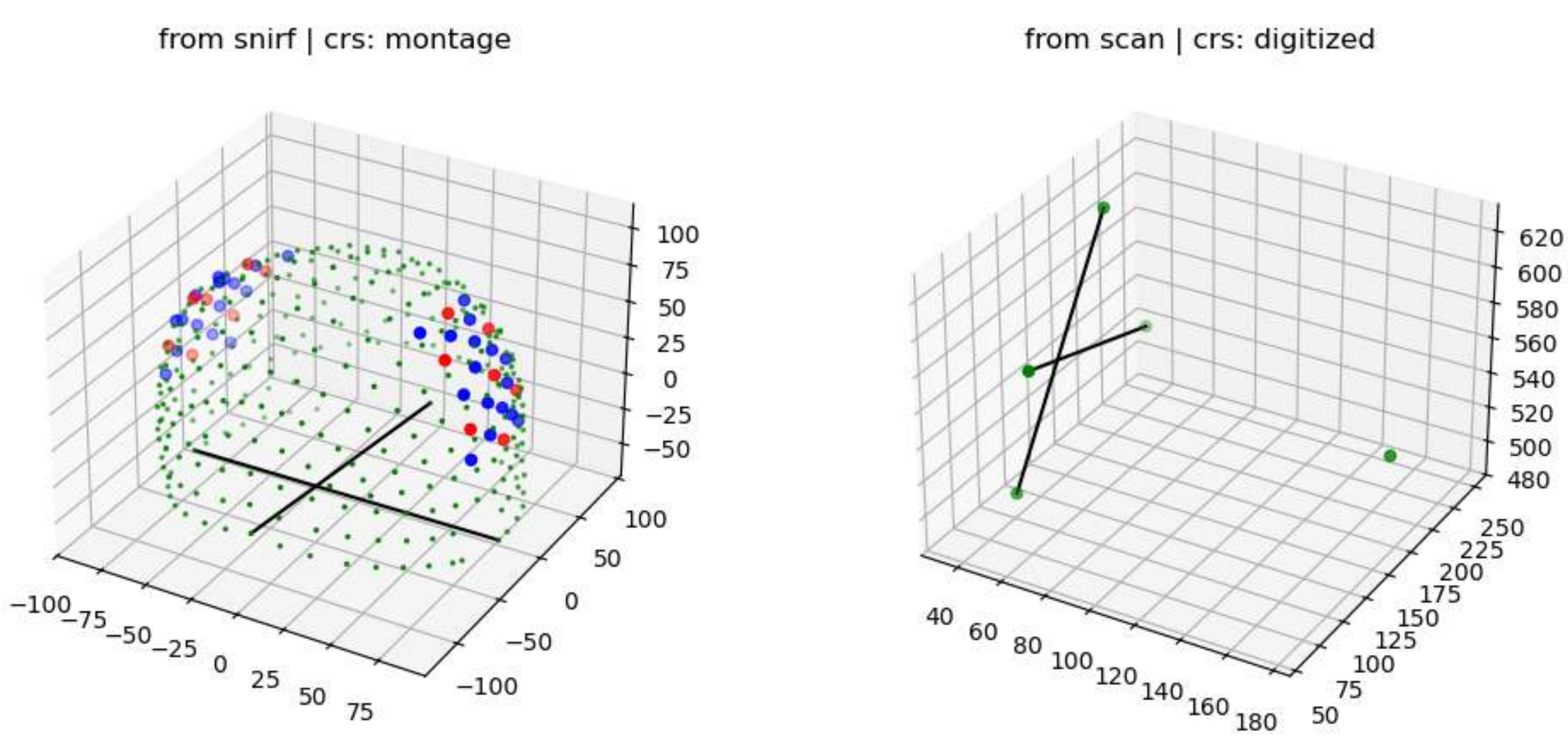

Subsequently, to bring the coordinates into the same space, from the landmarks a transformation (translations and rotations) is derived. This transforms the coordinates from the snirf file to the CRS of the photogramettric scan.

The following plot illustrates the transformed coordinates of sources (red) and detectors (blue). Deviations between these coordinates and the head surface are expected, since the optode positions where specified on a different head geometry.

[17]:
```
trafo = cedalion.geometry.registration.register_trans_rot(landmarks, montage_elements)

filtered_montage_elements = montage_elements.where(
    (montage_elements.type == cdc.PointType.SOURCE)
    | (montage_elements.type == cdc.PointType.DETECTOR),
    drop=True,
)
filtered_montage_elements_t = filtered_montage_elements.points.apply_transform(trafo)

pvplt = pv.Plotter()
vbx.plot_surface(pvplt, surface_mesh, color="w", opacity=.2)
vbx.plot_labeled_points(pvplt, filtered_montage_elements_t)
pvplt.show()
```

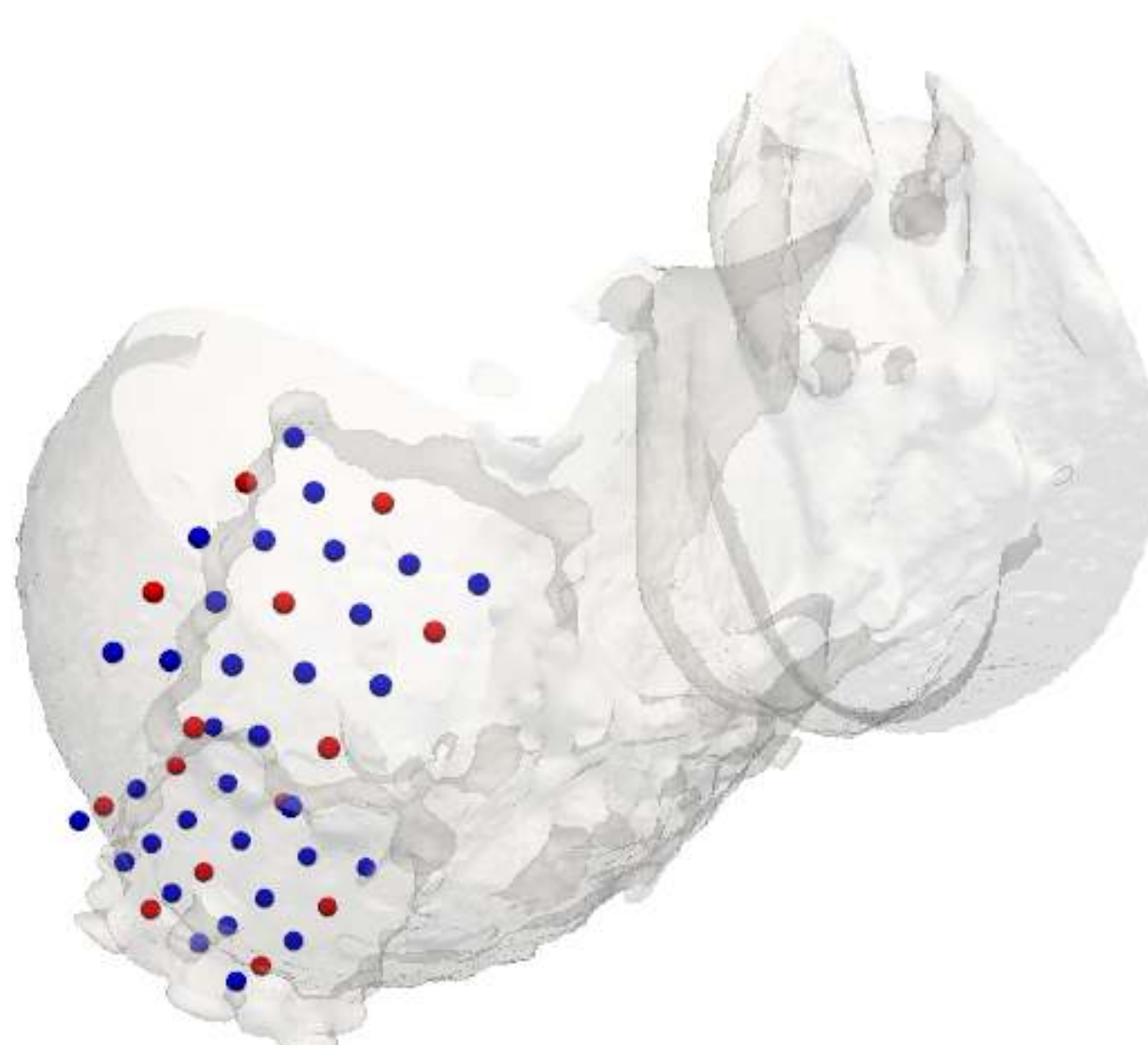

6.3 Iterative closest point algorithm to find labels for detected optode centers

Finally, the mapping is derived by iteratively trying to find a transformation that yilds the best match between the snirf and the scanned coordinates.

The following plot visualizes the result: * Green points represent optode centers * Next to them there shall

be labels assumed by ICP algorithm (*show_labels* = *True*)

[18]:
```
# iterative closest point registration
idx = cedalion.geometry.registration.icp_with_full_transform(
    scalp_coords, filtered_montage_elements_t, max_iterations=100
)

# extract labels for detected optodes
label_dict = {}
for i, label in enumerate(filtered_montage_elements.coords["label"].values):
    label_dict[i] = label
labels = [label_dict[index] for index in idx]

# write labels to scalp_coords
scalp_coords = scalp_coords.assign_coords(label=labels)

# add landmarks
geo3Dscan = geo3d_from_scan(scalp_coords, landmarks)

display(geo3Dscan)
```

```
<xarray.DataArray (label: 51, digitized: 3)> Size: 1kB
[mm] 155.1 187.1 595.0 136.7 153.6 468.9 … 37.48 107.1 489.6 48.39 175.4 625.0
Coordinates:
    type     (label) object 408B PointType.SOURCE … PointType.LANDMARK
    group    (label) <U1 204B 'O' 'O' 'O' 'O' 'O' 'O' … 'L' 'L' 'L' 'L' 'L'
  * label    (label) <U3 612B 'S9' 'D9' 'D23' 'D6' … 'Iz' 'Cz' 'Lpa' 'Rpa'
Dimensions without coordinates: digitized
Attributes:
    units:    mm
```

[19]:
```
f,ax = plt.subplots(1,2, figsize=(12,6))
cedalion.vis.anatomy.scalp_plot(
    rec["amp"],
    montage_elements,
    cedalion.nirs.channel_distances(rec["amp"], montage_elements),
    ax=ax[0],
    optode_labels=True,
    cb_label="channel dist. / mm",
    cmap="plasma",
    vmin=25,
    vmax=42,
)
ax[0].set_title("montage from snirf file")
cedalion.vis.anatomy.scalp_plot(
    rec["amp"],
    geo3Dscan,
    cedalion.nirs.channel_distances(rec["amp"], geo3Dscan),
    ax=ax[1],
    optode_labels=True,
    cb_label="channel dist. / mm",
    cmap="plasma",
```

```
    vmin=25,
    vmax=42,
)
ax[1].set_title("montage from photogrammetric scan")
plt.tight_layout()
```

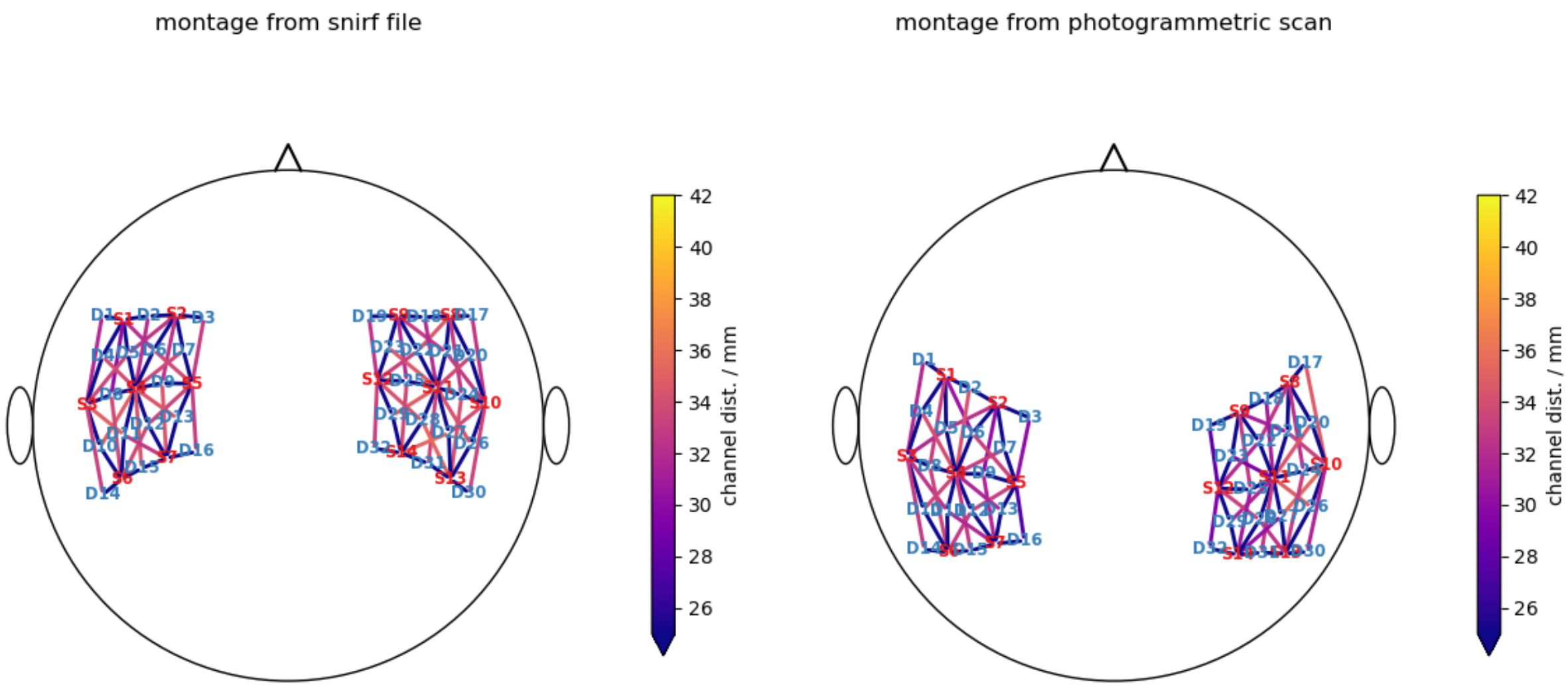

Visualization of successfull assignment *(show_labels = True)*

[20]:
```
pvplt = pv.Plotter()
cedalion.vis.anatomy.plot_brain_and_scalp(None, surface_mesh.mesh, None, None,␣
 ↪plotter=pvplt)
vbx.plot_labeled_points(pvplt, geo3Dscan, show_labels=True)
pvplt.show()
```

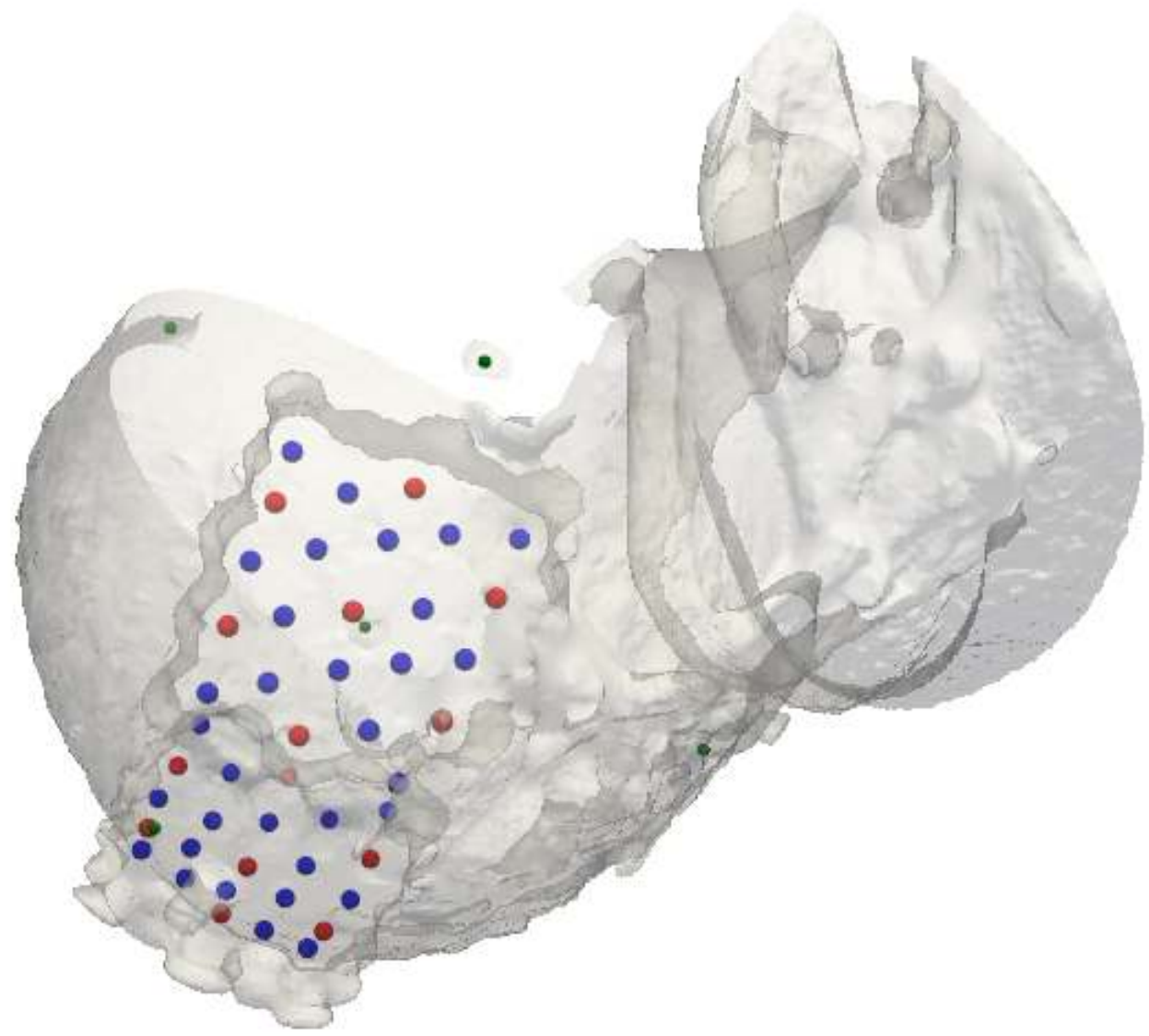

6.4 Alternative approach without landmarks

Mapping the optode labels can fail for example because of a bad landmark selection.

In such cases it is possible to find a new transformation by manually labeling three optodes. This is done by selecting them in a given order. For that it helps to have a visualization of the montage of your experiment.

[21]:
```
if fname_montage_img:
    # Load and display the image
    img = mpimg.imread(fname_montage_img)
    plt.figure(figsize=(12, 10))
    plt.imshow(img)
    plt.axis("off")  # Turn off axis labels and ticks
    plt.show()
else:
    print("No montage image specified.")
```

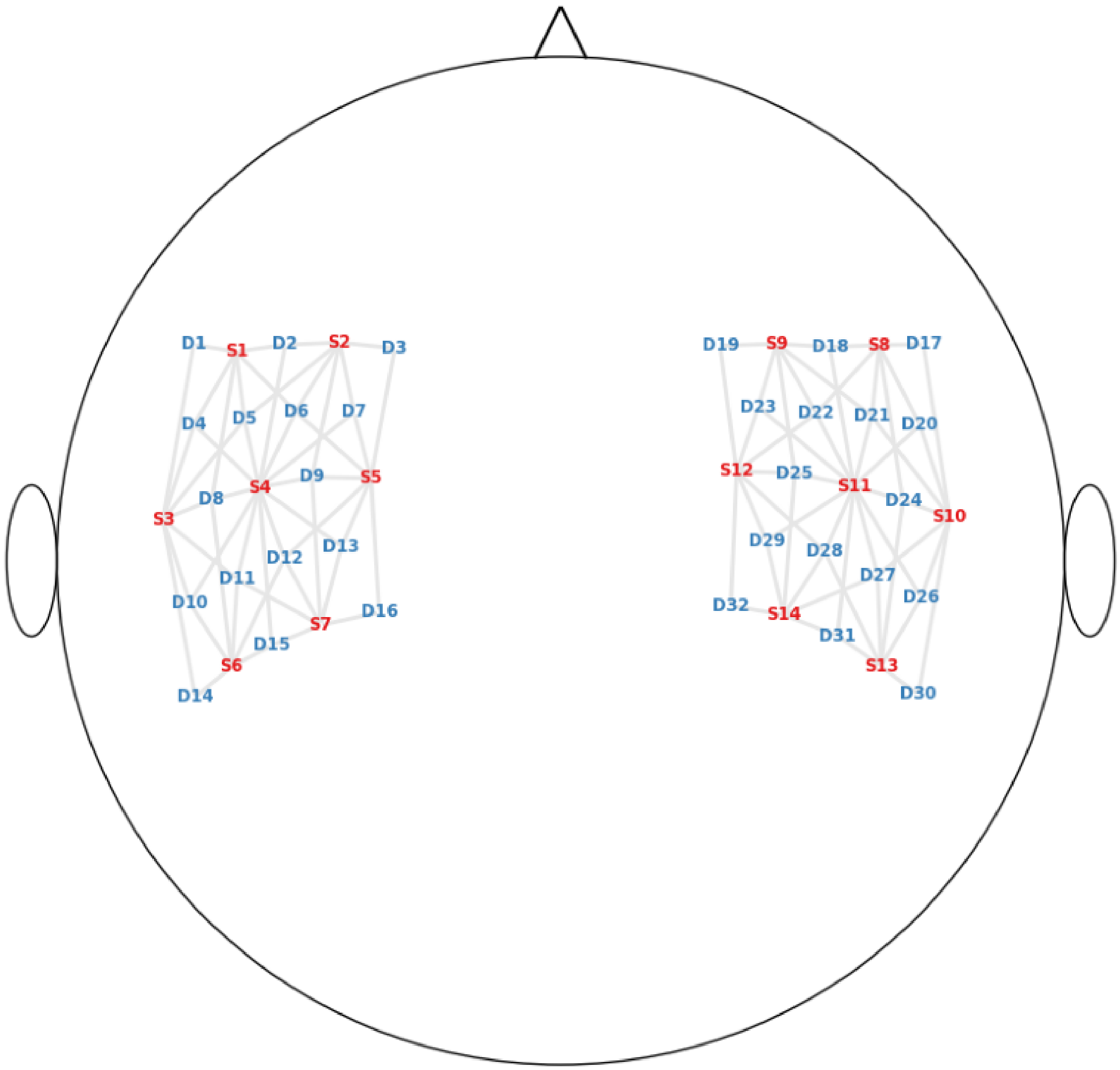

Search for three optodes that are evenly spreaded across the head surface. Afterwards prompt the user to right click on each of them.

[22]:
```
spread_point_labels = find_spread_points(filtered_montage_elements)
print("Select those points")
print(spread_point_labels)

points = []
pvplt = pv.Plotter()
vbx.plot_surface(pvplt, surface_mesh, opacity=1.0)
vbx.plot_labeled_points(pvplt, sticker_centers, color="r", ppoints = points)
pvplt.show()
```

```
/opt/miniconda3/envs/cedalion_250922/lib/python3.11/site-
packages/xarray/core/variable.py:315: UnitStrippedWarning: The unit of the quantity is
```

```
stripped when downcasting to ndarray.
  data = np.asarray(data)
```

```
Select those points
['S1' 'D32' 'D10']
```

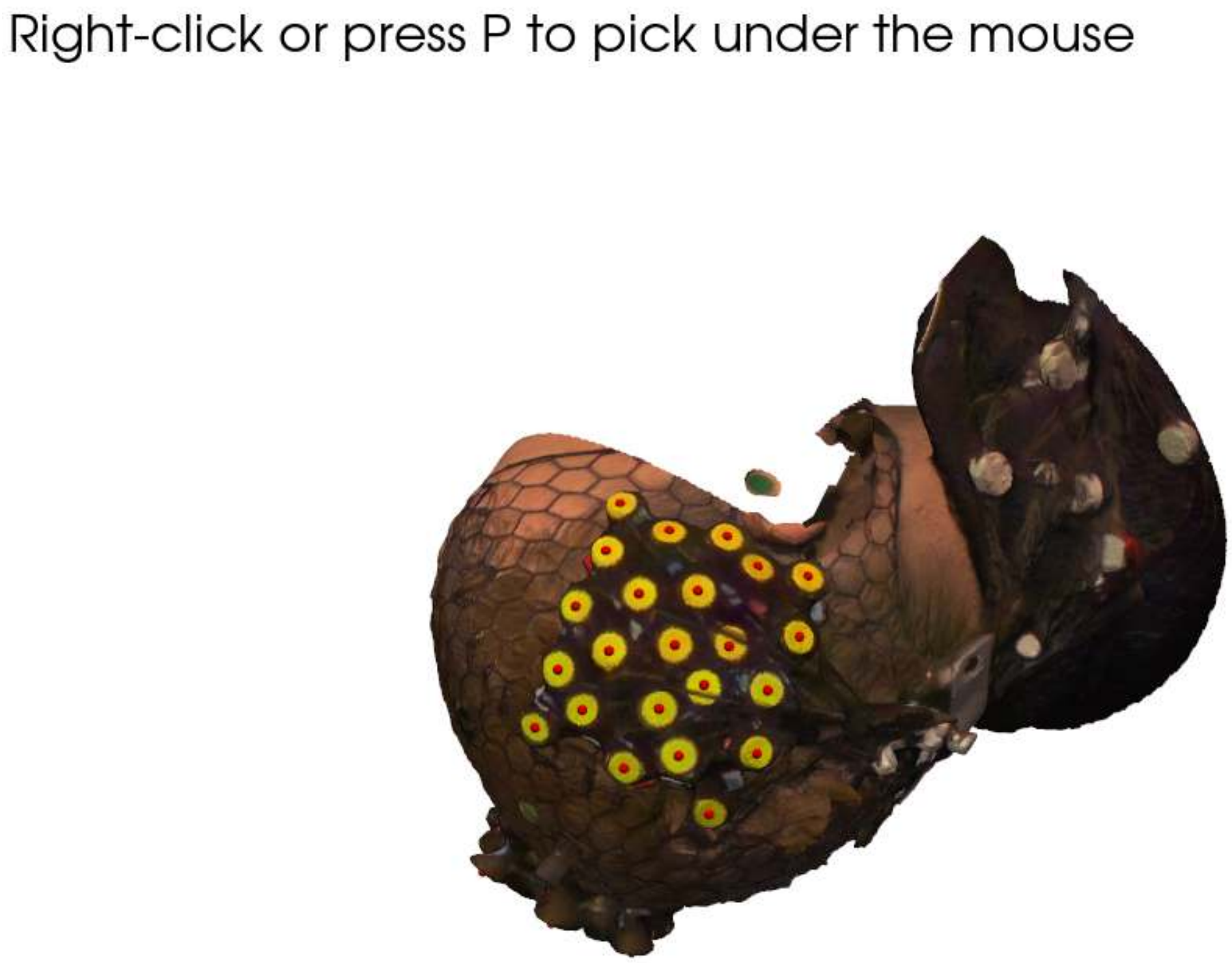

Retrieve picked positions:

[23]:
```python
if INTERACTIVE:
    labeled_points = points
else:
    # For documentation purposes and to enable automatically rendered example notebooks
    # we provide the hand-picked coordinates here, too.
    labeled_points = [13, 12, 6]

display(labeled_points)

if len(labeled_points) != 3:
    raise ValueError("Please select the 3 specified points.")
```

```
[13, 12, 6]
```

Write the selected labels to the corresponding points of `xarray.DataArray` scalp_coords:

[24]:
```
new_labels = scalp_coords_altcase.label.values.copy()
for i, idx in enumerate(labeled_points):
    new_labels[idx] = spread_point_labels[i]
scalp_coords_altcase = scalp_coords_altcase.assign_coords(label=new_labels)
scalp_coords_altcase
```

[24]:
```
<xarray.DataArray (label: 46, digitized: 3)> Size: 1kB
[mm] 155.1 187.1 595.0 136.7 153.6 468.9 … 165.2 189.2 579.2 117.1 222.3 599.2
Coordinates:
    type     (label) object 368B PointType.UNKNOWN … PointType.UNKNOWN
    group    (label) <U1 184B 'O' 'O' 'O' 'O' 'O' 'O' … 'O' 'O' 'O' 'O' 'O'
  * label    (label) <U4 736B 'O-38' 'O-12' 'O-43' … 'O-39' 'O-44' 'O-36'
Dimensions without coordinates: digitized
```

Find the affine transformation for the newly labeled points and apply it to the montage optodes

[25]:
```
trafo2 = cedalion.geometry.registration.register_trans_rot(
    scalp_coords_altcase, montage_elements
)

filtered_montage_elements = montage_elements.where(
    (montage_elements.type == cdc.PointType.SOURCE)
    | (montage_elements.type == cdc.PointType.DETECTOR),
    drop=True,
)
filtered_montage_elements_t = filtered_montage_elements.points.apply_transform(trafo2)
```

and run ICP algorithm for label assignment once again, extract labels for detected optodes and plot the results

[26]:
```
# iterative closest point registration
idx = cedalion.geometry.registration.icp_with_full_transform(
    scalp_coords_altcase, filtered_montage_elements_t, max_iterations=100
)
# extract labels for detected optodes
label_dict = {}
for i, label in enumerate(filtered_montage_elements.coords["label"].values):
    label_dict[i] = label
labels = [label_dict[index] for index in idx]

# write labels to scalp_coords
scalp_coords_altcase = scalp_coords_altcase.assign_coords(label=labels)

# add landmarks
geo3Dscan_alt = geo3d_from_scan(scalp_coords_altcase, landmarks)
```

[27]:
```
f,ax = plt.subplots(1,2, figsize=(12,6))
cedalion.vis.anatomy.scalp_plot(
    rec["amp"],
    montage_elements,
    cedalion.nirs.channel_distances(rec["amp"], montage_elements),
    ax=ax[0],
    optode_labels=True,
```

```
    cb_label="channel dist. / mm",
    cmap="plasma",
    vmin=25,
    vmax=42,
)

ax[0].set_title("montage from snirf file")
cedalion.vis.anatomy.scalp_plot(
    rec["amp"],
    geo3Dscan_alt,
    cedalion.nirs.channel_distances(rec["amp"], geo3Dscan_alt),
    ax=ax[1],
    optode_labels=True,
    cb_label="channel dist. / mm",
    cmap="plasma",
    vmin=25,
    vmax=42,
)
ax[1].set_title("montage from photogrammetric scan")
plt.tight_layout()
```

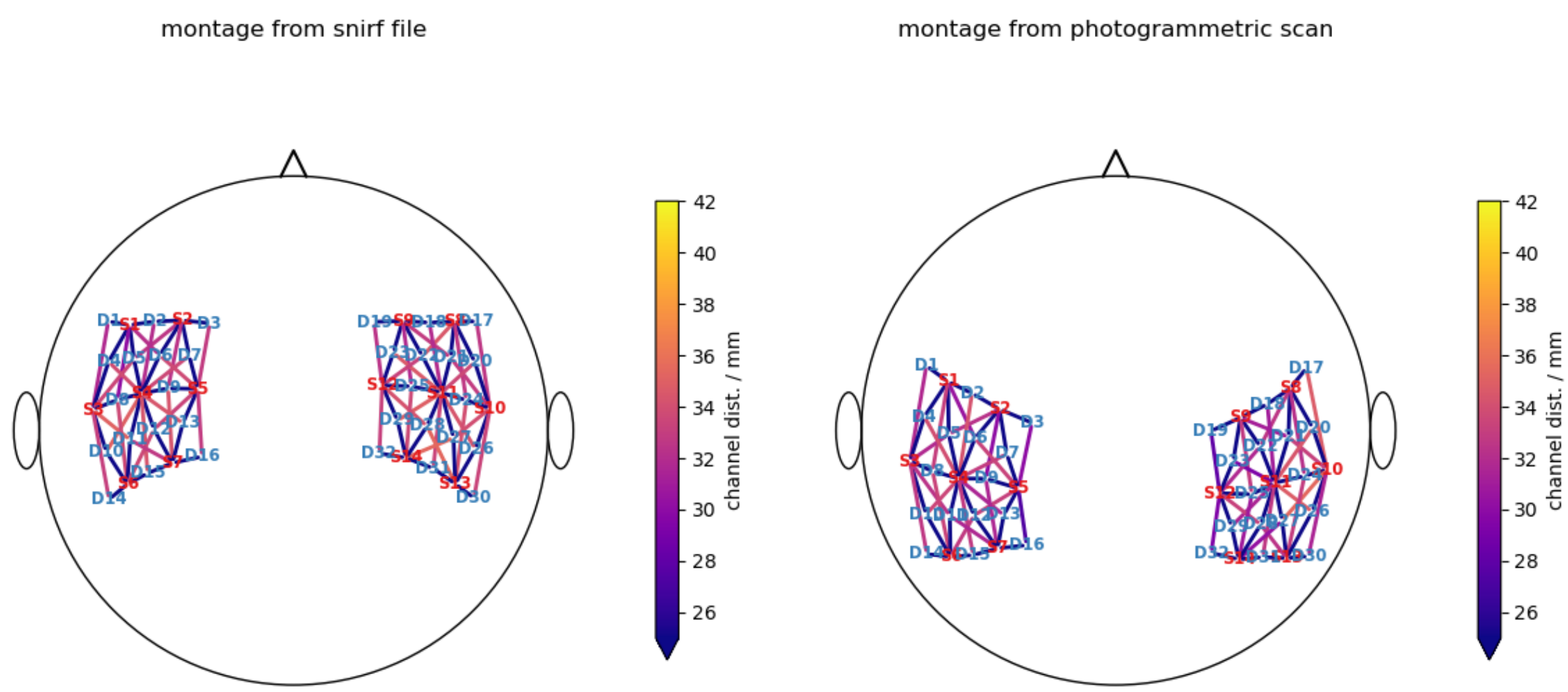

7. Saving the result

Finally, the detected optode positions are stored to disk for later processing. Two approaches are shown: storing the coordinates in a BIDS-compliant TSV file, or storing them in a modified copy of the original SNIRF file.

Storing coordinates in a BIDS-compliant TSV file

[28]:
```
with TemporaryDirectory() as output_dir:
    # write result to temporary tsv file
    output_dir = Path(output_dir)
    tsv_filename = output_dir / "sub-01_optodes.tsv"

    cedalion.io.bids.export_to_bids_optodes_tsv(
        tsv_filename, geo3Dscan, float_format=".2f"
    )

    # reread written file and print first 10 lines
    with tsv_filename.open("r") as fin:
        print("".join(fin.readlines()[:10]))
```

```
name    type    x       y       z
S9      source  155.13  187.07  594.99
D9      detector        136.73  153.57  468.89
D23     detector        155.98  203.59  585.50
D6      detector        135.87  136.81  474.79
S2      source  148.13  133.26  487.91
D13     detector        138.09  172.12  465.88
D10     detector        88.19   145.23  462.33
S11     source  132.55  211.85  600.92
D21     detector        129.83  195.07  611.31
```

Storing coordinates in a SNIRF file

[29]:
```
with TemporaryDirectory() as output_dir:
    # take the original recording container and replace its geo3d attribute
    rec = cedalion.data.get_fingertappingDOT()
    rec.geo3d = geo3Dscan

    # write the result to a temporary snirf file
    output_dir = Path(output_dir)
    snirf_filename = output_dir / "snirf_with_scanned_optodes.snirf"
    cedalion.io.write_snirf(snirf_filename, rec)

    # reread the written file
    # SNIRF files do not label the coordinate reference system. Here, we specify the
    # name manually to match what we used before.
    loaded_rec = cedalion.io.read_snirf(snirf_filename, crs="digitized")[0]
    geo3d_loaded = loaded_rec.geo3d

    # the order of optodes in snirf's probe info. element may differ from geo3Dscan
    # assert that all optodes exist
    assert(sorted(geo3d_loaded.label.values) == sorted(geo3Dscan.label.values))

    # assert that all optode coordinates match geo3Dscan
    assert((geo3d_loaded.loc[geo3Dscan.label] == geo3Dscan).all())
```

References

[30]:
```
cedalion.bib.dump_to_notebook()
```

```
<IPython.core.display.HTML object>
```

Next: 3 — Signal Processing — the full preprocessing pipeline.

S3: Signal Processing

This notebook demonstrates Cedalion's capabilities to assess signal quality and correct motion artifacts.

Several signal quality metrics are implemented in the package `cedalion.sigproc.quality`. From these metrics boolean masks are created which indicate whether the quality of a given time point or segment is acceptable or not. By combining these boolean masks, complex selection criteria can be formulated.

Learning objectives

In this notebook you will learn to:

- Apply a complete fNIRS preprocessing pipeline: quality assessment → OD conversion → motion correction → filtering → haemoglobin concentration
- Compute and apply SCI and SNR quality masks to prune bad channels
- Use TDDR and wavelet correction for motion artefacts
- Understand how each preprocessing step changes the signal

[1]:
```
# This cells setups the environment when executed in Google Colab.
try:
    import google.colab
    !curl -s https://raw.githubusercontent.com/ibs-lab/cedalion/dev/scripts/
↪colab_setup.py -o colab_setup.py
    # Select branch with --branch "branch name" (default is "dev")
    %run colab_setup.py
except ImportError:
    pass
```

[2]:
```
import cedalion
import cedalion.data
import cedalion.sigproc.motion as motion
import cedalion.sigproc.quality as quality
import cedalion.vis.anatomy
import cedalion.vis.blocks as vbx
import cedalion.vis.colors as colors
import matplotlib.pyplot as p
import numpy as np
import pandas as pd
import xarray as xr
from cedalion import units
from cedalion.vis.quality import plot_quality_mask

xr.set_options(display_expand_data=False)
```

[2]:
```
<xarray.core.options.set_options at 0x7f7805640210>
```

Load Data

The example starts by loading an example datasets via `cedalion.data`:

[3]:
```
rec = cedalion.data.get_fingertappingDOT()
```

Recording Container

The `Recording` container carries time series and related objects through the program in ordered dictionaries.

The dataset contains a single time series of fNIRS raw amplitudes. This is stored in the attribute `rec.timeseries` with key `'amp'`:

[4]:
```
rec.timeseries.keys()
```

[4]:
```
odict_keys(['amp'])
```

Among the data stored in the `Recording`container, the time series are access most frequently. Therefore, a shortcut is provided. The user can access items in `.timeseries` directly on the `Recording` container:

[5]:
```
rec["amp"] is rec.timeseries["amp"]
```

[5]:
```
True
```

Next to the fNIRS time series, the dataset contains also time series data from auxiliary sensors which are stored in `.aux_ts`:

[6]:
```
rec.aux_ts.keys()
```

[6]:
```
odict_keys(['ACCEL_X_1', 'ACCEL_Y_1', 'ACCEL_Z_1', 'GYRO_X_1', 'GYRO_Y_1', 'GYRO_Z_1',
'ExGa1', 'ExGa2', 'ExGa3', 'ExGa4', 'ECG', 'Respiration', 'PPG', 'SpO2', 'Heartrate',
'GSR', 'Temperature'])
```

Information about stimulus events is stored in a `pandas.DataFrame` under `.stim`. For each stimulus event the onset and duration is stored in seconds. Each event also includes a value indicating stimulus strength (e.g., the loudness of an auditory stimulus), which can be used to scale the amplitude of the modeled hemodynamic response. Finally, the trial type string label allows distinguishing different event types.

[7]:
```
rec.stim
```

[7]:
```
           onset  duration  value trial_type
0      23.855104      10.0    1.0          1
1      54.132736      10.0    1.0          1
2      84.410368      10.0    1.0          1
3     114.688000      10.0    1.0          1
4     146.112512      10.0    1.0          1
..           …         …     …          …
125  1431.535616      10.0    1.0          5
126  1526.038528      10.0    1.0          5
127  1650.819072      10.0    1.0          5
128  1805.418496      10.0    1.0          5
129  1931.116544      10.0    1.0          5

[130 rows x 4 columns]
```

When loading SNIRF files, trial types are derived from numeric stimulus markers. Cedalion registers the accessor `.cd` on DataFrames through which the user can modify stimulus dataframes. In the following the function `.cd.rename_events` is used to translate the numeric stimulus markers into descriptive names.

Users are free to choose any labels, but a consistent naming scheme is recommended because it makes selecting events easier. The dataset contains two motor tasks: finger tapping and ball squeezing executed both with the left and right hand:

[8]:
```python
rec.stim.cd.rename_events(
    {
        "1": "Rest",
        "2": "FTapping/Left",
        "3": "FTapping/Right",
        "4": "BallSqueezing/Left",
        "5": "BallSqueezing/Right",
    }
)

rec.stim
```

[8]:
```
            onset  duration  value           trial_type
0       23.855104      10.0    1.0                 Rest
1       54.132736      10.0    1.0                 Rest
2       84.410368      10.0    1.0                 Rest
3      114.688000      10.0    1.0                 Rest
4      146.112512      10.0    1.0                 Rest
..            …         …     …                    …
125   1431.535616      10.0    1.0  BallSqueezing/Right
126   1526.038528      10.0    1.0  BallSqueezing/Right
127   1650.819072      10.0    1.0  BallSqueezing/Right
128   1805.418496      10.0    1.0  BallSqueezing/Right
129   1931.116544      10.0    1.0  BallSqueezing/Right

[130 rows x 4 columns]
```

Selecting all BallSqueezing tasks:

[9]:
```python
with pd.option_context("display.max_rows", 5):
    display(rec.stim[rec.stim.trial_type.str.startswith("BallSqueezing")])
```

```
            onset  duration  value           trial_type
97       8.486912      10.0    1.0   BallSqueezing/Left
98     161.021952      10.0    1.0   BallSqueezing/Left
..            …         …     …                    …
128   1805.418496      10.0    1.0  BallSqueezing/Right
129   1931.116544      10.0    1.0  BallSqueezing/Right

[33 rows x 4 columns]
```

Selecting all motor tasks with the left hand:

[10]:
```python
with pd.option_context("display.max_rows", 5):
    display(rec.stim[rec.stim.trial_type.str.endswith("Left")])
```

```
            onset  duration  value          trial_type
65      99.549184      10.0    1.0       FTapping/Left
66     129.597440      10.0    1.0       FTapping/Left
..            …         …     …                   …
112   1962.541056      10.0    1.0  BallSqueezing/Left
113   1994.194944      10.0    1.0  BallSqueezing/Left

[33 rows x 4 columns]
```

Time Series

The 'amp' time series is represented as a `xarray.DataArray` with dimensions 'channel', 'wavelength' and 'time'. Three coordinate arrays are linked to the 'channel' dimension, specifying for each channel a string label as well as the string label and the corresponding source and detector labels (e.g. the first channel 'S1D1' is between source 'S1' and detector 'D1'). Coordinates of the 'wavelength' dimension indicate that this CW-fNIRS measurement was done at 760 and 850 nm. The 'time' dimensions has timestamps and an absolute sample counter as coordinates.

The `DataArray` is quantified in units of Volts.

[11]:
```
rec["amp"]
```

[11]:
```
<xarray.DataArray (channel: 100, wavelength: 2, time: 8794)> Size: 14MB
[V] 0.0874 0.08735 0.08819 0.08887 0.0879 … 0.09108 0.09037 0.09043 0.08999
Coordinates:
  * time        (time) float64 70kB 0.0 0.2294 0.4588 … 2.017e+03 2.017e+03
    samples     (time) int64 70kB 0 1 2 3 4 5 … 8788 8789 8790 8791 8792 8793
  * channel     (channel) object 800B 'S1D1' 'S1D2' 'S1D4' … 'S14D31' 'S14D32'
    source      (channel) object 800B 'S1' 'S1' 'S1' 'S1' … 'S14' 'S14' 'S14'
    detector    (channel) object 800B 'D1' 'D2' 'D4' 'D5' … 'D29' 'D31' 'D32'
  * wavelength  (wavelength) float64 16B 760.0 850.0
Attributes:
    data_type_group:  unprocessed raw
```

Montage

As described in the first tutorial, the probe geometry is stored in `rec.geo3d` as a `DataArray` of type `cedalion.typing.LabeledPoints`.

[12]:
```
rec.geo3d
```

[12]:
```
<xarray.DataArray (label: 346, digitized: 3)> Size: 8kB
[mm] -77.82 15.68 23.17 -61.91 21.23 56.49 … 14.23 -38.28 81.95 -0.678 -37.03
Coordinates:
    type     (label) object 3kB PointType.SOURCE … PointType.LANDMARK
  * label    (label) <U6 8kB 'S1' 'S2' 'S3' 'S4' … 'FFT10h' 'FT10h' 'FTT10h'
Dimensions without coordinates: digitized
```

[13]:
```
cedalion.vis.anatomy.plot_montage3D(rec["amp"], rec.geo3d)
```

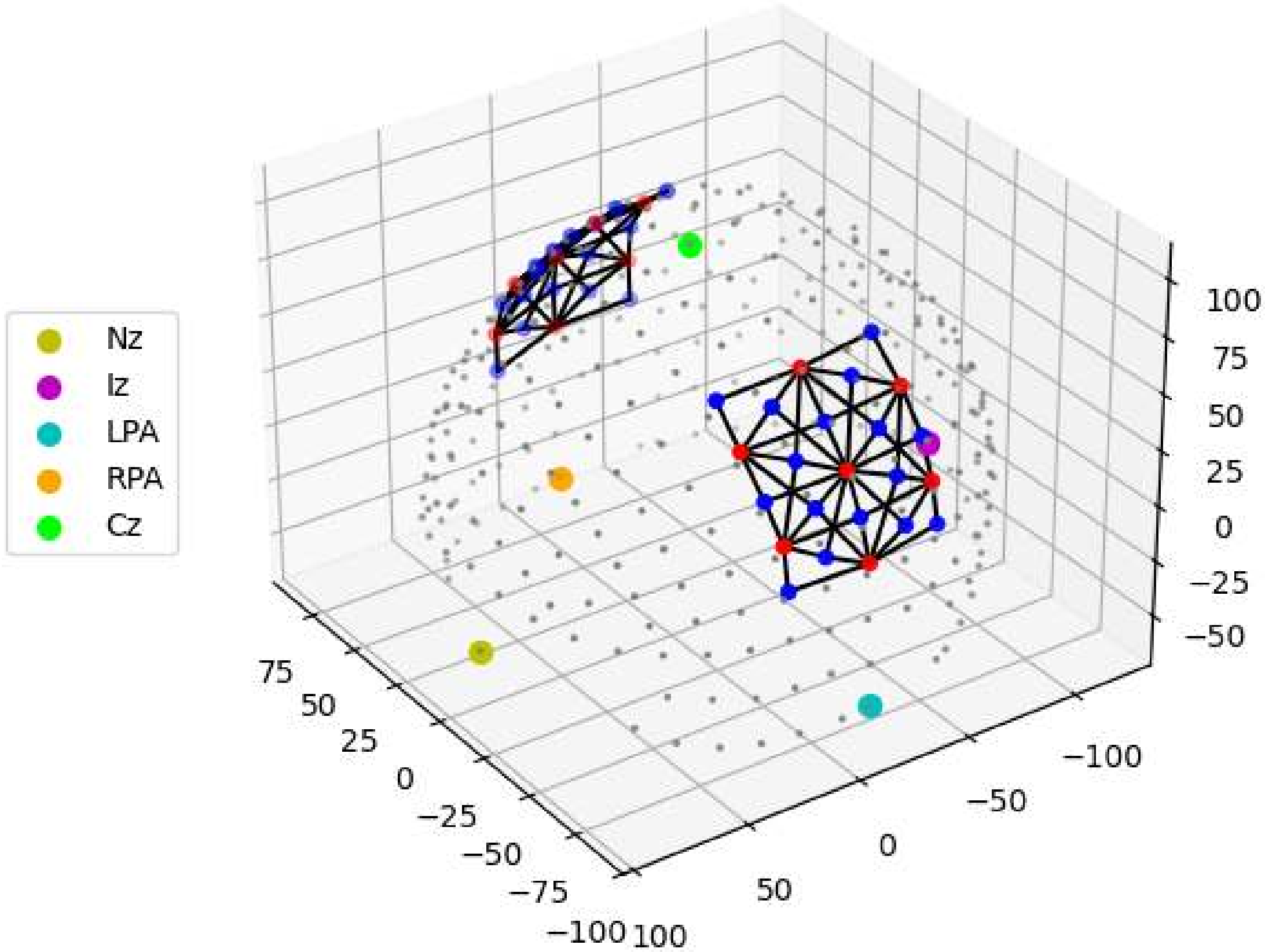

Using functions of `matplotlib` time trace for a single channel can be plotted. Note, how the time series `amp` is indexed by label to select channel and wavelengths.

The package `cedalion.vis.blocks` (imported as `vbx`) provides visualizations building blocks, such as adding stimulus markers to a plot.

[14]:
```
# example time trace
amp = rec["amp"]
ch = "S12D25"
f, ax = p.subplots(1, 1, figsize=(12, 3))
ax.set_prop_cycle("color", cedalion.vis.colors.COLORBREWER_Q8)
ax.plot(amp.time, amp.sel(channel=ch, wavelength=760), label="amp. 760 nm")
ax.plot(amp.time, amp.sel(channel=ch, wavelength=850), label="amp. 850 nm")
vbx.plot_stim_markers(ax, rec.stim, y=1)
ax.set_xlabel("time / s")
ax.set_ylabel("amplitude / V")
ax.set_xlim(0, 150)
ax.legend(loc="upper right")
ax.set_title(ch);
```

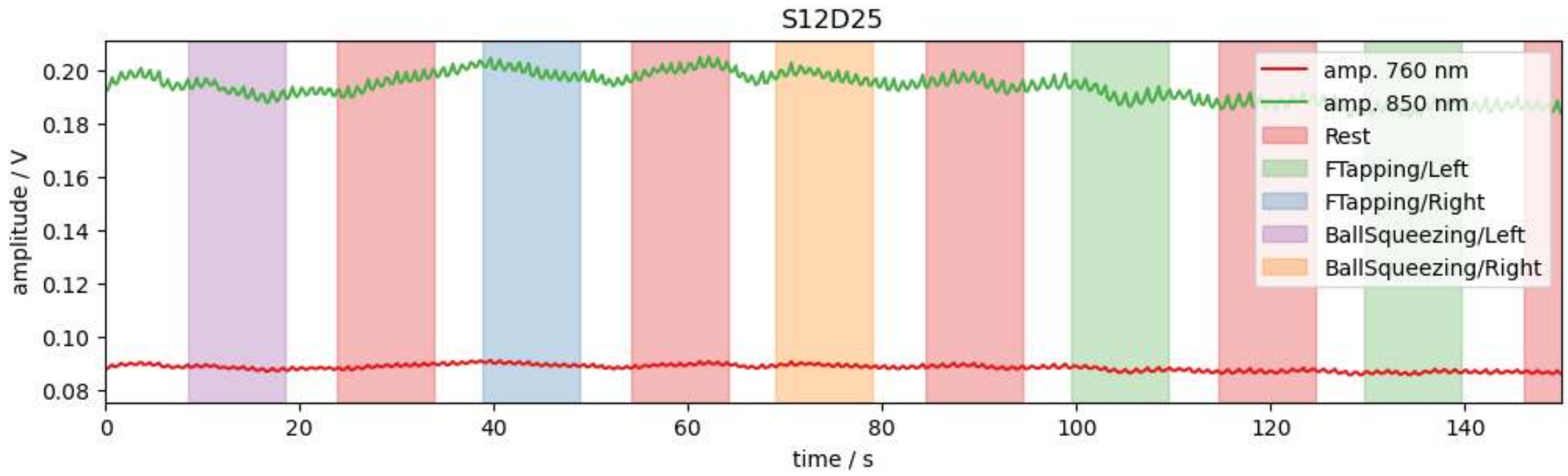

Quality Metrics : SCI & PSP

To assess the signal quality the Scalp Coupling Index (SCI) and Peak Spectral Power (PSP) are calculated with functions from `cedalion.sigproc.quality`. Both metrics are computed in 10-second sliding windows. Each metric a configurable threshold. Values exceeding the threshold indicate good signal quality. The functions `sci` and `psp` each return two `DataArrays`: one with the metric values, and one with a boolean mask indicating where the threshold is exceeded.

Since the metrics are calculated for each time window the time axis changed.

[15]:
```
sci_threshold = 0.75
window_length = 10 * units.s
sci, sci_mask = quality.sci(rec["amp"], window_length, sci_threshold)

psp_threshold = 0.03
psp, psp_mask = quality.psp(rec["amp"], window_length, psp_threshold)


display(sci.rename("sci"))
display(sci_mask.rename("sci_mask"))
```

```
<xarray.DataArray 'sci' (channel: 100, time: 200)> Size: 160kB
0.9949 0.9949 0.9775 0.9958 0.9914 0.9941 … 0.9369 0.9498 0.9628 0.9718 0.9545
Coordinates:
  * time      (time) float64 2kB 0.0 10.09 20.19 … 1.998e+03 2.008e+03
    samples   (time) int64 2kB 0 44 88 132 176 220 … 8580 8624 8668 8712 8756
  * channel   (channel) object 800B 'S1D1' 'S1D2' 'S1D4' … 'S14D31' 'S14D32'
    source    (channel) object 800B 'S1' 'S1' 'S1' 'S1' … 'S14' 'S14' 'S14'
    detector  (channel) object 800B 'D1' 'D2' 'D4' 'D5' … 'D29' 'D31' 'D32'
```

```
<xarray.DataArray 'sci_mask' (channel: 100, time: 200)> Size: 20kB
True True True True True True True True … True True True True True True True
Coordinates:
  * time      (time) float64 2kB 0.0 10.09 20.19 … 1.998e+03 2.008e+03
    samples   (time) int64 2kB 0 44 88 132 176 220 … 8580 8624 8668 8712 8756
  * channel   (channel) object 800B 'S1D1' 'S1D2' 'S1D4' … 'S14D31' 'S14D32'
    source    (channel) object 800B 'S1' 'S1' 'S1' 'S1' … 'S14' 'S14' 'S14'
    detector  (channel) object 800B 'D1' 'D2' 'D4' 'D5' … 'D29' 'D31' 'D32'
```

Visualize the metrics and quality masks:

[16]:
```
# define three colomaps: redish below a threshold, blueish above
sci_norm, sci_cmap = colors.threshold_cmap("sci_cmap", 0.0, 1.0, sci_threshold)
psp_norm, psp_cmap = colors.threshold_cmap("psp_cmap", 0.0, 0.30, psp_threshold)


def plot_sci(sci):
    # plot the heatmap
    f, ax = p.subplots(1, 1, figsize=(12, 10))

    m = ax.pcolormesh(sci.time, np.arange(len(sci.channel)), sci, shading="nearest",␣
 ↪cmap=sci_cmap, norm=sci_norm)
    cb = p.colorbar(m, ax=ax)
    cb.set_label("SCI")
    ax.set_xlabel("time / s")
    p.tight_layout()
    ax.yaxis.set_ticks(np.arange(len(sci.channel)))
    ax.yaxis.set_ticklabels(sci.channel.values, fontsize=7)


def plot_psp(psp):
    f, ax = p.subplots(1, 1, figsize=(12, 10))

    m = ax.pcolormesh(psp.time, np.arange(len(psp.channel)), psp, shading="nearest",␣
 ↪cmap=psp_cmap, norm=psp_norm)
    cb = p.colorbar(m, ax=ax)
    cb.set_label("PSP")
    ax.set_xlabel("time / s")
    p.tight_layout()
    ax.yaxis.set_ticks(np.arange(len(psp.channel)))
    ax.yaxis.set_ticklabels(psp.channel.values, fontsize=7)
```

[17]:
```
plot_sci(sci)
plot_quality_mask(sci > sci_threshold, f"SCI > {sci_threshold}")
plot_psp(psp)
plot_quality_mask(psp > psp_threshold, f"PSP > {psp_threshold}")
```

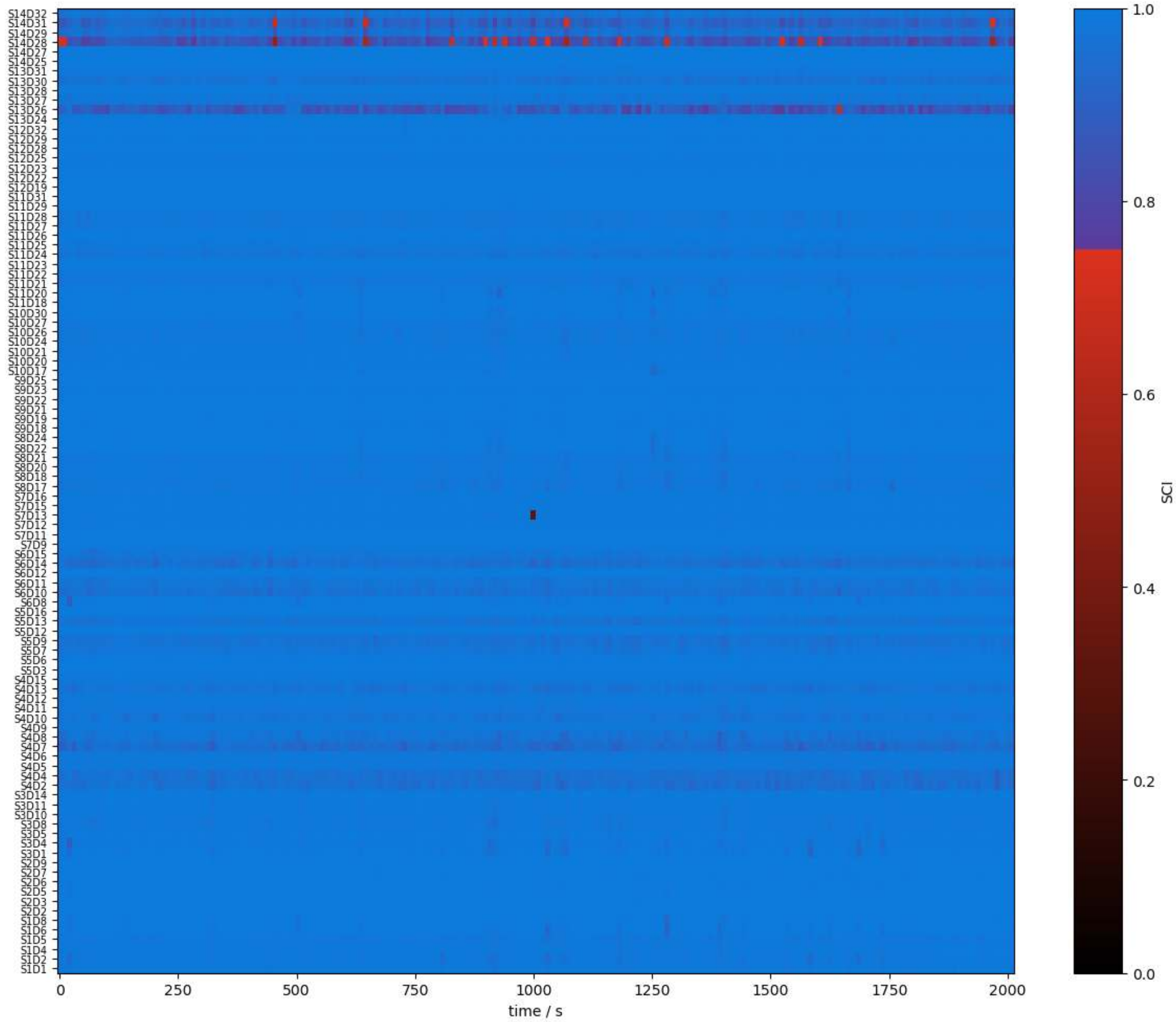
time / s
0
250
500
750
1000
1250
1500
1750
2000
SCI
0.0
0.2
0.4
0.6
0.8
1.0

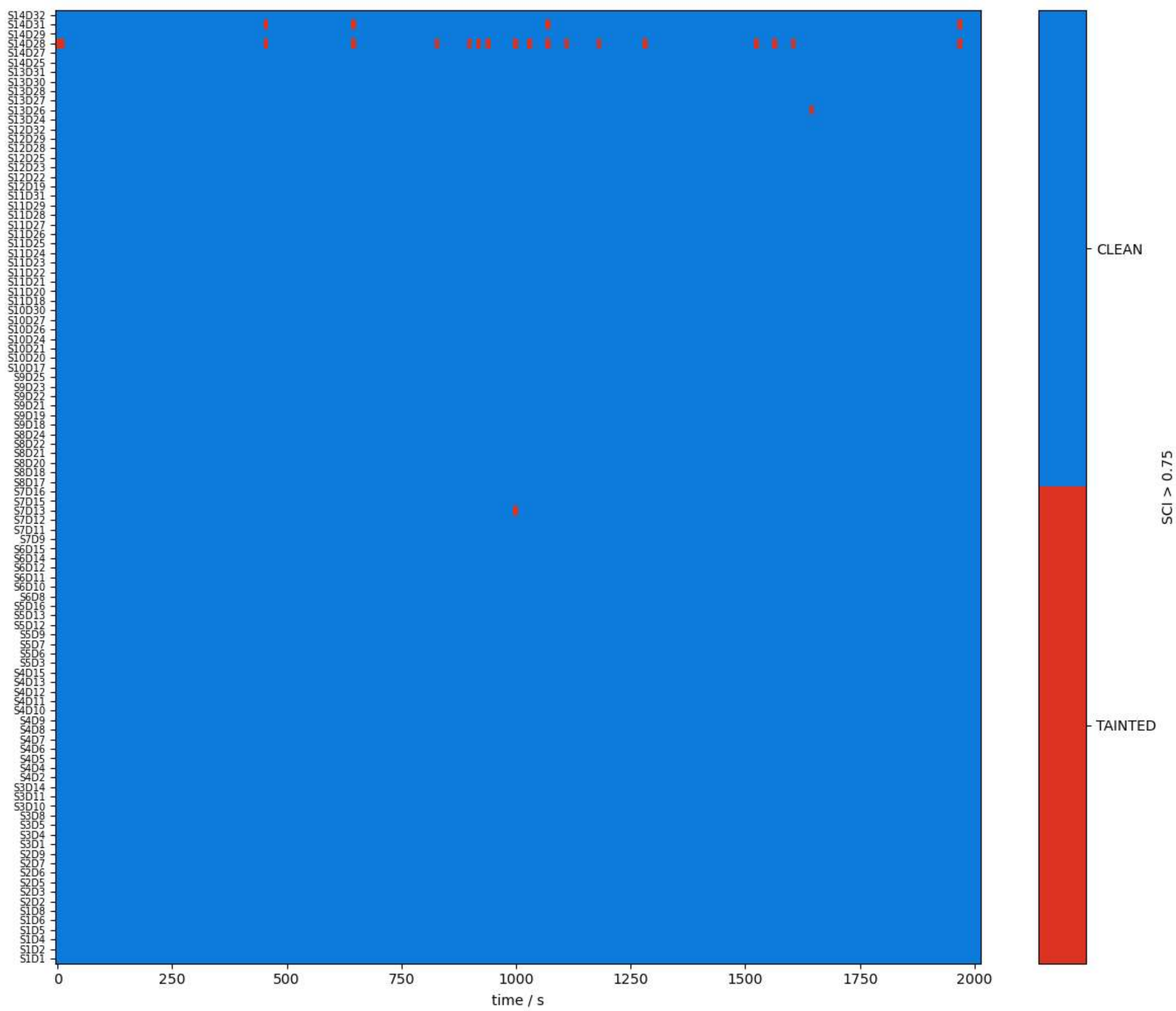
S14D32
S14D31
S14D29
S14D28
S14D27
S14D25
S13D31
S13D30
S13D28
S13D27
S13D26
S13D24
S12D32
S12D29
S12D28
S12D25
S12D23
S12D22
S12D19
S11D31
S11D29
S11D28
S11D27
S11D26
S11D25
S11D24
S11D23
S11D22
S11D21
S11D20
S11D18
S10D30
S10D27
S10D26
S10D24
S10D21
S10D20
S10D17
S9D25
S9D23
S9D22
S9D21
S9D19
S9D18
S8D24
S8D22
S8D21
S8D20
S8D18
S8D17
S7D16
S7D15
S7D13
S7D12
S7D11
S7D9
S6D15
S6D14
S6D12
S6D11
S6D10
S6D8
S5D16
S5D13
S5D12
S5D9
S5D7
S5D6
S5D3
S4D15
S4D13
S4D12
S4D11
S4D10
S4D9
S4D8
S4D7
S4D6
S4D5
S4D4
S4D2
S3D14
S3D11
S3D10
S3D8
S3D5
S3D4
S3D1
S2D9
S2D7
S2D6
S2D5
S2D3
S2D2
S1D8
S1D6
S1D5
S1D4
S1D2
S1D1
0
250
500
750
1000
1250
1500
1750
2000
time / s
CLEAN
TAINTED
SCI > 0.75

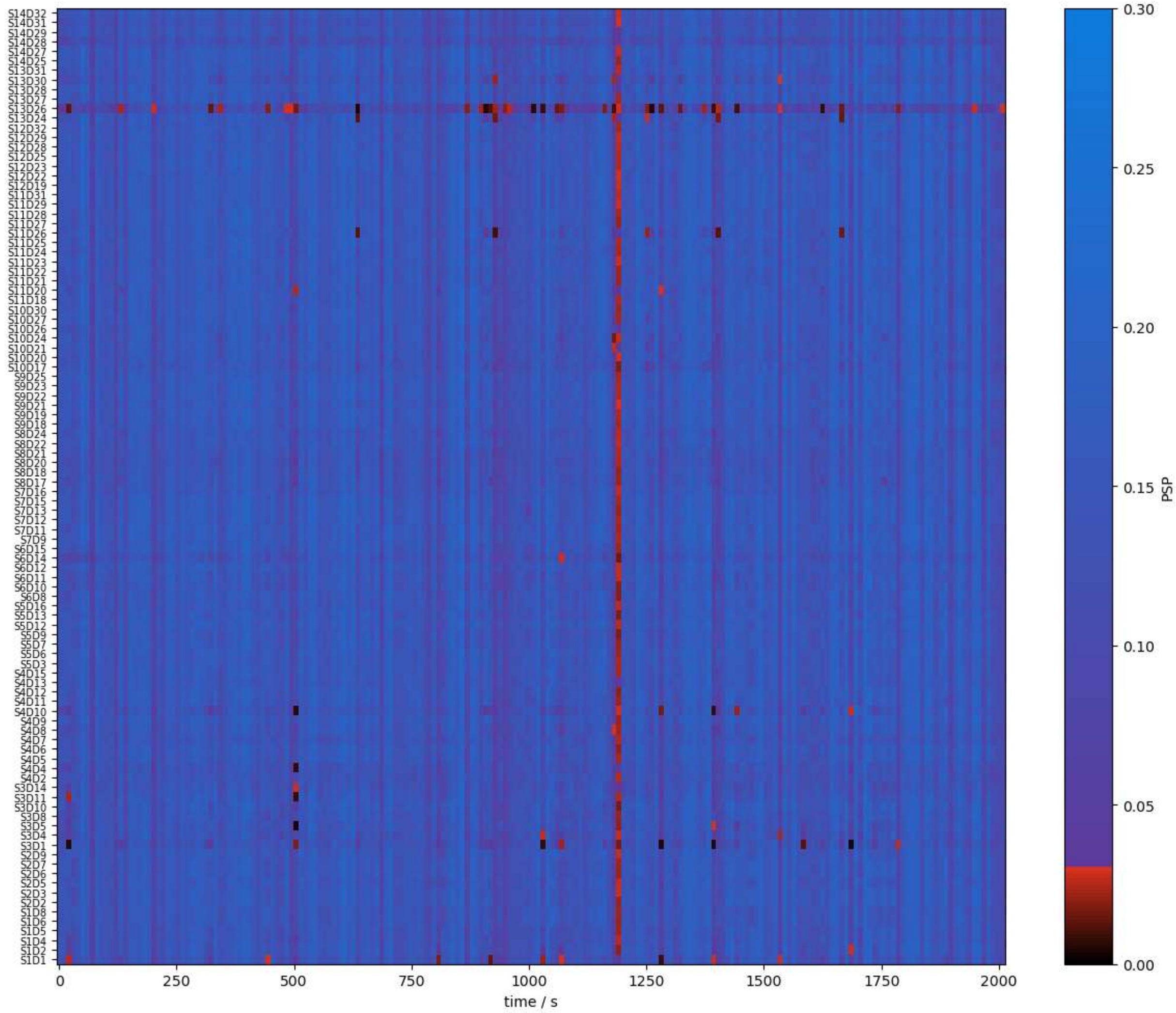
time / s
PSP

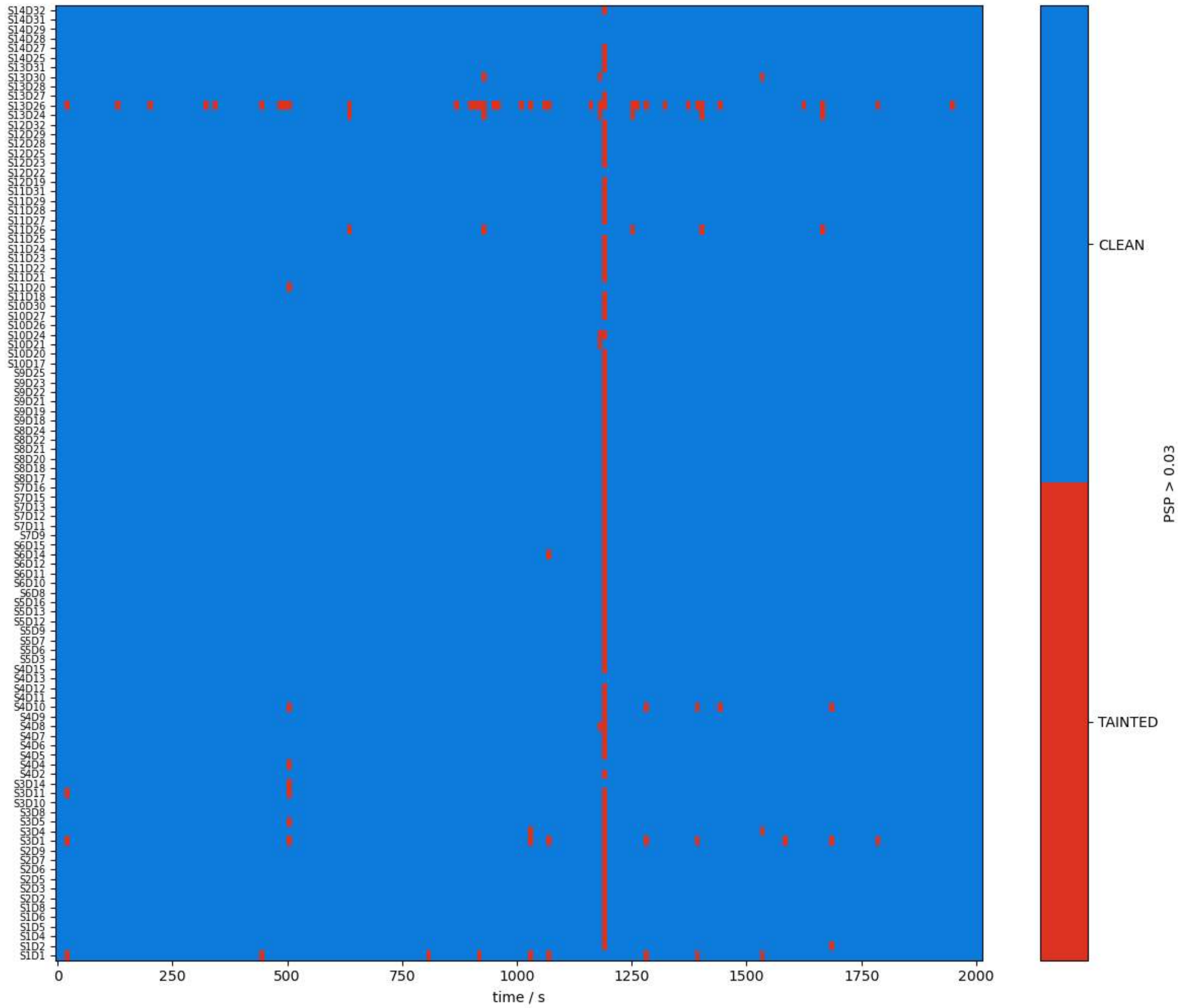

A window shall be considered clean only if both SCI and PSP exceed their respective thresholds. The user can combine the two boolean masks with a logical **and**, then compute the fraction of time windows where both conditions are true.

[18]:
```
combined_mask = sci_mask & psp_mask

display(combined_mask)
plot_quality_mask(combined_mask, "combined_mask")
```

```
<xarray.DataArray (channel: 100, time: 200)> Size: 20kB
True True False True True True True True … True True True True True True True
Coordinates:
  * time      (time) float64 2kB 0.0 10.09 20.19 … 1.998e+03 2.008e+03
    samples   (time) int64 2kB 0 44 88 132 176 220 … 8580 8624 8668 8712 8756
  * channel   (channel) object 800B 'S1D1' 'S1D2' 'S1D4' … 'S14D31' 'S14D32'
    source    (channel) object 800B 'S1' 'S1' 'S1' 'S1' … 'S14' 'S14' 'S14'
    detector  (channel) object 800B 'D1' 'D2' 'D4' 'D5' … 'D29' 'D31' 'D32'
```

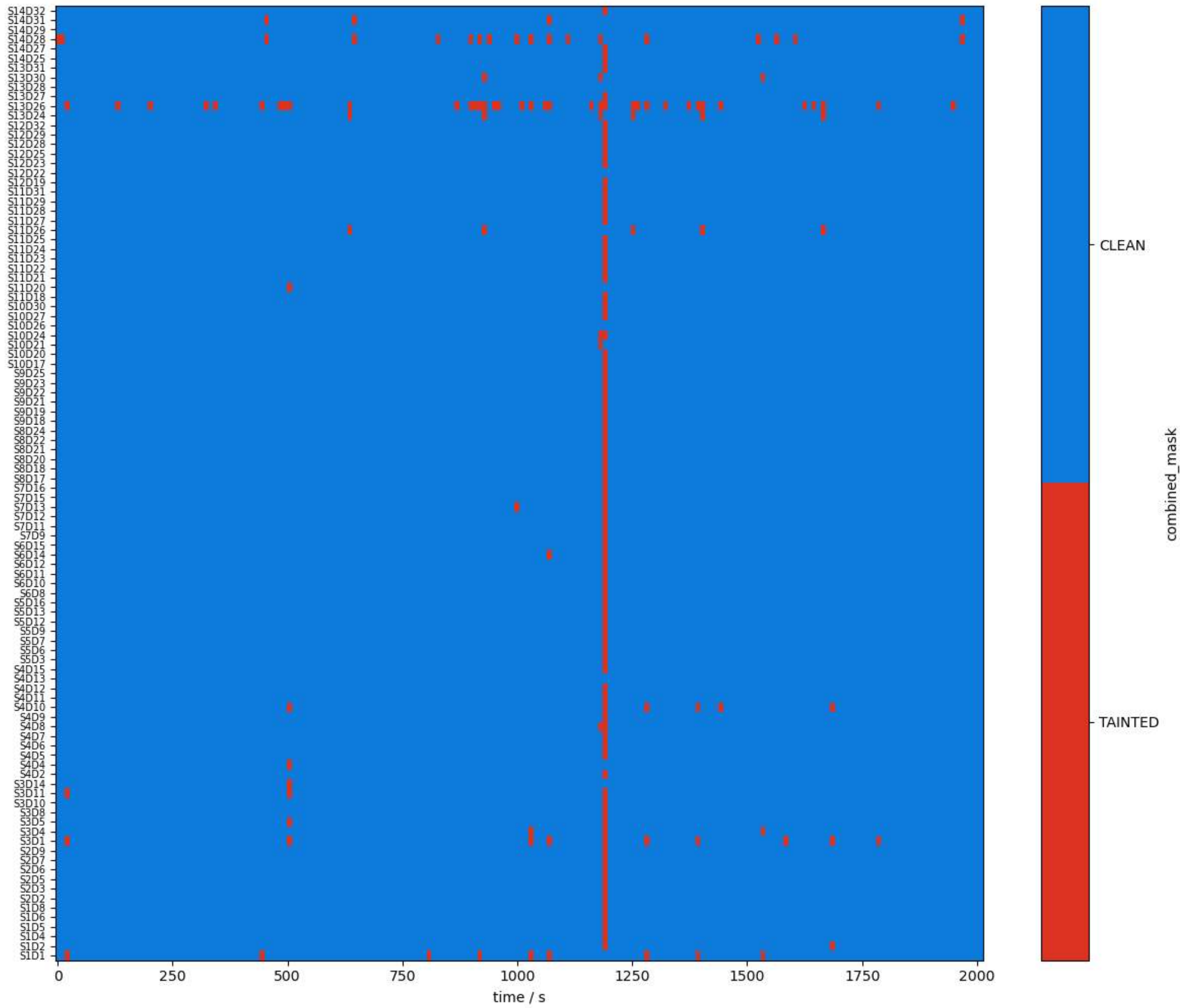

The next cell calculates the percentage of clean time windows per channel. Afterwards, two channels are identified that are clean in fewer than 95% of the time windows.

[19]:
```python
perc_time_clean = combined_mask.sum(dim="time") / len(sci.time)

display(perc_time_clean)

print("Channels clean less than 95% of the recording:")
display(perc_time_clean[perc_time_clean < 0.95])
```

```
<xarray.DataArray (channel: 100)> Size: 800B
0.955 0.99 0.995 0.995 0.995 0.995 0.995 … 0.995 0.995 0.91 1.0 0.98 0.995
Coordinates:
  * channel   (channel) object 800B 'S1D1' 'S1D2' 'S1D4' … 'S14D31' 'S14D32'
    source    (channel) object 800B 'S1' 'S1' 'S1' 'S1' … 'S14' 'S14' 'S14'
    detector  (channel) object 800B 'D1' 'D2' 'D4' 'D5' … 'D29' 'D31' 'D32'

Channels clean less than 95% of the recording:

<xarray.DataArray (channel: 2)> Size: 16B
0.815 0.91
```

```
Coordinates:
  * channel   (channel) object 16B 'S13D26' 'S14D28'
    source    (channel) object 16B 'S13' 'S14'
    detector  (channel) object 16B 'D26' 'D28'
```

Visualize the percentage of clean times with a scalp plot:

[20]:
```
f, ax = p.subplots(1,1,figsize=(6.5,6.5))

cedalion.vis.anatomy.scalp_plot(
    rec["amp"],
    rec.geo3d,
    perc_time_clean,
    ax,
    cmap="RdYlGn",
    vmin=0.7,
    vmax=1,
    title=None,
    cb_label="Percentage of clean time",
    channel_lw=2,
    optode_labels=True
)
f.tight_layout()
```

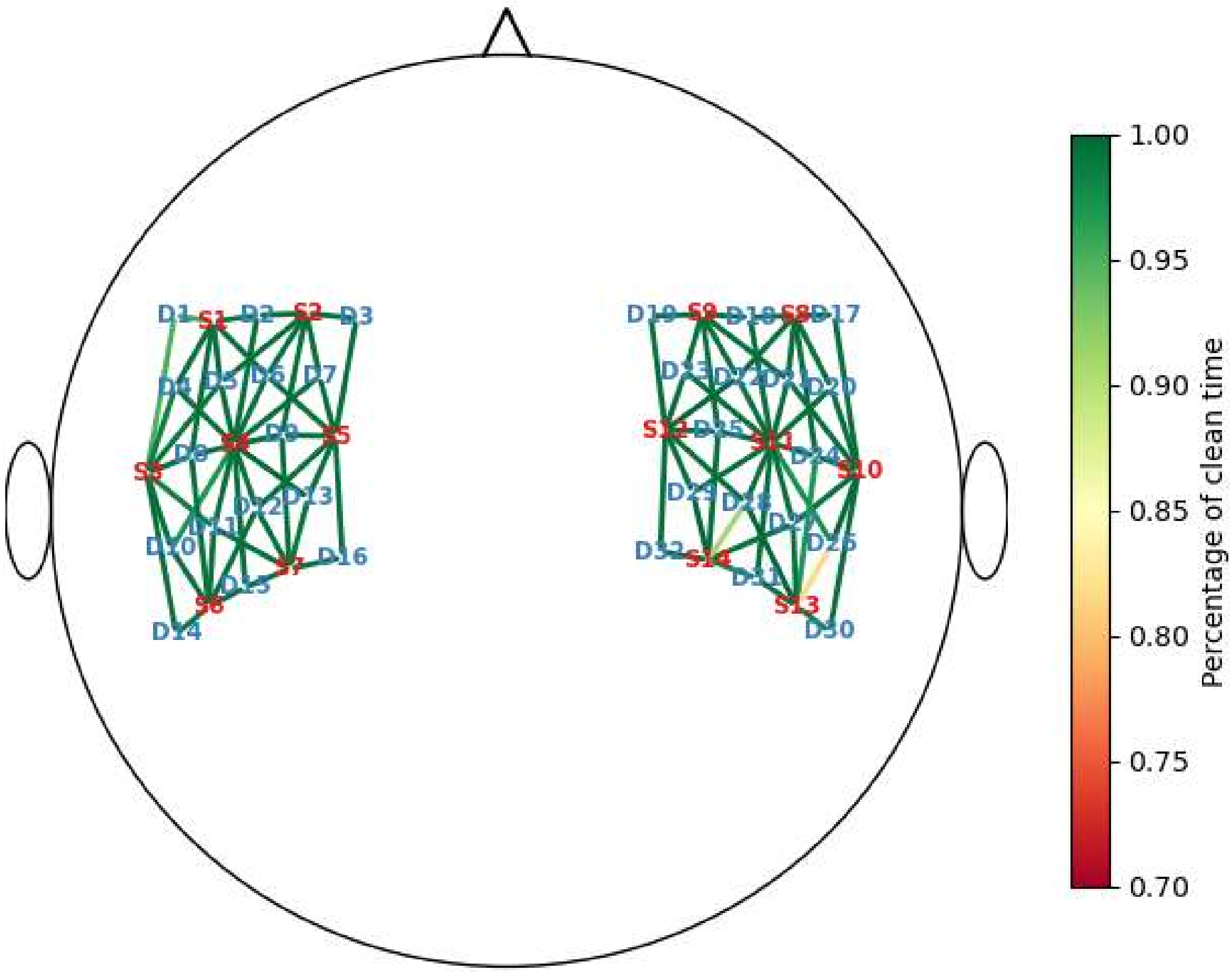

Correct Motion Artifacts

Using the function `cedalion.nirs.cw.int2od` the raw amplitudes are converted to optical densities. Two motion-artifact correction methods are then applied.

- First, Temporal Derivative Distribution Repair (TDDR) is used to repair unusually large jumps in the time series.
- Second, a wavelet-based motion correction is applied.

The correction algorithms operate on optical densities. After correction, the corresponding corrected amplitudes are derived.

The modified time series are stored under different names in the `Recording` container.

[21]:
```
rec["od"] = cedalion.nirs.cw.int2od(rec["amp"])
rec["od_tddr"] = motion.tddr(rec["od"])
```

```python
rec["od_wavelet"] = motion.wavelet(rec["od_tddr"])
rec["amp_corrected"] = cedalion.nirs.cw.od2int(
    rec["od_wavelet"], rec["amp"].mean("time")
)
```

Recalculate the SCI and PSP metrics on the corrected amplitudes and visualize the combined masks before and after correction.

[22]:
```python
# recalculate sci & psp on cleaned data
sci_corr, sci_corr_mask = quality.sci(
    rec["amp_corrected"], window_length, sci_threshold
)
psp_corr, psp_corr_mask = quality.psp(
    rec["amp_corrected"], window_length, psp_threshold
)
combined_corr_mask = sci_corr_mask & psp_corr_mask
```

[23]:
```python
plot_quality_mask(combined_mask, "combined mask")
plot_quality_mask(combined_corr_mask, "combined corrected mask")
```

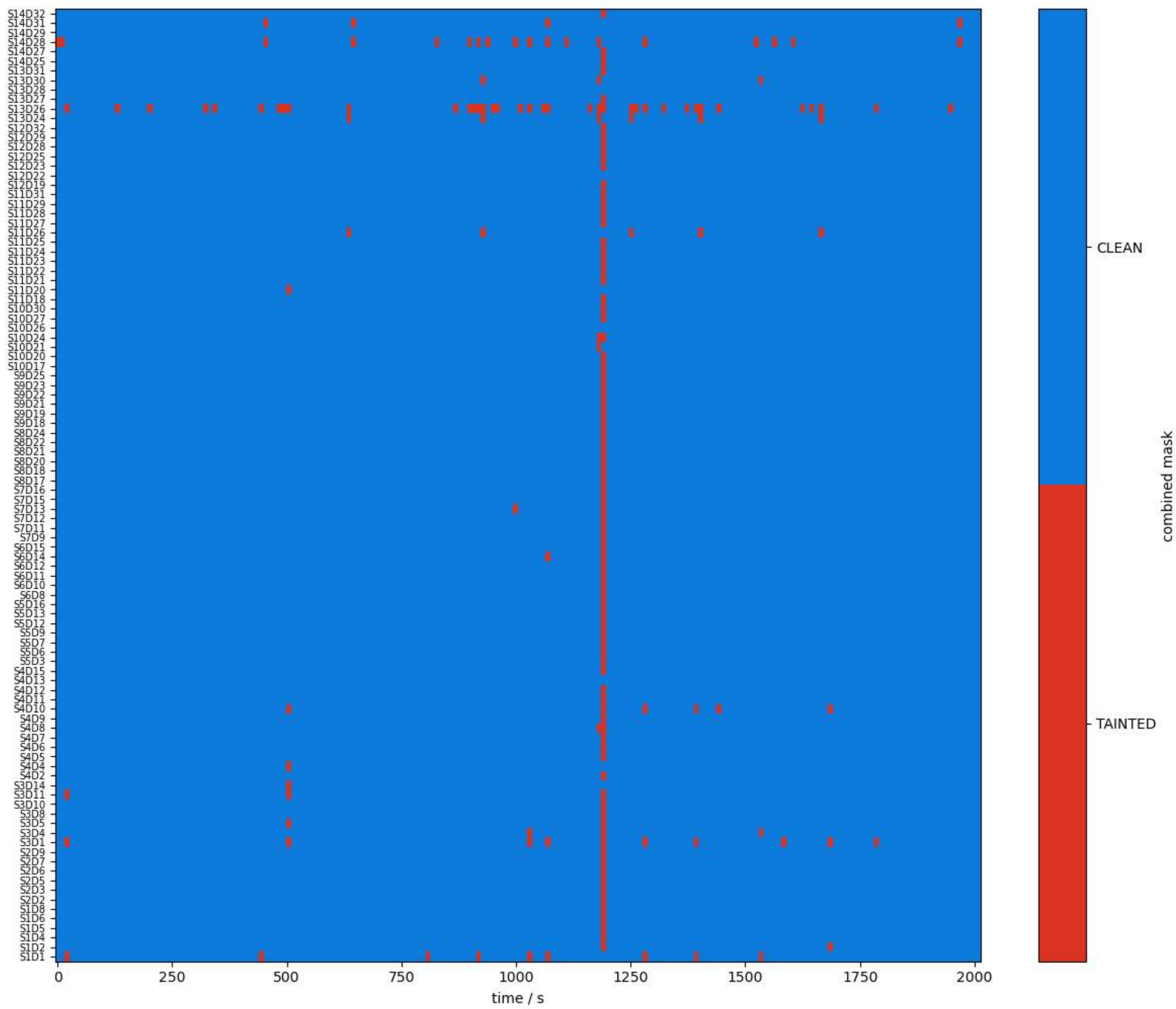

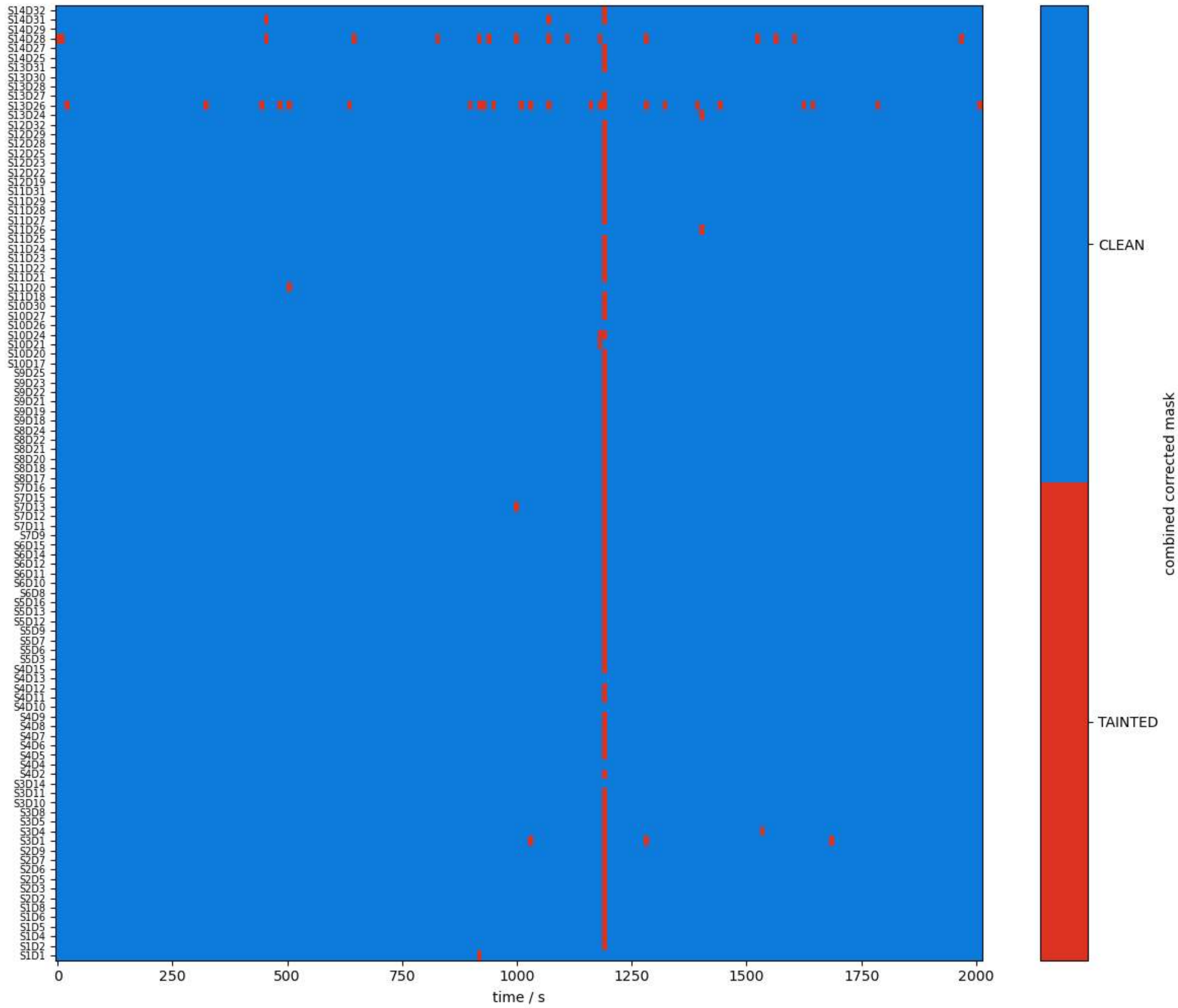

Compare masks before and after motion artifact correction

Using logical operations on the quality masks, the next cell identifies the time windows affected by the correction algorithms.

[24]:
```
changed_windows = (combined_mask == quality.TAINTED) & (combined_corr_mask == quality.
    ↪CLEAN)

plot_quality_mask(
    changed_windows,
    "mask of time windows cleaned by motion correction",
    bool_labels=["unchanged", "improved"],
)

changed_windows = (combined_mask == quality.CLEAN) & (combined_corr_mask == quality.
    ↪TAINTED)

plot_quality_mask(
    changed_windows,
```

```
    "mask of time windows corrupted by motion correction",
    bool_labels=["unchanged", "worsened"],
)
```

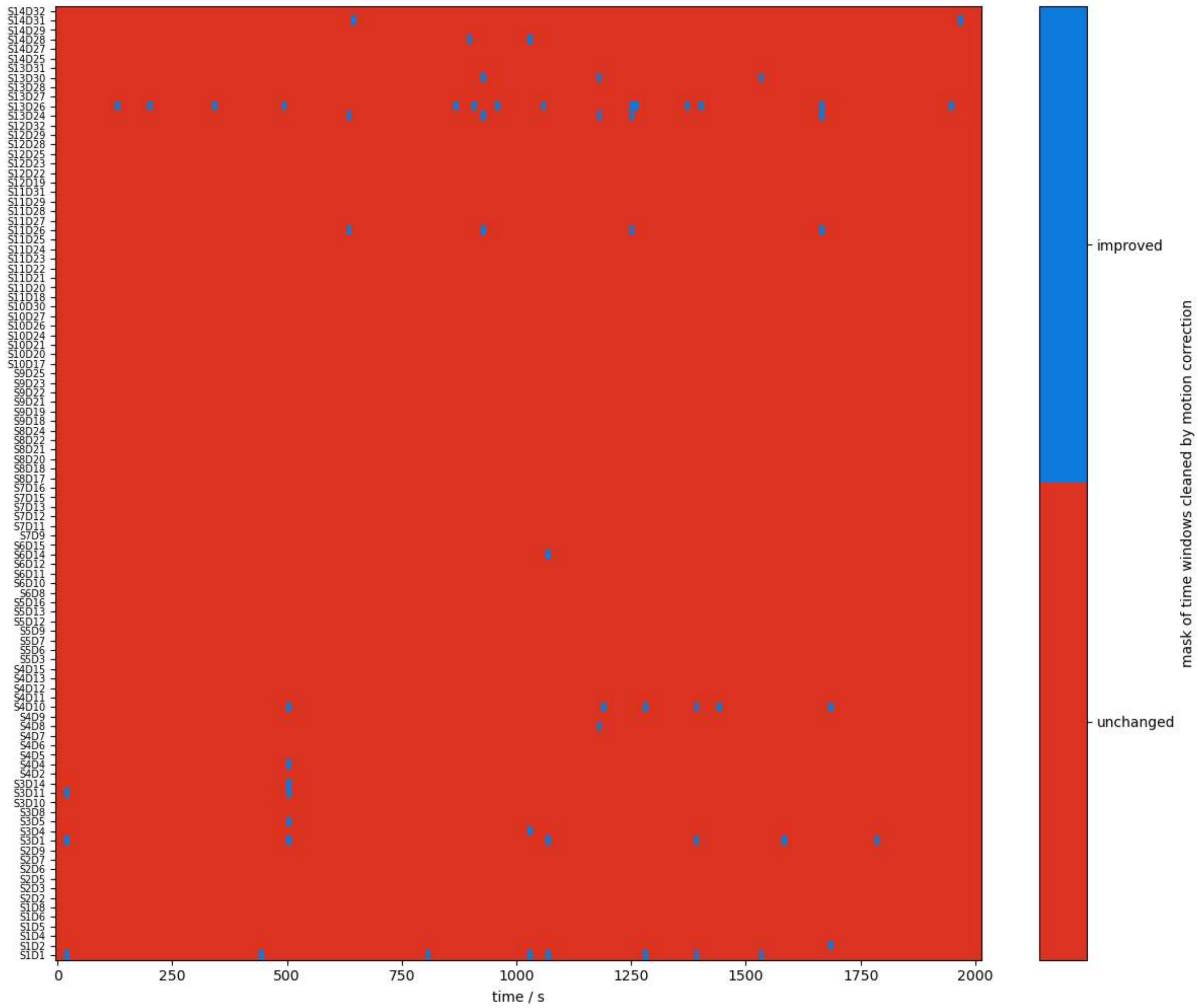

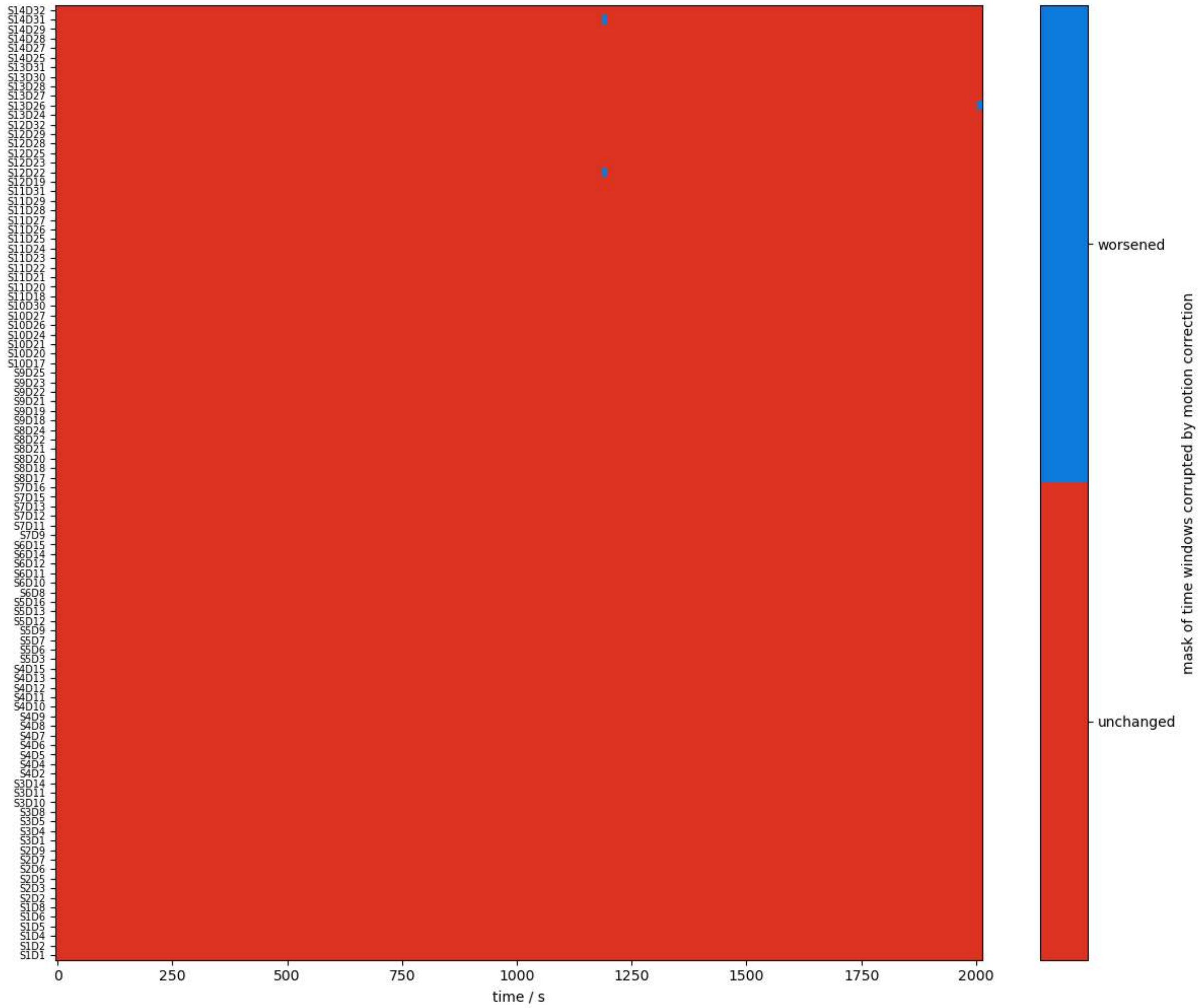

Recalculate and visualize the percentage of clean time:

[25]:
```python
perc_time_clean_corr = combined_corr_mask.sum(dim="time") / len(sci.time)

f, ax = p.subplots(1,2,figsize=(14,6.5))

cedalion.vis.anatomy.scalp_plot(
    rec["amp"],
    rec.geo3d,
    perc_time_clean,
    ax[0],
    cmap="RdYlGn",
    vmin=0.80,
    vmax=1,
    title="before correction",
    cb_label="Percentage of clean time",
    channel_lw=2,
    optode_labels=True
)
```

```
cedalion.vis.anatomy.scalp_plot(
    rec["amp"],
    rec.geo3d,
    perc_time_clean_corr,
    ax[1],
    cmap="RdYlGn",
    vmin=0.80,
    vmax=1,
    title="after correction",
    cb_label="Percentage of clean time",
    channel_lw=2,
    optode_labels=True
)
f.tight_layout()
```

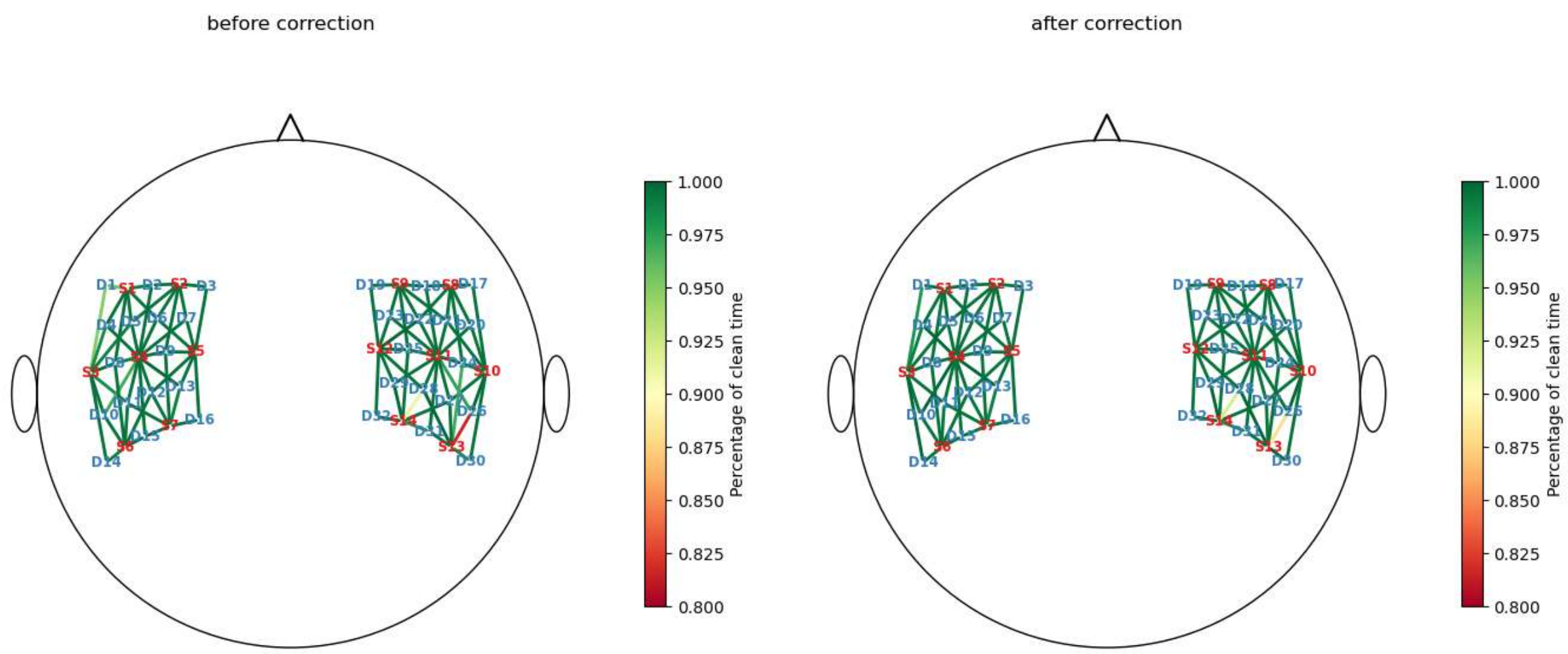

Global Variance of the Temporal Derivative (GVTD)

The GVTD metric allows identifying global bad time segments. Here, it is calculated for the original and corrected amplitudes.

[26]:
```
gvtd, gvtd_mask = quality.gvtd(rec["amp"])
gvtd_corr, gvtd_prr_mask = quality.gvtd(rec["amp_corrected"])
```

The 10 segments with highest GVTD values are selected:

[27]:
```
top10_bad_segments = sorted(
    [seg for seg in quality.mask_to_segments(combined_mask.all("channel"))],
    key=lambda t: gvtd.sel(time=slice(t[0], t[1])).max(),
    reverse=True,
)[:10]
```

The following plot shows the GVTD metric before and after corrections. The 10 selected segments are highlighted in red. The combined masks are further reduced to indicate, if all channels at a given time are clean.

The impact of the correction methods is evident in the reduced number of spikes in the GVTD trace and the improvements seen in the quality mask. Time windows that previously did not satisfy the all-channels-clean criteria meet it after correction.

[28]:
```
f,ax = p.subplots(4,1,figsize=(16,6), sharex=True)
ax[0].plot(gvtd.time, gvtd)
ax[1].plot(combined_mask.time, combined_mask.all("channel"))
ax[2].plot(gvtd_corr.time, gvtd_corr)
ax[3].plot(combined_corr_mask.time, combined_corr_mask.all("channel"))
ax[0].set_ylim(0, 0.02)
ax[2].set_ylim(0, 0.02)
ax[0].set_ylabel("GVTD")
ax[2].set_ylabel("GVTD")
ax[1].set_ylabel("all channels clean\n (before)")
ax[3].set_ylabel("all channels clean\n (after)")
ax[3].set_xlabel("time / s")

for i in range(4):
    vbx.plot_segments(ax[i], top10_bad_segments)
```

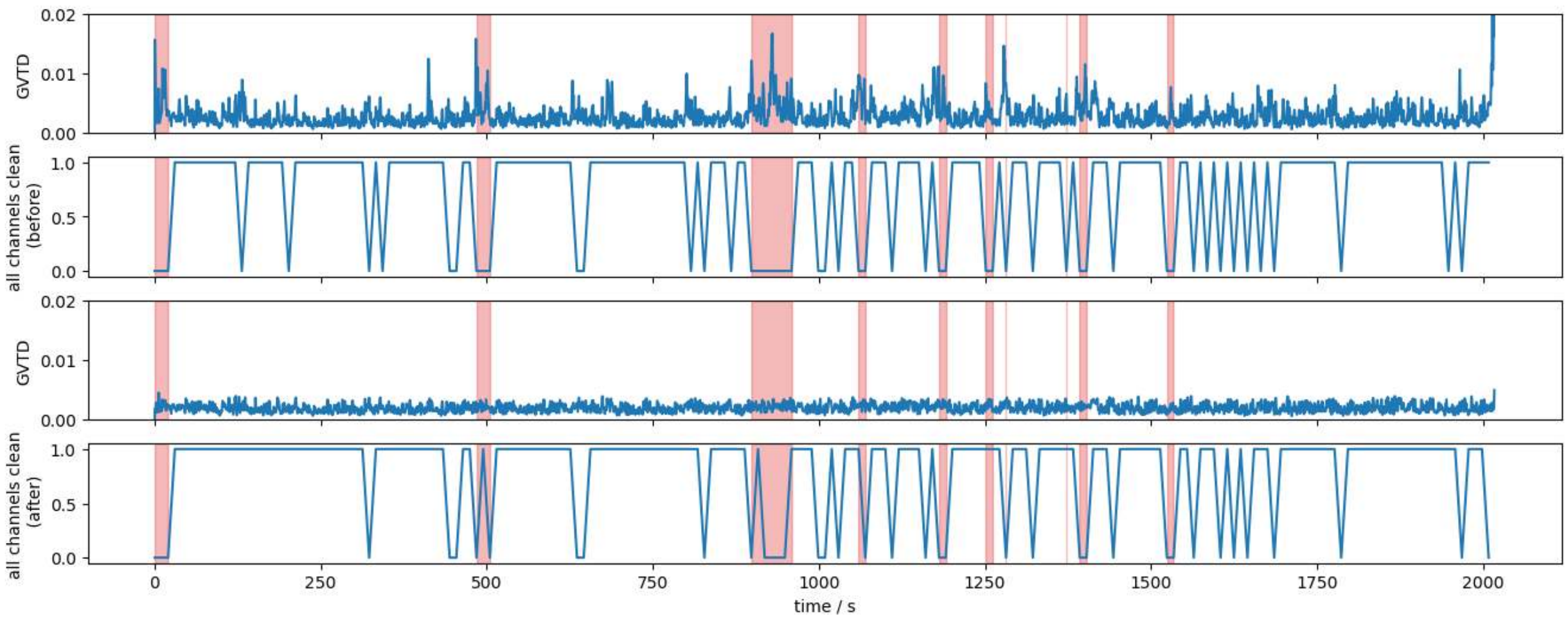

Visualize motion correction in selected segments

To illustrate the effect of TDDR and wavelet-based motion correction on the time series, the following cell plots the amplitudes for the ten selected segments in two channels before and after correction.

[29]:
```
example_channels = ["S4D10", "S13D26"]

f, ax = p.subplots(5,4, figsize=(16,16), sharex=False)
ax = ax.T.flatten()
padding = 15
i = 0
for ch in example_channels:
    for (start, end) in top10_bad_segments:
        ax[i].set_prop_cycle(color=["#e41a1c", "#ff7f00", "#377eb8", "#984ea3"])
        for wl in rec["od"].wavelength.values:
```

```
            sel = rec["od"].sel(time=slice(start-padding, end+padding), channel=ch,␣
↪wavelength=wl)
            ax[i].plot(sel.time, sel, label=f"{wl:.0f} nm orig")
            sel = rec["od_wavelet"].sel(time=slice(start-padding, end+padding),␣
↪channel=ch, wavelength=wl)
            ax[i].plot(sel.time, sel, label=f"{wl:.0f} nm corr")
            ax[i].set_title(ch)
        ax[i].legend(ncol=2, loc="upper center")
        ylim = ax[i].get_ylim()
        ax[i].set_ylim(ylim[0], ylim[1]+0.25*(ylim[1]-ylim[0])) # make space for legend

        i += 1

p.tight_layout()
```

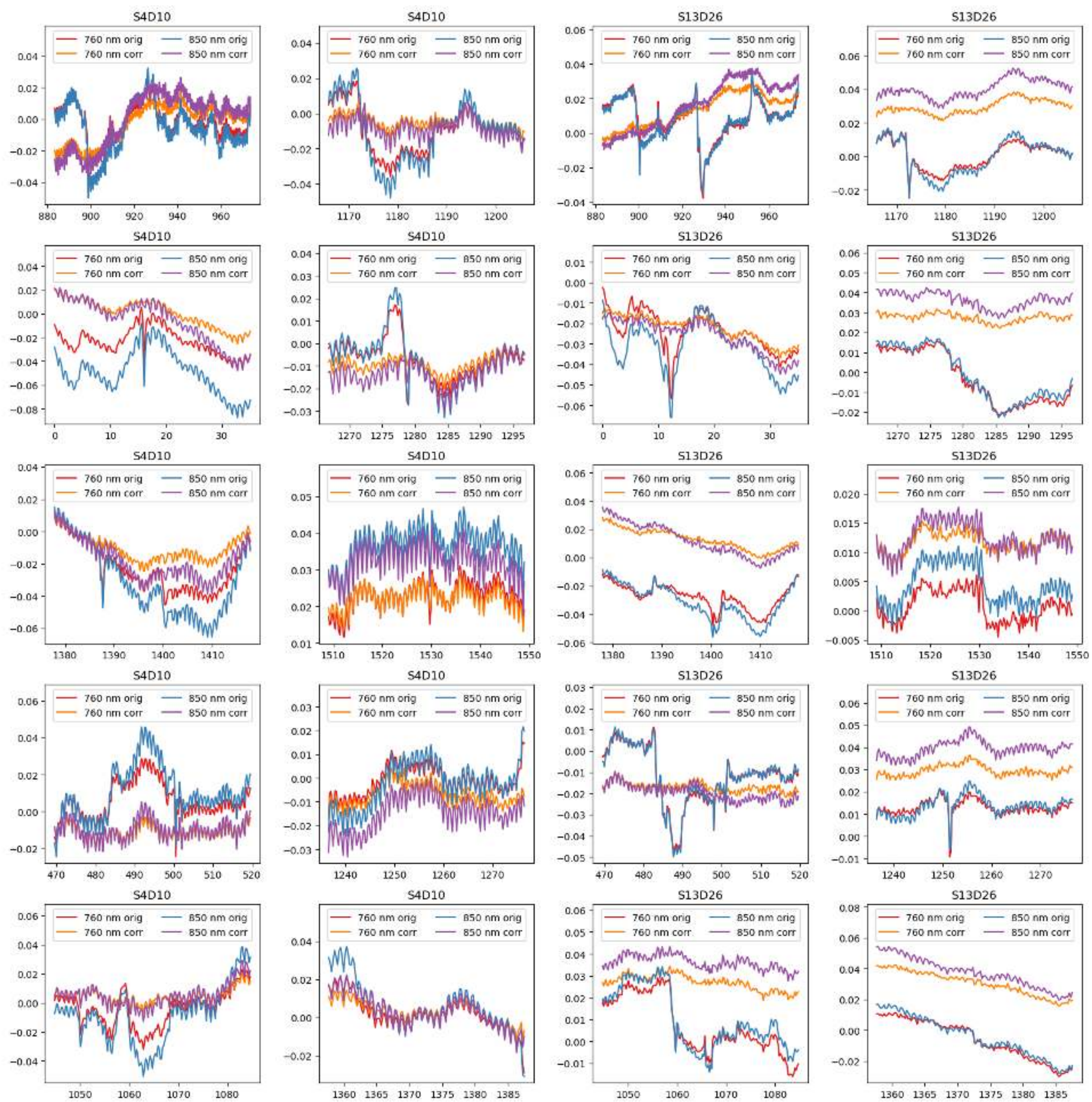

Final channel selection

Before correction, the channels "S13D26" and "S14D28" had the most unclean time windows. Although the correction methods recovered some of these windows, the fraction of clean windows for both channels still remains below 95%.

[30]:
```python
perc_time_clean_corr[perc_time_clean_corr < 0.95]
```

[30]:
```
<xarray.DataArray (channel: 2)> Size: 16B
0.88 0.92
Coordinates:
  * channel   (channel) object 16B 'S13D26' 'S14D28'
    source    (channel) object 16B 'S13' 'S14'
    detector  (channel) object 16B 'D26' 'D28'
```

To prune bad channels from time series the function `cedalion.sigproc.quality.prun_ch` is available. It takes a list of quality masks, combines them and discards channels from the time series.

[31]:
```python
signal_quality_selection_masks = [perc_time_clean >= .95]

rec["amp_pruned"], pruned_channels = quality.prune_ch(
    rec["amp"], signal_quality_selection_masks, "all"
)
display(rec["amp_pruned"])
display(pruned_channels)
```

```
<xarray.DataArray (channel: 98, wavelength: 2, time: 8794)> Size: 14MB
[V] 0.0874 0.08735 0.08819 0.08887 0.0879 … 0.09108 0.09037 0.09043 0.08999
Coordinates:
  * time        (time) float64 70kB 0.0 0.2294 0.4588 … 2.017e+03 2.017e+03
    samples     (time) int64 70kB 0 1 2 3 4 5 … 8788 8789 8790 8791 8792 8793
  * channel     (channel) object 784B 'S1D1' 'S1D2' 'S1D4' … 'S14D31' 'S14D32'
    source      (channel) object 784B 'S1' 'S1' 'S1' 'S1' … 'S14' 'S14' 'S14'
    detector    (channel) object 784B 'D1' 'D2' 'D4' 'D5' … 'D29' 'D31' 'D32'
  * wavelength  (wavelength) float64 16B 760.0 850.0
Attributes:
    data_type_group:  unprocessed raw
```

```
array(['S13D26', 'S14D28'], dtype=object)
```

References

[32]:
```python
cedalion.bib.dump_to_notebook()
```

```
<IPython.core.display.HTML object>
```

Next: 4 — Model-driven Analysis — GLM-based HRF estimation.

S4: Model-driven (GLM) Analysis

This tutorial demonstrates how to use a General Linear Model (GLM) to model the recorded time series as a superposition of hemodynamic responses and nuisance effects.

Learning objectives

In this notebook you will learn to:

- Build a GLM design matrix with HRF, drift, and short-channel regressors
- Fit the GLM per channel using AR-IRLS and inspect beta coefficients
- Visualise channel-space activation maps on a scalp topography
- Understand the role of short-separation channel regression for noise removal

[1]:
```
# This cells setups the environment when executed in Google Colab.
try:
    import google.colab
    !curl -s https://raw.githubusercontent.com/ibs-lab/cedalion/dev/scripts/
 ↪colab_setup.py -o colab_setup.py
    # Select branch with --branch "branch name" (default is "dev")
    %run colab_setup.py
except ImportError:
    pass
```

[2]:
```
import cedalion
import cedalion.sigproc.quality as quality
import cedalion.sigproc.motion as motion
import cedalion.sigproc.physio as physio


from cedalion import units
import cedalion.xrutils as xrutils
import cedalion.models.glm as glm
import cedalion.data

import cedalion.vis.blocks as vbx
from cedalion.vis.anatomy import scalp_plot, plot_montage3D
from cedalion.vis.colors import p_values_cmap

import numpy as np
import xarray as xr

import matplotlib.pyplot as p
from statsmodels.stats.multitest import multipletests

xr.set_options(display_expand_data=False)
xrutils.unit_stripping_is_error()
```

Load Data

The dataset is loaded and events are renamed. Afterwards, the stimulus data frame is summarized.

[3]:
```
rec = cedalion.data.get_fingertappingDOT()

# assign better trial_type labels
rec.stim.cd.rename_events(
    {
        "1": "Control",
        "2": "FTapping/Left",
        "3": "FTapping/Right",
        "4": "BallSqueezing/Left",
        "5": "BallSqueezing/Right",
    }
)
```

[4]:
```
# count trials
rec.stim.groupby("trial_type")[["trial_type"]].count().rename(
    {"trial_type": "# trials"},
    axis=1,
)
```

[4]:
```
                     # trials
trial_type
BallSqueezing/Left        17
BallSqueezing/Right       16
Control                   65
FTapping/Left             16
FTapping/Right            16
```

Trim dataset

Trim the dataset to roughly 5 minutes to keep computation times low while still preserving three trials per condition. Also exclude the 'BallSqueezing' conditions.

[5]:
```
tmin = 5
tmax = 315

rec.stim = rec.stim[
    (tmin <= rec.stim.onset)
    & (rec.stim.onset <= tmax)
    & (rec.stim.trial_type.isin(["Control", "FTapping/Left", "FTapping/Right"]))
]
# use a slice to select a time range
rec["amp_cropped"] = rec["amp"].sel(time=slice(tmin,tmax))

# count remaining trials
rec.stim.groupby("trial_type")[["trial_type"]].count().rename(
    {"trial_type": "# trials"},
    axis=1,
)
```

[5]:
```
                # trials
trial_type
Control               10
```

```
FTapping/Left         3
FTapping/Right        3
```

Preprocessing

The same motion-correction methods as described in the third tutorial notebook are applied, and the bad channels identified there are removed. The cleaned optical-density time series are then converted to concentration changes. Finally, a low-pass filter with a 0.5 Hz cutoff is applied to remove the cardiac component.

[6]:
```
rec["od"] = cedalion.nirs.cw.int2od(rec["amp_cropped"])
rec["od_tddr"] = motion.tddr(rec["od"])
rec["od_wavelet"] = motion.wavelet(rec["od_tddr"])

bad_channels = ['S13D26', 'S14D28']

rec["od_clean"] = rec["od_wavelet"].sel(channel=~rec["od"].channel.isin(bad_channels))

# differential pathlength factors
dpf = xr.DataArray(
    [6, 6],
    dims="wavelength",
    coords={"wavelength": rec["amp"].wavelength},
)

rec["conc"] = cedalion.nirs.cw.od2conc(rec["od_clean"], rec.geo3d, dpf,␣
 ↪spectrum="prahl")

# Here we use a lowpass-filter to remove the cardiac component.
# Drift will be modeled in the design matrix.

fmin = 0 * units.Hz
fmax = 0.5 * units.Hz

rec["conc_filtered"] = cedalion.sigproc.frequency.freq_filter(rec["conc"], fmin, fmax)

TS_NAME = "conc_filtered"
```

[7]:
```
display(rec[TS_NAME])
```

```
<xarray.DataArray 'concentration' (chromo: 2, channel: 98, time: 1352)> Size: 2MB
[µM] 0.8276 0.7548 0.7043 0.6868 0.6978 … -0.08036 -0.07753 -0.07426 -0.07086
Coordinates:
  * chromo    (chromo) <U3 24B 'HbO' 'HbR'
  * time      (time) float64 11kB 5.046 5.276 5.505 5.734 … 314.5 314.7 314.9
    samples   (time) int64 11kB 22 23 24 25 26 27 … 1369 1370 1371 1372 1373
  * channel   (channel) object 784B 'S1D1' 'S1D2' 'S1D4' … 'S14D31' 'S14D32'
    source    (channel) object 784B 'S1' 'S1' 'S1' 'S1' … 'S14' 'S14' 'S14'
    detector  (channel) object 784B 'D1' 'D2' 'D4' 'D5' … 'D29' 'D31' 'D32'
```

Montage and Channel Distances

The hexagonal montage has longer (3-3.5cm) and shorter (~1.7-2.2cm) channels. We define a cut-off at 22.5 mm to separate long and short channels.

[8]:
```
plot_montage3D(rec["amp"], rec.geo3d)
```

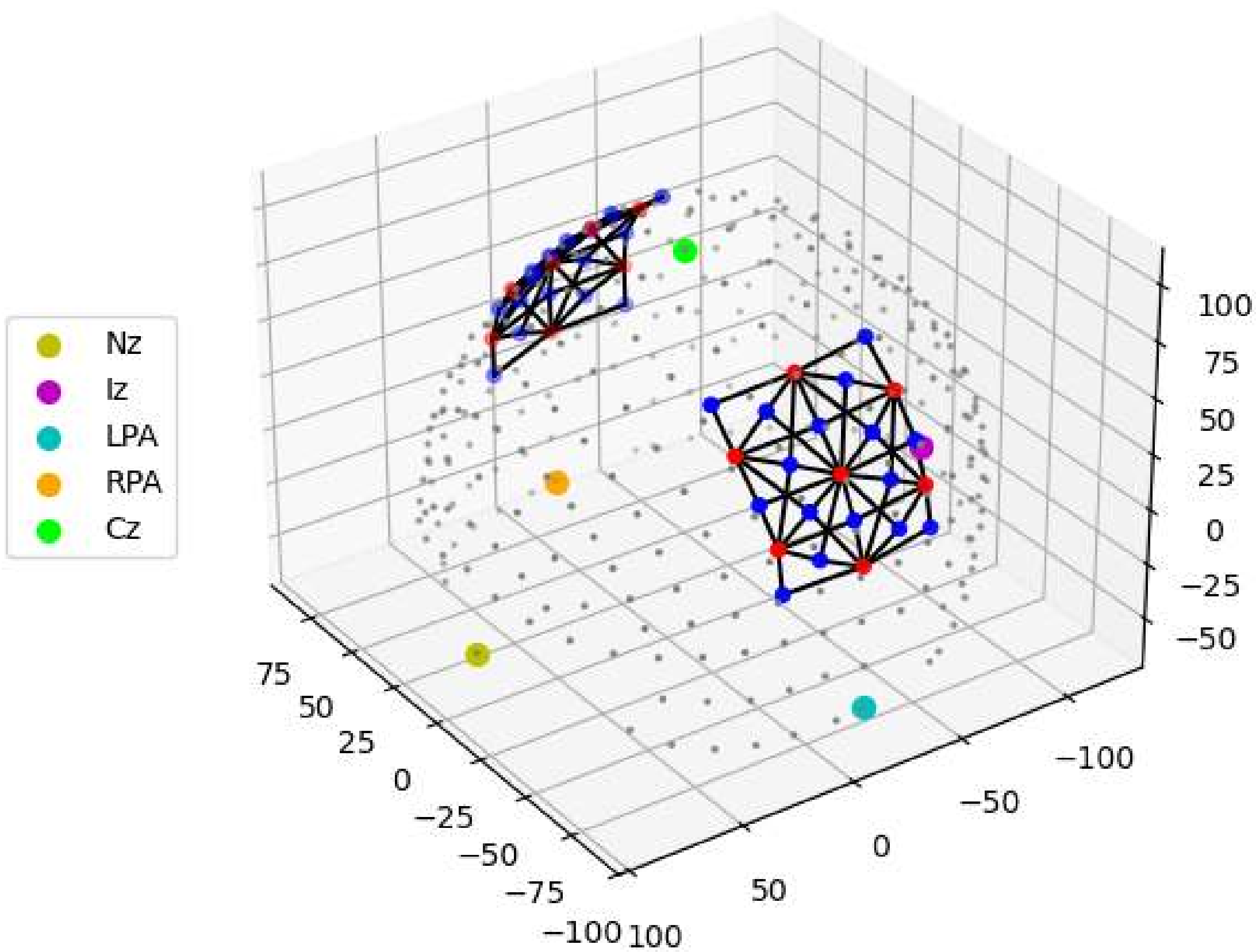

Calculate and histogram channel distances:

[9]:
```
distances = cedalion.nirs.channel_distances(rec["amp"], rec.geo3d)

p.figure(figsize=(8,4))
p.hist(distances, 40)
p.axvline(22.5, c="r", ls="--")
p.xlabel("channel distance / mm")
p.ylabel("channel count");
```

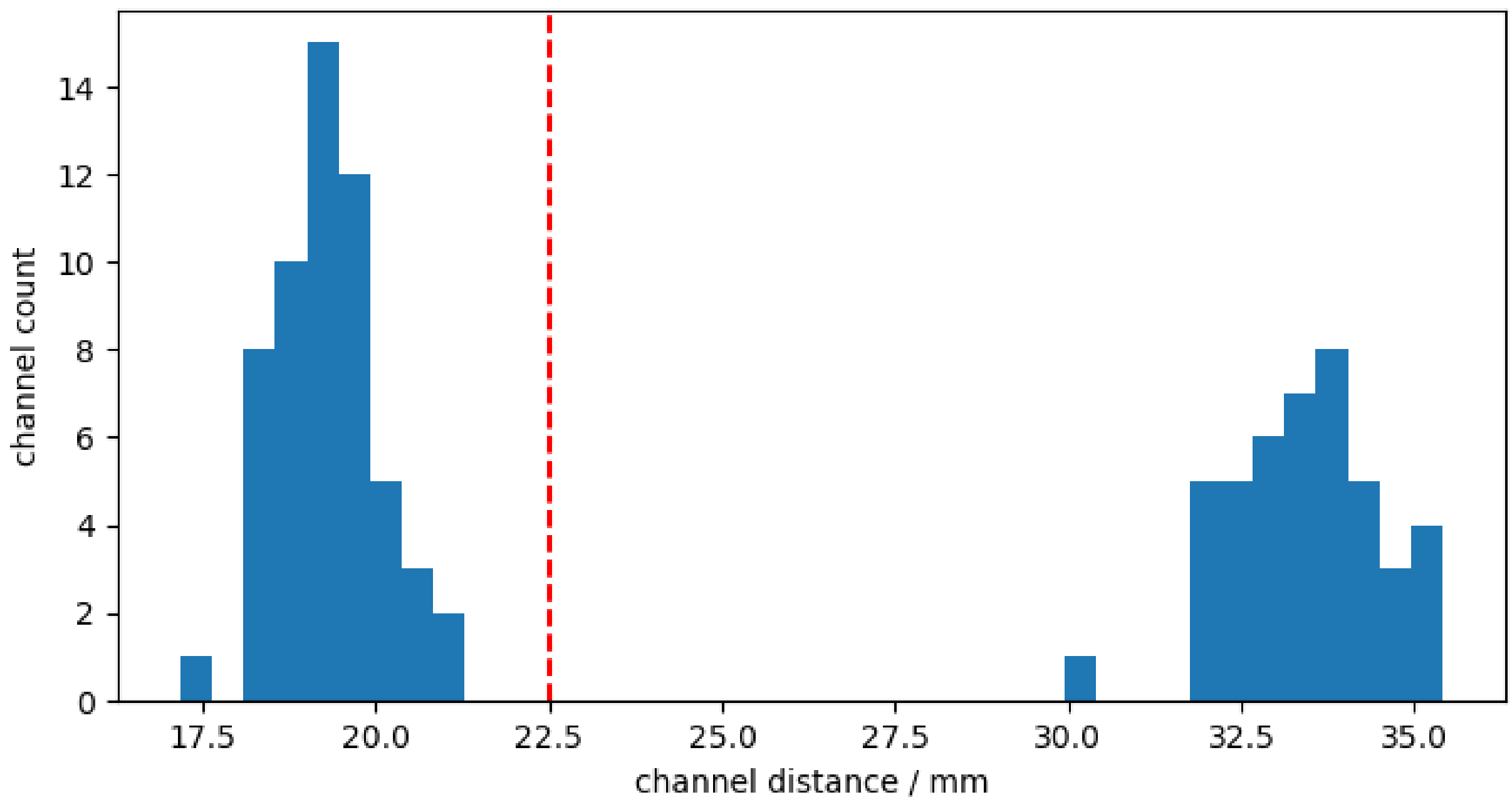

Split long and short channels

[10]:
```python
rec["ts_long"], rec["ts_short"] = cedalion.nirs.split_long_short_channels(
    rec[TS_NAME], rec.geo3d, distance_threshold=22.5 * units.mm
)

# This renaming is for display purposes only.
display(rec["ts_long"].rename("long channels"))
display(rec["ts_short"].rename("short channels"))
```

```
<xarray.DataArray 'long channels' (chromo: 2, channel: 44, time: 1352)> Size: 952kB
[µM] 0.3715 0.3284 0.2975 0.2849 0.2892 … 0.006362 0.008495 0.01122 0.01438
Coordinates:
  * chromo    (chromo) <U3 24B 'HbO' 'HbR'
  * time      (time) float64 11kB 5.046 5.276 5.505 5.734 … 314.5 314.7 314.9
    samples   (time) int64 11kB 22 23 24 25 26 27 … 1369 1370 1371 1372 1373
  * channel   (channel) object 352B 'S1D6' 'S1D8' 'S2D5' … 'S14D25' 'S14D27'
    source    (channel) object 352B 'S1' 'S1' 'S2' 'S2' … 'S13' 'S14' 'S14'
    detector  (channel) object 352B 'D6' 'D8' 'D5' 'D9' … 'D28' 'D25' 'D27'
```

```
<xarray.DataArray 'short channels' (chromo: 2, channel: 54, time: 1352)> Size: 1MB
[µM] 0.8276 0.7548 0.7043 0.6868 0.6978 … -0.08036 -0.07753 -0.07426 -0.07086
Coordinates:
  * chromo    (chromo) <U3 24B 'HbO' 'HbR'
  * time      (time) float64 11kB 5.046 5.276 5.505 5.734 … 314.5 314.7 314.9
    samples   (time) int64 11kB 22 23 24 25 26 27 … 1369 1370 1371 1372 1373
  * channel   (channel) object 432B 'S1D1' 'S1D2' 'S1D4' … 'S14D31' 'S14D32'
    source    (channel) object 432B 'S1' 'S1' 'S1' 'S1' … 'S14' 'S14' 'S14'
    detector  (channel) object 432B 'D1' 'D2' 'D4' 'D5' … 'D29' 'D31' 'D32'
```

Fitting a General Linear Model

The GLM for fNIRS explains the time series (Y) for each channel and chromophore individually as a linear superposition of regressors, some representing the hemodynamic response and others addressing nuisance effects such as superficial or physiological components and signal drifts. The regressors form the design matrix (G) and the fit determines the unknown coefficients ( ) for each regressor in the presence of unmodeled noise (E).

$$Y = G\beta + E$$

Building the Design Matrix

The design matrix comprises the hemodynamic response, short channel and drift regressors.

We model the hemodynamic response function (HRF) using a set of Gaussian basis kernels. The basis is defined by the durations of pre- and post-stimulus periods, the temporal spacing between successive kernels, and the standard deviation (width) of each Gaussian.

Using an instance of `cedalion.models.glm.basis_functions.GaussianKernels`, a basis for the long-channel time series is created and visualized.

[11]:
```
gaussian_kernels = glm.basis_functions.GaussianKernels(
    t_pre=2 * units.s,
    t_post=15 * units.s,
    t_delta=1.5 * units.s,
    t_std=2 * units.s,
)

hrf_basis = gaussian_kernels(rec["ts_long"])

for i in range(hrf_basis.sizes["component"]):
    p.plot(hrf_basis.time, hrf_basis[:,i])
    p.xlabel("relative time / s")
p.axvline(0, c="k")
```

[11]:
```
<matplotlib.lines.Line2D at 0x74d73f4f2d50>
```

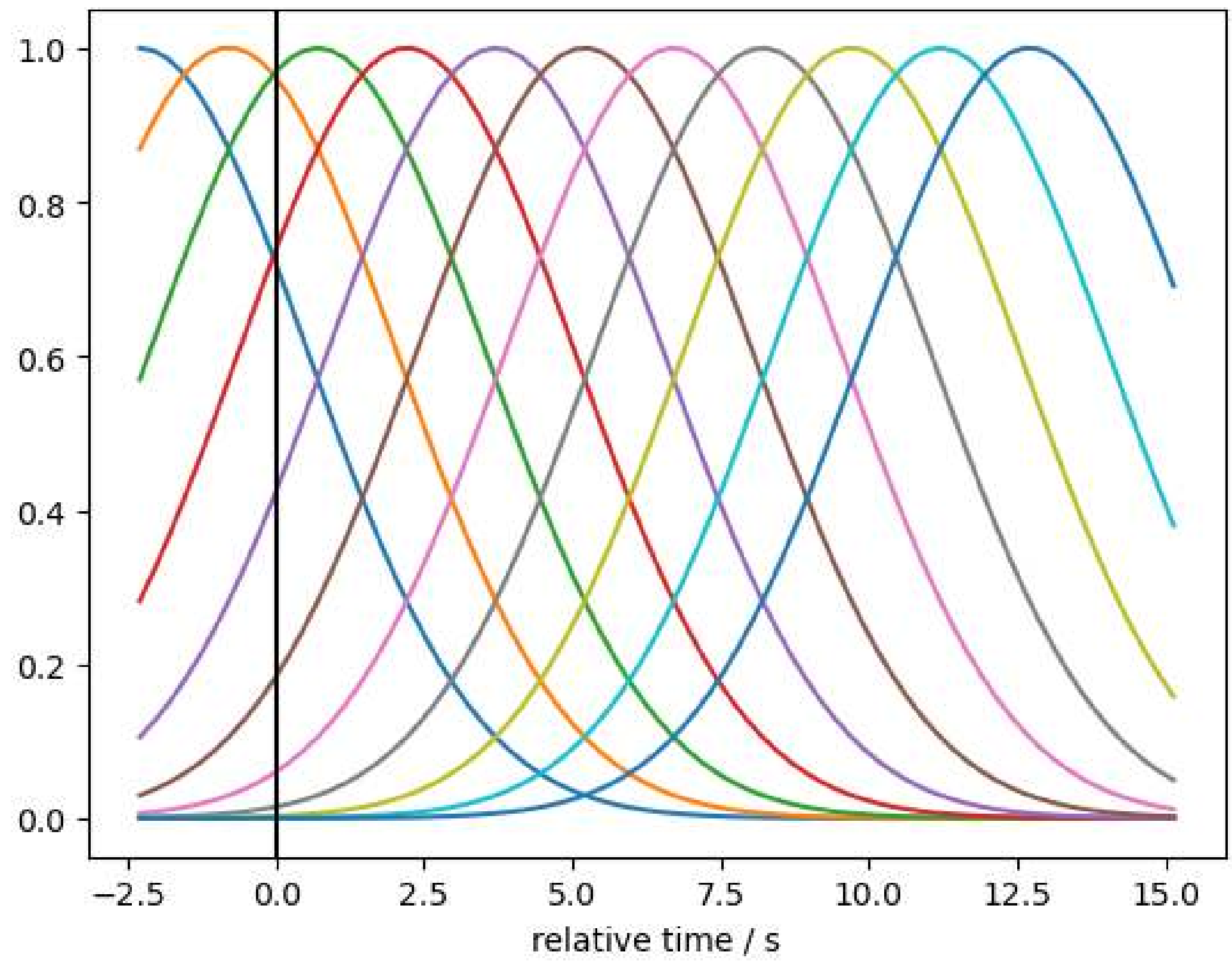

In Cedalion the class `glm.DesignMatrix` manages the regressors.

Functions in the package `glm.design_matrix` create regressors and return them as instances of `DesignMatrix`. These can be combined with the `&`-operator.

In this example, all short channels are averaged to model a global (mostly) superficial component.

Any remaining signal drift is modeled with cosine basis functions up tp 0.02 Hz.

[12]:
```
dms = (
    glm.design_matrix.hrf_regressors(
        rec["ts_long"],
        rec.stim,
        gaussian_kernels,
    )
    & glm.design_matrix.drift_cosine_regressors(rec[TS_NAME], fmax=0.02 * units.Hz)
    & glm.design_matrix.average_short_channel_regressor(rec["ts_short"])
)
```

When fitting a GLM to fNIRS data, some regressors are shared across all channels, wheras others are channel-specific. For example, if superficial physiology is modeled using the nearest short-separation channel for each measurement channel, the resulting short-channel regressor is channel-specific and therefore differs across channels.

Consequently, the `DesignMatrix` distinguishes between common regressors and channel-wise regressors. In this example the design matrix has only common regressors.

Each regressor has a string label for clarity. The convention used by Cedalion is to use labels of the form `'HRF <trial_typ> <number>'` for the HRF regressors and `'Drift <type> <number>'` for the drift components.

As with the trial-type labels, adopting this naming scheme makes it straightforward to select regressors. In this instance, the stimulus 'BallSqueezing/Left' is modeled with multiple regressors. They can be easily distinguished from other HRF or drift terms by selecting labels that begin with 'HRF BallSqueezing/Left'.

[13]:
```
display(dms)
display(dms.common)
display(dms.channel_wise)
```

```
DesignMatrix(common=['HRF Control 00','HRF Control 01','HRF Control 02','HRF Control␣
↪03','HRF Control 04','HRF Control 05','HRF Control 06','HRF Control 07','HRF Control␣
↪08','HRF Control 09','HRF Control 10','HRF FTapping/Left 00','HRF FTapping/Left␣
↪01','HRF FTapping/Left 02','HRF FTapping/Left 03','HRF FTapping/Left 04','HRF␣
↪FTapping/Left 05','HRF FTapping/Left 06','HRF FTapping/Left 07','HRF FTapping/Left␣
↪08','HRF FTapping/Left 09','HRF FTapping/Left 10','HRF FTapping/Right 00','HRF␣
↪FTapping/Right 01','HRF FTapping/Right 02','HRF FTapping/Right 03','HRF FTapping/
↪Right 04','HRF FTapping/Right 05','HRF FTapping/Right 06','HRF FTapping/Right␣
↪07','HRF FTapping/Right 08','HRF FTapping/Right 09','HRF FTapping/Right 10','Drift␣
↪Cos 0','Drift Cos 1','Drift Cos 2','Drift Cos 3','Drift Cos 4','Drift Cos 5','Drift␣
↪Cos 6','Drift Cos 7','Drift Cos 8','Drift Cos 9','Drift Cos 10','Drift Cos␣
↪11','short'], channel_wise=[])
```

```
<xarray.DataArray (time: 1352, regressor: 46, chromo: 2)> Size: 995kB
0.0 0.0 0.0 0.0 0.0 0.0 0.0 … 0.9999 0.9999 -0.9999 -0.9999 0.4137 0.03177
Coordinates:
  * time       (time) float64 11kB 5.046 5.276 5.505 5.734 … 314.5 314.7 314.9
  * regressor  (regressor) <U21 4kB 'HRF Control 00' … 'short'
  * chromo     (chromo) <U3 24B 'HbO' 'HbR'
    samples    (time) int64 11kB 22 23 24 25 26 27 … 1369 1370 1371 1372 1373
```

```
[]
```

Visualize the design matrix

[14]:
```
# select common regressors
dm = dms.common
display(dm)

# using xr.DataArray.plot
f, ax = p.subplots(1,1,figsize=(12,10))
dm.sel(chromo="HbO", time=dm.time < 600).T.plot()
p.title("Shared Regressors")
p.show()
```

```
<xarray.DataArray (time: 1352, regressor: 46, chromo: 2)> Size: 995kB
0.0 0.0 0.0 0.0 0.0 0.0 0.0 … 0.9999 0.9999 -0.9999 -0.9999 0.4137 0.03177
Coordinates:
  * time       (time) float64 11kB 5.046 5.276 5.505 5.734 … 314.5 314.7 314.9
  * regressor  (regressor) <U21 4kB 'HRF Control 00' … 'short'
  * chromo     (chromo) <U3 24B 'HbO' 'HbR'
    samples    (time) int64 11kB 22 23 24 25 26 27 … 1369 1370 1371 1372 1373
```

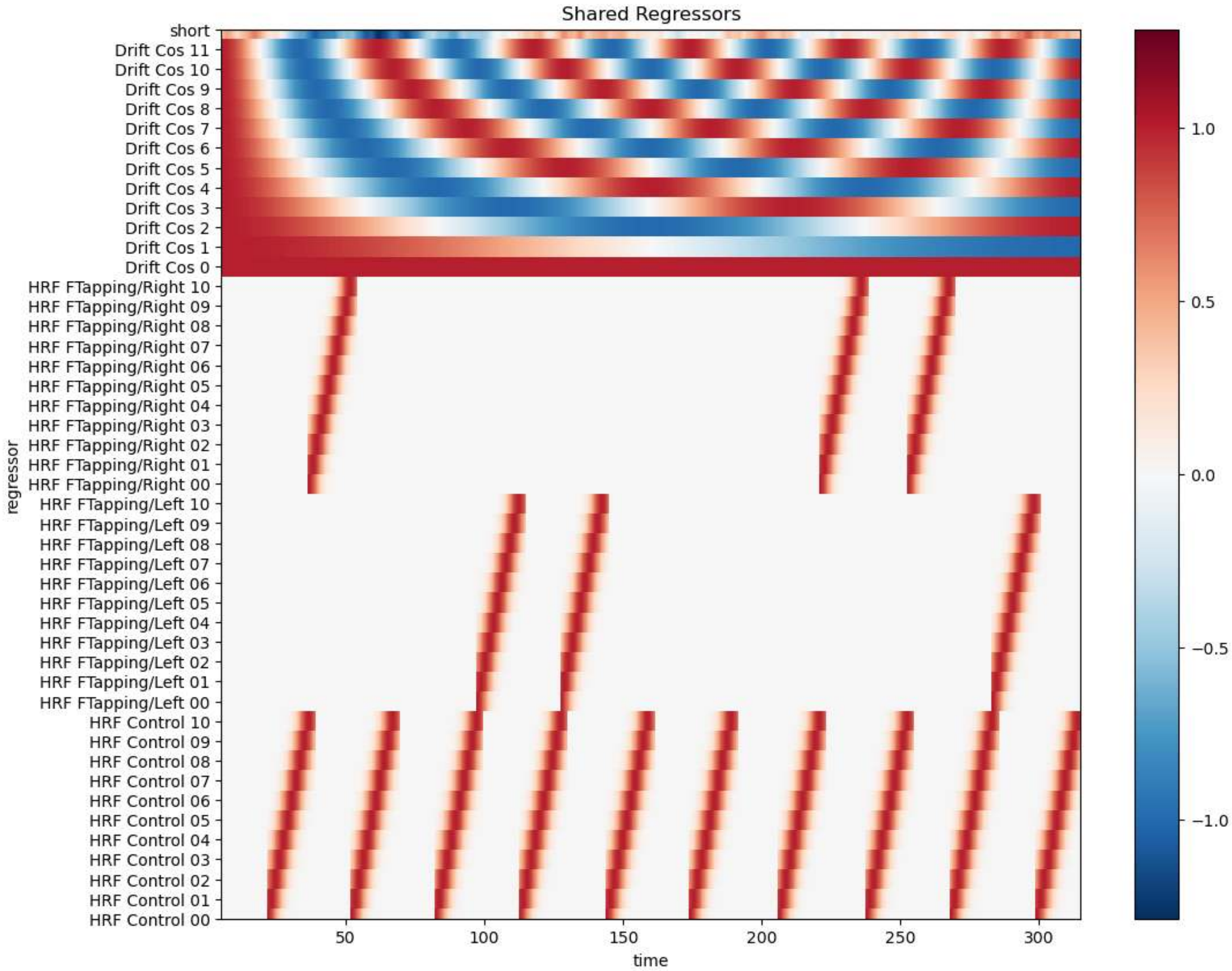

[15]:
```
# line plots of all regressors
f, ax = p.subplots(2,1,sharex=True, figsize=(12,6))

for i, chromo in enumerate(["HbO", "HbR"]):
    for reg in dm.regressor.values:
        label = reg if i == 0 else None
        ax[i].plot(dm.time, dm.sel(chromo=chromo, regressor=reg), label=label)

    vbx.plot_stim_markers(ax[i], rec.stim, y=1)
    ax[i].grid()
    ax[i].set_title(chromo)
    ax[i].set_ylim(-1.5,1.5)
f.suptitle("All Common Regressors")

f.legend(ncol=5, loc="upper center", bbox_to_anchor=(0.5, 0))
#ax[0].set_xlim(0,240);
ax[1].set_xlabel("time / s");
f.set_tight_layout(True)
```

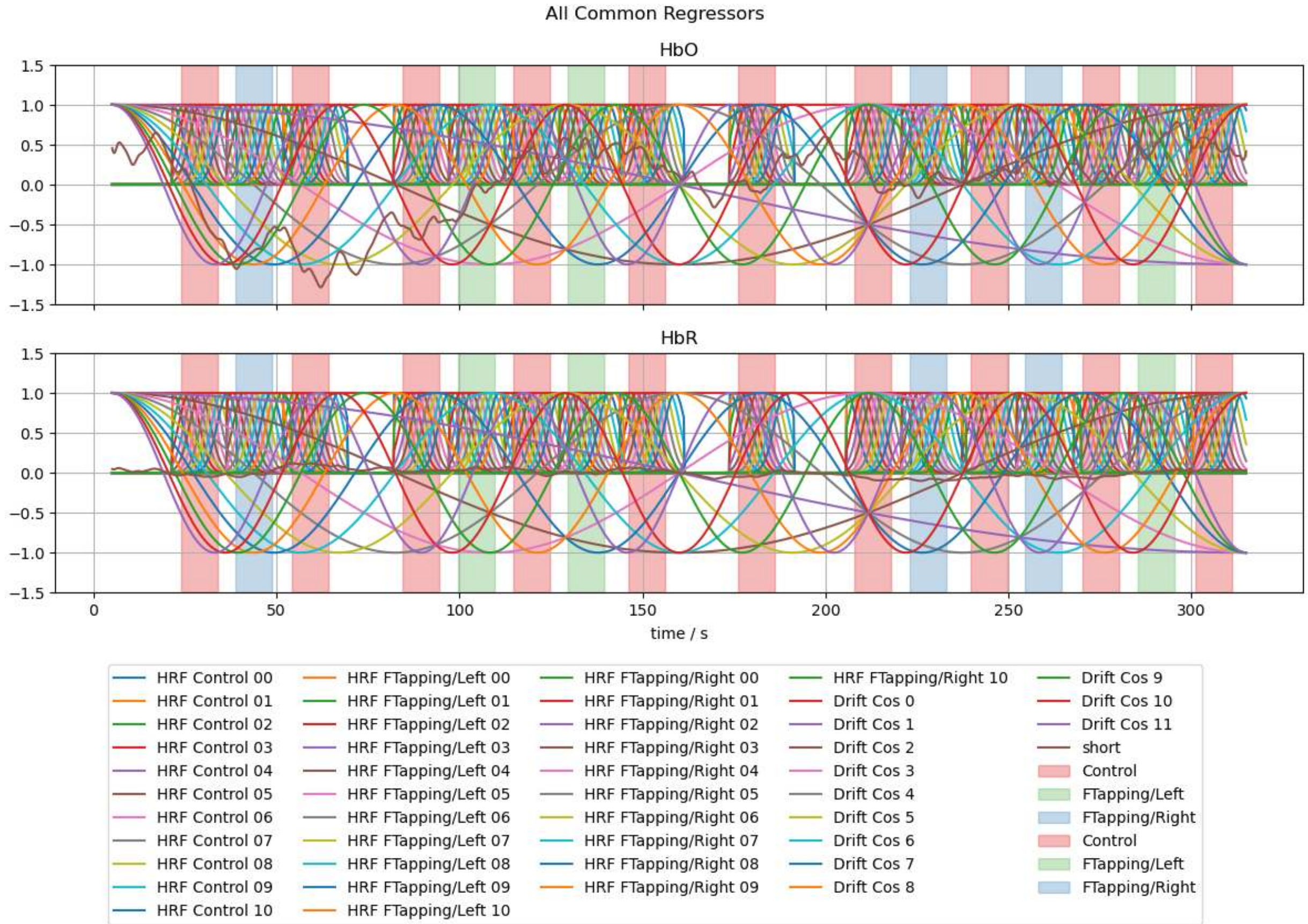

Fitting the Model

To fit the GLM, use `glm.fit`, which requires the time series and the design matrix as inputs. Optionally, you can choose a noise model from the following currently supported options:

- ols (default): ordinary least squares
- rls: recursive least squares
- wls: weighted least squares
- ar_irls: autoregressive iteratively reweighted least squares
- gls: generalized least squares
- glsar: generalized least squares with autoregressive covariance structure

Model fitting is implemented using the `statsmodels` library. Because each channel-chromophore pair is fitted independently, the output consists of one `statsmodels.RegressionResults` object per channel and chromophore. These are arranged in a `DataArray` object with dimensions `('channel', 'chromo')`.

The user can investigate fit results, assess parameter uncertainties and perform hypothesis tests through functions of the `RegressionResults` objects. To facilitate calling these functions on all result objects in a `DataArray`, Cedalion provides the `.sm` accessor. For example, model coefficients (betas) can be obtained with `result.sm.params`, which calls `params` on all result objects and returns a DataArray with the parameters. For the full list of available methods and attributes, see the statsmodels documentation. .

[16]:
```
#results = glm.fit(rec["ts_long"], dms, noise_model="ols", max_jobs=-1)
results = glm.fit(rec["ts_long"], dms, noise_model="ar_irls", max_jobs=-1)

display(results)
```

```
<xarray.DataArray (channel: 44, chromo: 2)> Size: 704B
<statsmodels.robust.robust_linear_model.RLMResultsWrapper object at 0x74d73ef…
Coordinates:
  * chromo    (chromo) <U3 24B 'HbO' 'HbR'
  * channel   (channel) object 352B 'S1D6' 'S1D8' 'S2D5' … 'S14D25' 'S14D27'
    source    (channel) object 352B 'S1' 'S1' 'S2' 'S2' … 'S13' 'S14' 'S14'
    detector  (channel) object 352B 'D6' 'D8' 'D5' 'D9' … 'D28' 'D25' 'D27'
Attributes:
    description:  AR_IRLS
```

Inspecting Fit Results

The result of the fit is an array of statsmodels result objects. Each contains the fitted parameters and other functionality to inspect fit parameter uncertainty or to perform hypothesis tests.

[17]:
```
results[0,0].item().params
```

[17]:
```
HRF Control 00            0.276679
HRF Control 01           -1.322791
HRF Control 02            3.381821
HRF Control 03           -5.959108
HRF Control 04            7.910174
HRF Control 05           -8.189405
HRF Control 06            6.586288
HRF Control 07           -4.007499
HRF Control 08            1.722847
HRF Control 09           -0.484760
HRF Control 10            0.070155
HRF FTapping/Left 00      0.234327
HRF FTapping/Left 01     -0.918640
HRF FTapping/Left 02      1.663890
HRF FTapping/Left 03     -1.340175
HRF FTapping/Left 04     -0.837867
HRF FTapping/Left 05      3.703104
HRF FTapping/Left 06     -4.886997
HRF FTapping/Left 07      3.755480
HRF FTapping/Left 08     -1.792997
HRF FTapping/Left 09      0.528106
HRF FTapping/Left 10     -0.077728
HRF FTapping/Right 00    -0.858642
HRF FTapping/Right 01     4.243697
HRF FTapping/Right 02   -11.265726
HRF FTapping/Right 03    20.680841
HRF FTapping/Right 04   -28.569771
HRF FTapping/Right 05    30.562608
HRF FTapping/Right 06   -25.349188
HRF FTapping/Right 07    15.793779
HRF FTapping/Right 08    -6.882461
HRF FTapping/Right 09     1.949848
HRF FTapping/Right 10    -0.283047
Drift Cos 0               0.019738
Drift Cos 1              -0.062680
```

```
Drift Cos 2            -0.048399
Drift Cos 3            -0.004764
Drift Cos 4             0.098752
Drift Cos 5             0.038450
Drift Cos 6             0.049932
Drift Cos 7             0.015421
Drift Cos 8             0.003108
Drift Cos 9             0.050348
Drift Cos 10            0.032108
Drift Cos 11           -0.038858
short                   0.654660
dtype: float64
```

Cedalion provides the `.sm` accessor on arrays of result objects to make accessing the statsmodels functionality easier.

[18]:
```
display(results.sm.params)
```

```
<xarray.DataArray (channel: 44, chromo: 2, regressor: 46)> Size: 32kB
0.2767 -1.323 3.382 -5.959 7.91 … -0.008269 -0.0007833 0.01253 0.01076 0.2799
Coordinates:
  * regressor  (regressor) object 368B 'HRF Control 00' … 'short'
  * chromo     (chromo) <U3 24B 'HbO' 'HbR'
  * channel    (channel) object 352B 'S1D6' 'S1D8' 'S2D5' … 'S14D25' 'S14D27'
    source     (channel) object 352B 'S1' 'S1' 'S2' 'S2' … 'S13' 'S14' 'S14'
    detector   (channel) object 352B 'D6' 'D8' 'D5' 'D9' … 'D28' 'D25' 'D27'
Attributes:
    description:  AR_IRLS
```

The `glm.predict` function takes the original time series, the design matrix and the fitted parameters. Using these it predicts the sum of all regressors scaled with the best fitted parameters.

When only a subset of the fitted parameters is provided, only those regressors are considered. This allows to predict the signal or background components separately.

[19]:
```
predicted = glm.predict(rec["ts_long"], results.sm.params, dms)

predicted_background = glm.predict(
    rec["ts_long"],
    results.sm.params.sel(
        regressor=results.sm.params.regressor.str.startswith("Drift")
        | results.sm.params.regressor.str.startswith("short")
    ),
    dms,
)
predicted_signal = glm.predict(
    rec["ts_long"],
    results.sm.params.sel(regressor=results.sm.params.regressor.str.startswith("HRF")),
    dms,
)

meas_hrf_only = rec["ts_long"].pint.dequantify() - predicted_background
```

Compare the preprocessed data to the full model

[20]:
```
channels = ["S3D1", "S12D22", "S9D25", "S11D31"]

f, ax = p.subplots(len(channels),1, figsize=(16, 3*len(channels)), sharex=True)

for i_ch, ch in enumerate(channels):
    ax[i_ch].plot(rec["ts_long"].time, rec["ts_long"].sel(channel=ch, chromo="HbO"),␣
 ↪"-", c="orange", label="Data HbO")
    ax[i_ch].plot(rec["ts_long"].time, rec["ts_long"].sel(channel=ch, chromo="HbR"),␣
 ↪"-", c="purple", label="Data HbR")
    ax[i_ch].plot(predicted_background.time, predicted.sel(channel=ch, chromo="HbO"),␣
 ↪"r-", label="Model HbO")
    ax[i_ch].plot(predicted_background.time, predicted.sel(channel=ch, chromo="HbR"),␣
 ↪"b-", label="Model HbR")
    vbx.plot_stim_markers(ax[i_ch], rec.stim, y=1)
    ax[i_ch].set_title(f"{ch} - {distances.sel(channel=ch).item().magnitude:.1f} mm")
    ax[i_ch].set_ylim(-1.75,1.75)
    ax[i_ch].legend(loc="upper left", ncol=8)
```

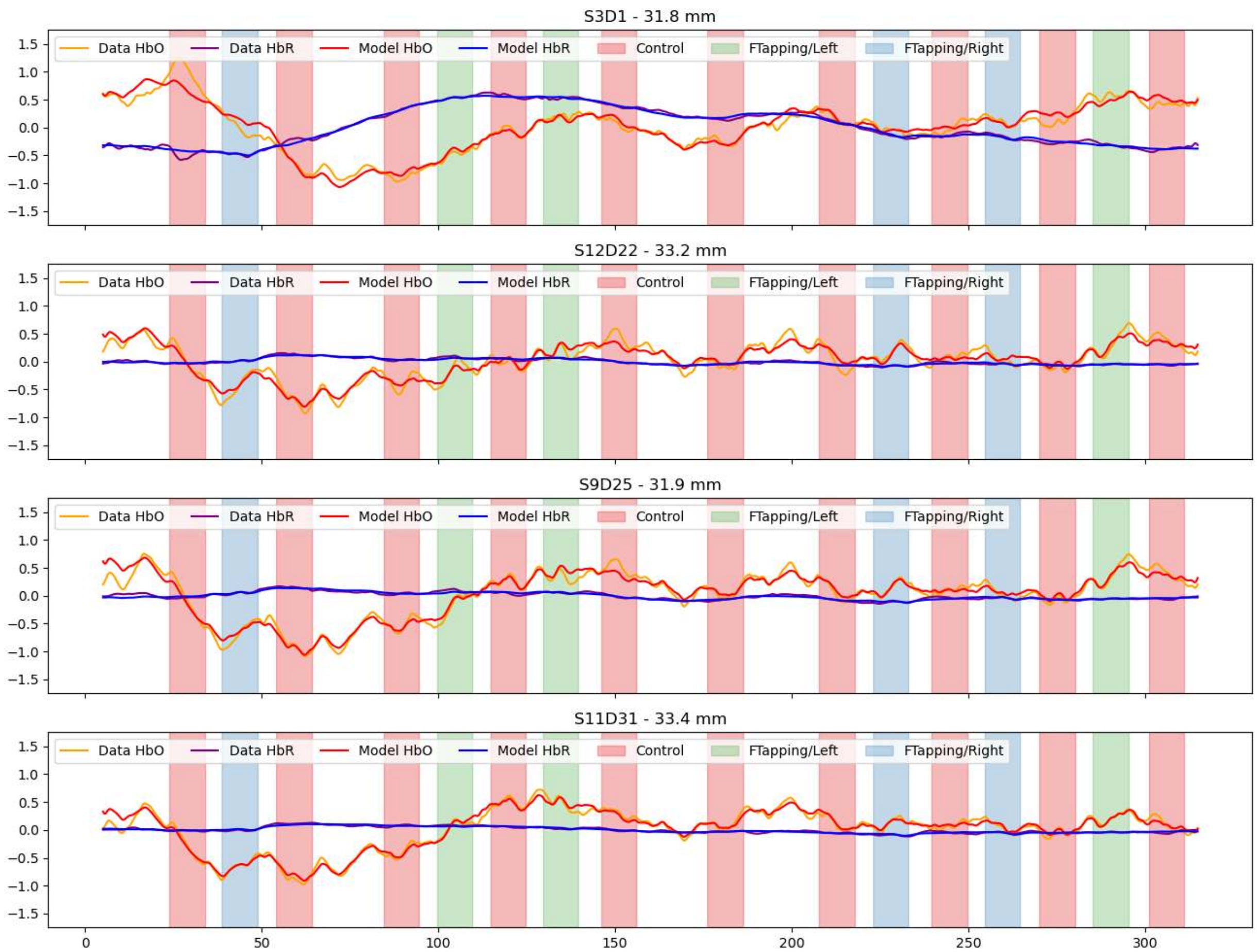

Subtract the components modelled by short and drift regressors and show the fitted activations

[21]:
```
f, ax = p.subplots(len(channels),1, figsize=(16, 3*len(channels)), sharex=True)
```

```
for i_ch, ch in enumerate(channels):
    ax[i_ch].plot(meas_hrf_only.time, meas_hrf_only.sel(channel=ch, chromo="HbO"),␣
 ↪"-", c="orange", label="Data - Model Drift HbO" )
    ax[i_ch].plot(meas_hrf_only.time, meas_hrf_only.sel(channel=ch, chromo="HbR"),␣
 ↪"-", c="purple", label="Data - Model Drift HbR")
    ax[i_ch].plot(predicted_signal.time, predicted_signal.sel(channel=ch,␣
 ↪chromo="HbO"), "r-", label="Model HRF HbO")
    ax[i_ch].plot(predicted_signal.time, predicted_signal.sel(channel=ch,␣
 ↪chromo="HbR"), "b-", label="Model HRF HbR")
    vbx.plot_stim_markers(ax[i_ch], rec.stim, y=1)

    ax[i_ch].legend(loc="upper left", ncol=8)
    ax[i_ch].set_title(ch)
    #ax[i_ch].set_ylim(-1.25, 1.25)
    ax[i_ch].set_ylim(-.5, .5)
```

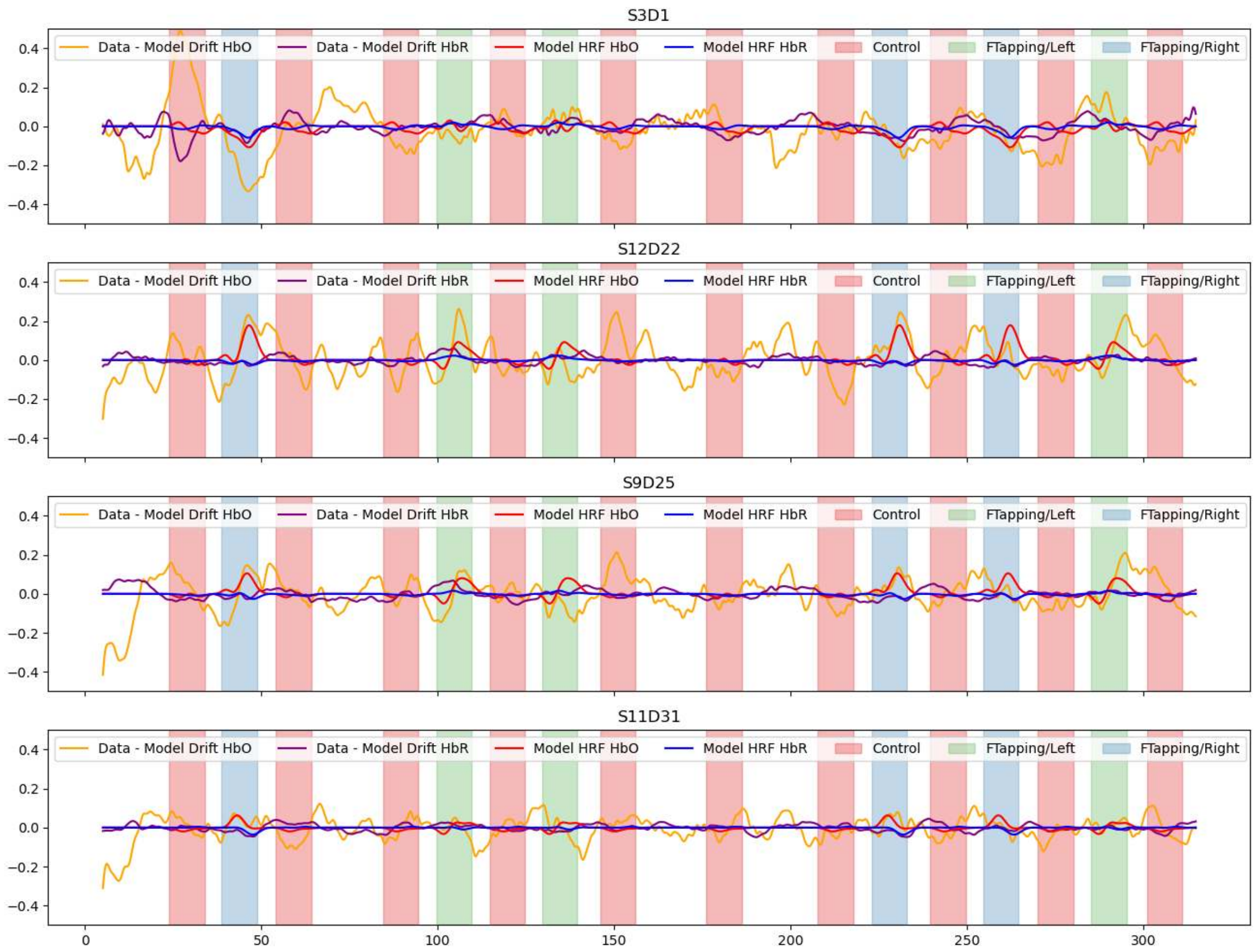

Plot goodness of fit metrics

To assess how well the fitted model describes the data, to metrics are calculated and visualized in scalp plots - the median absolute residuals (MAR) and - the coefficient of determination R^2

Note the scale difference between HbO and HbR for the MAR metric.

[22]:
```
mar = np.abs(predicted - rec["ts_long"].pint.dequantify()).median("time")

ss_res = ((rec["ts_long"].pint.dequantify() - predicted)**2).sum("time")
ss_tot = ((rec["ts_long"] - rec["ts_long"].mean("time"))**2).pint.dequantify().
 ↪sum("time")
r2 = (1 - ss_res / ss_tot)

f,ax = p.subplots(2,2, figsize=(12,10))

for i, chromo in enumerate(["HbO", "HbR"]):
    scalp_plot(
        rec["ts_long"],
        rec.geo3d,
        # results.sm.pvalues.sel(regressor=reg, chromo=chromo),
        mar.sel(chromo=chromo),
        ax[0,i],
        cmap="RdYlGn_r",
        vmin=0,
        vmax={"HbO" : 0.15, "HbR" : 0.05}[chromo],
        title=f"MAR - {chromo}",
        cb_label="MAR / µM",
        channel_lw=2,
        optode_labels=True,
        optode_size=24,
    )
    scalp_plot(
        rec["ts_long"],
        rec.geo3d,
        r2.sel(chromo=chromo),
        ax[1,i],
        cmap="RdYlGn",
        vmin=0,
        vmax=1,
        title=f"$R^2$ - {chromo}",
        cb_label="$R^2$",
        channel_lw=2,
        optode_labels=True,
        optode_size=24,
    )

f.set_tight_layout(True)
```

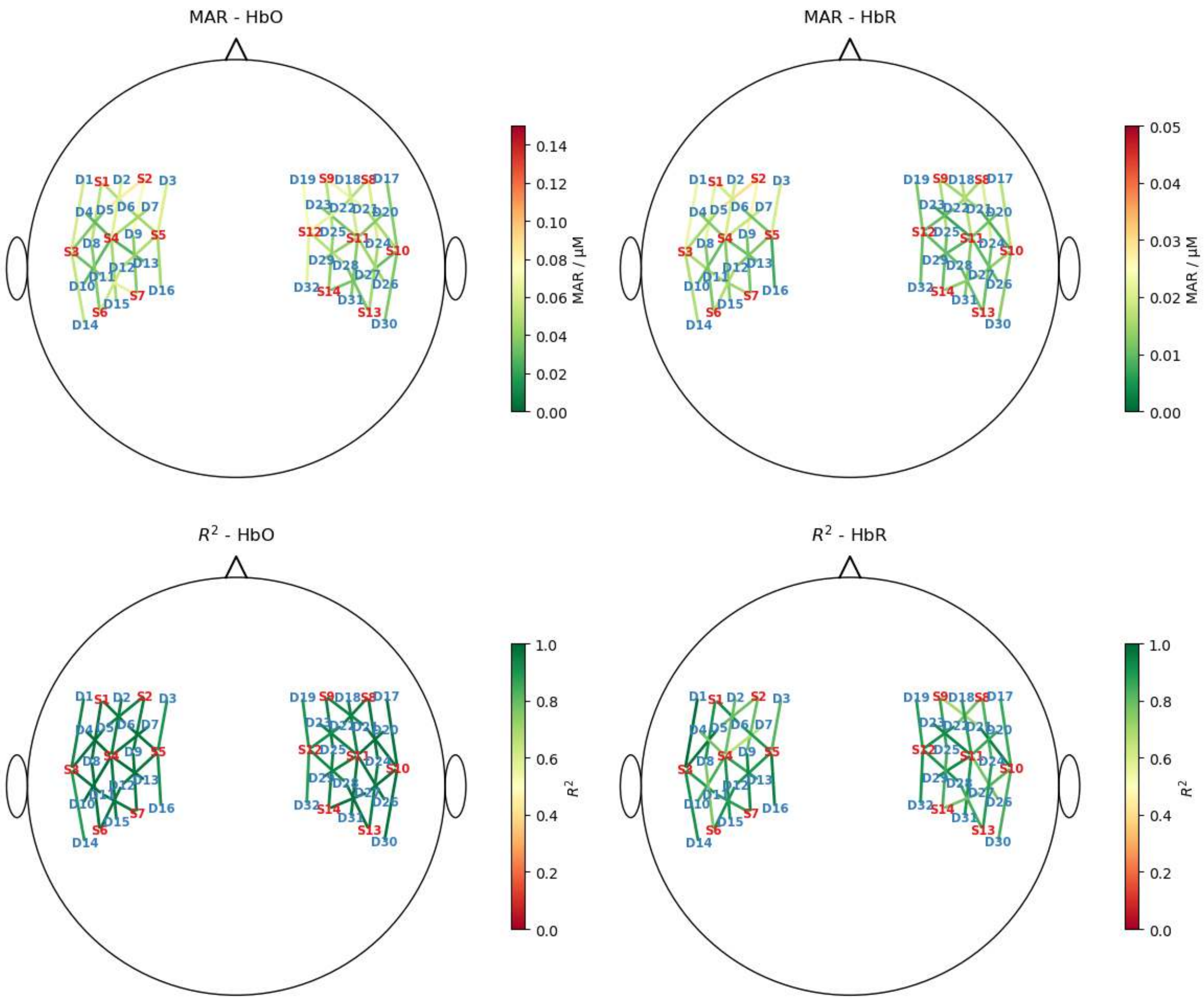

Hypothesis tests

We would like to investigate which channels show activation during finger tapping. Answering this question with a hypothesis test requires specifying a null hypothesis. In this case, the null hypothesis states that the *size of the activation* during left- or right-hand finger tapping does not differ from the control condition.

For simple models with one regressor per condition, `statsmodels` provides a convenient interface for formulating such null hypotheses directly.

But in the present case, the HRF for each condition is modeled using multiple Gaussian kernels and the *size of the activation* needs to be formalized.

The null hypothesis H0 can be formulated as a linear combination of the fitted parameters β_i and a contrast vector c_i which should be zero if the null hypothesis is true. For example, in a fit with only three parameters, this would look like:

$$H0 : x = \begin{pmatrix} \beta_1 \\ \beta_2 \\ \beta_3 \end{pmatrix}^\top \cdot \begin{pmatrix} c_1 \\ c_2 \\ c_3 \end{pmatrix} = 0$$

Through the specification of different contrast vectors, different hypothesis can be formulated. For example:

$$c = \begin{pmatrix} 1 \\ 0 \\ -1 \end{pmatrix} \rightarrow H0 : \beta_1 = \beta_3$$

i.e the contrast vector encodes the hypothesis that there is no difference between β_1 and β_3.

Each such hypothesis is then tested, by checking if the linear combination significantly deviates from zero.

In the present case, we define the *size of the activation* as the area under the HRF. Because the model is linear, the area under the fitted HRF can be computed as a weighted sum of the Gaussian kernel areas A_i, with the weights given by the fitted parameters β_i:

$$A_{HRF} = \begin{pmatrix} \beta_1 \\ \beta_2 \\ \vdots \\ \beta_N \end{pmatrix}^\top \cdot \begin{pmatrix} A_1 \\ A_2 \\ \vdots \\ A_n \end{pmatrix}$$

Comparing with the formulas above, it becomes clear that by using the areas of the kernel basis functions as contrast weights and by assigning opposite signs to the conditions being compared, we can formulate the null hypothesis that the *size of the activation* (as quantified by HRF area) does not differ between two conditions.

Finally, we restrict the area computation to a time window in which the main hemodynamic response is expected, so that the calculated area reflects the activation of interest rather than unrelated fluctuations.

The following cell defines a function that computes contrast vectors from the HRF areas during 5 to 10 seconds post-stimulus then uses this function to formulate the two hypotheses: - “HRF FTapping/Left = HRF Control” and - “HRF FTapping/Right = HRF Control”.

[23]:
```
def gaussian_kernel_timewindowed_auc_contrast(
    dms, df_stim, condition1: str, condition2: str, tmin: float, tmax: float
):
    """This function computes contrast vectors based on the time-windowed are of the␣
 ↪regressors."""

    time = dms.common.time

    # create two masks, that for each condition contains 1.0 only for
    # time samples between onset+tmin and onset+tmax. All other entries
    # zero
    mask_cond1 = np.zeros(len(time))
    mask_cond2 = np.zeros(len(time))

    for _, row in df_stim.iterrows():
        t1, t2 = row["onset"]+tmin, row["onset"]+tmax
        if row["trial_type"].startswith(condition1):
            mask_cond1[(t1 <= time) & (time <= t2)] = 1.
        if row["trial_type"].startswith(condition2):
            mask_cond2[(t1 <= time) & (time <= t2)] = 1.

    # each gaussian regressor is multiplied with the mask for its condition. This sets
    # all parts of the regressor outside the time window to zero. Through integration␣
 ↪the remaining
```

```python
    # area under the curve is calculated.

    nregressors = dms.common.sizes["regressor"]
    contrast = np.zeros(nregressors)
    for i in range(nregressors):
        if dms.common.regressor.values[i].startswith(f"HRF {condition1}"):
            contrast[i] = np.trapezoid(dms.common[:,i,0]*mask_cond1, dms.common.time)
        if dms.common.regressor.values[i].startswith(f"HRF {condition2}"):
            contrast[i] = - np.trapezoid(dms.common[:,i,0]*mask_cond2, dms.common.time)

    return contrast

hypothesis_labels = [
    "HRF FTapping/Left = HRF Control",
    "HRF FTapping/Right = HRF Control",
]

hypotheses = [
    gaussian_kernel_timewindowed_auc_contrast(dms, rec.stim, "FTapping/Left",␣
 ↪"Control", 5, 10),
    gaussian_kernel_timewindowed_auc_contrast(dms, rec.stim, "FTapping/Right",␣
 ↪"Control", 5, 10),
]

display(hypotheses)
```

```
[array([ -0.38536386,  -1.54846768,  -4.83084777, -11.81761093,
        -22.81664425, -35.12676303, -43.40391258, -43.25178978,
        -34.75902577, -22.41329998, -11.52180284,   0.11269669,
          0.45496975,   1.42568445,   3.50208166,   6.7877193 ,
         10.48759214,  13.0030286 ,  12.99994119,  10.48098719,
          6.78010497,   3.49667564,   0.        ,   0.        ,
          0.        ,   0.        ,   0.        ,   0.        ,
          0.        ,   0.        ,   0.        ,   0.        ,
          0.        ,   0.        ,   0.        ,   0.        ,
          0.        ,   0.        ,   0.        ,   0.        ,
          0.        ,   0.        ,   0.        ,   0.        ,
          0.        ,   0.        ]),
 array([ -0.38536386,  -1.54846768,  -4.83084777, -11.81761093,
        -22.81664425, -35.12676303, -43.40391258, -43.25178978,
        -34.75902577, -22.41329998, -11.52180284,   0.        ,
          0.        ,   0.        ,   0.        ,   0.        ,
          0.        ,   0.        ,   0.        ,   0.        ,
          0.        ,   0.        ,   0.12240493,   0.48687159,
          1.50425073,   3.64608706,   6.97863256,  10.65571472,
         13.06351251,  12.91859368,  10.303389  ,   6.59305506,
          3.36289301,   0.        ,   0.        ,   0.        ,
          0.        ,   0.        ,   0.        ,   0.        ,
          0.        ,   0.        ,   0.        ,   0.        ,
          0.        ,   0.        ])]
```

Using the calculated contrasts (i.e. hypotheses) t-tests are perforemd in each channel and chromophore. The

accessor .sm is used to call the t_test function on all results, yielding a DataArray of ContrastResults:

[24]:
```
contrast_results = results.sm.t_test(hypotheses)
display(contrast_results)
```

```
<xarray.DataArray (channel: 44, chromo: 2)> Size: 704B
                             Test for Constraints                          …
Coordinates:
  * chromo    (chromo) <U3 24B 'HbO' 'HbR'
  * channel   (channel) object 352B 'S1D6' 'S1D8' 'S2D5' … 'S14D25' 'S14D27'
    source    (channel) object 352B 'S1' 'S1' 'S2' 'S2' … 'S13' 'S14' 'S14'
    detector  (channel) object 352B 'D6' 'D8' 'D5' 'D9' … 'D28' 'D25' 'D27'
Attributes:
    description:  AR_IRLS
```

Inspecting a single contrast result: the two rows labeled 'c0' and 'c1' correspond to the two hypotheses/contrasts "HRF FTapping/Left = HRF Control" and "HRF FTapping/Right = HRF Control".

[25]:
```
display(contrast_results[0,0].item())
```

```
<class 'statsmodels.stats.contrast.ContrastResults'>
                             Test for Constraints
==============================================================================
                 coef    std err          z      P>|z|      [0.025      0.975]
------------------------------------------------------------------------------
c0             1.0722      0.886      1.211      0.226      -0.664       2.808
c1             1.4259      0.885      1.611      0.107      -0.309       3.161
==============================================================================
```

Apply FDR-control and visualize corrected p-values for all channels

In the following, the p-values of all tests are visualized in a scalp plot. A Benjamini-Hochberg FDR correction is applied.

[26]:
```
# create a colormap for p-values
norm, cmap = p_values_cmap()

nhypo = len(hypotheses)
f, ax = p.subplots(2,len(hypotheses),figsize=(6 * nhypo,8))

for i_row, chromo in enumerate(["HbO", "HbR"]):
    for i_hypo, hypo in enumerate(hypotheses):

        # get p_values for all channels and apply fdr correction
        # use Benjamini-Hochberg here
        p_values = contrast_results.sm.p_values().sel(chromo=chromo, hypothesis=i_hypo)
        _, q_values, _, _ = multipletests(p_values, alpha=0.05, method="fdr_bh")

        scalp_plot(
            rec["ts_long"],
            rec.geo3d,
            np.log10(q_values),
            ax[i_row][i_hypo],
            cmap=cmap,
```

```
            norm=norm,
            title=f"'{hypothesis_labels[i_hypo]}'?\n{chromo}",
            cb_label="BH-corrected p-value",
            channel_lw=2,
            optode_labels=False,
            cb_ticks_labels=[(np.log10(i), str(i)) for i in [0.0001, 0.001, 0.005, 0.
↪01, 0.05, 1.]],
            optode_size=24,
        )


f.tight_layout()
```

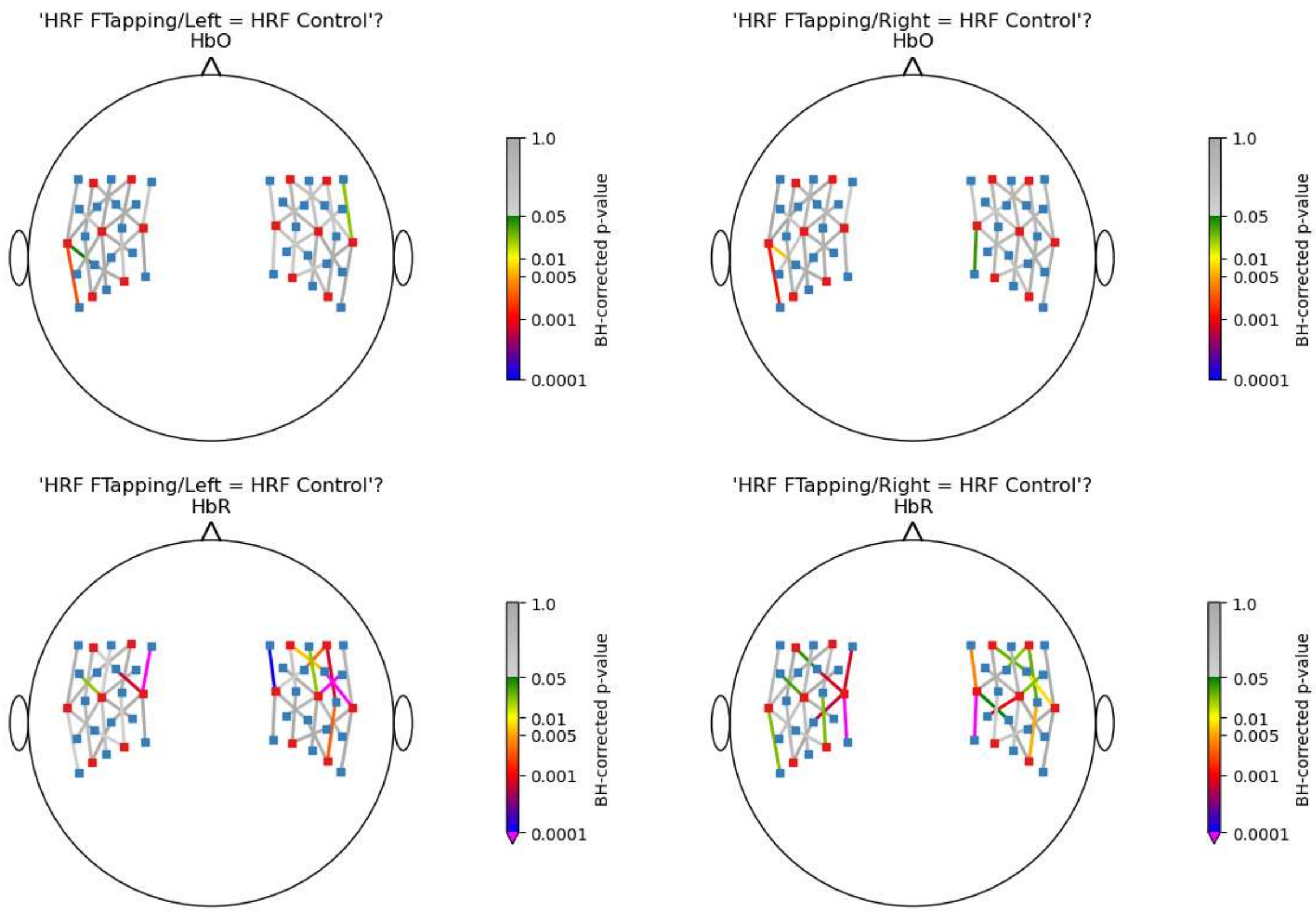

Extract and plot HRFs with uncertainties

For extracting the HRF a special design matrix must be built. It spans only the duration of a single trial, contains only the HRF regressors and that HRF regressors are not convolved over the stimulus duration. The last point is irrelevant for Gaussian-kernel bases, since these kernels are not convolved in the first place.

[27]:
```
dms_extract = glm.design_matrix.hrf_extract_regressors(rec["ts_long"], rec.stim,␣
↪gaussian_kernels)
display(dms_extract.common)
```

```
<xarray.DataArray (time: 77, regressor: 33, chromo: 2)> Size: 41kB
1.0 1.0 0.8694 0.8694 0.5698 0.5698 … 0.1581 0.3804 0.3804 0.6912 0.6912
Coordinates:
```

```
* time       (time) float64 616B -2.294 -2.064 -1.835 … 14.68 14.91 15.14
* regressor  (regressor) <U21 3kB 'HRF Control 00' … 'HRF FTapping/Right 10'
* chromo     (chromo) <U3 24B 'HbO' 'HbR'
```

Passing `dms_extract` to `glm.predict` would predict the hemodynamic response function for the parameters estimated in the fit.

In addition, we would like to assess how the uncertainties of the fitted parameters translate into uncertainties of the extracted HRFs.

Using the covariance matrix of the fitted parameters and a multivariate normal distribution, the function `glm.predict_with_uncertainty` samples sets of parameters around the best fit values. This ensemble of parameter sets, which are still compatible with the reported uncertainties of the fit, are used to calculte the mean HRF as well as an error band (standard deviation) around the mean response.

[28]:
```python
hrfs_control_mean, hrfs_control_std = glm.predict_with_uncertainty(
    rec["ts_long"],
    results,
    dms_extract,
    results.sm.params.regressor.str.startswith("HRF Control"),
)

for i_hypo, trial_type in enumerate(["HRF FTapping/Left", "HRF FTapping/Right"]):
    hrfs_mean, hrfs_std = glm.predict_with_uncertainty(
        rec["ts_long"],
        results,
        dms_extract,
        results.sm.params.regressor.str.startswith(trial_type),
    )

    p_values_hbo = contrast_results.sm.p_values().sel(chromo="HbO", hypothesis=i_hypo)
    p_values_hbr = contrast_results.sm.p_values().sel(chromo="HbR", hypothesis=i_hypo)

    # apply fdr correction (q-values are shown in captions)
    _, q_values_hbo, _, _ = multipletests(p_values_hbo, alpha=0.05, method="fdr_bh")
    _, q_values_hbr, _, _ = multipletests(p_values_hbr, alpha=0.05, method="fdr_bh")

    channels = hrfs_mean.channel.values

    f, ax = p.subplots(5, 9, figsize=(16, 9), sharex=True, sharey=True)
    ax = ax.flatten()

    for i_ch, ch in enumerate(channels[: len(ax)]):
        q_hbo = np.log10(q_values_hbo[i_ch])
        q_hbr = np.log10(q_values_hbr[i_ch])

        mm_hbo = hrfs_mean.sel(channel=ch, chromo="HbO")
        mm_hbr = hrfs_mean.sel(channel=ch, chromo="HbR")
        ss_hbo = hrfs_std.sel(channel=ch, chromo="HbO")
        ss_hbr = hrfs_std.sel(channel=ch, chromo="HbR")

        ax[i_ch].plot(mm_hbo.time, mm_hbo, "r-", label="HbO")
        ax[i_ch].fill_between(
```

```
            mm_hbo.time, mm_hbo - ss_hbo, mm_hbo + ss_hbo, fc="r", alpha=0.3
        )
        ax[i_ch].plot(mm_hbr.time, mm_hbr, "b-", label="HbR")
        ax[i_ch].fill_between(
            mm_hbr.time, mm_hbr - ss_hbr, mm_hbr + ss_hbr, fc="b", alpha=0.3
        )

        mm_hbo = hrfs_control_mean.sel(channel=ch, chromo="HbO")
        mm_hbr = hrfs_control_mean.sel(channel=ch, chromo="HbR")
        ss_hbo = hrfs_control_std.sel(channel=ch, chromo="HbO")
        ss_hbr = hrfs_control_std.sel(channel=ch, chromo="HbR")

        ax[i_ch].plot(mm_hbo.time, mm_hbo, "orange", label="HbO Control")
        ax[i_ch].fill_between(
            mm_hbo.time, mm_hbo - ss_hbo, mm_hbo + ss_hbo, fc="orange", alpha=0.3
        )
        ax[i_ch].plot(mm_hbr.time, mm_hbr, "magenta", label="HbR Control")
        ax[i_ch].fill_between(
            mm_hbr.time, mm_hbr - ss_hbr, mm_hbr + ss_hbr, fc="magenta", alpha=0.3
        )

        ax[i_ch].set_title(
            f"{ch}\nlogq: HbO:{q_hbo:.1f} HbR:{q_hbr:.1f}", fontdict={"fontsize": 8}
        )
        ax[i_ch].set_ylim(-0.3, 0.3)
        ax[i_ch].grid()
        ax[i_ch].axvline(0, c="k", lw=2)
        if i_ch == 0:
            ax[i_ch].legend(loc="upper left")
    f.suptitle(trial_type)
    f.set_tight_layout(True)
```

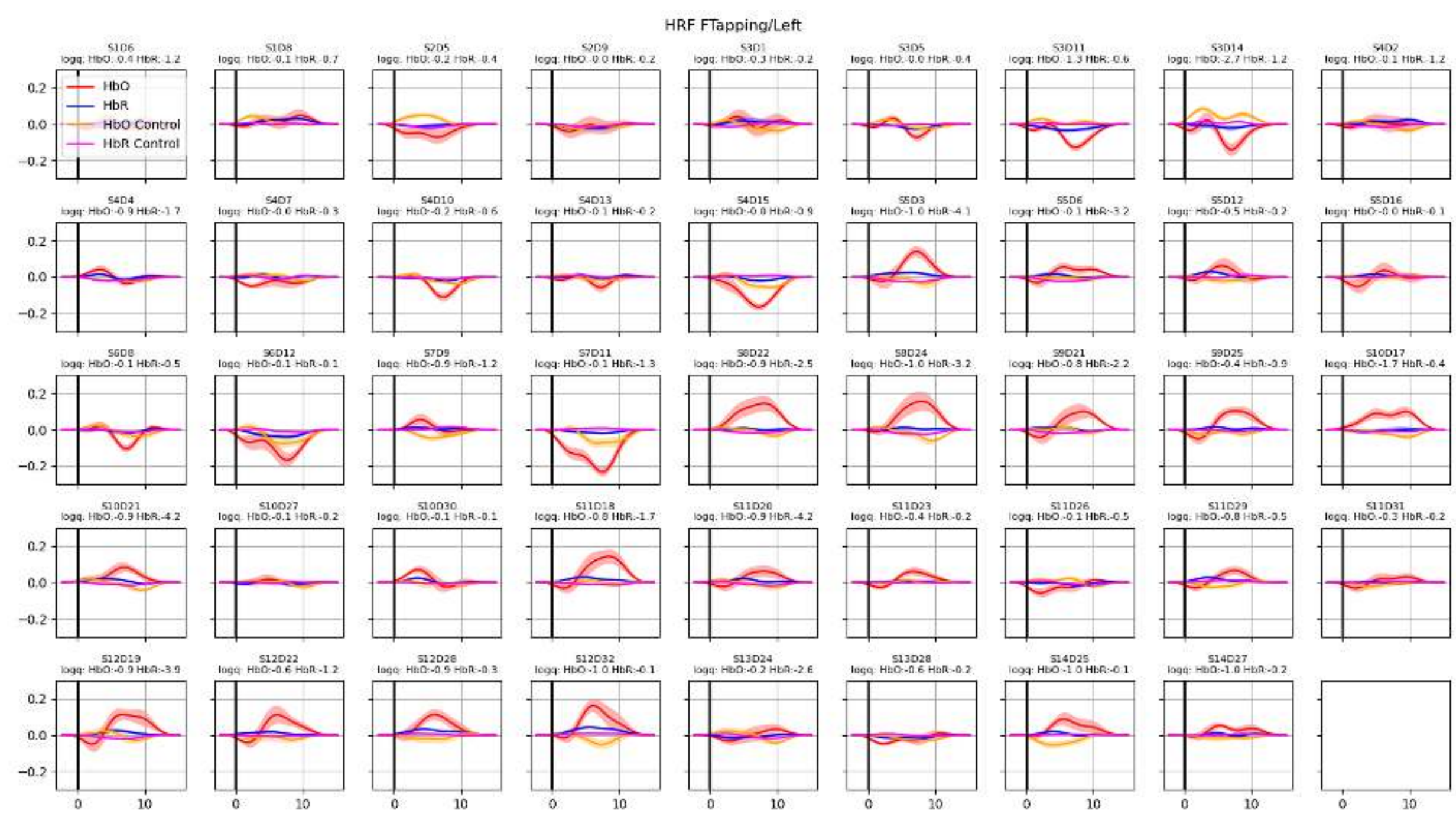

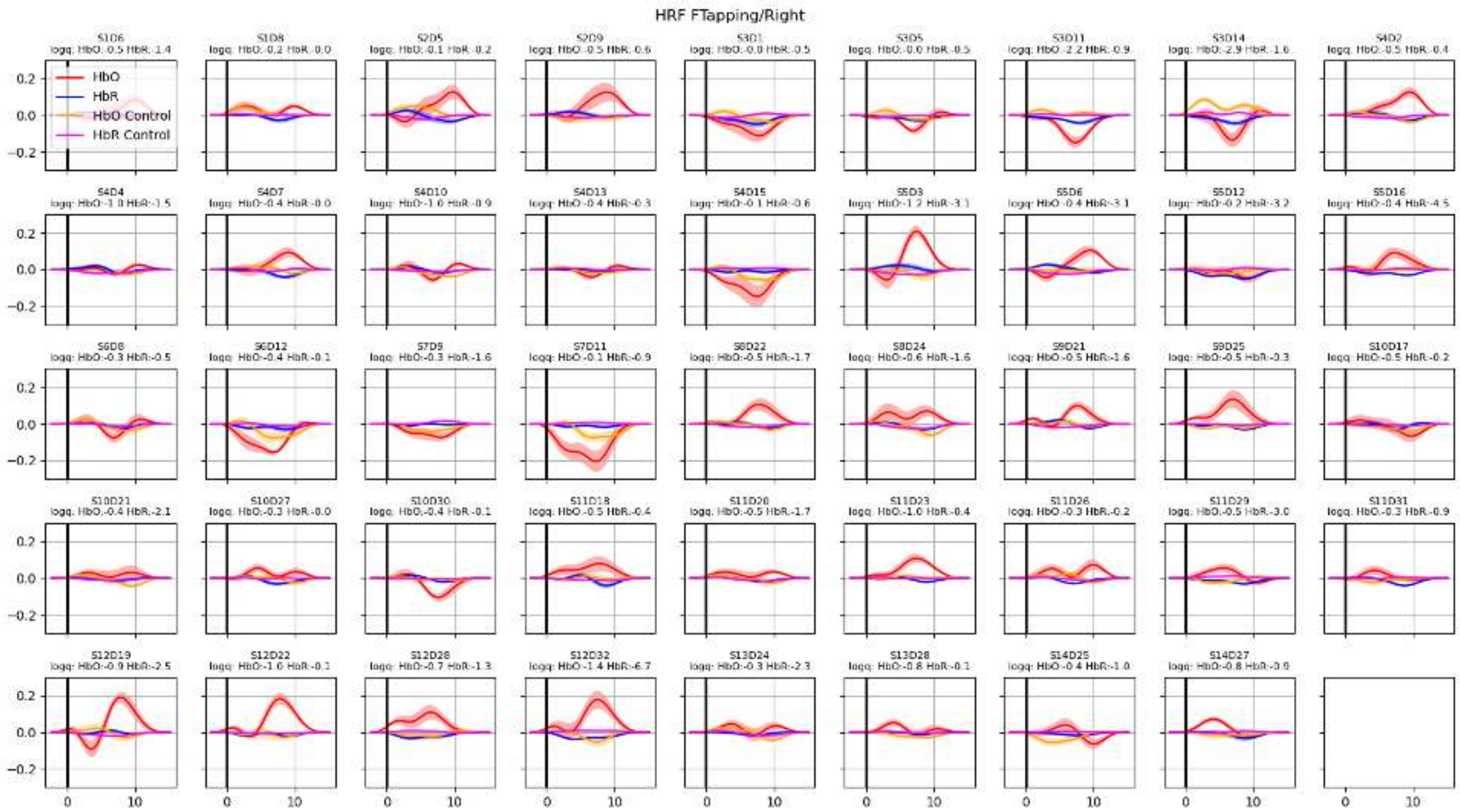

References

[29]:
```
cedalion.bib.dump_to_notebook()
```

<IPython.core.display.HTML object>

Next: 5 — Image Reconstruction — DOT cortical imaging.

S5: DOT - Image Reconstruction

Learning objectives

In this notebook you will learn to:

- Reconstruct HbO/HbR images from channel-space data using the DOT forward model
- Visualise 3-D activation images on the brain surface
- Project vertex-space images onto anatomical parcels for ROI analysis

[1]:
```
# This cells setups the environment when executed in Google Colab.
try:
    import google.colab
    !curl -s https://raw.githubusercontent.com/ibs-lab/cedalion/dev/scripts/
 ↪colab_setup.py -o colab_setup.py
    # Select branch with --branch "branch name" (default is "dev")
    %run colab_setup.py
except ImportError:
    pass
```

Notebook configuration

Decide for an example with a sparse probe or a high density probe for DOT. The notebook will load example data accordingly.

Also specify, if precomputed results of the photon propagation should be used and if the 3D visualizations should be interactive.

[2]:
```
# the head model used in this example
HEAD_MODEL = "colin27"

# the dataset used in this example
DATASET = "fingertappingDOT"

# used precomputed forward model results
PRECOMPUTED_SENSITIVITY = True

FORWARD_MODEL = "MCX"

# set this flag to True to enable interactive 3D plots
INTERACTIVE_PLOTS = False
```

[3]:
```
import pyvista as pv

if INTERACTIVE_PLOTS:
    pv.set_jupyter_backend('server')
else:
    pv.set_jupyter_backend('static')

from pathlib import Path
from tempfile import TemporaryDirectory

import cedalion
import cedalion.data
```

```
import cedalion.dataclasses as cdc
import cedalion.dot
import cedalion.dot as dot
import cedalion.io
import cedalion.sigproc.motion as motion
import cedalion.sigproc.physio as physio
import cedalion.sigproc.quality as quality
import cedalion.vis.anatomy.sensitivity_matrix
import cedalion.vis.blocks as vbx
import matplotlib.pyplot as p
import numpy as np
import xarray as xr
from cedalion import units
from cedalion.io.forward_model import load_Adot
from IPython.display import Image

xr.set_options(display_expand_data=False);
```

[4]:
```
# helper function to display gifs in rendered notbooks
def display_image(fname : str):
    display(Image(data=open(fname,'rb').read(), format='png'))
```

Working Directory

In this notebook tHe output of the fluence and sensitivity calculations are stored in a temporary directory. This will be deleted when the notebook ends.

[5]:
```
temporary_directory = TemporaryDirectory()
working_directory = Path(temporary_directory.name)
```

Load a DOT finger-tapping dataset

- loading via `cedalion.data`
- give stimulus events descriptive string labels

[6]:
```
rec = cedalion.data.get_fingertappingDOT()

rec.stim.cd.rename_events(
    {
        "1": "Control",
        "2": "FTapping/Left",
        "3": "FTapping/Right",
        "4": "BallSqueezing/Left",
        "5": "BallSqueezing/Right",
    }
)
```

The location of the probes is obtained from the snirf metadata.

The units ('mm') are adopted and the coordinate system is named 'digitized'.

[7]:
```
display(rec.geo3d)
print(f"CRS='{rec.geo3d.points.crs}'")
```

```
<xarray.DataArray (label: 346, digitized: 3)> Size: 8kB
[mm] -77.82 15.68 23.17 -61.91 21.23 56.49 … 14.23 -38.28 81.95 -0.678 -37.03
Coordinates:
    type     (label) object 3kB PointType.SOURCE … PointType.LANDMARK
  * label    (label) <U6 8kB 'S1' 'S2' 'S3' 'S4' … 'FFT10h' 'FT10h' 'FTT10h'
Dimensions without coordinates: digitized

CRS='digitized'
```

[8]:
```
cedalion.vis.anatomy.plot_montage3D(rec["amp"], rec.geo3d)
```

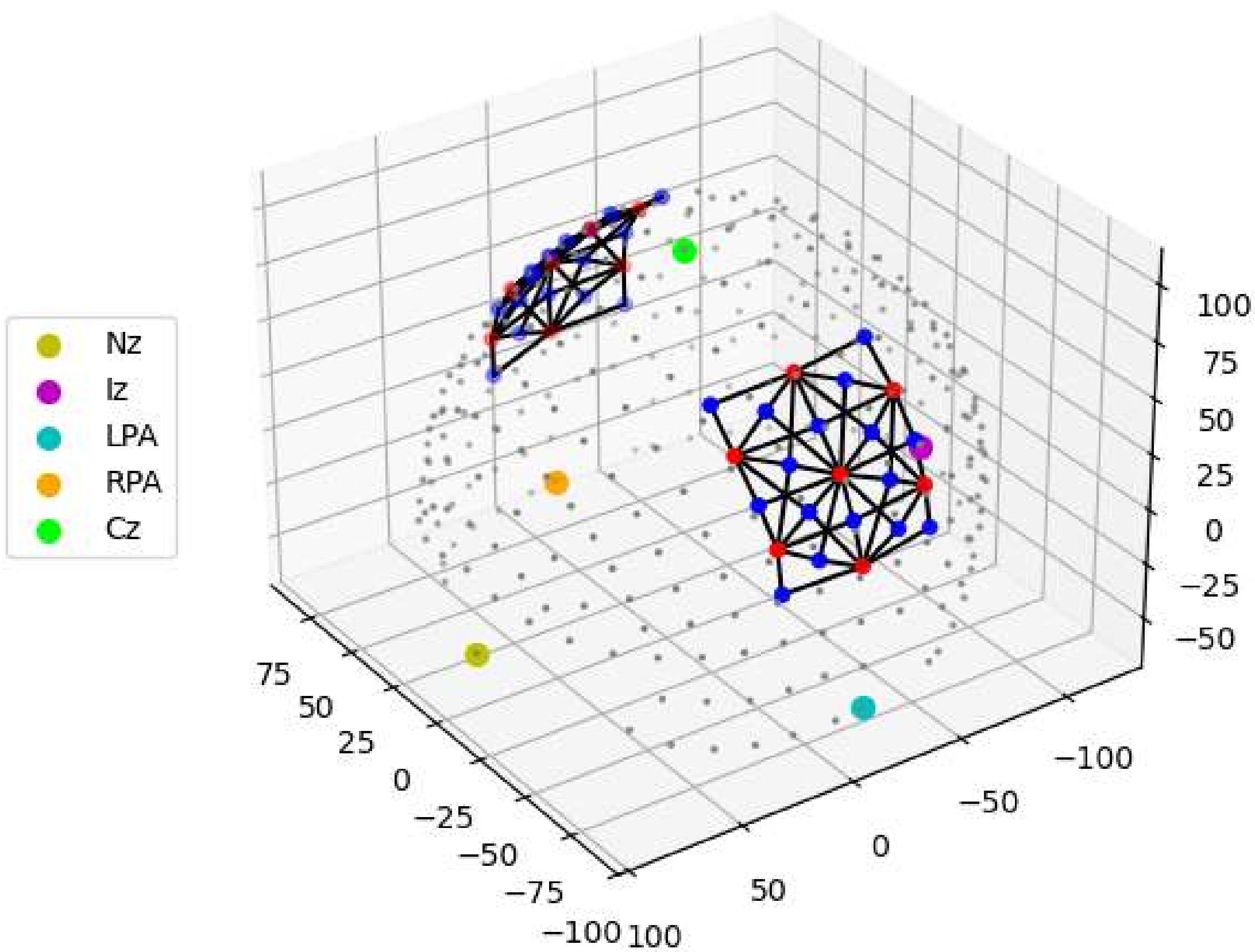

Preprocessing

[9]:
```
rec["od"] = cedalion.nirs.cw.int2od(rec["amp"])
rec["od_tddr"] = motion.tddr(rec["od"])
rec["od_wavelet"] = motion.wavelet(rec["od_tddr"])

# see 2_tutorial_preprocessing.ipynb for channel selection
bad_channels = ['S13D26', 'S14D28']

rec["od_clean"] = rec["od_wavelet"].sel(channel=~rec["od"].channel.isin(['S13D26',␣
↪'S14D28']))
```

```
od_var = quality.measurement_variance(rec["od_clean"], calc_covariance=False)

rec["od_mean_subtracted"], global_comp = physio.global_component_subtract(
    rec["od_clean"], ts_weights=1 / od_var, k=0
)

rec["od_freqfiltered"] = rec["od_mean_subtracted"].cd.freq_filter(
    fmin=0.01, fmax=0.5, butter_order=4
)
```

Calculate block averages in optical density

```
[10]: # segment data into epochs
epochs = rec["od_freqfiltered"].cd.to_epochs(
        rec.stim, # stimulus dataframe
        ["FTapping/Left", "FTapping/Right"],  # select events, discard the others
        before=5 * cedalion.units.s, # seconds before stimulus
        after=30 * cedalion.units.s, # seconds after stimulus
)

# calculate baseline
baseline = epochs.sel(reltime=(epochs.reltime < 0)).mean("reltime")
# subtract baseline
epochs_blcorrected = epochs - baseline

# group trials by trial_type. For each group individually average the epoch dimension
blockaverage = epochs_blcorrected.groupby("trial_type").mean("epoch")

# Plot block averages. Please ignore errors if the plot is too small in the HD case

noPlts2 = int(np.ceil(np.sqrt(len(blockaverage.channel))))
f,ax = p.subplots(noPlts2,noPlts2, figsize=(12,10))
ax = ax.flatten()
for i_ch, ch in enumerate(blockaverage.channel):
    for ls, trial_type in zip(["-", "--"], blockaverage.trial_type):
        ax[i_ch].plot(blockaverage.reltime, blockaverage.sel(wavelength=760,␣
↪trial_type=trial_type, channel=ch), "r", lw=2, ls=ls)
        ax[i_ch].plot(blockaverage.reltime, blockaverage.sel(wavelength=850,␣
↪trial_type=trial_type, channel=ch), "b", lw=2, ls=ls)

    ax[i_ch].grid(1)
    ax[i_ch].set_title(ch.values)
    ax[i_ch].set_ylim(-.01, .01)
    ax[i_ch].set_axis_off()
    ax[i_ch].axhline(0, c="k")
    ax[i_ch].axvline(0, c="k")

p.suptitle("760nm: r | 850nm: b | left: - | right: --")
p.tight_layout()
```

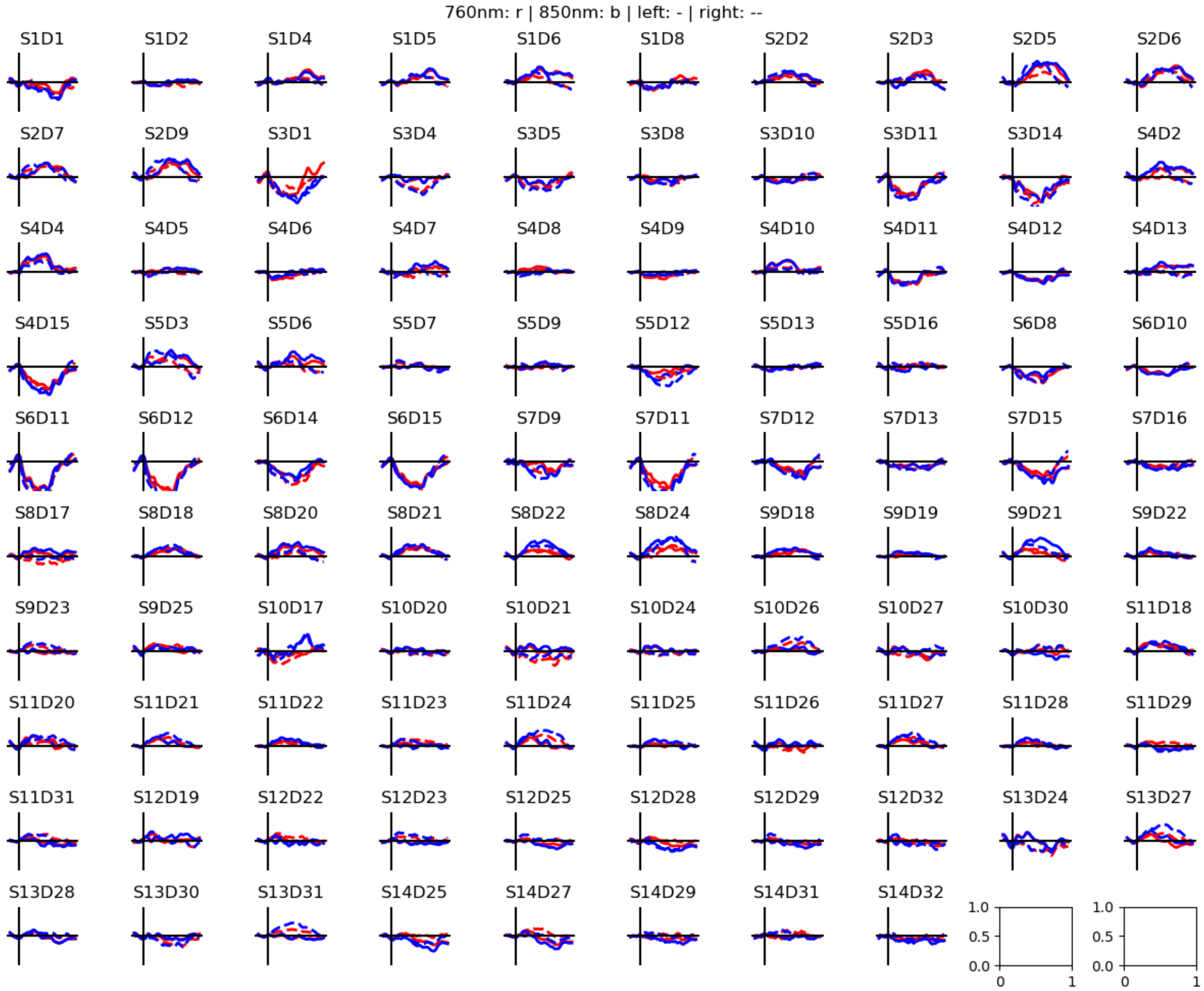

Constructing the TwoSurfaceHeadModel

See 1 - Head models and Forward Modelling for forther options to construct head models.

[11]:
```
if HEAD_MODEL in ["colin27", "icbm152"]:
    # use a factory method for the standard head models
    head_ijk = cedalion.dot.get_standard_headmodel(HEAD_MODEL)
else:
    raise ValueError("unknown head model")
```

Optode Registration

The optode coordinates from the recording must be aligned with the scalp surface. A spring-relaxation algorithm is used to conform the montage to the scalp surface.

[12]:
```
geo3d_snapped_ijk, alignment_details = head_ijk.align_and_relax_to_scalp(rec.geo3d,␣
 ↪rec["amp"])
display(geo3d_snapped_ijk)
```

```
<xarray.DataArray (label: 346, ijk: 3)> Size: 8kB
[] 14.82 141.3 98.21 30.84 146.4 129.8 … 172.5 139.7 41.32 172.7 125.3 42.26
```

```
Coordinates:
    type     (label) object 3kB PointType.SOURCE … PointType.LANDMARK
  * label    (label) <U6 8kB 'S1' 'S2' 'S3' 'S4' … 'FFT10h' 'FT10h' 'FTT10h'
Dimensions without coordinates: ijk
```

[13]:
```
plt = pv.Plotter()
vbx.plot_surface(plt, head_ijk.brain, color="w")
vbx.plot_surface(plt, head_ijk.scalp, opacity=.1)
vbx.plot_labeled_points(plt, geo3d_snapped_ijk)
plt.show()
```

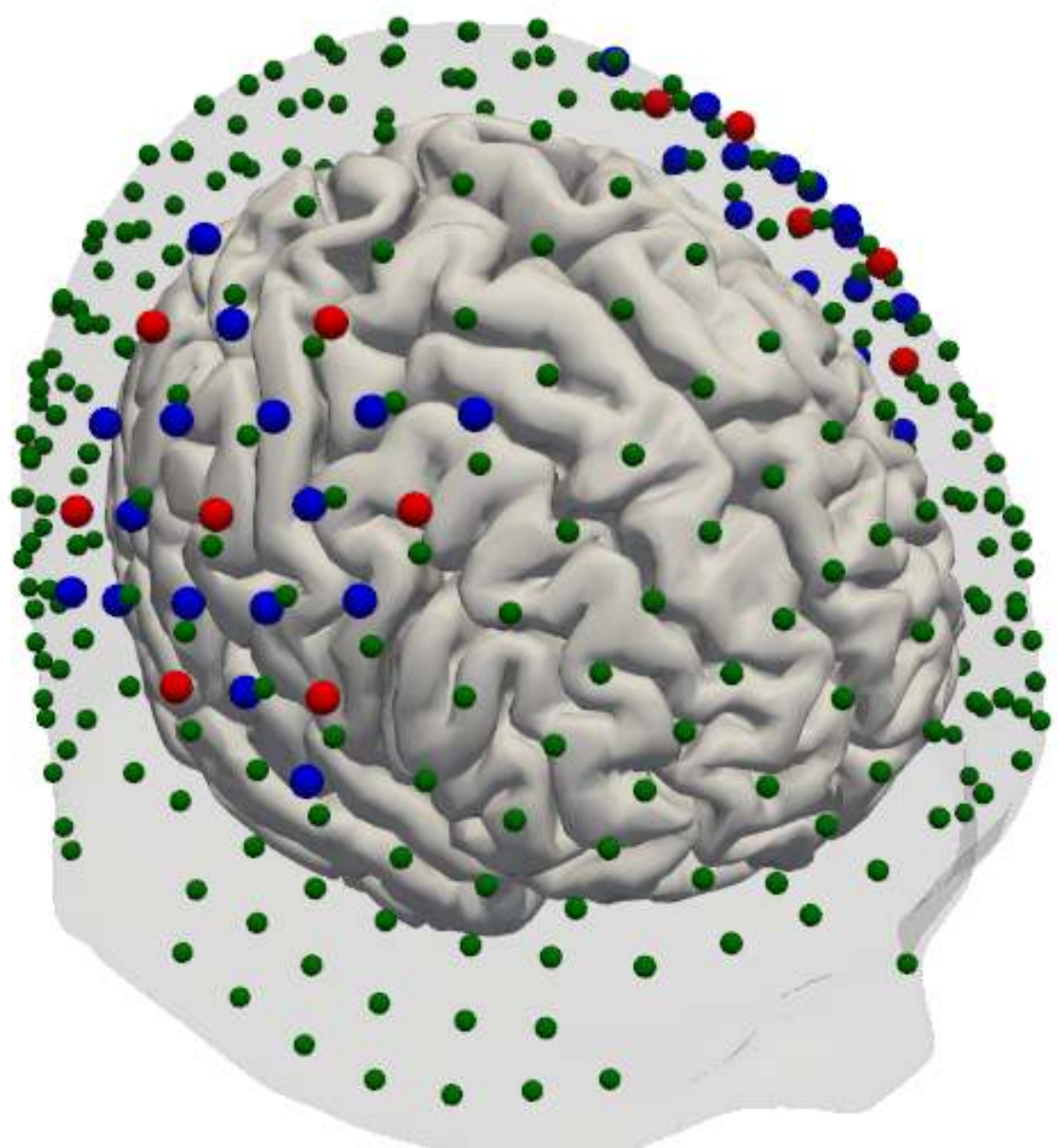

[14]:
```
# remove landmarks from geo3d for subsequent plots
geo3d_plot = geo3d_snapped_ijk[geo3d_snapped_ijk.type != cdc.PointType.LANDMARK]
```

Sensitivity matrix

[15]:
```
Adot = cedalion.data.get_precomputed_sensitivity(DATASET, HEAD_MODEL)
```

Plot Sensitivity Matrix

[16]:
```
plotter = cedalion.vis.anatomy.sensitivity_matrix.Main(
    sensitivity=Adot,
    brain_surface=head_ijk.brain,
    head_surface=head_ijk.scalp,
    labeled_points=geo3d_plot,
)
plotter.plot(high_th=0, low_th=-3)
plotter.plt.show()
```

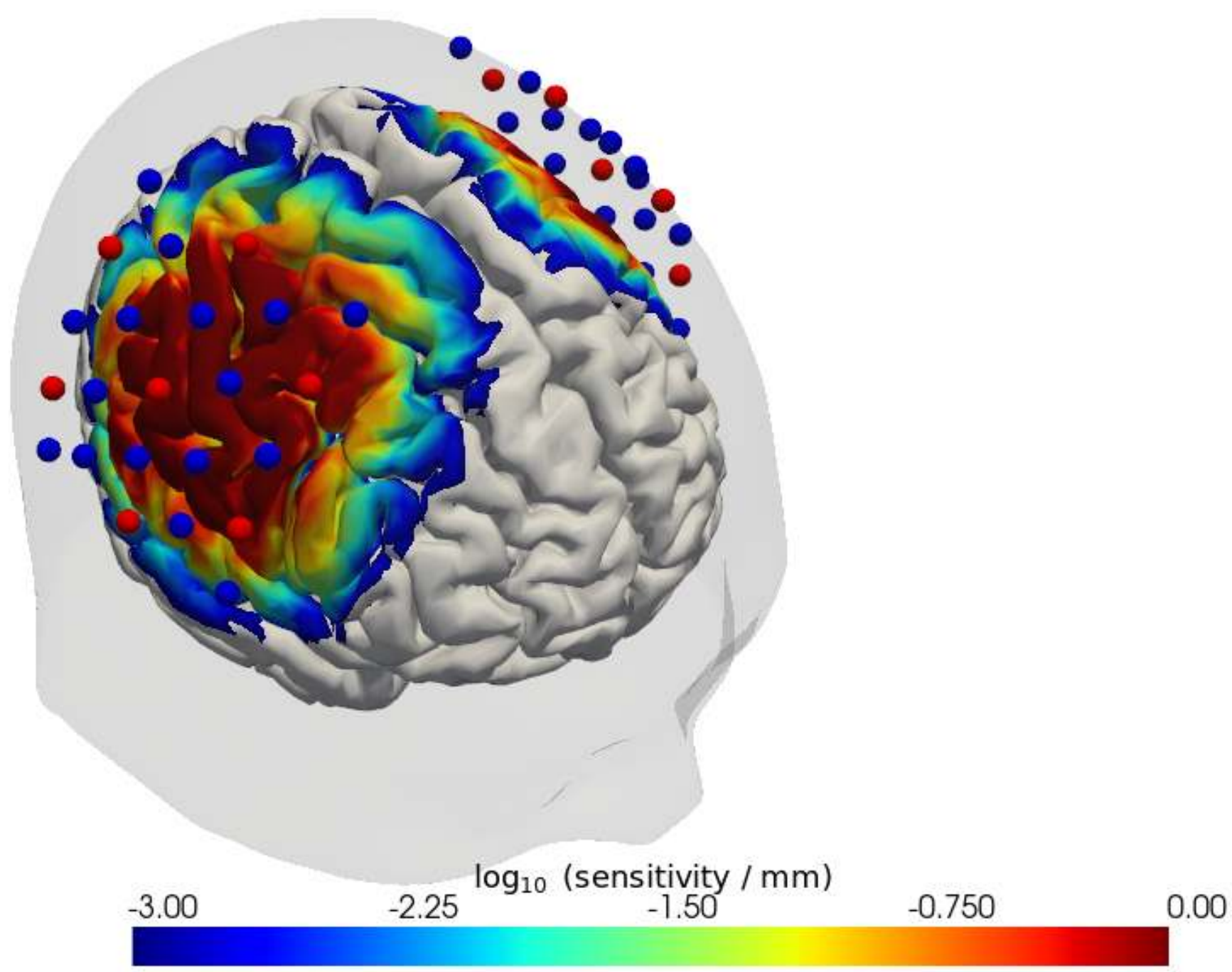

Run the image reconstruction

The class `cedalion.dot.ImageRecon` implements several methods to solve the inverse problem of reconstructing activations in brain and scalp tissue that would explain the measured optical density changes in channel space.

[17]:
```
recon = cedalion.dot.ImageRecon(
    Adot,
    recon_mode="mua2conc",
    brain_only=False,
    alpha_meas=0.01,
    alpha_spatial=None,
```

```
    apply_c_meas=False,
    spatial_basis_functions=None,
)
```

In this example the channel-space block averages should be reconstructed into image space:

[18]:
```
blockaverage
```

[18]:
```
<xarray.DataArray (trial_type: 2, channel: 98, wavelength: 2, reltime: 154)> Size: 483kB
0.001354 0.001405 0.001391 0.001314 … 0.0003461 0.0004625 0.0005884 0.0007154
Coordinates:
  * reltime     (reltime) float64 1kB -5.038 -4.809 -4.58 … 29.54 29.77 30.0
  * channel     (channel) object 784B 'S1D1' 'S1D2' 'S1D4' … 'S14D31' 'S14D32'
    source      (channel) object 784B 'S1' 'S1' 'S1' 'S1' … 'S14' 'S14' 'S14'
    detector    (channel) object 784B 'D1' 'D2' 'D4' 'D5' … 'D29' 'D31' 'D32'
  * wavelength  (wavelength) float64 16B 760.0 850.0
  * trial_type  (trial_type) object 16B 'FTapping/Left' 'FTapping/Right'
```

Run the reconstruction and display results. Note how the OD time series with dimensions (`'channel'`, `'wavelength'`, `...`) transformed into a concentration time series with dimensions (`'chromo'`, `'vertex'`, `...`). The other dimensions (`'trial_type'`, `'reltime'`) did not change.

The `'vertex'` dimension has two coordinates: - `is_brain`: a boolean mask that discriminates between vertices on the brain and scalp surfaces - `parcel`: a string label attributed to each vertex according to a brain parcellation scheme (see section 'Parcel Space')

[19]:
```
img = recon.reconstruct(blockaverage)
img
```

[19]:
```
<xarray.DataArray (chromo: 2, vertex: 35050, trial_type: 2, reltime: 154)> Size: 173MB
[µM] -1.653e-13 -1.538e-13 -1.424e-13 … -1.305e-12 -1.305e-12 -1.28e-12
Coordinates:
  * chromo      (chromo) <U3 24B 'HbO' 'HbR'
    parcel      (vertex) object 280kB 'VisCent_ExStr_8_LH' … 'scalp'
    is_brain    (vertex) bool 35kB True True True True … False False False
  * reltime     (reltime) float64 1kB -5.038 -4.809 -4.58 … 29.54 29.77 30.0
  * trial_type  (trial_type) object 16B 'FTapping/Left' 'FTapping/Right'
Dimensions without coordinates: vertex
```

Visualizing image reconstruction results

In the following, different cedalion plot functions will be showcased to visualize concentration changes on the brain and scalp surfaces.

Channel Space

The function `cedalion.vis.anatomy.scalp_plot_gif` allows to create an animated gif of channel-space OD changes projected on the scalp.

Here, it is used to show the time course of the blockaveraged OD changes.

[20]:
```
# configure the plot
data_ts = blockaverage.sel(wavelength=850, trial_type="FTapping/Right")
# scalp_plot_gif expects the time dimension to be named 'time'
```

```python
data_ts = data_ts.rename({"reltime": "time"})
filename_scalp = "scalp_plot_ts"

# call plot function
cedalion.vis.anatomy.scalp_plot_gif(
    data_ts,
    rec.geo3d,
    filename=filename_scalp,
    time_range=(-5, 30, 0.5) * units.s,
    scl=(-0.01, 0.01),
    fps=6,
    optode_size=6,
    optode_labels=True,
    str_title="OD 850 nm",
)
```

[21]:
```python
display_image(f"{filename_scalp}.gif")
```

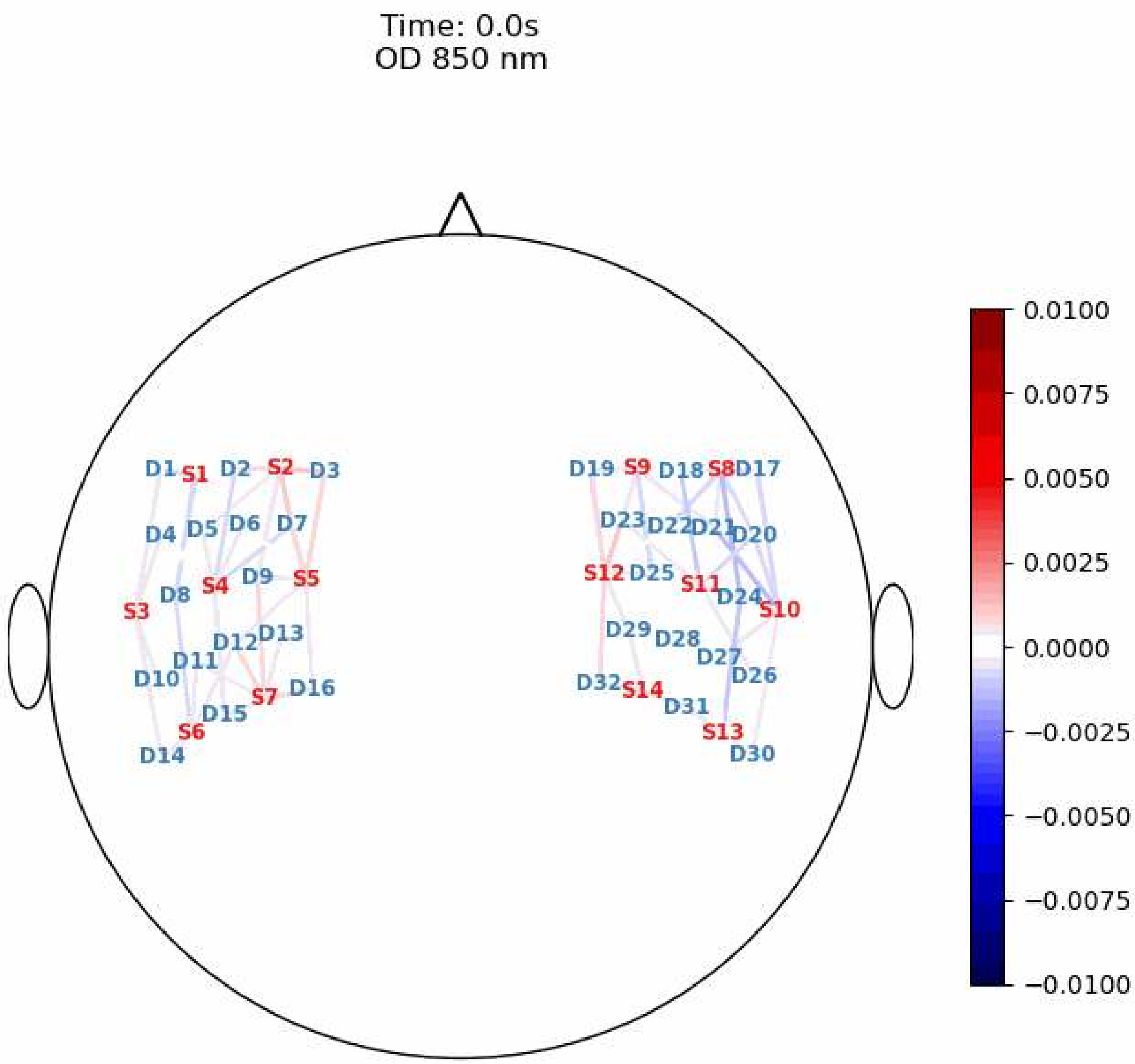

Image Space

Single-View Animations of Activitations on the Brain

The function `cedalion.vis.anatomy.image_recon_view` allows to create an animated gif of image-space concentration changes projected on the brain.

[22]:
```
filename_view = 'image_recon_view'

# image_recon_view expects input with dimensions
# ("vertex", "chromo", "time") (in that order)
X_ts = img.sel(trial_type="FTapping/Right").rename({"reltime": "time"})
X_ts = X_ts.transpose("vertex", "chromo", "time")

scl = np.percentile(np.abs(X_ts.sel(chromo='HbO')).pint.dequantify(), 99)
clim = (-scl,scl)

cedalion.vis.anatomy.image_recon_view(
    X_ts,  # time series data; can be 2D (static) or 3D (dynamic)
    head_ijk,
    cmap='seismic',
    clim=clim,
    view_type='hbo_brain',
    view_position='left',
    title_str='HbO / µM',
    filename=filename_view,
    SAVE=True,
    time_range=(-5,30,0.5)*units.s,
    fps=6,
    geo3d_plot = geo3d_plot,
    wdw_size = (1024, 768)
)
```

[23]:
```
display_image(f"{filename_view}.gif")
```

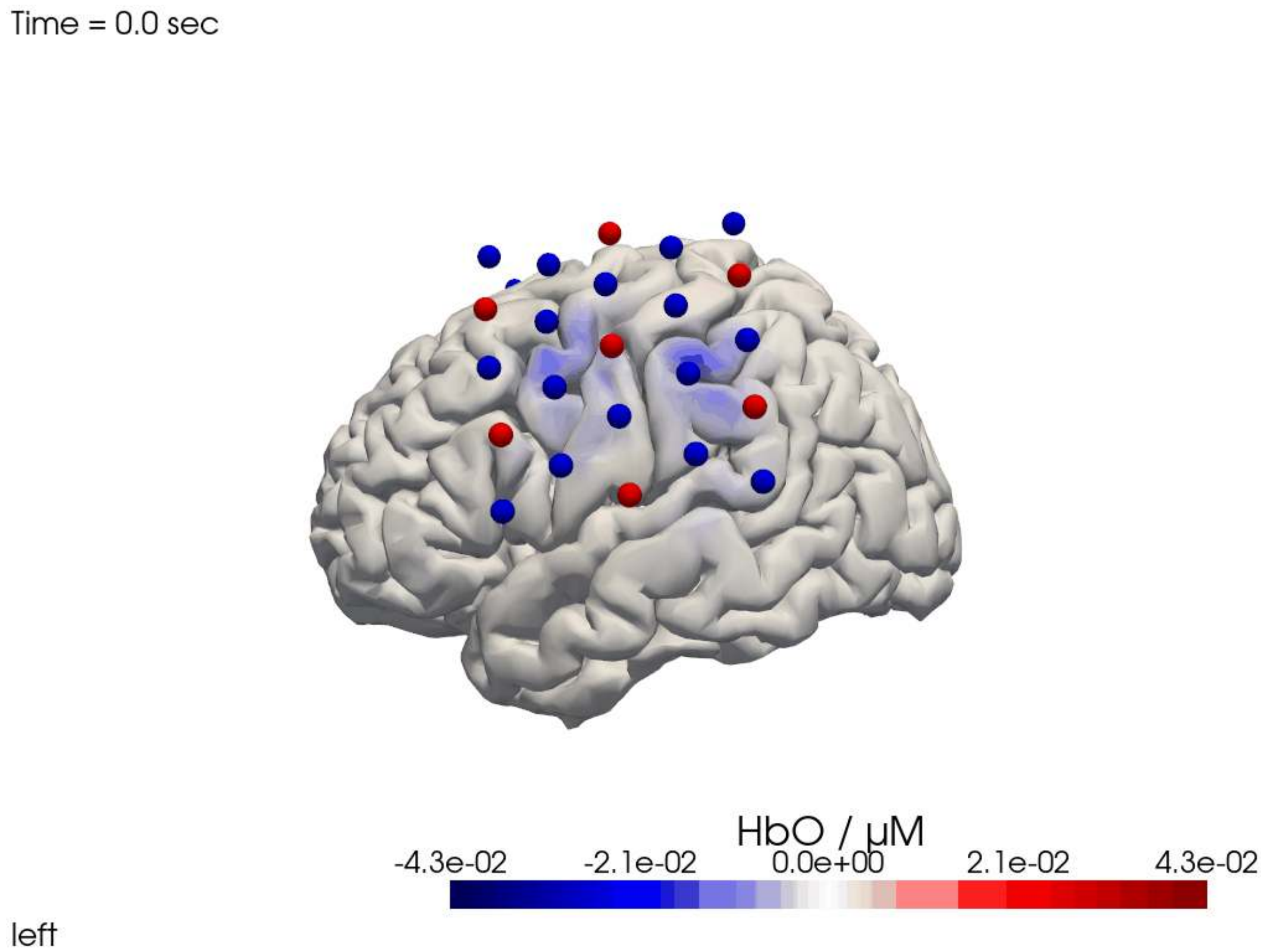

Alternatively, we can just select a single time point and plot activity as a still image at that time. Note the different file suffix (.png).

[24]:
```
# selects the nearest time sample at t=4s in X_ts
# note: sel does not accept quantified units
X_ts_plot = X_ts.sel(time=4, method="nearest")

filename_view = 'image_recon_view_still'

cedalion.vis.anatomy.image_recon_view(
    X_ts_plot,  # time series data; can be 2D (static) or 3D (dynamic)
    head_ijk,
    cmap='seismic',
    clim=clim,
    view_type='hbo_brain',
    view_position='left',
    title_str='HbO / µM',
    filename=filename_view,
    SAVE=True,
    time_range=(-5,30,0.5)*units.s,
    fps=6,
    geo3d_plot = geo3d_plot,
    wdw_size = (1024, 768)
```

```
)
```

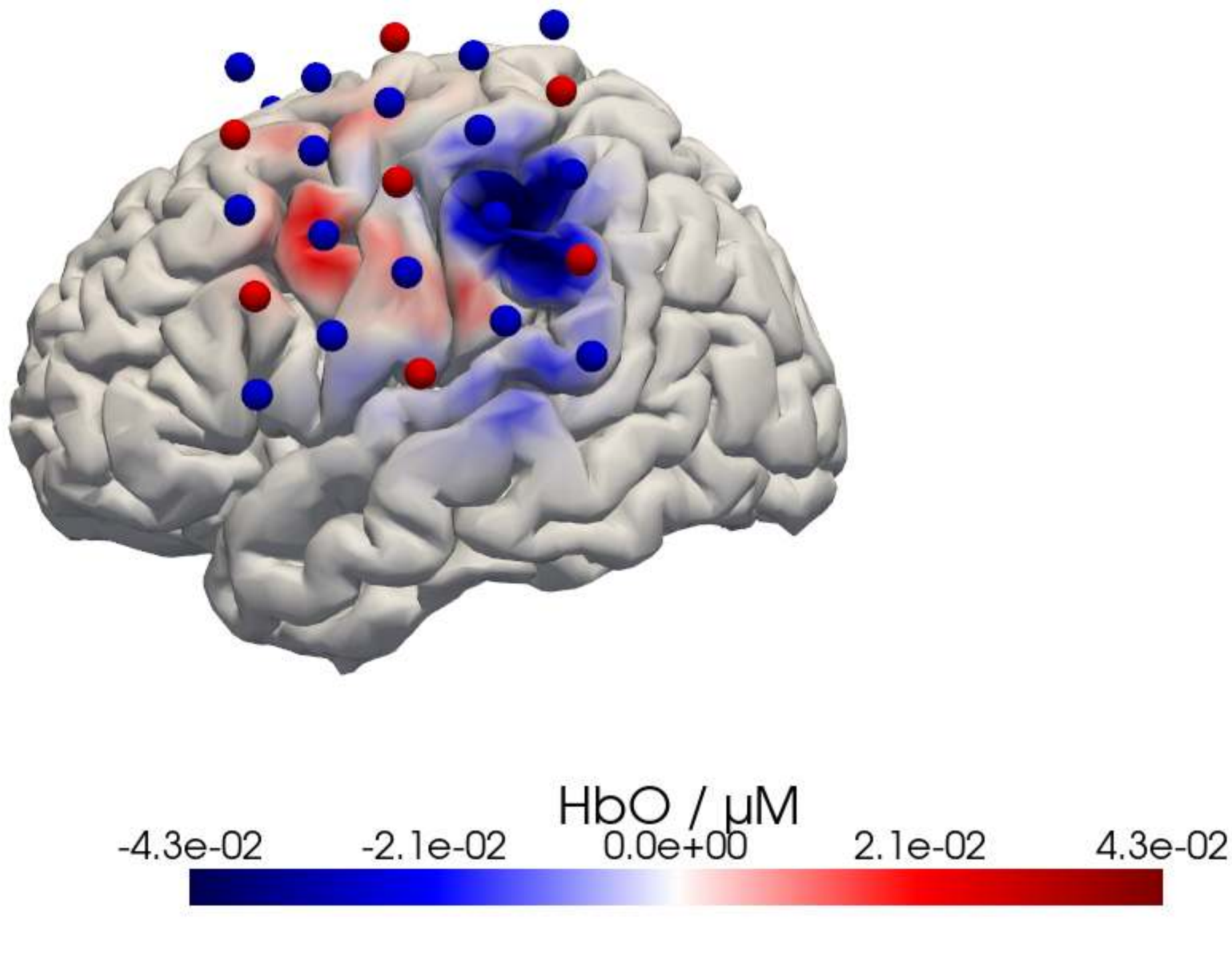

[25]:
```
display_image(f"{filename_view}.png")
```

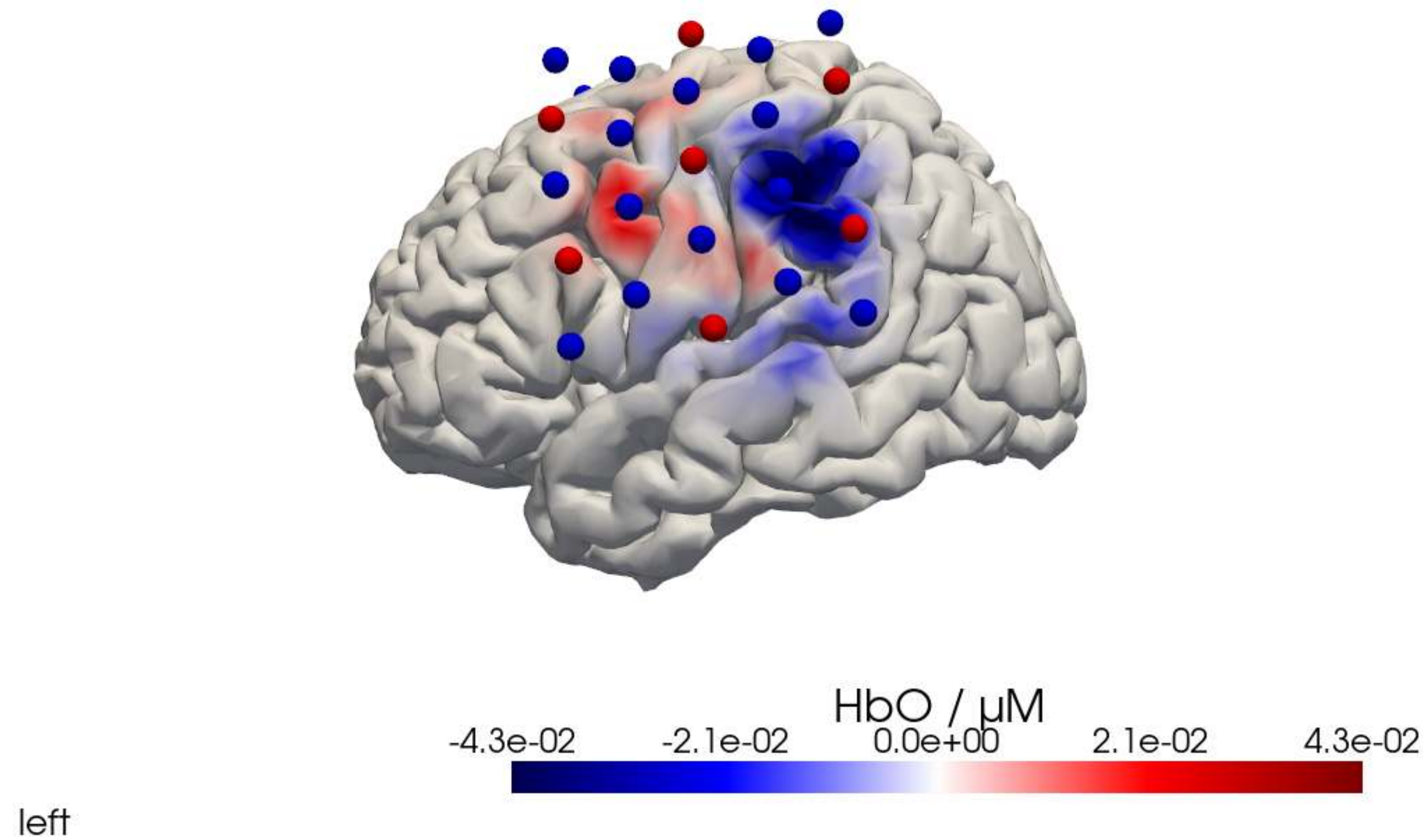

Multi-View Animations of Activitations on the Brain

The function `cedalion.vis.anatomy.image_recon_multi_view` shows the activity on the brain from all angles as still image or animated across time:

[26]:
```
filename_multiview = 'image_recon_multiview'

# prepare data
X_ts = img.sel(trial_type="FTapping/Right").rename({"reltime": "time"})
X_ts = X_ts.transpose("vertex", "chromo", "time")

scl = np.percentile(np.abs(X_ts.sel(chromo='HbO')).pint.dequantify(), 99)
clim = (-scl,scl)

cedalion.vis.anatomy.image_recon_multi_view(
    X_ts,  # time series data; can be 2D (static) or 3D (dynamic)
    head_ijk,
    cmap='seismic',
    clim=clim,
    view_type='hbo_brain',
    title_str='HbO / µM',
    filename=filename_multiview,
    SAVE=True,
```

```
    time_range=(-5,30,0.5)*units.s,
    fps=6,
    geo3d_plot = None, #  geo3d_plot
    wdw_size = (1024, 768)
)
```

[27]:
```
display_image(f"{filename_multiview}.gif")
```

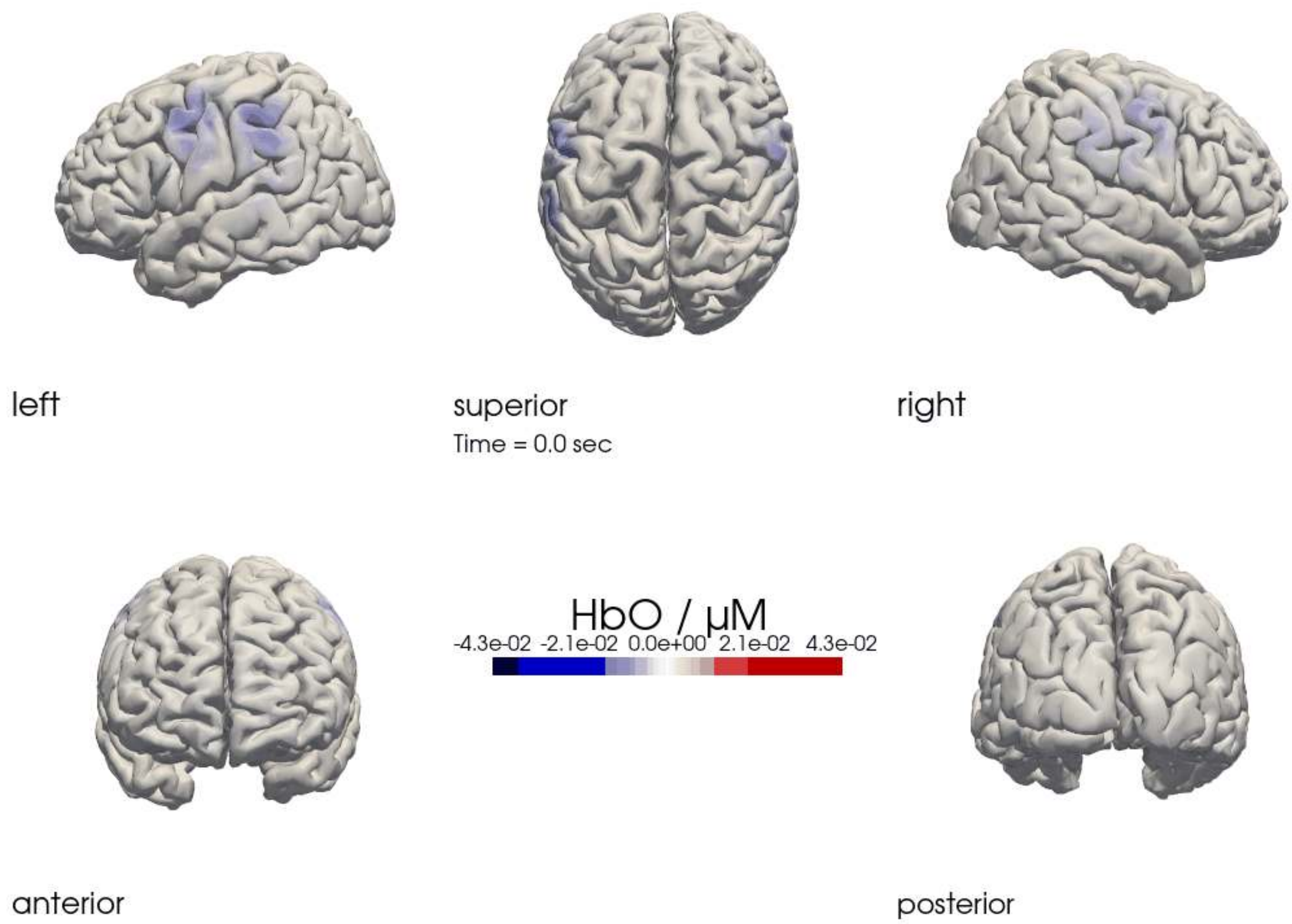

Multi-View Animations of Activitations on the Scalp

This gives us activity on the scalp after recon from all angles as still image or across time

[28]:
```
filename_multiview_scalp = 'image_recon_multiview_scalp'

# prepare data
X_ts = img.sel(trial_type="FTapping/Right").rename({"reltime": "time"})
X_ts = X_ts.transpose("vertex", "chromo", "time")

scl = np.percentile(np.abs(X_ts.sel(chromo='HbO')).pint.dequantify(), 99)
clim = (-scl,scl)

cedalion.vis.anatomy.image_recon_multi_view(
    X_ts,  # time series data; can be 2D (static) or 3D (dynamic)
```

```
    head_ijk,
    cmap='seismic',
    clim=clim,
    view_type='hbo_scalp',
    title_str='HbO / uM',
    filename=filename_multiview_scalp,
    SAVE=True,
    time_range=(-5,30,0.5)*units.s,
    fps=6,
    geo3d_plot = geo3d_plot,
    wdw_size = (1024, 768)
)
```

[29]:
```
display_image(f"{filename_multiview_scalp}.gif")
```

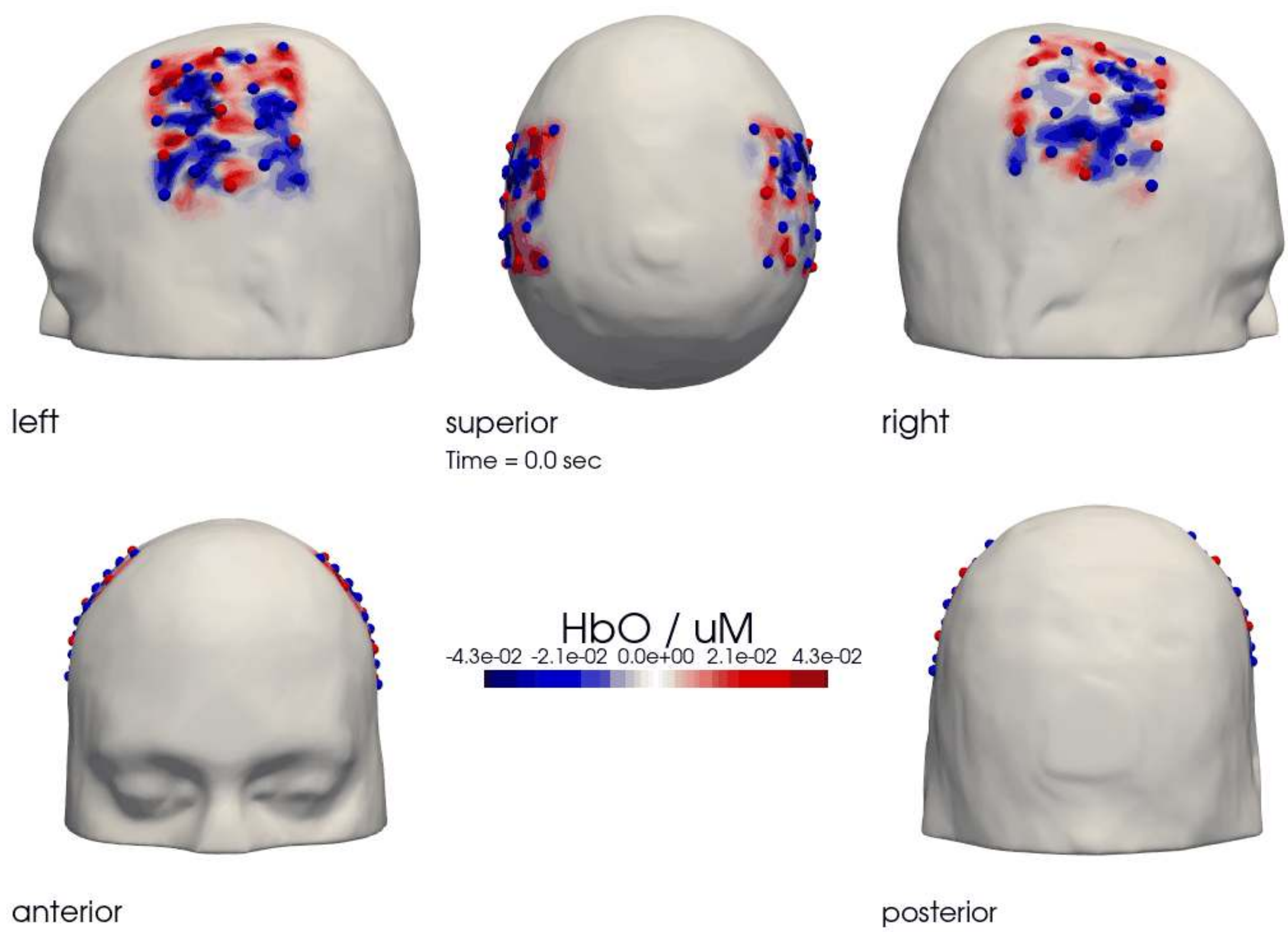

Parcel Space

The Schaefer Atlas [?] as implemented in Cedalion provides nearly 600 labels for different regions of the brain. Each vertex of the brain surface has its correspondng parcel label assigned as a coordinate.

[30]:
```
head_ijk.brain.vertices
```

[30]:
```
<xarray.DataArray (label: 25000, ijk: 3)> Size: 600kB
[] 82.52 19.84 68.31 83.52 20.32 65.89 … 96.28 105.3 86.73 127.6 34.46 60.38
Coordinates:
  * label            (label) int64 200kB 0 1 2 3 4 … 24996 24997 24998 24999
  * fsaverage_vertex (label) int64 200kB 42 61 75 89 … 333018 333020 333066
  * parcel           (label) object 200kB 'VisCent_ExStr_8_LH' … 'VisCent_…
  * mni152_r         (label) float64 200kB -6.792 -5.846 -10.87 … 6.633 37.95
  * mni152_a         (label) float64 200kB -104.8 -104.3 … -19.16 -89.49
  * mni152_s         (label) float64 200kB -1.408 -3.845 … 15.99 -10.04
Dimensions without coordinates: ijk
```

To obtain the average hemodynamic response in a parcel, the baseline-subtraced concentration changes of all vertices in a parcel are averaged. As baseline the first sample is used.

[31]:
```python
# only consider brain vertices in the following.
img_brain = img.sel(vertex=img.is_brain)

# use the first sample along the reltime dimension as baseline
baseline =  img_brain.isel(reltime=0)
img_brain_blsubtracted = img_brain - baseline

display(img_brain_blsubtracted.rename("img_brain_blsubtracted"))
```

```
<xarray.DataArray 'img_brain_blsubtracted' (chromo: 2, vertex: 25000,
                                             trial_type: 2, reltime: 154)> Size: 123MB
[µM] 0.0 1.158e-14 2.29e-14 3.571e-14 … -3.329e-12 -3.24e-12 -3.051e-12
Coordinates:
  * chromo      (chromo) <U3 24B 'HbO' 'HbR'
    parcel      (vertex) object 200kB 'VisCent_ExStr_8_LH' … 'VisCent_ExStr…
    is_brain    (vertex) bool 25kB True True True True … True True True True
  * reltime     (reltime) float64 1kB -5.038 -4.809 -4.58 … 29.54 29.77 30.0
  * trial_type  (trial_type) object 16B 'FTapping/Left' 'FTapping/Right'
Dimensions without coordinates: vertex
```

Using parcel labels, vertices belonging to the same brain region can be easily grouped together with the `DataArray.groupby` function. On these vertex groups many reduction methods can be executed. He we use the function `mean()` to average over vertices.

Note how the time series dimension changed from `vertex` to `parcel`:

[32]:
```python
# average over parcels
avg_HbO = img_brain_blsubtracted.sel(chromo="HbO").groupby('parcel').mean()
avg_HbR = img_brain_blsubtracted.sel(chromo="HbR").groupby('parcel').mean()

display(avg_HbO.rename("avg_HbO"))
```

```
<xarray.DataArray 'avg_HbO' (parcel: 601, trial_type: 2, reltime: 154)> Size: 1MB
[µM] 0.0 4.521e-11 1.252e-10 2.449e-10 … -1.266e-13 -1.245e-13 -1.249e-13
Coordinates:
    chromo      <U3 12B 'HbO'
  * reltime     (reltime) float64 1kB -5.038 -4.809 -4.58 … 29.54 29.77 30.0
  * trial_type  (trial_type) object 16B 'FTapping/Left' 'FTapping/Right'
  * parcel      (parcel) object 5kB 'Background+FreeSurfer_Defined_Medial_Wal…
```

The montage in this dataset covers only parts of the head. Consequently, many brain regions lack significant

signal coverage due to the absence of optodes.

To focus on relevant regions, a subset of parcels from the somatosensory and motor regions in both hemispheres is selected.

[33]:
```
selected_parcels = [
    "SomMotA_1_LH", "SomMotA_3_LH", "SomMotA_4_LH",
    "SomMotA_5_LH", "SomMotA_9_LH", "SomMotA_10_LH",

    "SomMotA_1_RH", "SomMotA_2_RH", "SomMotA_3_RH",
    "SomMotA_4_RH", "SomMotA_6_RH", "SomMotA_7_RH"
]
```

The following plot visualizes the montage and the selected parcels:

[34]:
```
# map parcel labels to colors
parcel_colors = {
    parcel: p.cm.jet(i / (len(selected_parcels) - 1))
    for i, parcel in enumerate(selected_parcels)
}

plt = pv.Plotter()

vbx.plot_surface(
    plt,
    head_ijk.scalp,
    color="w",
    opacity=.1
)

vbx.plot_surface(
    plt,
    head_ijk.brain,
    color=np.asarray(
        [
            parcel_colors.get(parcel, (0.8, 0.8, 0.8, 1.))
            for parcel in head_ijk.brain.vertices.parcel.values
        ]
    ),
    silhouette=True,
)

vbx.plot_labeled_points(plt, geo3d_plot)

plt.add_legend(labels=parcel_colors.items(), face="o", size=(0.3, 0.3))

vbx.camera_at_cog(plt, head_ijk.brain, rpos=(0, 0, 1), up=(0, 1, 0), fit_scene=True)
plt.show()
```

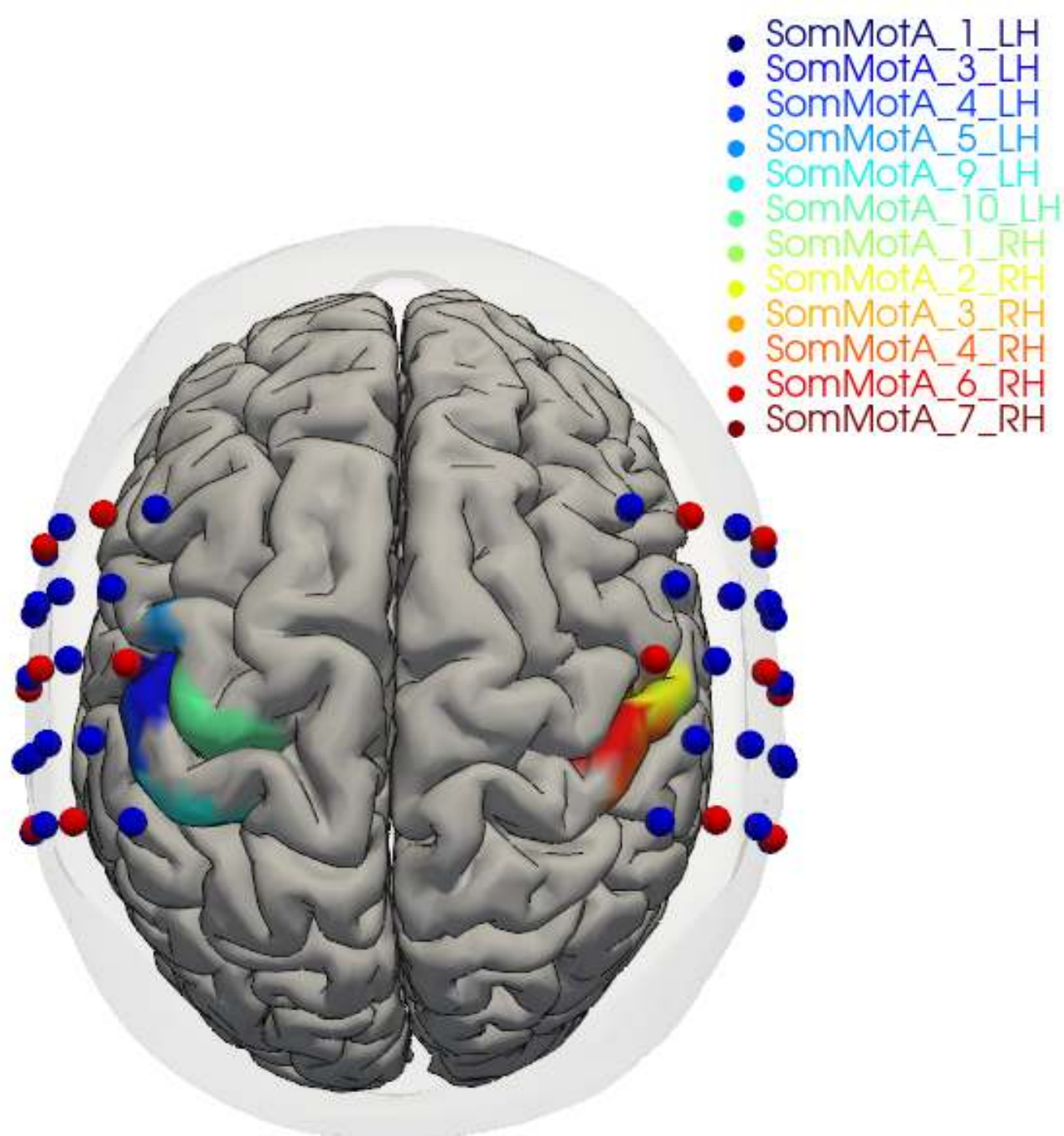

Plot averaged time traces in each parcel for the 'FTapping/Right' and 'FTapping/Left' conditions:

[35]:
```
f,ax = p.subplots(2,6, figsize=(20,5))
ylims = -0.02, 0.05
ax = ax.flatten()
for i_par, par in enumerate(selected_parcels):
    ax[i_par].plot(avg_HbO.sel(parcel = par, trial_type = "FTapping/Right").reltime,␣
 ↪avg_HbO.sel(parcel = par, trial_type = "FTapping/Right"), "r", lw=2, ls='-')
    ax[i_par].plot(avg_HbR.sel(parcel = par, trial_type = "FTapping/Right").reltime,␣
 ↪avg_HbR.sel(parcel = par, trial_type = "FTapping/Right"), "b", lw=2, ls='-')

    ax[i_par].grid(1)
    ax[i_par].set_title(par, color=parcel_colors[par])
    ax[i_par].set_ylim(*ylims)

    if i_par % 6 == 0:
        ax[i_par].set_ylabel("µM")
    if i_par >=6:
        ax[i_par].set_xlabel("$t_{rel} / s$")

p.suptitle("Parcellations: HbO: r | HbR: b | FTapping/Right", y=1)
p.tight_layout()
```

```
f,ax = p.subplots(2,6, figsize=(20,5))
ax = ax.flatten()
for i_par, par in enumerate(selected_parcels):
    ax[i_par].plot(avg_HbO.sel(parcel = par, trial_type = "FTapping/Left").reltime, avg_HbO.sel(parcel = par, trial_type = "FTapping/Left"), "r", lw=2, ls='-')
    ax[i_par].plot(avg_HbR.sel(parcel = par, trial_type = "FTapping/Left").reltime, avg_HbR.sel(parcel = par, trial_type = "FTapping/Left"), "b", lw=2, ls='-')

    ax[i_par].grid(1)
    ax[i_par].set_title(par, color=parcel_colors[par])
    ax[i_par].set_ylim(*ylims)

    if i_par % 6 == 0:
        ax[i_par].set_ylabel("µM")
    if i_par >=6:
        ax[i_par].set_xlabel("$t_{rel} / s$")

p.suptitle("Parcellations: HbO: r | HbR: b | FTapping/Left", y=1)
p.tight_layout()
```

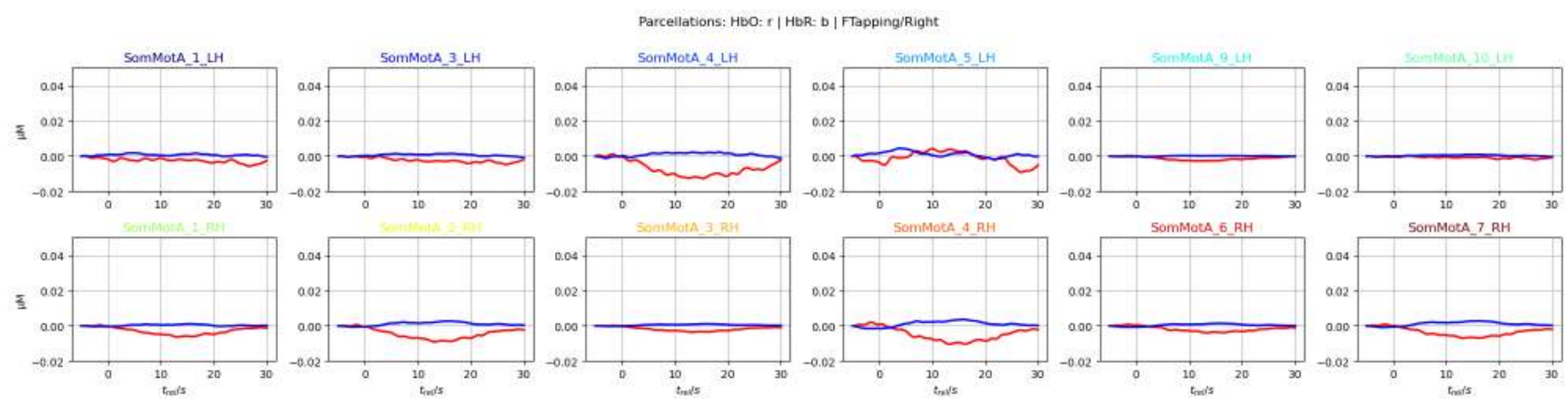

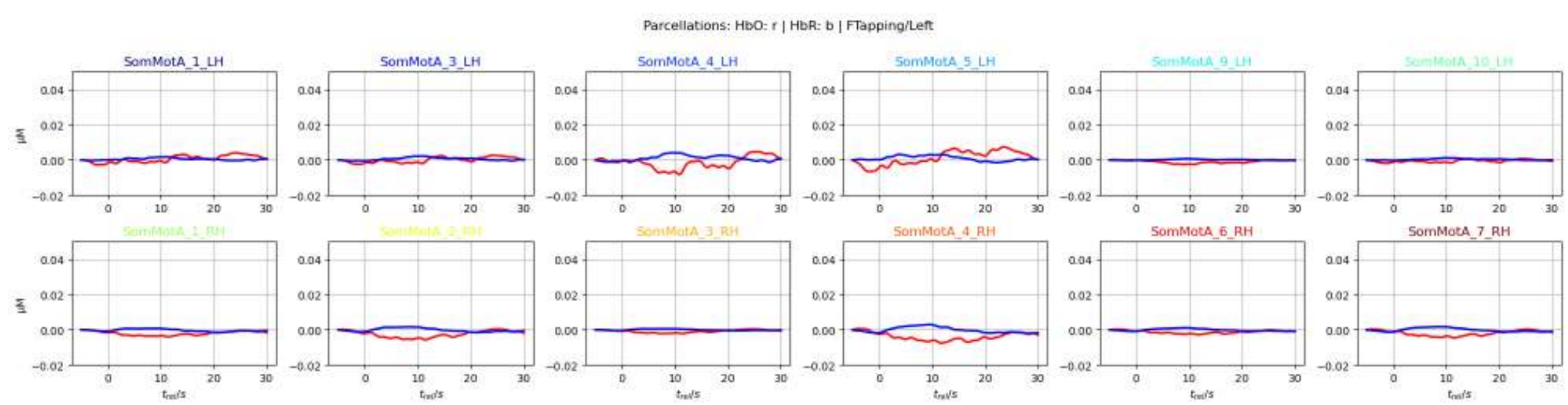

References

[36]:
```
cedalion.bib.dump_to_notebook()
```

```
<IPython.core.display.HTML object>
```

Next: 6 — Data-driven Analysis — unsupervised decomposition.

S6: Data-Driven (ML) Analysis

Learning objectives

In this notebook you will learn to:

- Apply canonical correlation analysis (CCA) to fNIRS data without a stimulus model
- Interpret components identified by data-driven decomposition
- Understand when data-driven methods are preferable to GLM approaches

This notebook has three sections:

- Multimodal-source-decomposition methods on simulated fNIRS-EEG data
- ICA Source Extraction
- Single Trial Classification

[1]:
```
# This cells setups the environment when executed in Google Colab.
try:
    import google.colab
    !curl -s https://raw.githubusercontent.com/ibs-lab/cedalion/dev/scripts/
 ↪colab_setup.py -o colab_setup.py
    # Select branch with --branch "branch name" (default is "dev")
    %run colab_setup.py
except ImportError:
    pass
```

[2]:
```
import cedalion
import cedalion.data
import cedalion.sigproc.quality as quality
import matplotlib.pyplot as plt
import numpy as np
import scipy as sp
import xarray as xr
from cedalion import units
import cedalion.sigproc.physio as physio
import cedalion.sigproc.motion as motion
from cedalion.sigdecomp.unimodal import ICA_ERBM
from cedalion.sigproc.frequency import sampling_rate
from cedalion.sim.datasets.synthetic_fnirs_eeg import (
    BimodalToyDataSimulation,
    standardize,
)

# Limit display to show 3 items at each edge, then "..."
np.set_printoptions(threshold=20, edgeitems=2)
xr.set_options(display_expand_data=False)

# To reaload packages and modules automatically.
%load_ext autoreload
%autoreload 2
```

Multimodal Source Decomposition Methods on Simulated fNIRS-EEG data

In this tutorial, we show how different multimodal source-decomposition methods can be used on an example toy fNIRS-EEG dataset to extract the underlying common (neural) sources. In particular, we cover Canonical Correlation Analysis (CCA), its regularized and temporally-embedded variants, and the multimodal Source Power Co-Modulation (mSPoC) algorithm from [Dähne, et.al. 2013]. All these methods can be found in the `cedalion.sigdecomp.multimodal` package.

[3]:
```
# Define helper plotting function for later use
def plot_source_comparisson(
    sx_power, sy, sx_power_model, sy_model, corrx, corry, corrxy, title
):
    """Plot comparison between original and reconstructed sources."""

    fig, ax = plt.subplots(2, 1, figsize=(12, 4), sharex=True)

    ax[0].plot(sx_power.time, sx_power, label="sx_power", color="red")
    ax[0].plot(
        sx_power_model.time,
        sx_power_model,
        label="sx_power reconstructed",
        color="blue",
    )
    ax[0].set_title("Sx | Correlation: {:.3f}".format(corrx))
    ax[0].set_ylabel("Amplitude")
    ax[0].legend()
    ax[0].grid()

    ax[1].plot(sy.time, sy, label="sy", color="green")
    ax[1].plot(sy_model.time, sy_model, label="sy reconstructed", color="orange")
    ax[1].set_title("Sy | Correlation: {:.3f}".format(corry))
    ax[1].set_xlabel("Time")
    ax[1].set_ylabel("Amplitude")
    ax[1].legend()
    ax[1].grid()

    plt.suptitle(
        f"{title} Reconstructed Sources | Correlation: {corrxy:.3f}", fontsize=16
    )
    plt.tight_layout()
    plt.show()
```

Simulated Dataset

For the simulated data we follow a small extension of the approach presented in [Dähne et al. 2013] that can be found whithin Cedalion, inside the `sim.datasets.synthetic_fnirs_eeg` module. #### Brief toy data description: EEG (x) and fNIRS (y) recordings are generated from a pseudo-random linear mixing forward model. Sources s_x, s_y are split into background (independent between modalities) and target (co-modulate between modalities). Each EEG background source is generated from random oscillatory signals in a given frequency band multiplied by a slow-varying random amplitude-modulation function. The latter is the envelope of s_x, and it serves as an estimate for the source bandpower timecourse. fNIRS background sources are generated from slow-varying random amplitude-modulation functions, using the same approach as for the envelope of s_x. The target sources are built using the same technique, but this time the same envelope

used to modulate s_x is used as the fNIRS source s_y. The s_x target sources can be also time-lagged (dT > 0) with respect to the target source s_y to simulate physiological delays between modalities. Upon simulation, fNIRS sources and recordings are downsampled to a new sampling interval, a.k.a. epochs, `T_epoch`.

Random background mixing matrices A_x and A_y are built using normal distributions, while we use Gaussian Radial Basis Functions (RBF) plus white noise to generate the ones for target sources. The latter approach gives the spatial patterns a more realistic local structure, by using the same center for the RBF for A_x and A_y.

The SNR can be tuned with the `gamma` parameter, which regulates the relative strength between target source and background source contributions in channel space. The exact relationship between SNR [dB] and `gamma` is given by $20\ log_{10}(\gamma)$.

The EEG recordings and their channel-wise powerband timecourses can be accesed via the `x` and `x_power` attributes. The former has the full sampling rate used during simulation (i.e. `rate`), while the latter has been downsampled so to be in the same time basis as the fNIRS recordings, `y`, and therefore directly comparable. The simulated target sources are contained in `sx_t` and `sy_t`, each of them with their own sampling rate, and the powerband timecourse of the former source can be obtained from `sx_power`.

Finally, the simulated data can be fed into a small preprocessing step, where each dataset and source is standardized (zero mean and unit variance) and split into train and test sets.

[4]:
```
# Configuration dictionary for the simulation
# (equivalently, can be loaded from a YAML file)
config_dict = {
    "Ny": 28,  # Number of channels
    "Nx": 32,
    "Ns_all": 100,  # Total number of sources
    "Ns_target": 1,  # Number of target sources
    "T": 300,  # Total simulation time (s)
    "T_epoch": 0.1,  # Length of epochs / Y sampling interval (s)
    "rate": 100,  # Sampling rate (Hz)
    "f_min": 8,  # Frequency band (Hz)
    "f_max": 12,
    "dT": 0,  # Time lag between target sources
    "invert_sy": False,  # Invert sy target source
    "gamma_e": 0.5,  # Noise strenght factor
    "gamma": 0.6,  # SNR parameter
    "ellx": 0.5,  # Width of RBF
    "elly": 0.2,
    "sigma_noise": 0.1,  # RBF white noise strength
}

# Create a simulation object with the configuration dictionary
sim = BimodalToyDataSimulation(config_dict, seed=137, mixing_type="structured")
SNR = 20 * np.log10(sim.args.gamma)  # Calculate SNR in dB
print(f"SNR: {SNR:.2f}")

# Plot target sources, channels, and mixing patterns (set xlim for better␣
↪visualization)
sim.plot_targets(xlim=(0, 30))
sim.plot_channels(N=2, xlim=(0, 30))
sim.plot_mixing_patterns()
```

```python
# Run small preprocessing step to standardize and split data into train and test sets
train_test_split = 0.8  # Proportion of data to use for training
preprocess_data_dict = sim.preprocess_data(train_test_split)
x_train, x_test = preprocess_data_dict["x_train"], preprocess_data_dict["x_test"]
x_power_train, x_power_test = (
    preprocess_data_dict["x_power_train"],
    preprocess_data_dict["x_power_test"],
)
y_train, y_test = preprocess_data_dict["y_train"], preprocess_data_dict["y_test"]
sx, sx_power, sy = (
    preprocess_data_dict["sx"],
    preprocess_data_dict["sx_power"],
    preprocess_data_dict["sy"],
)
```

Random seed set as 137

Simulating sources…

Finished

SNR: -4.44

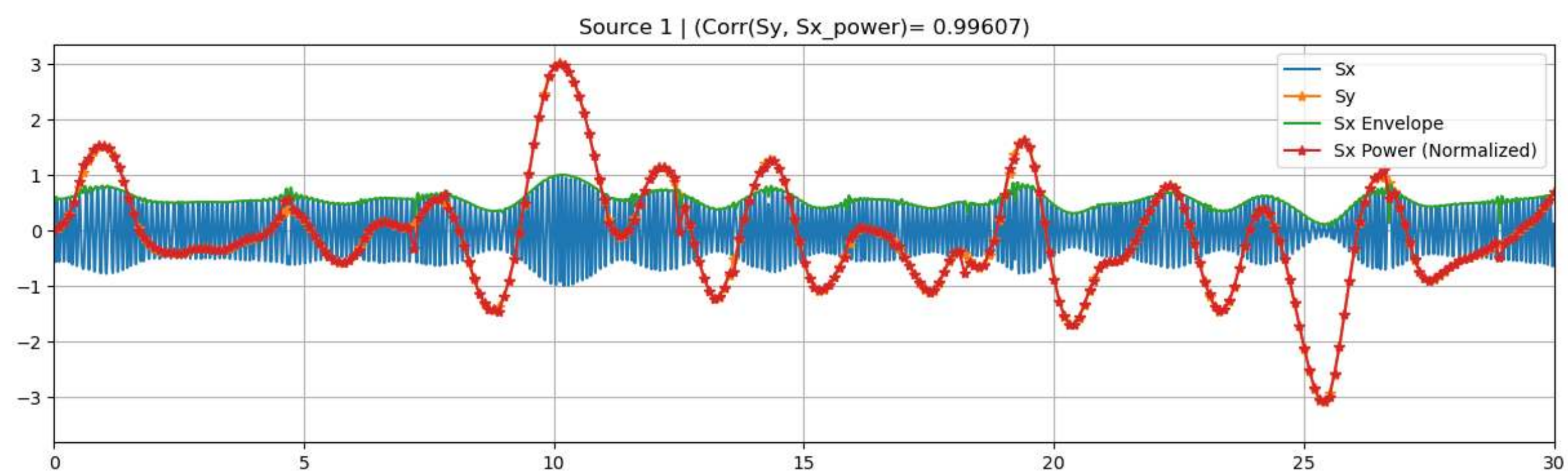

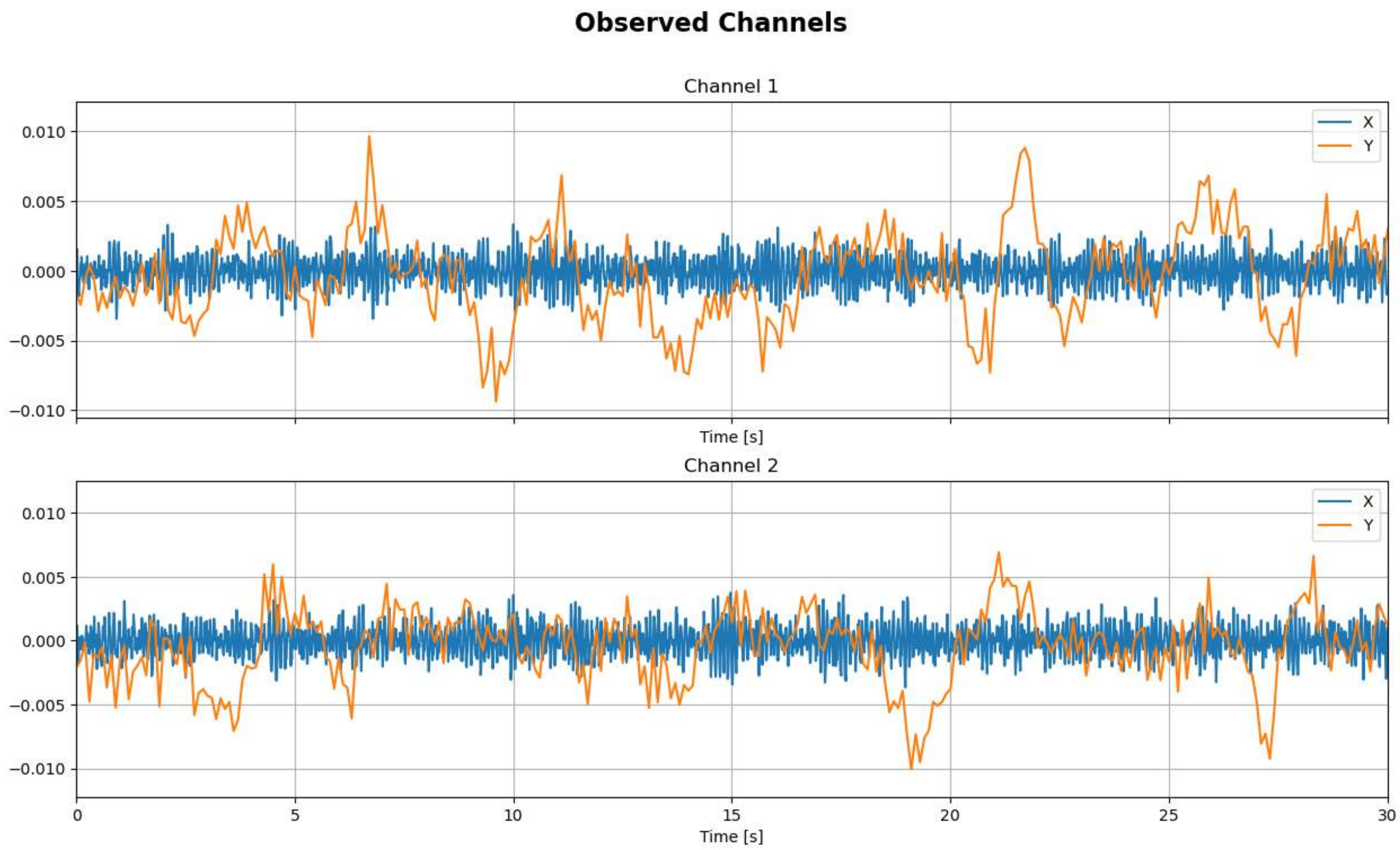

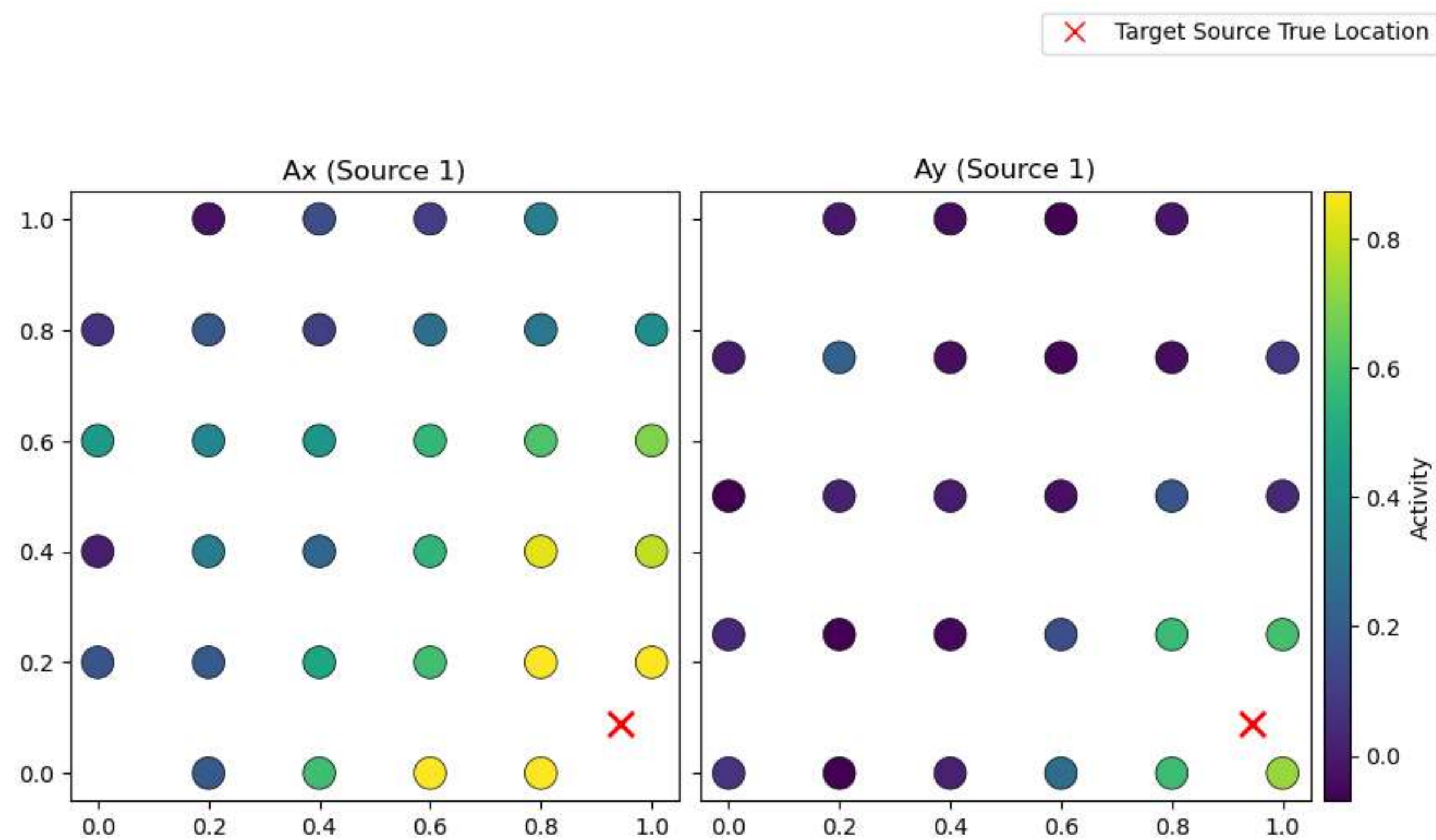

Regularized CCA

Canonical Correlation Analysis (CCA) looks for linear projections that maximize the correlation between two datasets X and Y. In certain scenarios, such as when dealing with high-dimensionality problems (more features than samples) or co-linearities between observations, or when looking for sparse or smooth solutions,

regularized CCA is expected to achieve a better performance on the source reconstruction task and help with interpretability. Some of the most well-established regularization/penalty terms are the L1-norm, leading to Sparse CCA, the L2-norm, leading to Ridge CCA, and their combination, known as ElasticNet CCA.

All these methods can be found as classes in the `cca_models` module inside the multimodal package: `CCA`, `SparseCCA`, `RidgeCCA`, and `ElasticNetCCA`. The latter contains the first three models as particular cases, so each of them can be also instantiated by picking specific values of the regularization parameters in the ElasticNetCCA class. Its implementation is based on [Parkhomenki et al. 2009].

We now show example use cases of these classes utilizing the toy-model fNIRS-EEG data simulated above. To this end, we will use the bandpower timecourse of x as one of the inputs, and the timecourse of y as the second one, both sharing the same sampling rate. The reason behind this choice is that, from a neuroscientific perspective, we expect these quantities to co-modulate, rather than their directy timecourses.

[5]:
```
from cedalion.sigdecomp.multimodal.cca import (
    CCA,
    ElasticNetCCA,
    RidgeCCA,
    SparseCCA,
    StructuredSparseCCA,
)
```

CCA

We have four optional parameters when initializing `CCA`: `N_components` is the number of components to extract. If `None`, the number of components is set to the minimum number of features between modalities. `max_iter` refers to the maximum number of iterations for the algorithm and `tol` regulates the tolerance for convergence by checking the norm of the difference between successive vector solutions during iteration. `scale` (bool) determines whether to scale the data during normalization to unit variance or just center it.

[6]:
```
# Initialize model
cca = CCA(N_components=1, max_iter=1000, tol=1e-6, scale=True)
```

We can now fit the model to some (x_train, y_train). Each of the input datasets need to be an `xr.DataArray` object with exactly two dimensions: one for features and the other one for samples. Both x and y datasets need to share the same sample dimension name and shape, while features can be different. The dimension naming can be specified with optional parameters. By default, the sample dimension is expected to be named 'time' and the feature dimensions 'channel'.

[7]:
```
# Fit model
cca.fit(
    x_power_train,
    y_train,
    sample_name="time",
    featureX_name="channel",
    featureY_name="channel",
)

display(cca.Wx)
```

```
<xarray.DataArray (channel: 32, CCA_X: 1)> Size: 256B
0.02794 0.1259 0.04475 0.174 0.03087 … 0.008922 0.02993 0.2225 -0.002854
Coordinates:
  * channel   (channel) <U3 384B 'X1' 'X2' 'X3' 'X4' … 'X29' 'X30' 'X31' 'X32'
  * CCA_X     (CCA_X) <U3 12B 'Sx1'
```

The learned filters, available via **cca.Wx**, and **cca.Wy**, can be used to transform a different pair (x_test, y_test). This test set must have the exact same feature dimension (name and shape) as the train data. The sample dimension should be the same between x_test and y_test, and its name must coincide with the one used during training. The number of samples, however, can be different between train and test sets.

[8]:
```
# Transform data
sx_power_cca, sy_cca = cca.transform(x_power_test, y_test)

print(f"Latent space dimensions: {cca.latent_featureX_name, cca.latent_featureY_name}")
sx_power_cca
```

```
Latent space dimensions: ('CCA_X', 'CCA_Y')
```

[8]:
```
<xarray.DataArray (time: 600, CCA_X: 1)> Size: 5kB
-0.2433 0.1107 0.5429 2.071 1.619 0.1281 … 2.217 1.67 2.113 1.802 1.129 0.6403
Coordinates:
  * time     (time) float64 5kB 240.1 240.2 240.3 240.4 … 299.8 299.9 300.0
  * CCA_X    (CCA_X) <U3 12B 'Sx1'
```

The **transform** method returns the "reconstructed sources", with the same sample dimension as the test input but with a new "latent_feature" dimension, generated automatically from the class/model name.

If the decomposition was performed successfully, the reconstructed sources should be close to the ground truth ones used in the linear forward model of the simulated data.

[9]:
```
# Normalize
sx_power_cca = standardize(sx_power_cca).T
sy_cca = standardize(sy_cca).T

# Calculate correlations
corrxy = np.corrcoef(sx_power_cca[0], sy_cca[0])[0, 1]
corrx = np.corrcoef(sx_power_cca[0], sx_power[0])[0, 1]
corry = np.corrcoef(sy_cca[0], sy[0])[0, 1]

# Plot results
plot_source_comparisson(
    sx_power[0], sy[0], sx_power_cca[0], sy_cca[0], corrx, corry, corrxy, title="CCA"
)
```

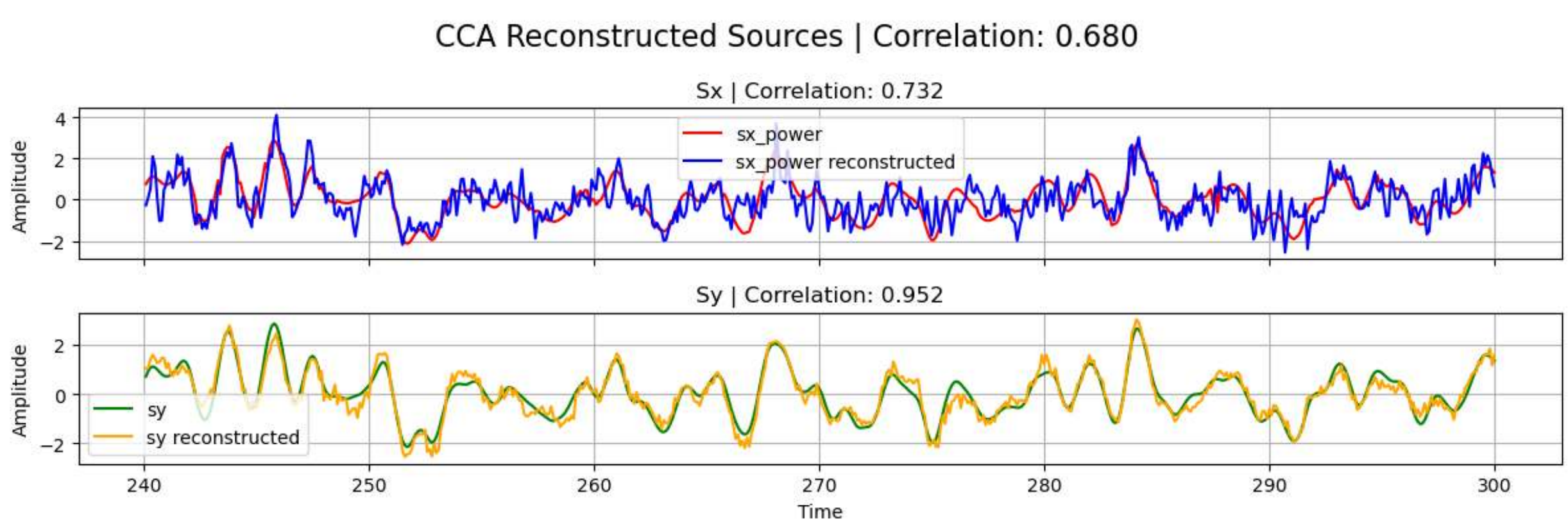

Sparse CCA, Ridge CCA, and ElasticNet CCA

`ElasticNetCCA` admits the same optional parameters as `CCA` on top of `l1_reg` and `l2_reg` to encode the regularization parameteres for the L1 and L2 penalty terms, respectively. These parameters take a list of the form $[\lambda_x, \lambda_y]$, containing the regularization parameters for W_x, and W_y, respectively. If, instead, a float λ is passed, then $\lambda_x = \lambda_y = \lambda$. L1 and L2 parameters need to be non-negative, with zero-values corresponding to no regularization. Additionally, `l1_reg` components must be smaller than 0.5, while `l2_reg` components are unbounded. `SparseCCA` is a sub-class of `ElasticNetCCA`, obtained by setting `l2_reg=0` on the latter. Analogously, `RidgeCCA` is equivalent to `ElasticNetCCA` with `l1_reg=0`. By choosing both regularizers to be zero, we recover the same `CCA` implementation we explored above.

With these regularized methods we can achieve better performance compared to standard CCA, even in lower SNR scenarios, as long as we make the right choice of hyperparamters. The latter gives us more flexibility, but requires a bit of extra work to achieve the optimal model's performance. For such a hyperparameter exploration, for instance, one can run a small random or grid search together with cross-validation in a validation set.

For the sake of simplicity, we won't follow that approach here, but rather show how the performance of `ElasticNetCCA` on the test set varies with different hyperparameter configurations. In particular, by looking at the `l1_reg=_l2_reg=0` in parameter space, we can see the increase in performance compared with CCA.

[10]:
```
# Define ranges for L1 and L2 parameters
l1_reg_list = np.linspace(0, 0.1, 25)
l2_reg_list = np.linspace(0, 2, 25)

# Initialize a list to store correlations regularization parameters
correlations_list = np.zeros((len(l1_reg_list), len(l1_reg_list), 3))

# Loop through parameters
for i, l1 in enumerate(l1_reg_list):
    for j, l2 in enumerate(l2_reg_list):
        # Initialize (default names for sample and feature dimensions
        # is 'time' and 'channel')
        elastic_cca = ElasticNetCCA(
            N_components=1,
            l1_reg=[l1, l1],  # L1 regularization parameter (same for both datasets)
            l2_reg=l2,
        )  # L2 regularization parameter (same for both datasets)

        # Fit model
        elastic_cca.fit(x_power_train, y_train)

        # Transform data
        sx_power_elastic_cca, sy_elastic_cca = elastic_cca.transform(
            x_power_test, y_test
        )

        # Normalize
        sx_power_elastic_cca = standardize(sx_power_elastic_cca).T[0]
        sy_elastic_cca = standardize(sy_elastic_cca).T[0]

        # Calculate correlations
        corrxy_elastic = np.corrcoef(sx_power_elastic_cca, sy_elastic_cca)[0, 1]
```

```python
        corrx_elastic = np.corrcoef(sx_power_elastic_cca, sx_power)[0, 1]
        corry_elastic = np.corrcoef(sy_elastic_cca, sy)[0, 1]

        correlations_list[i, j] = [corrxy_elastic, corrx_elastic, corry_elastic]

# Find (L1, L2) configuration with highest avarage correlation between corrx and corry
corr_avg = np.mean(correlations_list[:, :, 1:], axis=-1)
i, j = np.unravel_index(np.argmax(corr_avg), corr_avg.shape)
max_corrxy, max_corrx, max_corry = correlations_list[i, j]
best_l1_reg = l1_reg_list[i]
best_l2_reg = l2_reg_list[j]

# CCA correlation with L1=L2=0
cca_corr = correlations_list[0, 0, :]

print(
    f"Best L1 regularization: {best_l1_reg:.5f}, "
    f"Best L2 regularization: {best_l2_reg:.5f}"
)
print(
    f"Max correlation: {max_corrxy:.3f}, "
    f"Correlation X: {max_corrx:.3f}, "
    f"Correlation Y: {max_corry:.3f}"
)
print(
    f"CCA correlation (L1=L2=0): {cca_corr[0]:.3f}, "
    f"Correlation X: {cca_corr[1]:.3f}, "
    f"Correlation Y: {cca_corr[2]:.3f}"
)

# Plot the correlation heatmap
plt.figure(figsize=(10, 8))
plt.imshow(
    corr_avg,
    cmap="magma",
    aspect="auto",
    origin="lower",
    extent=[l2_reg_list[0], l2_reg_list[-1], l1_reg_list[0], l1_reg_list[-1]],
    interpolation="nearest",
)
plt.colorbar(label="Average Correlation")
plt.xlabel("L2 Regularization")
plt.ylabel("L1 Regularization")
plt.title("Correlation Heatmap for ElasticNet CCA")
plt.scatter(best_l2_reg, best_l1_reg, color="red", label="Best Parameters")
plt.legend()
plt.show()
```

```
Best L1 regularization: 0.06250, Best L2 regularization: 0.58333
Max correlation: 0.688, Correlation X: 0.738, Correlation Y: 0.956
CCA correlation (L1=L2=0): 0.680, Correlation X: 0.732, Correlation Y: 0.952
```

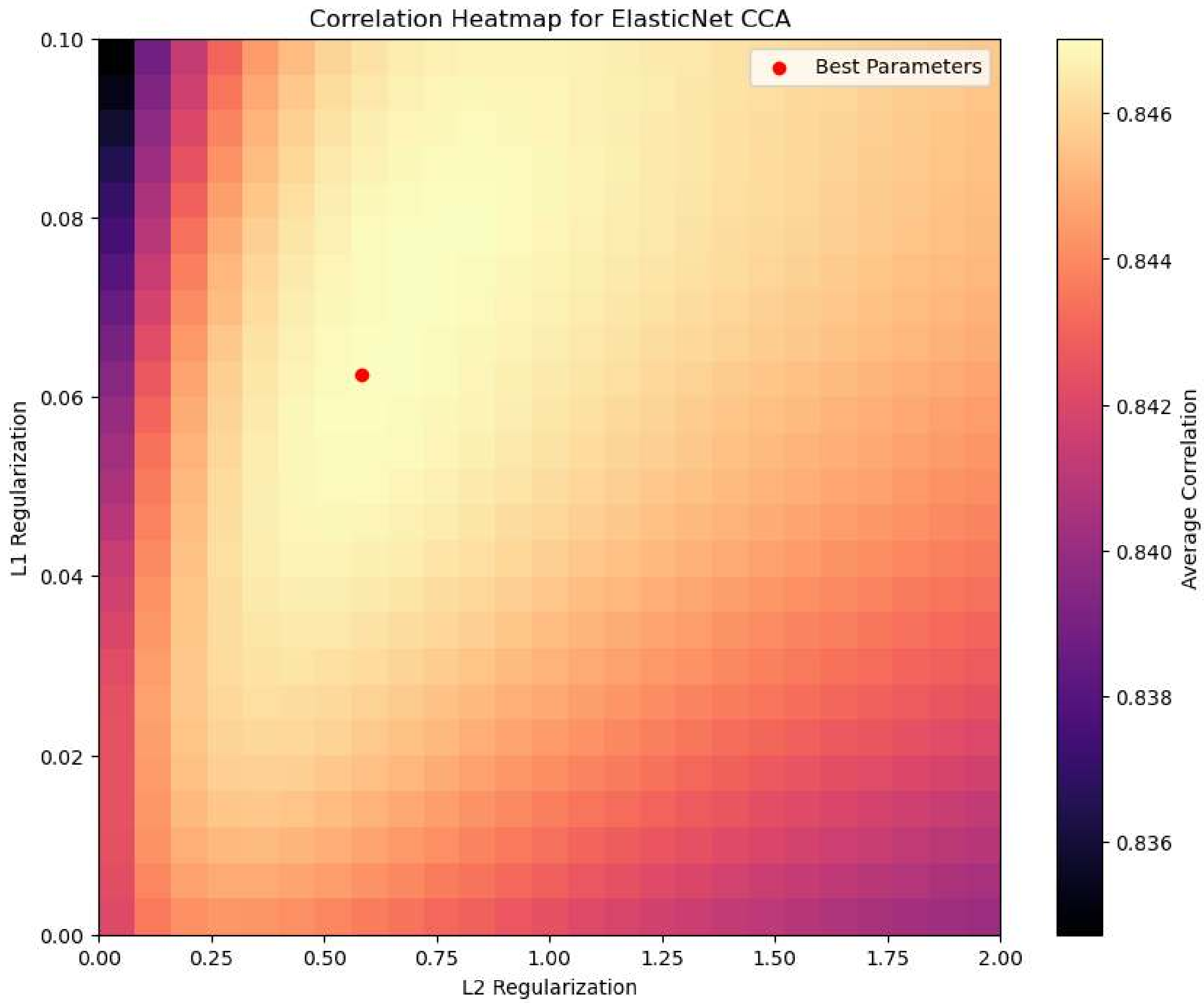

For the other regularization models, initializing, fitting and transforming work in the same way:

[11]:
```
# Initialize models (use optimal parameters found above)
elasticnet_cca = ElasticNetCCA(
    N_components=1,
    l1_reg=best_l1_reg,  # Use optimal L1, L2 found above
    l2_reg=best_l2_reg,
)

# Equals ElasticNetCCA with l1_reg=best_l1_reg and l2_reg=0
sparse_cca = SparseCCA(N_components=1, l1_reg=best_l1_reg)
# Equals ElasticNetCCA with l1_reg=0 and l2_reg=best_l2_reg
ridge_cca = RidgeCCA(N_components=1, l2_reg=best_l2_reg)

# Fit models
elasticnet_cca.fit(x_power_train, y_train)
sparse_cca.fit(x_power_train, y_train)
ridge_cca.fit(x_power_train, y_train)

# Transform data
```

```
sx_power_elastic, sy_elastic = elasticnet_cca.transform(x_power_test, y_test)
sx_power_sparse, sy_sparse = sparse_cca.transform(x_power_test, y_test)
sx_power_ridge, sy_ridge = ridge_cca.transform(x_power_test, y_test)

# Normalize
sx_power_elastic = standardize(sx_power_elastic).T[0]
sy_elastic = standardize(sy_elastic).T[0]

sx_power_sparse = standardize(sx_power_sparse).T[0]
sy_sparse = standardize(sy_sparse).T[0]

sx_power_ridge = standardize(sx_power_ridge).T[0]
sy_ridge = standardize(sy_ridge).T[0]


# Calculate correlations
corrxy_elastic = np.corrcoef(sx_power_elastic, sy_elastic)[0, 1]
corrx_elastic = np.corrcoef(sx_power_elastic, sx_power)[0, 1]
corry_elastic = np.corrcoef(sy_elastic, sy)[0, 1]

corrxy_sparse = np.corrcoef(sx_power_sparse, sy_sparse)[0, 1]
corrx_sparse = np.corrcoef(sx_power_sparse, sx_power)[0, 1]
corry_sparse = np.corrcoef(sy_sparse, sy)[0, 1]

corrxy_ridge = np.corrcoef(sx_power_ridge, sy_ridge)[0, 1]
corrx_ridge = np.corrcoef(sx_power_ridge, sx_power)[0, 1]
corry_ridge = np.corrcoef(sy_ridge, sy)[0, 1]

# Plot
plot_source_comparisson(
    sx_power[0],
    sy[0],
    sx_power_elastic,
    sy_elastic,
    corrx_elastic,
    corry_elastic,
    corrxy_elastic,
    title="ElasticNet CCA",
)
plot_source_comparisson(
    sx_power[0],
    sy[0],
    sx_power_sparse,
    sy_sparse,
    corrx_sparse,
    corry_sparse,
    corrxy_sparse,
    title="Sparse CCA",
)
plot_source_comparisson(
    sx_power[0],
```

```python
    sy[0],
    sx_power_ridge,
    sy_ridge,
    corrx_ridge,
    corry_ridge,
    corrxy_ridge,
    title="Ridge CCA",
)
```

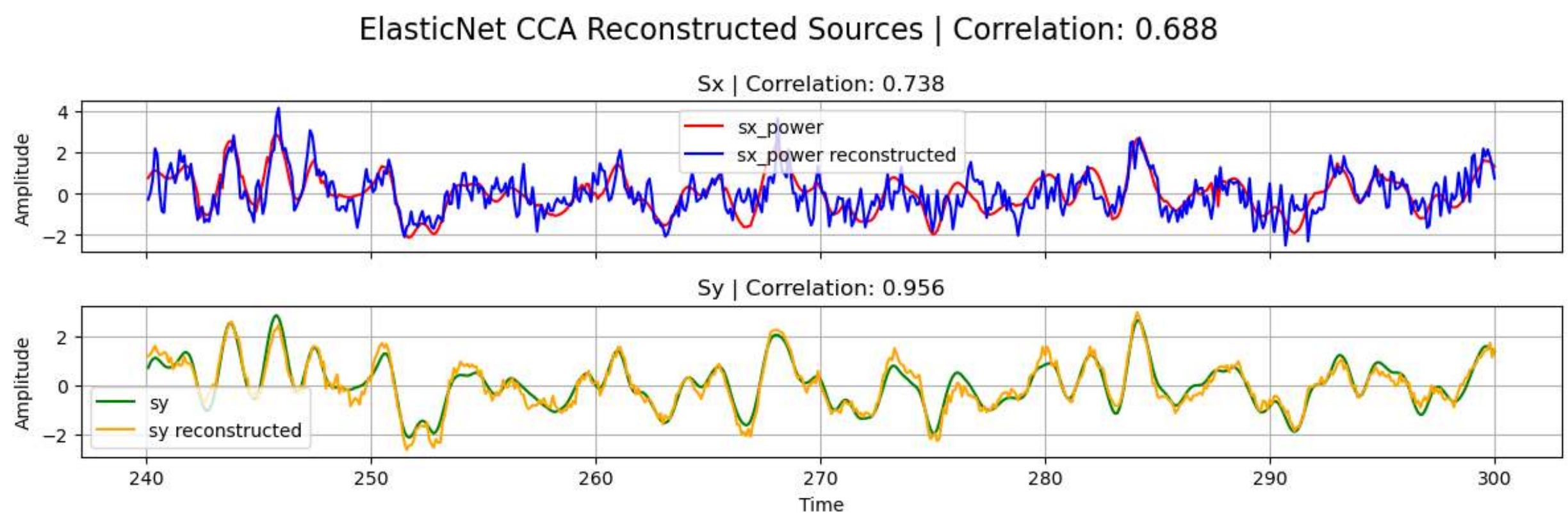

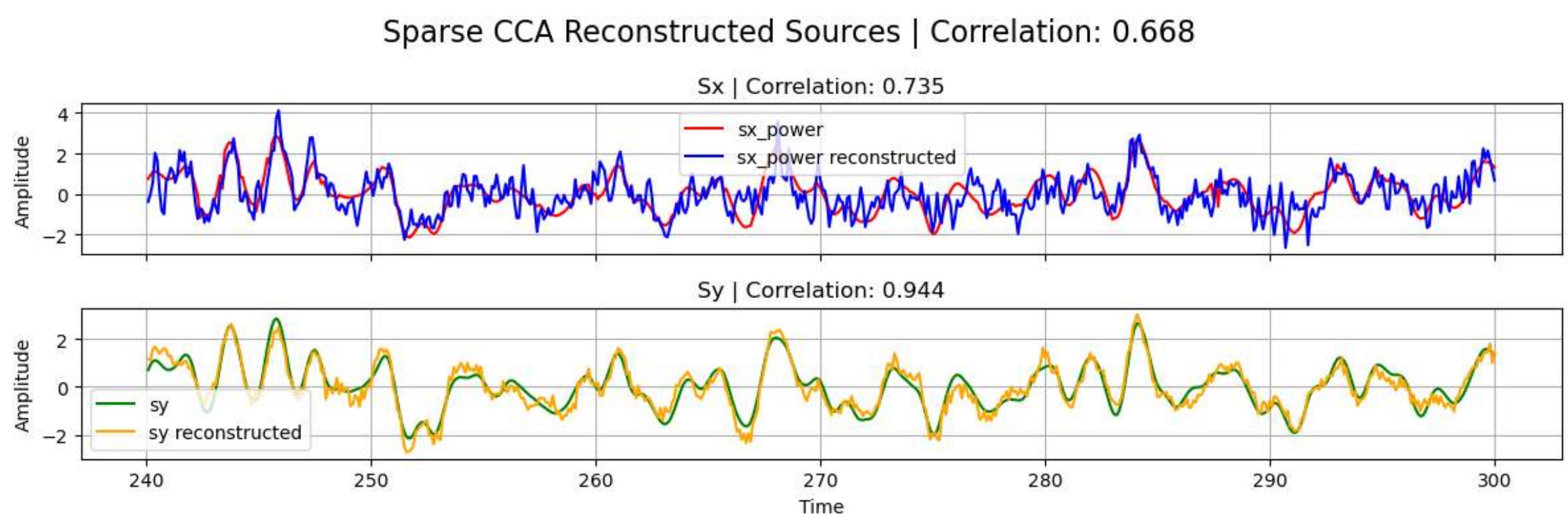

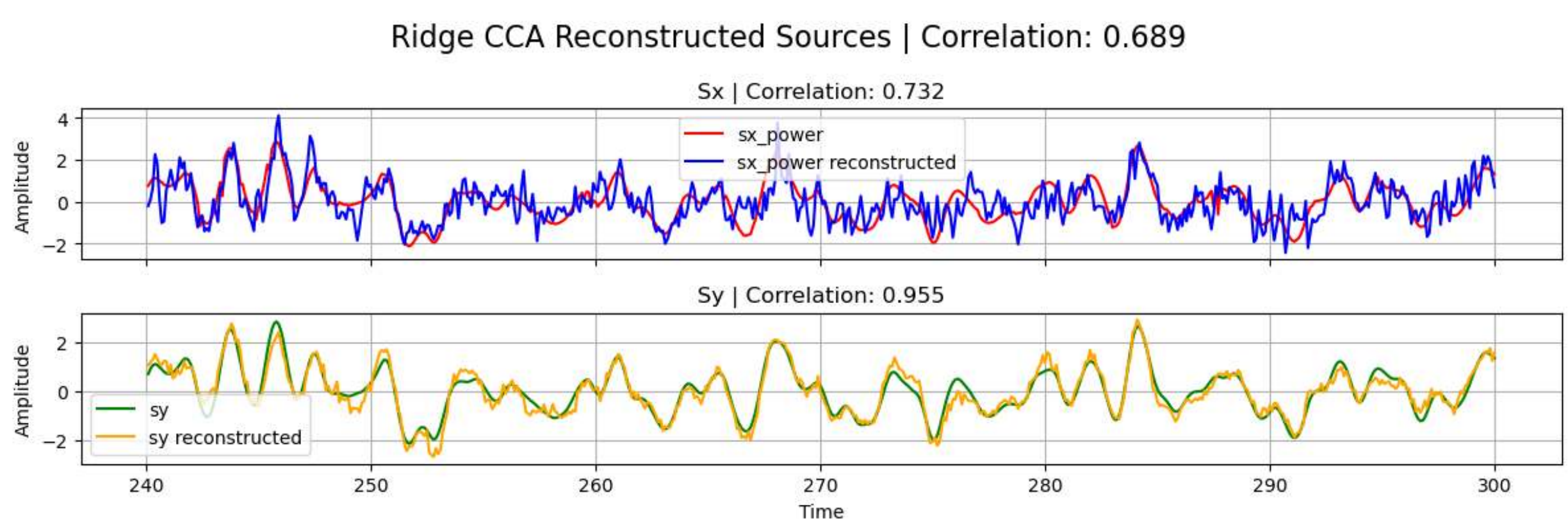

Structured Sparse CCA (ssCCA)

ElasticNet regularization can be modified to include contextual information about the local structure of the dataset, leading to structured CCA. This is particularly relevant in neuroimaging, where features (e.g. channels or brain regions) follow specific spatial distributions. One way of incorporating this prior information is to modify the L2-norm penalty term on the filters W via $||W||_2^2 \to W^T L W$ for some matrix L that captures the local dependencies among features. When this structure contraint is combined with an L1 penalization, the resulting method is called structured sparse CCA (ssCCA) and is implemented in Cedalion as the `StructuredSparseCCA` class, following [Chen et al. 2013].

This class admits the same parameters as `ElasticNetCCA` together with `Lx` and `Ly`, corresponding to structure matrices used for each dataset. The latter need to be square matrices of shapes (Nx, Nx) and (Ny, Ny), in terms of the number of features of each dataset. This time, the `l2_reg` parameter regulates the strength of the structured penalty term, and must be non-negative but otherwise unbounded.

One popular choice for such structure contraint is the Laplacian matrix, which is used as a graph representation of the dataset, in which features (channels) are the nodes, and (weighted) edges indicate which and how features are connected. We now show a simple example of a Laplacian matrix, build via an adjacency matrix that uses a binary nearest-neighbord strategy.

[12]:
```
# Helper function to build Laplacian matrix
def build_laplace(nodes, eps):
    """Builds Laplacian matrix of a graph.

    The nodes are the components of the 1D vector nodes,
    by giving unit weight to connected nodes only if they are close enough.
    The latter condition is determined by comparing the 2-norm between nodes and eps.
    """

    N = len(nodes)
    Adj = np.zeros([N, N])  # Adjacency matrix
    D = np.eye(N)  # Degree matrix

    for i, xi in enumerate(nodes):
        for j, xj in enumerate(nodes):
            if i == j:  # Skip diagonal entries
                continue
            are_close = np.linalg.norm(xi - xj) < eps
            Adj[i, j] = 1 if are_close else 0

        D[i, i] = np.sum(Adj[i])

    L = D - Adj  # Laplace matrix

    return L, Adj
```

[13]:
```
# Read channel positions from simulation
x_channels_pos = sim.x_montage.values
y_channels_pos = sim.y_montage.values

# Build Laplacian matrix from each montage
# (eps thresholds chosen by small experimentation)
Lx, Adjx = build_laplace(x_channels_pos, eps=sim.args.ellx)
```

```
Ly, Adjy = build_laplace(y_channels_pos, eps=sim.args.elly)

# Plot Adjacency matrices
plt.figure(figsize=(10, 5))
plt.subplot(1, 2, 1)
plt.imshow(Adjx, cmap="viridis", interpolation="nearest")
plt.title("Adjacency Matrix for X Montage")
plt.colorbar(label="Weight")
plt.subplot(1, 2, 2)
plt.imshow(Adjy, cmap="viridis", interpolation="nearest")
plt.title("Adjacency Matrix for Y Montage")
plt.colorbar(label="Weight")
plt.tight_layout()
```

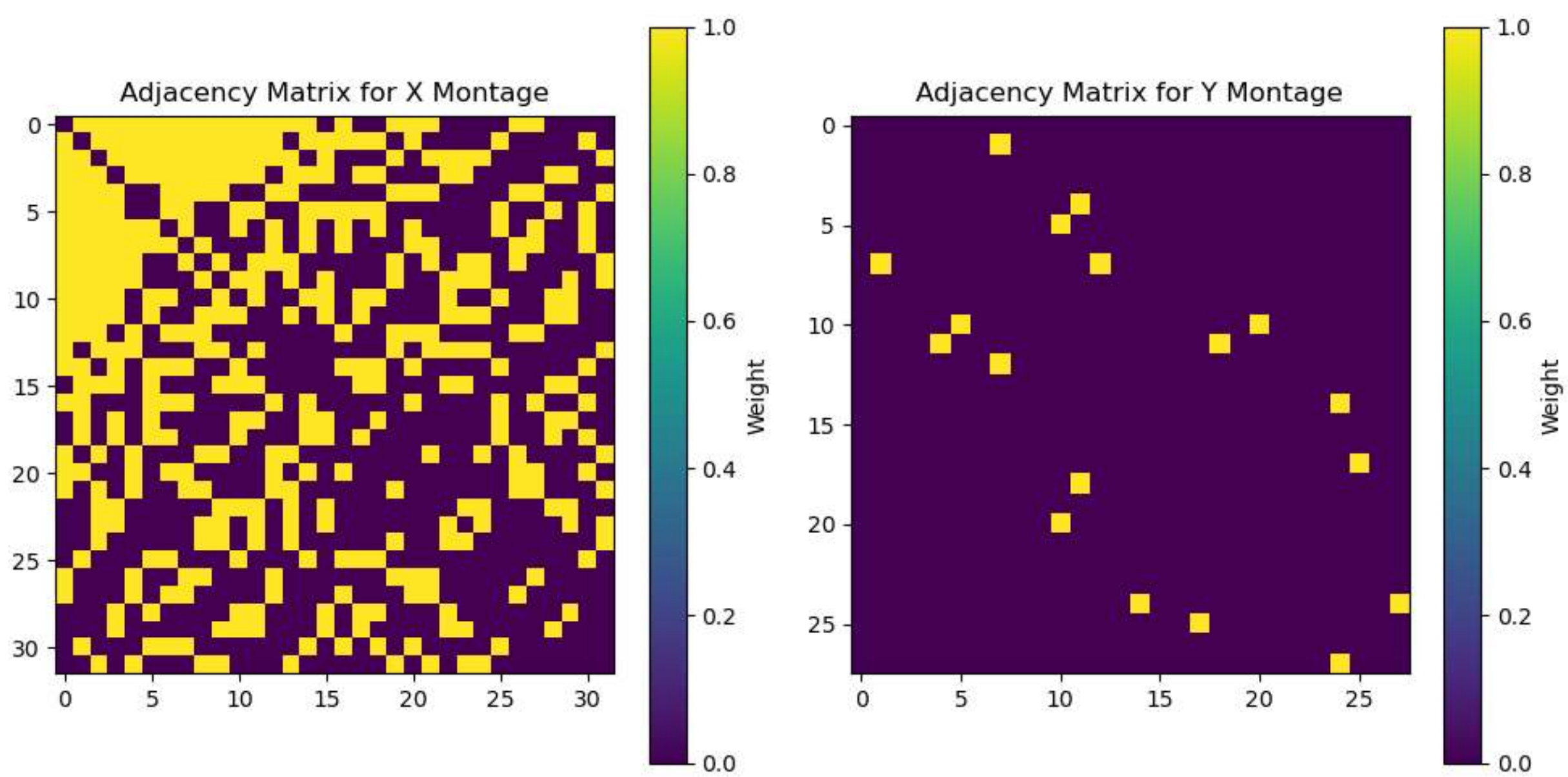

The choice of structure/Laplace matrix introduces yet another layer of complexity to the model, which needs further fine-tunning to achieve its optimal performance. Since making an exhaustive investigation is beyond the scope of this tutorial notebook, here we just chose the nearest-neighbor thresholds to coincide with the ground truth RBF width used in the simulated mixing matrices, and picked the same regularization parameters as for `ElasticNetCCA` above. Initialization, fitting, and transformation follows the same logic as before:

[14]:
```
sscca = StructuredSparseCCA(
    N_components=1,
    l1_reg=best_l1_reg,  # Use optimal L1, L2 found above
    l2_reg=best_l2_reg,
    Lx=Lx,  # Laplacian matrix for X montage
    Ly=Ly,  # Laplacian matrix for Y montage
)

# Fit model
sscca.fit(x_power_train, y_train)
```

```
# Transform data
sx_power_sscca, sy_sscca = sscca.transform(x_power_test, y_test)

# Normalize
sx_power_sscca = standardize(sx_power_sscca).T[0]
sy_sscca = standardize(sy_sscca).T[0]

# Calculate correlations
corrxy_sscca = np.corrcoef(sx_power_sscca, sy_sscca)[0, 1]
corrx_sscca = np.corrcoef(sx_power_sscca, sx_power)[0, 1]
corry_sscca = np.corrcoef(sy_sscca, sy)[0, 1]

# Plot
plot_source_comparisson(
    sx_power[0],
    sy[0],
    sx_power_sscca,
    sy_sscca,
    corrx_sscca,
    corry_sscca,
    corrxy_sscca,
    title="Structured Sparse CCA",
)
```

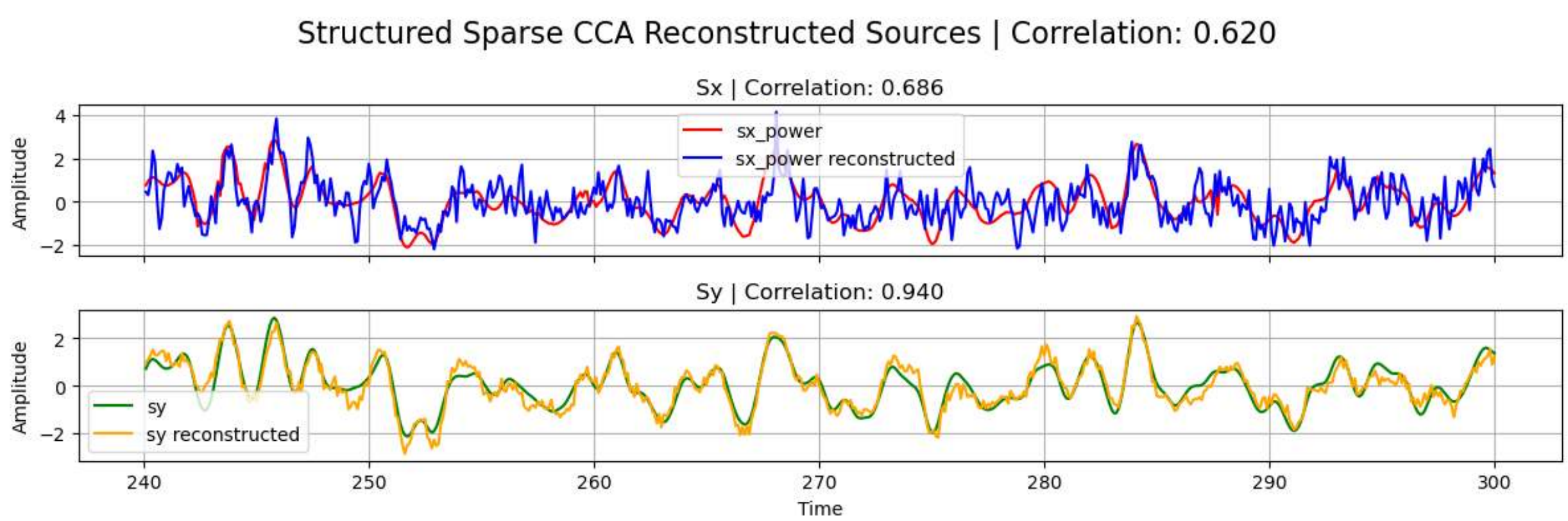

From the learned weights, `Wx` and `Wy`, one can estimate the spattial patterns `Ax` and `Ay` via a regression method [Haufe et al. 2014]. These quantities should be close to the original mixing matrices used in the linear forward model of the simulated data. Structured regularization is particularly promising for such a reconstruction task given the incorporation of spatial information during the decomposition:

[15]:
```
# Helper function to estimate spatial patterns from
# learned weights via regression approach
def compute_spatial_pattern_from_weight(
    X_xr, W_xr, sample_name="time", feature_name="channel"
):
    # Bring to standard order
    X_xr = X_xr.transpose(sample_name, feature_name)
```

```python
    W_xr = W_xr.transpose(feature_name, ...)

    # Work with numpy arrays from now on
    N = len(X_xr[sample_name])
    X = X_xr.data
    W = W_xr.data
    # Covariance matrix for X
    C = (X.T @ X) / (N - 1)
    # Covariance matrix for reconstructed sources
    Cs = W.T @ C @ W
    # Estimated spattial pattern
    A = C @ W @ sp.linalg.pinv(Cs)

    # Bring to DataArray format\
    A = xr.DataArray(A, dims=W_xr.dims, coords=W_xr.coords)

    return A


# Compute spatial patterns from learned weights for ssCCA
Ax_sscca = compute_spatial_pattern_from_weight(x_test, sscca.Wx)
Ay_sscca = compute_spatial_pattern_from_weight(y_test, sscca.Wy)

# Normalize
Ax_sscca /= Ax_sscca.max()
Ay_sscca /= Ay_sscca.max()

# Plot original mixing patterns
sim.plot_mixing_patterns(title="Original Mixing Patterns")
# Plot estimated spatial patterns
sim.plot_mixing_patterns(
    Ax=Ax_sscca.values,
    Ay=Ay_sscca.values,
    title="Reconstructed Spatial Patterns (ssCCA))",
)
```

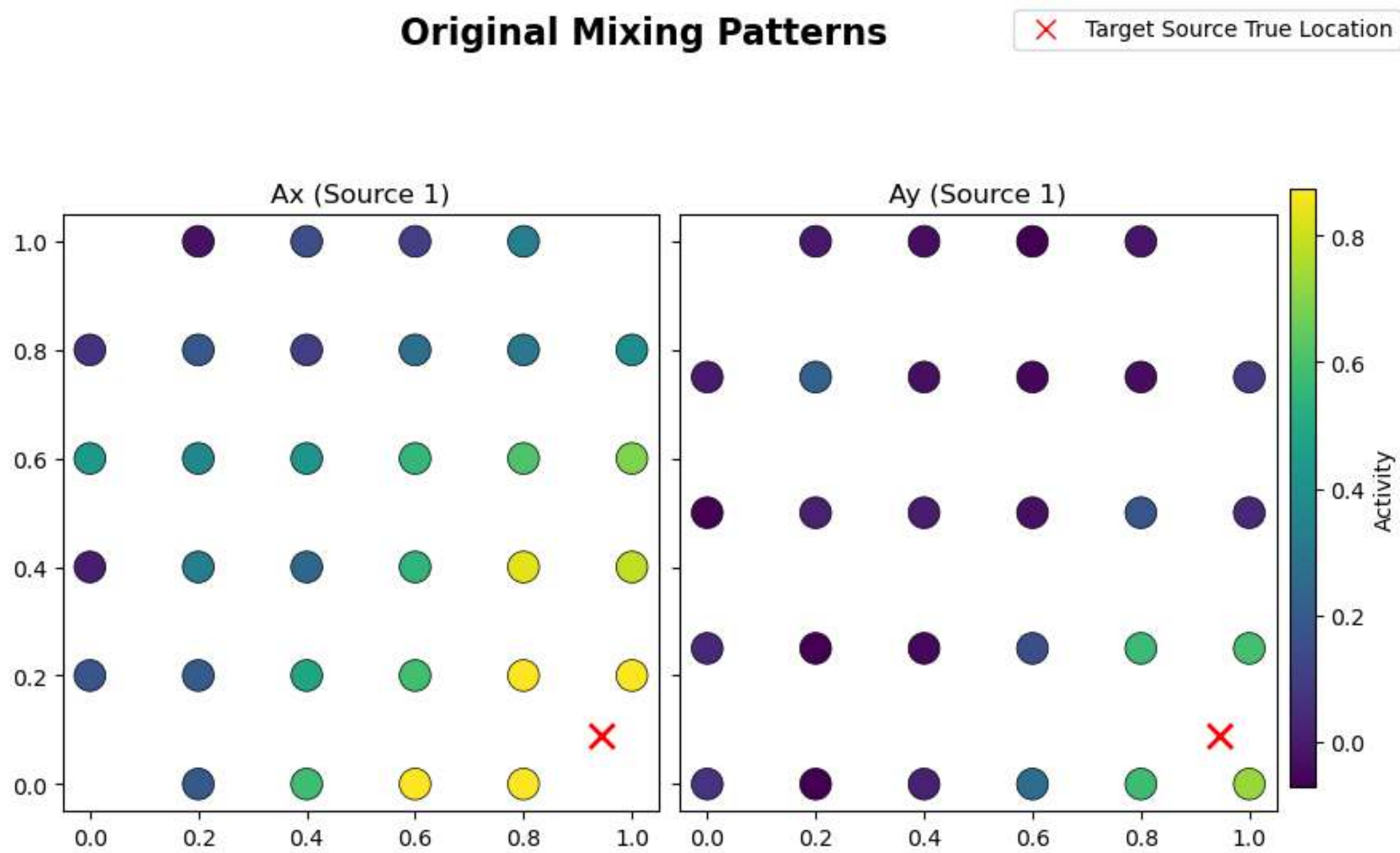

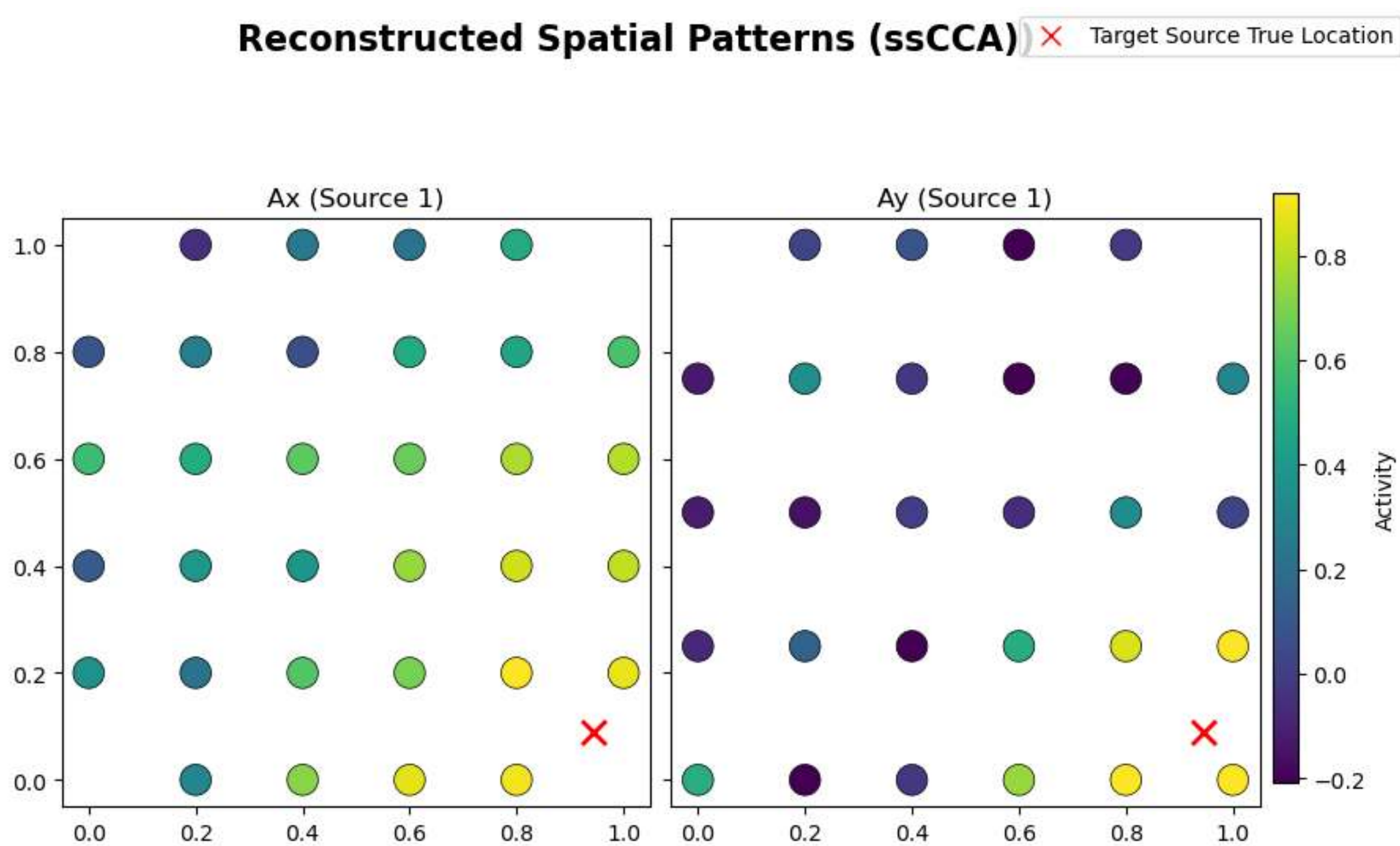

Additional Functionalities

Apart from the functionalities already covered for the regularized and standard CCA implementations, there are few addtional features that are worth mentioning. Given their usage are rather straightforward, we leave the exploration to the curious reader.

Multiple Components

So far we just covered the one-unit algorithms, i.e. we extracted only one component/source. As mentioned above, all these methods can be used to extract several components by changing the value of the `N_component` parameter. That value cannot be bigger than the minimum between the number of X and Y features. The way these extra components are extracted is by following a deflation approach, where at each iteration the contribution from the previous component is removed from the input matrix entering the decomposition.

[16]:
```
# Example case of multiple components CCA
cca_multiple_comp = CCA(N_components=10)
cca_multiple_comp.fit(x_power_train, y_train)
display(cca_multiple_comp.Wy)
```

```
<xarray.DataArray (channel: 28, CCA_Y: 10)> Size: 2kB
0.009316 -0.04114 0.3194 -0.1812 -0.2955 … -0.5022 -0.1405 -0.474 0.02501
Coordinates:
  * channel  (channel) <U3 336B 'Y1' 'Y2' 'Y3' 'Y4' … 'Y25' 'Y26' 'Y27' 'Y28'
  * CCA_Y    (CCA_Y) <U4 160B 'Sy1' 'Sy2' 'Sy3' 'Sy4' … 'Sy8' 'Sy9' 'Sy10'
```

Partial Least Squares (PLS)

PLS is a multimodal source-decomposition algorithm very similar to CCA. The latter maximizes correlation between the projected views, while PLS maximizes covariance. From a practical point of view, PLS can be obtained from CCA by sending the covariance matrices C_x and C_y to the identity matrices in the cost function. Therefore, both algorithms coincide when the input datasets are whitened.

Within Cedalion, we can use these methods via the classes `PLS`, and `SparsePLS` [Witten et al. 2009]. The Ridge version of PLS simply does not exist, because PLS itself can be understood as an extreme case of RidgeCCA where $\lambda_2 \to \infty$. Internally, the classes are obtained from `ElasticNetCCA` with the extra parameter `pls=True`.

[17]:
```
from cedalion.sigdecomp.multimodal.cca import PLS, SparsePLS
```

[18]:
```
# Example initialization of PLS and SparsePLS models
pls = PLS(N_components=1, max_iter=1000, tol=1e-6, scale=True)
sparse_pls = SparsePLS(N_components=1, l1_reg=[0.1, 0.01])
```

Temporally Embedded CCA (tCCA)

The methods covered so far assume modalities correlate instantaneously, which is certainly not the case in fNIRS-EEG fusion, for instance, where the common underlying sources are time-shifted due to hemodynamic delays. Temporally embedded CCA (tCCA) [Biessmann et al., 2010] captures such temporal offsets. While effective, the idea is rather simple: assuming y is the "delayed" modality, x is time-embedded by concatenating time-shifted copies along the feature dimension, leading to a time-embedded dataset $\tilde{x}$. The time lags must be preselected, introducing a new parameter to the model. Standard CCA (or any of the variants explored above) is then applied without further modifications on $(\tilde{x}, y)$, yielding temporal filters that capture delayed correlations between modalities.

All the CCA variants covered above admit a temporally embedded extension, and they are implemented in Cedalion in the `tcca_models` module, which contains the classes `tCCA`, `ElasticNetTCCA`, and `StructuredSparseTCCA`. Analogously to the simultaneuously-coupled models, Sparse tCCA and Ridge tCCA can be obtained from `ElasticNetTCCA` by the right choice of `l1_reg` and `l2_reg` parameters, but in this case they do not have their own classes.

[19]:
```python
from cedalion.sigdecomp.multimodal.tcca import (
    tCCA,
    # ElasticNetTCCA,
    # StructuredSparseTCCA,
)
```

In order to show how these models are used, let's build a time-shifted version of the simulated data from above:

[20]:
```python
# Use the same configuration dictionary as before but with a non-zero time lag
config_dict["dT"] = 2  # Time lag between target sources (s)

# Simulate
sim = BimodalToyDataSimulation(config_dict, seed=137, mixing_type="structured")
SNR = 20 * np.log10(sim.args.gamma)  # Calculate SNR in dB
print(f"SNR: {SNR:.2f}")
print("Time lag between target sources:", sim.args.dT, "s")

# Plot target sourcers, channels, and mixing patterns
# (add xlim now to see the effect of time lag)
sim.plot_targets(xlim=(0, 20))
# sim.plot_channels(N=2)
# sim.plot_mixing_patterns()

# Run small preprocessing step to standardize and
# split the data into train and test sets
train_test_split = 0.8  # Proportion of data to use for training
preprocess_data_dict = sim.preprocess_data(train_test_split)

x_train, x_test = preprocess_data_dict["x_train"], preprocess_data_dict["x_test"]

x_power_train, x_power_test = (
    preprocess_data_dict["x_power_train"],
    preprocess_data_dict["x_power_test"],
)

y_train, y_test = preprocess_data_dict["y_train"], preprocess_data_dict["y_test"]

sx, sx_power, sy = (
    preprocess_data_dict["sx"],
    preprocess_data_dict["sx_power"],
    preprocess_data_dict["sy"],
)
```

```
Random seed set as 137
Simulating sources…

Finished

SNR: -4.44
Time lag between target sources: 2 s
```

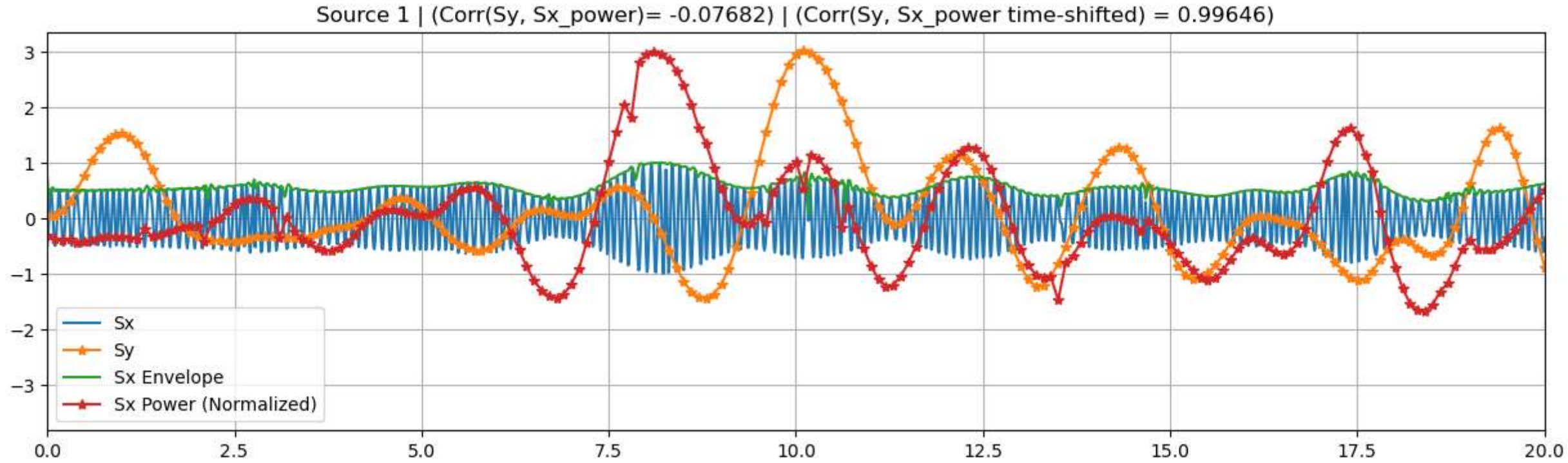

These time-embedded classes are built on top of their simultaneuously-coupled counterparts, sharing several attributes and methods. In particular, they accept the same parameters as before, in addition to `time_shifts` and `shift_source`.

The former must be a `Numpy` array, containing the time-lags to be applied to the x modality. These lags should be non-negative float numbers within the time domain of the data. Because of the latter, a validation/consistency check of this array is carried only after fitting the model. During that validation, a time-lag = 0 component would be added to `time_shift` if not present already (zero-lag x copy), and the resulting array is sorted in ascending order.

If the `shift_source` parameter is set to `True`, the reconstructed source s_x, obtained after transforming the data, is shifted by an `optimal_shift`, so it is temporally aligned with the reconstructed source s_y. This optimal shift attribute is estimated right after training by looking for the time-shifted x that produces the biggest correlation between reconstructed sources s_x and s_y. Note that the `optimal_shift` attribute is available even when `shift_source=False`, so one can apply the shift a posteri if desired. It is worth clarifying that this shift is only estimated using the (train) data used during fitting.

[21]:
```
# Temporal embedding parameters
dt = 1
N_lags = 5
time_shifts = np.arange(0, dt * N_lags, dt)
print(f"Time shifts: {time_shifts}")
print("True time lag between target sources:", sim.args.dT, "s")

# Initialize tCCA model
tcca = tCCA(
    N_components=1,
    max_iter=1000,
    tol=1e-6,
    scale=True,
    time_shifts=time_shifts,
    shift_source=True,
)
```

```
Time shifts: [0 1 2 3 4]
True time lag between target sources: 2 s
```

Fitting works in the same way as before, except that `sample_name` is assumed always to be `time`.

[22]:
```python
# Fit model
tcca.fit(x_power_train, y_train, featureX_name="channel", featureY_name="channel")

# At this point we have an estimate for the time lag between target sources
print(
    f"Estimated time lag between target sources "
    f"during training: {tcca.optimal_shift[0]} s"
)
display(tcca.Wx)
```

```
Estimated time lag between target sources during training: 2.0 s
```

```
<xarray.DataArray (time_shift: 5, channel: 32, tCCA_X: 1)> Size: 1kB
0.00386 0.002902 0.01951 -0.01833 -0.0143 … 0.03439 0.01409 0.003379 0.05622
Coordinates:
  * time_shift  (time_shift) int64 40B 0 1 2 3 4
  * channel     (channel) <U3 384B 'X1' 'X2' 'X3' 'X4' … 'X30' 'X31' 'X32'
  * tCCA_X      (tCCA_X) <U3 12B 'Sx1'
```

The learned weights `Wx` now have an extra `time_shift` dimension, where each component contains the filters applied to the copy of x shifted by that particular time-lag. `Wy` remains with the same shape as previous models.

Data transformation is also applied in the same manner, but in this case, if `shift_sources=True`, the reconstructed sources are truncated by removing the last `optimal shift` seconds, containing null values introduced by the zero-padding used during shifting.

[23]:
```python
# Transform data
sx_power_tcca, sy_tcca = tcca.transform(x_power_test, y_test)

display(sx_power_tcca, sx_power)
```

```
<xarray.DataArray (time: 580, tCCA_X: 1)> Size: 5kB
2.014 0.707 0.3418 0.8845 -0.2528 … -0.06712 -0.4116 0.5565 0.4488 0.2566
Coordinates:
  * time     (time) float64 5kB 240.1 240.2 240.3 240.4 … 297.8 297.9 298.0
  * tCCA_X   (tCCA_X) <U3 12B 'Sx1'
```

```
<xarray.DataArray (source: 1, time: 600)> Size: 5kB
0.8019 0.3854 -0.06178 -0.4672 -0.7737 -0.9842 … 1.732 2.067 2.226 2.188 1.977
Coordinates:
  * time     (time) float64 5kB 240.1 240.2 240.3 240.4 … 299.8 299.9 300.0
  * source   (source) <U2 8B 'S1'
```

Because of this sample number mismatch with the ground truth sources, we need to truncate the latter before any comparisson:

[24]:
```python
# Normalize
sx_power_tcca = standardize(sx_power_tcca).T[0]
sy_tcca = standardize(sy_tcca).T[0]

# Truncate ground truth sources to match the reconstructed sources
if len(sx_power_tcca) < len(sx_power.time):
    sx_power_trunc = sx_power[0, : len(sx_power_tcca)]
if len(sy_tcca) < len(sy.time):
```

```
    sy_trunc = sy[0, : len(sy_tcca)]

# Calculate correlations. Should be low because sx is shifted!
corrxy_tcca = np.corrcoef(sx_power_tcca, sy_tcca)[0, 1]
corrx_tcca = np.corrcoef(sx_power_tcca, sx_power_trunc)[0, 1]
corry_tcca = np.corrcoef(sy_tcca, sy_trunc)[0, 1]
```

We also train a standard CCA method so we can compare and see the importance of capturing the time lags in the data:

[25]:
```
# CCA
cca = CCA(N_components=1)
cca.fit(x_power_train, y_train)
sx_cca, sy_cca = cca.transform(x_power_test, y_test)
# Normalize
sx_cca = standardize(sx_cca).T[0]
sy_cca = standardize(sy_cca).T[0]
# Calculate correlations for CCA
corrxy_cca = np.corrcoef(sx_cca, sy_cca)[0, 1]
corrx_cca = np.corrcoef(sx_cca, sx_power)[0, 1]
corry_cca = np.corrcoef(sy_cca, sy)[0, 1]
```

[26]:
```
# Plot results
plot_source_comparisson(
    sx_power_trunc,
    sy_trunc,
    sx_power_tcca,
    sy_tcca,
    corrx_tcca,
    corry_tcca,
    corrxy_tcca,
    title="tCCA",
)
plot_source_comparisson(
    sx_power[0], sy[0], sx_cca, sy_cca, corrx_cca, corry_cca, corrxy_cca, title="CCA"
)
```

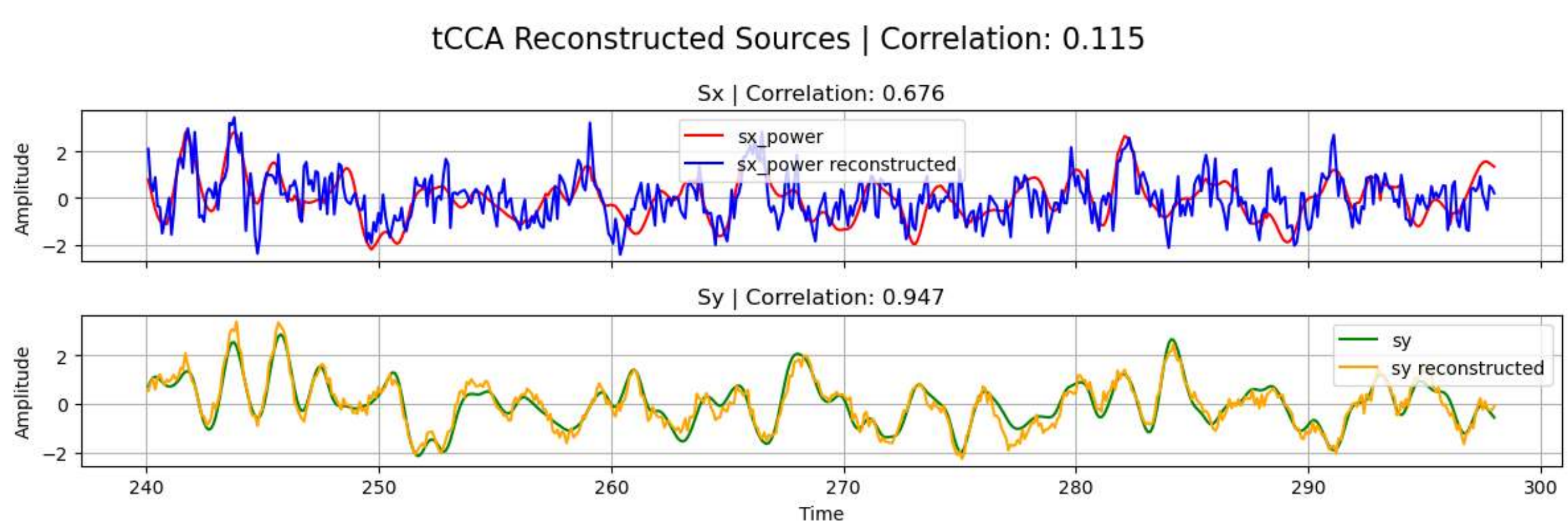

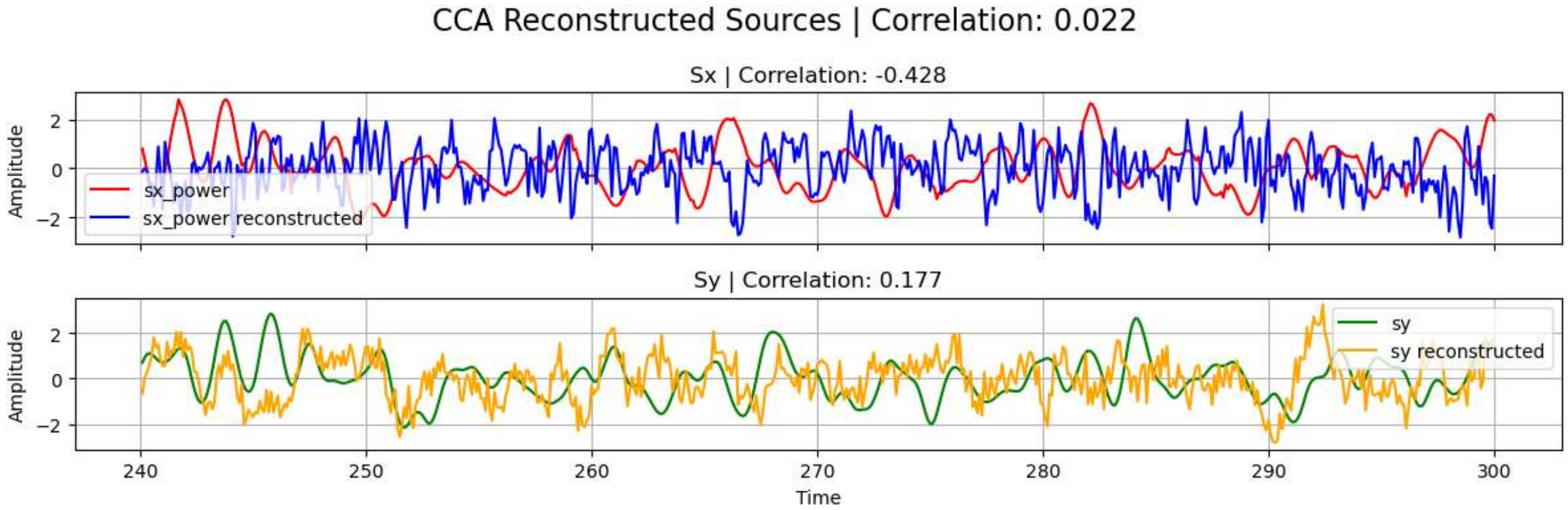

Note that, in the previous plot, the correlation between the reconstructed sources from tCCA is naturally low since s_x has been shifted with the estimated `optimal_shift`.

Aditional Functionalities

As for the regularized CCA classes, the temporally-embedded extensions can also extract multiple components (using the same deflation algorithm), and the PLS variant can be used by settting `pls=True` during initialization.

Multimodal Source Power Co-modulation (mSPoC)

EEG bandpower and hemodynamic responses captured by fNIRS are proven to comodulate during cognitive tasks. Conventional methods, such as CCA and its variants, however, present some limitations to integrate bandpower signals. More precisely, computing bandpower at channel level and then applying a backward linear method, as we have been doing so far, is not in line with the assumed linear generative model of EEG. mSPoC [Dähne, et.al. 2013] arises as a method that avoids these pitfalls by inverting the generative model prior to computing bandpower, and is implemented in Cedalion as the `mSPoC` class inside the `mspoc` module.

The algorithm takes two vector-valued time series $x(t)$, and $y(e)$, which are time-aligned but have different sample dimensions `Ntx` > `Nty`. It is expected that $x(t)$ has been previously bandpassed filtered to the band of interest. It finds components $s_x(t)$ and $s_y(e)$ via linear projection of the observations, such that the covariance between the temporally-embedded,bandpower of $s_x(t)$ and the time course of $s_y(e)$ is maximized. The bandpower of $s_x(t)$ is estimated by dividing the source into non-overlapping time windows (or epochs), such that there is one window per data point $y(e)$, and computing the variance within epochs. After such operation, both signals share the same sampling rate and their cross-covariance can be calculated. The solution to the optimization problem is captured by the spatial (`Wx`, `Wy`), and temporal (`Wt`) filters.

[27]:
```
from cedalion.sigdecomp.multimodal.mspoc import mSPoC
```

The `mSPoC` class accepts the very same parameters as the `tCCA` class, namely `N_components`, `max_iter`, `tol`, `scale`, `time_shifts`, and `shift_source`, with the addition of `N_restarts`. The latter determines the number of times the algorithm is repeated, which may find better extrema in the optimization problem, due to its stochastic nature. Typical values for `N_restarts` lie between 2 and 10, and it is recommended to choose smaller `max_iter` and bigger `tol` values, compared to simpler CCA methods, due the increased computational time required in mSPoC. As all previous CCA-based methods, mSPoC can also extract multiple components.

[28]:
```
# We run the same simulation as for tCCA above
config_dict["dT"] = 2
sim = BimodalToyDataSimulation(config_dict, seed=137, mixing_type="structured")
```

```python
SNR = 20 * np.log10(sim.args.gamma)  # Calculate SNR in dB
print(f"SNR: {SNR:.2f}")
print("Time lag between target sources:", sim.args.dT, "s")
sim.plot_targets(xlim=(0, 20))

# Run small preprocessing step to standardize and
# split the data into train and test sets
train_test_split = 0.8
preprocess_data_dict = sim.preprocess_data(train_test_split)

x_train, x_test = preprocess_data_dict["x_train"], preprocess_data_dict["x_test"]
x_power_train, x_power_test = (
    preprocess_data_dict["x_power_train"],
    preprocess_data_dict["x_power_test"],
)

y_train, y_test = preprocess_data_dict["y_train"], preprocess_data_dict["y_test"]
sx, sx_power, sy = (
    preprocess_data_dict["sx"],
    preprocess_data_dict["sx_power"],
    preprocess_data_dict["sy"],
)

# Temporal embedding parameters (same as for tCCA)
dt = 1
N_lags = 5
time_shifts = np.arange(0, dt * N_lags, dt)
print(f"Time shifts: {time_shifts}")
print("True time lag between target sources:", sim.args.dT, "s")

# Initialize mSPoC model
mspoc = mSPoC(
    N_components=1,
    N_restarts=2,
    max_iter=100,  # Note we use much smaller values than before
    tol=1e-4,
    scale=True,
    time_shifts=time_shifts,
    shift_source=True,
)
```

```
Random seed set as 137
Simulating sources…

Finished

SNR: -4.44
Time lag between target sources: 2 s
```

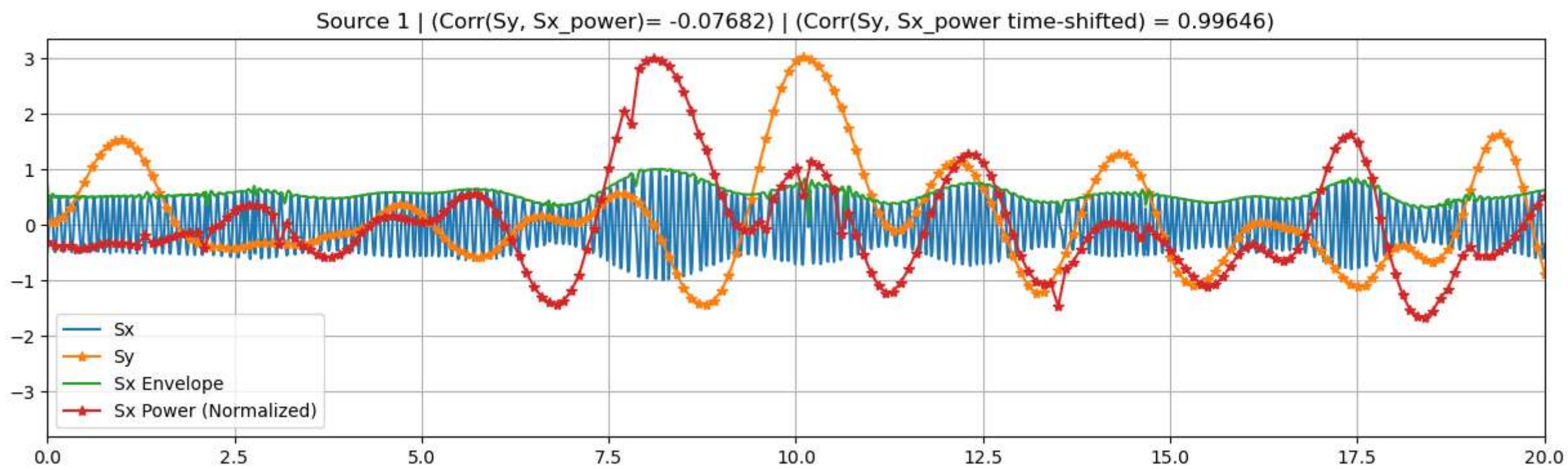


```
Time shifts: [0 1 2 3 4]
True time lag between target sources: 2 s
```

Fit and transform work analogously to the `tCCA` method, except that this time the number of samples in the x input should be bigger than the number of y samples.

[29]:
```
# Fit model (sample_name fixed to be 'time' in mSPoC)
mspoc.fit(
    x_train,
    y_train,  # Note that we use x_train here, not x_power_train
    featureX_name="channel",
    featureY_name="channel",
)

# At this point we have an estimate for the time lag between target sources
print(
    "Estimated time lag between target sources "
    f"during training: {mspoc.optimal_shift[0]} s"
)

# Learned filters
display(mspoc.Wx)
display(mspoc.Wt)
```

```
Estimated time lag between target sources during training: 2 s

<xarray.DataArray (channel: 32, mSPoC_X: 1)> Size: 256B
0.009032 -0.02669 0.05469 -0.04415 -0.02208 … -0.1543 -0.05827 -0.1278 -0.0265
Coordinates:
  * channel  (channel) <U3 384B 'X1' 'X2' 'X3' 'X4' … 'X29' 'X30' 'X31' 'X32'
  * mSPoC_X  (mSPoC_X) <U3 12B 'Sx1'

<xarray.DataArray (time_embedding: 5, mSPoC_T: 1)> Size: 40B
-0.06374 -0.0625 0.991 -0.08447 -0.05272
Coordinates:
  * time_embedding  (time_embedding) int64 40B 0 1 2 3 4
  * mSPoC_T         (mSPoC_T) <U3 12B 'St1'
```

The reconstructed sources returned by `transform` correspond to the temporally-embedded bandpower of the source s_x, and the time course of s_y. Because of this choice, the outputs are directly comparable, since

they have the same sampling time. As before, whenever `shift_source=True`, the time dimensions of the sources are truncated, removing the last `optimal_shift` seconds.

[30]:
```
# Transform data
sx_power_mspoc, sy_mspoc = mspoc.transform(x_test, y_test)
display(sx_power_mspoc, sx_power)
display(sy_mspoc, sy)

# Normalize
sx_power_mspoc = standardize(sx_power_mspoc).T[0]
sy_mspoc = standardize(sy_mspoc).T[0]
```

```
<xarray.DataArray (time: 580, mSPoC_X: 1)> Size: 5kB
0.9518 0.8879 0.838 1.385 1.158 1.05 … 0.8189 0.9737 0.626 1.605 2.467 1.912
Coordinates:
  * time     (time) float64 5kB 240.1 240.2 240.3 240.4 … 297.8 297.9 298.0
  * mSPoC_X  (mSPoC_X) <U3 12B 'Sx1'

<xarray.DataArray (source: 1, time: 600)> Size: 5kB
0.8019 0.3854 -0.06178 -0.4672 -0.7737 -0.9842 … 1.732 2.067 2.226 2.188 1.977
Coordinates:
  * time     (time) float64 5kB 240.1 240.2 240.3 240.4 … 299.8 299.9 300.0
  * source   (source) <U2 8B 'S1'

<xarray.DataArray (time: 580, mSPoC_Y: 1)> Size: 5kB
0.6547 1.091 1.502 0.9325 1.586 … -0.07052 -0.5063 -0.2778 -0.4498 -0.1842
Coordinates:
  * time     (time) float64 5kB 240.1 240.2 240.3 240.4 … 297.8 297.9 298.0
  * mSPoC_Y  (mSPoC_Y) <U3 12B 'Sy1'

<xarray.DataArray (source: 1, time: 600)> Size: 5kB
0.7055 0.9216 1.062 1.11 1.077 0.9927 … 1.532 1.584 1.563 1.5 1.425 1.356
Coordinates:
  * source   (source) <U2 8B 'S1'
  * time     (time) float64 5kB 240.1 240.2 240.3 240.4 … 299.8 299.9 300.0
```

As before, we truncate the ground truth sources for a direct comparison

[31]:
```
# Truncate ground truth sources to match the reconstructed sources
if len(sx_power_mspoc) < len(sx_power.time):
    sx_power_trunc = sx_power[0, : len(sx_power_mspoc)]
else:
    sx_power_trunc = sx_power[0]
if len(sy_mspoc) < len(sy.time):
    sy_trunc = sy[0, : len(sy_mspoc)]
else:
    sy_trunc = sy[0]

# Calculate correlations. corrxy_mspoc should be low because sx is shifted!
corrxy_mspoc = np.corrcoef(sx_power_mspoc, sy_mspoc)[0, 1]
corrx_mspoc = np.corrcoef(sx_power_mspoc, sx_power_trunc)[0, 1]
corry_mspoc = np.corrcoef(sy_mspoc, sy_trunc)[0, 1]

# Plot results
```

```
plot_source_comparisson(
    sx_power_trunc,
    sy_trunc,
    sx_power_tcca,
    sy_tcca,
    corrx_mspoc,
    corry_mspoc,
    corrxy_mspoc,
    title="mSPoC",
)
```

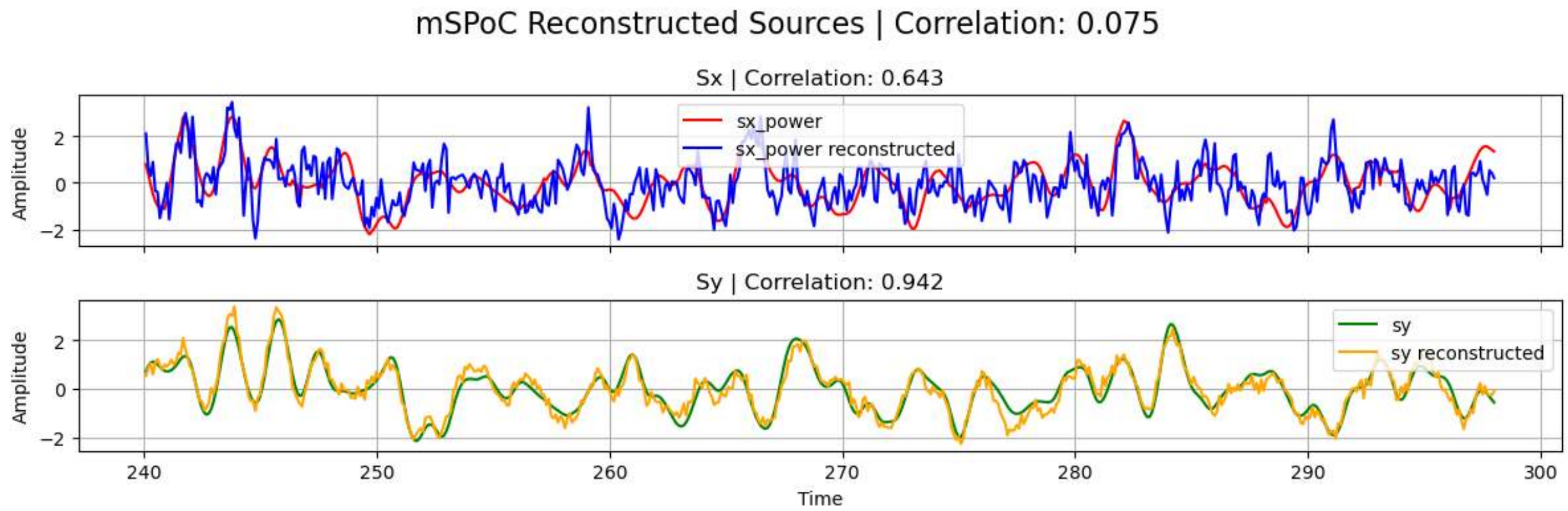

ICA Source Extraction

In this notebook we will investigate an example on how Independent Component Analysis by Entropy Rate Bound Minimization (ICA-ERBM) can be used to extract physiological sources from fNIRS data. For this example, we will use a finger tapping dataset.
Let $X \in \mathbb{R}^{N \times T}$ denote the finger tapping data with N channels and T time points. We assume that the data X consists of unknown independent sources $S \in \mathbb{R}^{N \times T}$ that were mixed through an unknown mixing matrix $A \in \mathbb{R}^{N \times N}$, such that

$$X = A \cdot S.$$

Source reconstruction in ICA-ERBM is done by minimizing the mutual information rate of the estimated sources. A demixing matrix W is determined and the estimated sources $\hat{S} \in \mathbb{R}^{N \times T}$ can be computed as

$$\hat{S} = W \cdot X.$$

Among the extracted sources, we will identify the ones that correspond to the PPG and Mayer Wave signals.

Loading Raw Finger Tapping Data

[32]:
```
# Load finger tapping data set
finger_tapping_data = cedalion.data.get_fingertappingDOT()

# Extract the fnirs recording
fnirs_data = finger_tapping_data['amp']
```

```
# Plot three channels of the fnirs data
fig, ax = plt.subplots(3, 1, sharex=True, figsize=(10, 5))
for i, ch in enumerate(["S1D1", "S1D2", "S7D9"]):
    ax[i].plot(fnirs_data.time, fnirs_data.sel(channel=ch, wavelength="760"), "r-",␣
 ↪label="760nm")
    ax[i].plot(fnirs_data.time, fnirs_data.sel(channel=ch, wavelength="850"), "b-",␣
 ↪label="850nm")
    ax[i].set_title(f"Channel {ch}")

ax[0].legend()
ax[2].set_xlim(0,60)
ax[2].set_xlabel("time / s")
plt.tight_layout()
```

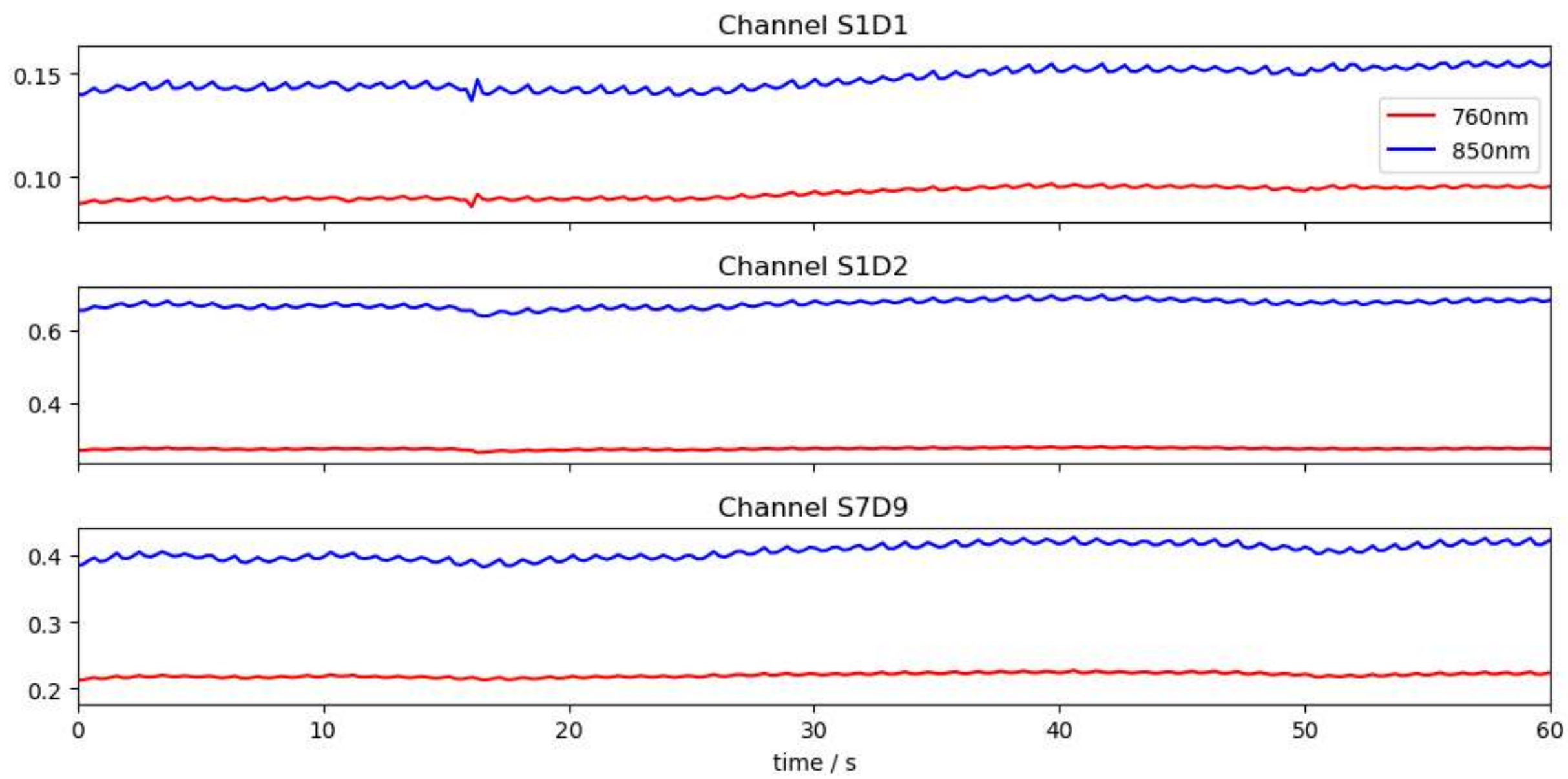

Conversion to Optical Density

[33]:
```
# Convert to Optical Density (OD)
fnirs_data_od = cedalion.nirs.cw.int2od(fnirs_data)
```

Channel Quality Assessment and Pruning

The Scalp Coupling Index (SCI) and Peak Spectral Power (PSP) are used for quality assessment. We compute SCI and PSP for each channel, and remove channels with less than 75% of clean time.

[34]:
```
# Calculate masks for SCI and PSP quality metrics
window_length = 5 * units.s
sci_thresh = 0.75
psp_thresh = 0.1
sci_psp_percentage_thresh = 0.75
```

```
sci, sci_mask = quality.sci(fnirs_data_od, window_length, sci_thresh)
psp, psp_mask = quality.psp(fnirs_data_od, window_length, psp_thresh)
sci_x_psp_mask = sci_mask & psp_mask
perc_time_clean = sci_x_psp_mask.sum(dim="time") / len(sci.time)
sci_psp_mask = [perc_time_clean >= sci_psp_percentage_thresh]

# Prune channels that do not pass the quality test
fnirs_data_pruned, drop_list = quality.prune_ch(fnirs_data_od, sci_psp_mask, "all")

# Display pruned channels
print(f"List of pruned channels: {drop_list}  ({len(drop_list)})")
```

```
List of pruned channels: ['S13D26']  (1)
```

High-pass filter

[35]:
```
# Filter the data
# fmax = 0 is used to indicate high-pass filtering
fnirs_data_filtered = fnirs_data_pruned.cd.freq_filter(fmin= 0.01, fmax= 0,␣
↪butter_order=4)

# Store sampling rate
fnirs_data_samplingrate = sampling_rate(fnirs_data_pruned.time).magnitude

# Plot the filtered data
fig, ax = plt.subplots(3, 1, sharex=True, figsize=(10, 5))
for i, ch in enumerate(["S1D1", "S1D2", "S7D9"]):
    ax[i].plot(fnirs_data_filtered.time, fnirs_data_filtered.sel(channel=ch,␣
↪wavelength="760"), "r-", label="760nm")
    ax[i].plot(fnirs_data_filtered.time, fnirs_data_filtered.sel(channel=ch,␣
↪wavelength="850"), "b-", label="850nm")
    ax[i].set_title(f"Channel {ch}")

ax[0].legend()
ax[2].set_xlim(0,60)
ax[2].set_label("time / s")
plt.tight_layout()
```

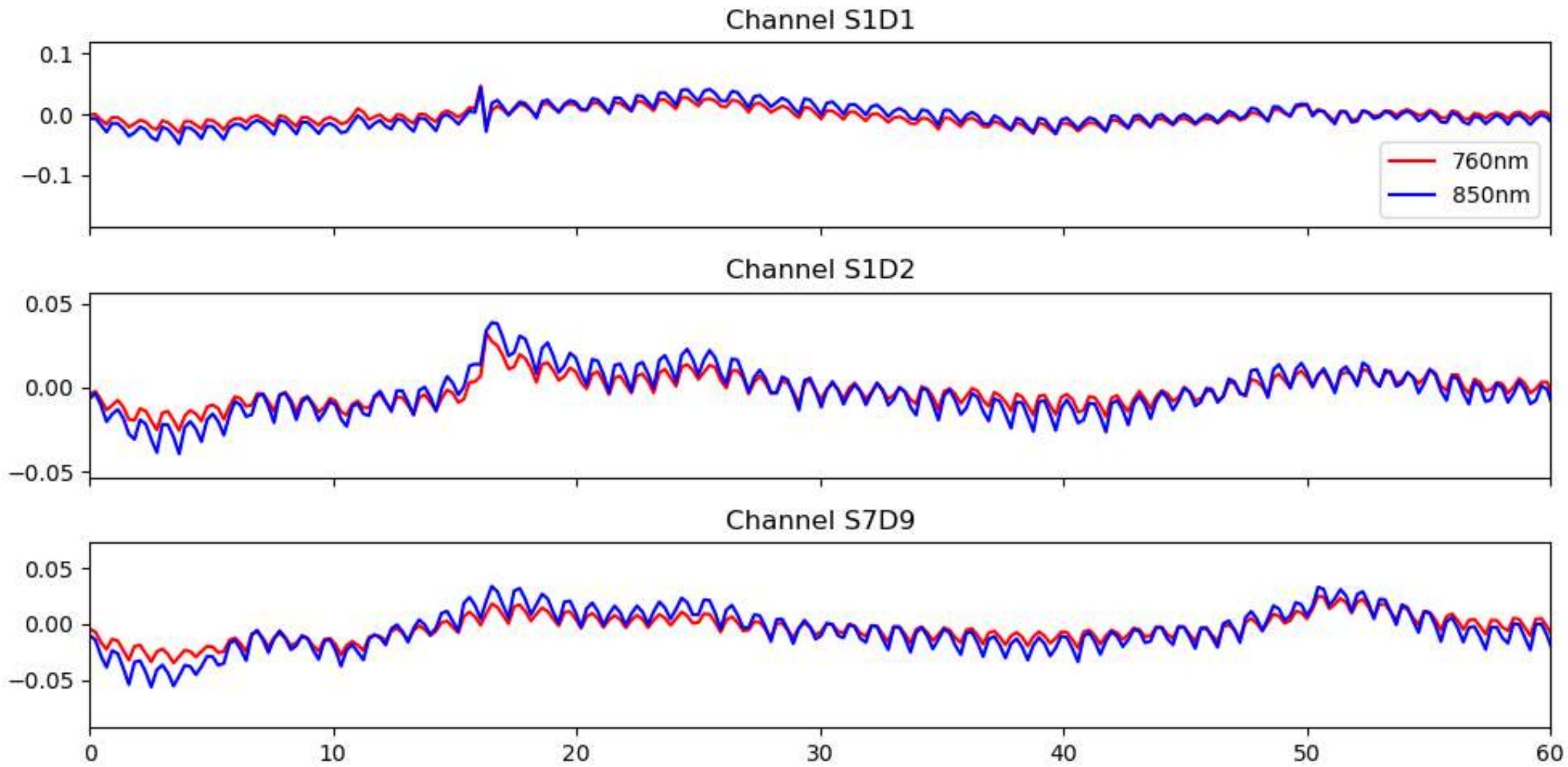

Select Channels and Time Slice for ICA

The entire finger tapping dataset was recorded over 30 minutes and contains 99 channels after pruning. Unfortunately, these dimensions result in a long runtime for ICA-ERBM. For this reason, we will use only a subset of the channels and a 10-minute slice of the selected channels. Despite the longer runtime, this example is also applicable to the full dataset.

[36]:
```
# Choose the best 30 channels based on the percentage of time clean
id_best_channels = np.argsort(perc_time_clean).values[-30:]
best_channels = fnirs_data['channel'][id_best_channels]

# Extract the best channels from the filtered data
fnirs_best_channels = fnirs_data_filtered.sel(channel = best_channels)

# Select a 10 min interval
duration = 10 * 60
buffer = 60
fnirs_best_channels = fnirs_best_channels.sel(time=slice(buffer, buffer + duration))

# Select the first wavelength
X = fnirs_best_channels.values[:, 0, :]
print(f"Shape of data for ICA-ERBM: {X.shape}")
```

```
Shape of data for ICA-ERBM: (30, 2616)
```

```
/opt/miniconda3/envs/cedalion_250922/lib/python3.11/site-
packages/xarray/core/variable.py:315: UnitStrippedWarning: The unit of the quantity is
stripped when downcasting to ndarray.
  data = np.asarray(data)
```

Apply ICA-ERBM

ICA-ERBM is applied to the selected channels. For the autoregressive filter used in ICA-ERBM, we use the default parameter $p = 11$. The source estimates are then computed as $\hat{S} = W \cdot X$.

[37]:
```
# Set filter length
p = 11

# Apply ICA-ERBM to the data
W = ICA_ERBM(X, p)

# Compute separated source as S = W * X
sources = W.dot(X)
```

[38]:
```
# Apply z-score normalization to the sources
sources_zscore = sp.stats.zscore(sources, axis=0)
```

Selection of PPG Source

From the reconstructed sources, we now want to identify those that are most similar to a PPG signal. To this end, we compare the frequency band in which the PPG signal is expected to have large amplitudes with the surrounding frequency bands. The sources with the highest contrast are selected. The PPG signal is expected to exhibit high amplitudes in a frequency band around 1 Hz.

[39]:
```
# Compute the frequency spectrum for each source
psd_sources = np.abs(np.fft.fft(sources, axis = 1))

# The frequencies corresponding to the spectrum
freqs = np.fft.fftfreq(sources.shape[1], 1/fnirs_data_samplingrate)

# Choose the indices of frequencies that are in the ppg band (0.75 - 1.25 Hz)
ppg_band_ind = np.logical_and(freqs >= 0.75, freqs <= 1.25)

# Choose the indices of frequencies that are in the band (0 - 0.75 Hz and 1.25 - 3.0␣
↪Hz)
comp_band =  np.logical_and(freqs >= 0, freqs < 0.75) +  np.logical_and(freqs > 1.25 ,␣
↪freqs <= 3.0)

# Compute the quotient of the  ppg band and the contrast band
psd_quotient = np.sum(psd_sources[:, ppg_band_ind], axis = 1 ) / np.sum(psd_sources[:,␣
↪comp_band], axis = 1 )

# Choose the indices of the sources with the highest contrast
max_contrast_index = np.argsort(psd_quotient, axis = 0 )[-5:]

# Reverse the order of the indices to have the highest contrast first
max_contrast_index = max_contrast_index[::-1]

# Choose the sources with the highest contrast
ppg_sources = sources_zscore[max_contrast_index, :]
```

[40]:
```
# Plot the sources with the highest contrast and their frequency spectrum
fig, ax = plt.subplots(ppg_sources.shape[0], 2, figsize=(12, 2 * ppg_sources.shape[0]))

for i in range(ppg_sources.shape[0]):
    # Plot the source for 60 seconds
    samples = int(fnirs_data_samplingrate * 60 * 1)
    ax[i, 0].plot( 1/fnirs_data_samplingrate * np.arange(0,samples), ppg_sources[i, :
 ↪samples], label=f"Source {max_contrast_index[i]+1}")
    ax[i, 0].set_title(f"Source {max_contrast_index[i] + 1}", fontsize=10)

    # Plot frequency spectrum of the source
    psd = np.abs(np.fft.rfft(ppg_sources[i, :])) ** 2
    x_freqs = np.fft.rfftfreq(ppg_sources.shape[1], 1 / fnirs_data_samplingrate)
    ax[i, 1].plot(x_freqs, psd, label="Contrast Band")
    ax[i, 1].set_title(f"Frequency Spectrum of Source {max_contrast_index[i]+1},␣
 ↪Contrast Quotient: {psd_quotient[max_contrast_index[i]]:.2f}",  fontsize=10)

    # Highlight the PPG band in the frequency spectrum
    highlight_ppg_band = np.logical_and(x_freqs >= 0.75, x_freqs <= 1.25)
    ax[i, 1].plot(x_freqs[highlight_ppg_band], psd[highlight_ppg_band],␣
 ↪color='orange', label='PPG Band')

ax[0, 1].legend()
ax[i, 0].set_xlabel("Time / s")
ax[i, 1].set_xlabel("Frequency / Hz")
fig.suptitle("PPG Sources", fontsize=16)
plt.tight_layout()
```

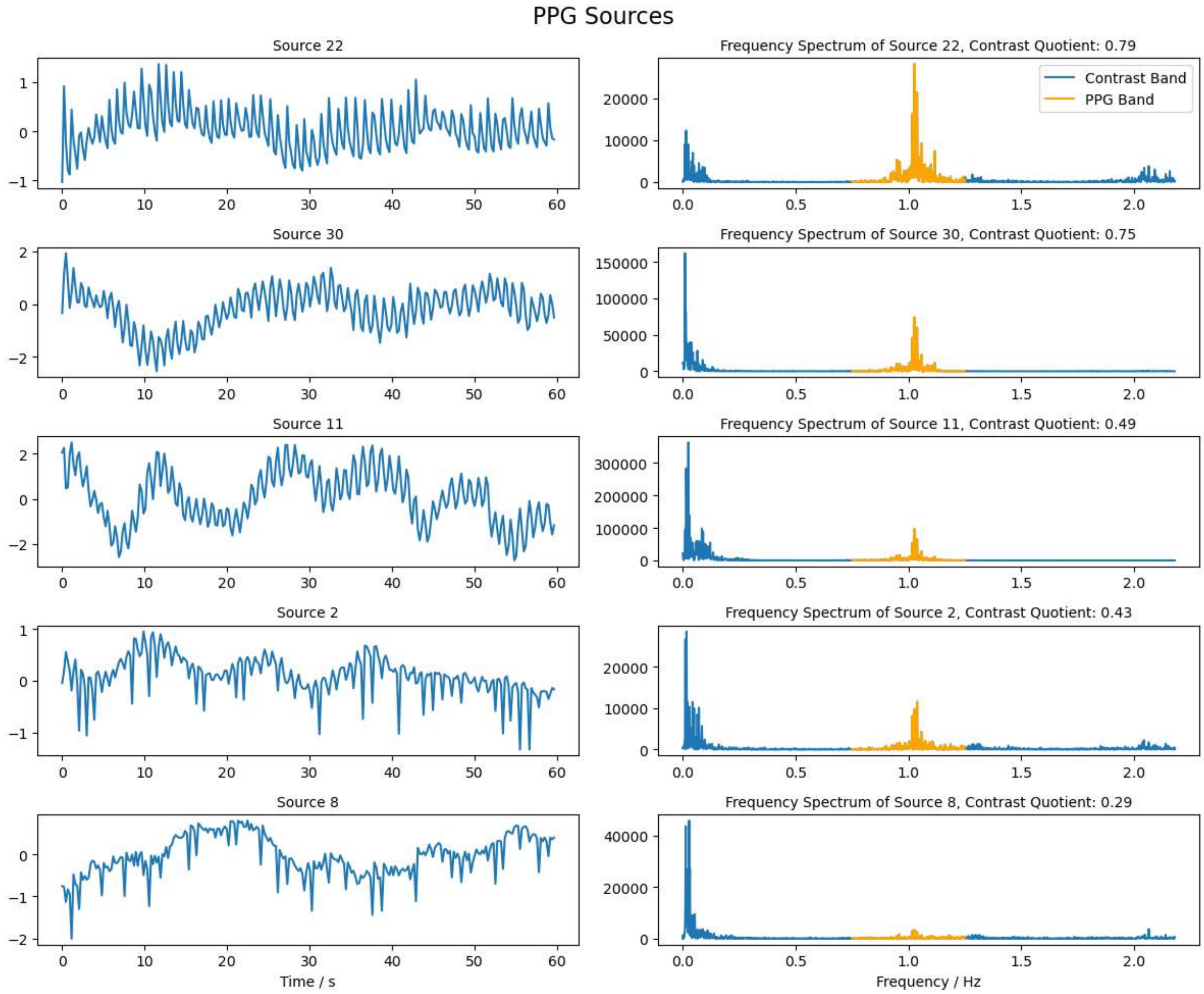

Selection of Mayer Wave Source

Mayer waves are expected to have a frequency around 0.1 Hz. Similar to the PPG sources above, we will use the contrast between the frequency band around 0.1 Hz and the surrounding bands to rank the sources and identify those that are most similar to the Mayer wave.

[41]:
```
# Choose the indices of frequencies that are in the Mayer Wave band (0.05 - 0.15 Hz)
mw_band_ind = np.logical_and(freqs >= 0.05, freqs <= 0.15)

# Choose the indices of frequencies that are in the band (0 - 0.05 Hz and 0.15 - 3.0 Hz)
comp_band =  np.logical_and(freqs >= 0, freqs < 0.05) +  np.logical_and(freqs > 0.15 , freqs <= 3.0)

# Compute the quotient of the Mayer Wave band and the contrast band
psd_quotient = np.sum(psd_sources[:, mw_band_ind], axis = 1 ) / np.sum(psd_sources[:, comp_band], axis = 1 )

# Choose the indices of the sources with the highest contrast
max_contrast_index = np.argsort(psd_quotient, axis = 0 )[-5:]
```

```
# Reverse the order of the indices to have the highest contrast first
max_contrast_index = max_contrast_index[::-1]

# Extract the sources with the highest contrast
mw_sources = sources_zscore[max_contrast_index, :]
```

[42]:
```
# Plot the sources with the highest contrast and their frequency spectrum
fig, ax = plt.subplots(mw_sources.shape[0], 2, figsize=(12, 2 * mw_sources.shape[0]))

for i in range(mw_sources.shape[0]):
    # Plot the source for 60 seconds
    samples = int(fnirs_data_samplingrate * 60 * 1)
    ax[i, 0].plot( 1/fnirs_data_samplingrate * np.arange(0,samples), mw_sources[i, :␣
 ↪samples], label=f"Source {max_contrast_index[i]+1}")
    ax[i, 0].set_title(f"Source {max_contrast_index[i] + 1}", fontsize=10)

    # Plot frequency spectrum of the source
    psd = np.abs(np.fft.rfft(mw_sources[i, :])) ** 2
    x_freqs = np.fft.rfftfreq(mw_sources.shape[1], 1 / fnirs_data_samplingrate)
    ax[i, 1].plot(x_freqs, psd, label="Contrast Band")
    ax[i, 1].set_title(f"Frequency Spectrum of Source {max_contrast_index[i]+1},␣
 ↪Contrast Quotient: {psd_quotient[max_contrast_index[i]]:.2f}",  fontsize=10)

    # Highlight the Mayer Wave band in the frequency spectrum
    highlight_mw_band = np.logical_and(x_freqs >= 0.05, x_freqs <= 0.15)
    ax[i, 1].plot(x_freqs[highlight_mw_band], psd[highlight_mw_band], color='orange',␣
 ↪label='Mayer Wave Band')

ax[0, 1].legend()
ax[i, 0].set_xlabel("Time / s")
ax[i, 1].set_xlabel("Frequency / Hz")
fig.suptitle("Mayer Wave Sources", fontsize=16)
plt.tight_layout()
```

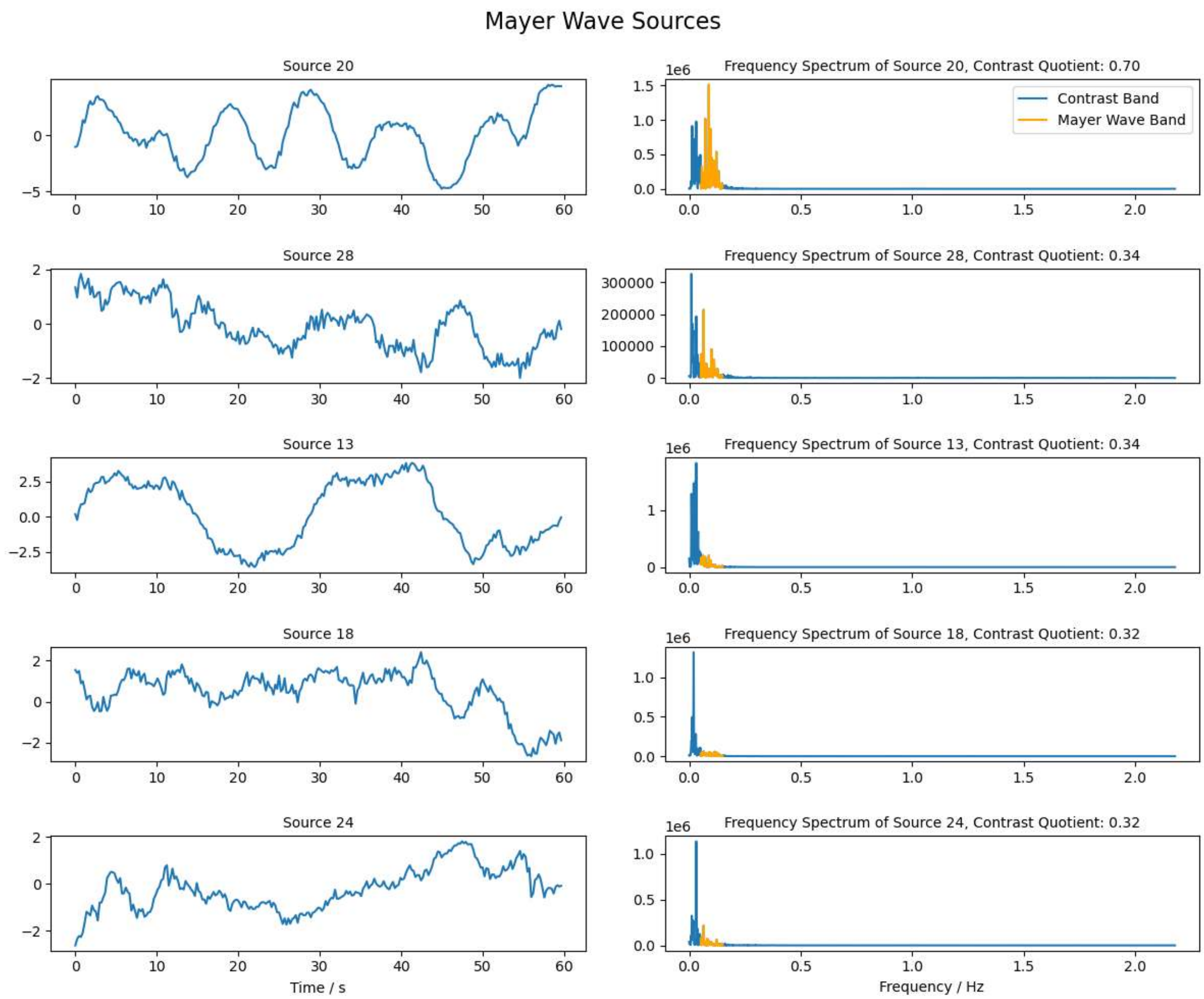

Single Trial Classification

The last section of this notebook demonstrates how Cedalion interfaces with scikit-learn to train a simple single-subject, single-trial classifier. The focus is on data flow: performing preprocessing with short-channel regression within a cross-validation scheme, extracting features from epochs, and passing these features to scikit-learn for training and evaluation while preserving feature metadata to allow tracing each feature back to its origin.

[43]:
```
from sklearn.discriminant_analysis import LinearDiscriminantAnalysis
from sklearn.preprocessing import LabelEncoder
from sklearn.inspection import permutation_importance

import cedalion.models.glm as glm
import cedalion.mlutils as mlutils
```

[44]:
```
rec = cedalion.data.get_fingertappingDOT()

# assign string labels to events and pool finger-tapping and ball-squeezing trials
rec.stim.cd.rename_events(
    {
```

```
        "1": "Control",
        "2": "Motor/Left",  # "FTapping/Left",
        "3": "Motor/Right", # "FTapping/Right",
        "4": "Motor/Left",  # "BallSqueezing/Left",
        "5": "Motor/Right", # "BallSqueezing/Right",
    }
)

# Keep only motor trials. Also remove the last trial so that there
# are equal numbers of trials for Motor/Left and Motor/Right
rec.stim = (
    rec.stim[rec.stim.trial_type.str.startswith("Motor")]
    .sort_values("onset")
    .reset_index(drop=True)
    .iloc[:-1]
)

display(rec.stim.groupby("trial_type").count())
display(rec.stim)
```

```
             onset  duration  value
trial_type
Motor/Left      32        32     32
Motor/Right     32        32     32

          onset  duration  value   trial_type
0      8.486912      10.0    1.0   Motor/Left
1     38.764544      10.0    1.0  Motor/Right
2     69.042176      10.0    1.0  Motor/Right
3     99.549184      10.0    1.0   Motor/Left
4    129.597440      10.0    1.0   Motor/Left
..          …         …    …            …
59  1837.301760      10.0    1.0   Motor/Left
60  1868.496896      10.0    1.0   Motor/Left
61  1899.003904      10.0    1.0  Motor/Right
62  1931.116544      10.0    1.0  Motor/Right
63  1962.541056      10.0    1.0   Motor/Left

[64 rows x 4 columns]
```

Preprocessing the dataset

As in previous notebooks, motion artifacts are corrected with the TDDR and wavelet algorithms. The data is transformed into optical densities and a global component is subtracted. After bandpass filtering, concentration changes are calculated.

[45]:
```
rec["od"] = cedalion.nirs.cw.int2od(rec["amp"])
rec["od_tddr"] = motion.tddr(rec["od"])
rec["od_wavelet"] = motion.wavelet(rec["od_tddr"])

# see 2_tutorial_preprocessing.ipynb for channel selection
bad_channels = ['S13D26', 'S14D28']
```

```
rec["od_clean"] = rec["od_wavelet"].sel(channel=~rec["od"].channel.isin(['S13D26',␣
↪'S14D28']))

od_var = quality.measurement_variance(rec["od_clean"], calc_covariance=False)

rec["od_mean_subtracted"], global_comp = physio.global_component_subtract(
    rec["od_clean"], ts_weights=1 / od_var, k=0
)

rec["od_freqfiltered"] = rec["od_mean_subtracted"].cd.freq_filter(
#rec["od_freqfiltered"] = rec["od_clean"].cd.freq_filter(
    fmin=0.01, fmax=0.5, butter_order=4
)

dpf = xr.DataArray(
    [6, 6],
    dims="wavelength",
    coords={"wavelength": rec["amp"].wavelength},
)

rec["conc"] = cedalion.nirs.cw.od2conc(rec["od_freqfiltered"], rec.geo3d, dpf)
```

Short-channel regression

Following the approach of von Lühmann et al. (2020), we apply a GLM to regress out physiological noise, thereby improving single-trial classification performance. To incorporate the GLM into a cross-validaton scheme, the design matrix will be masked for each cross-validation fold.

[46]:
```
# separate long and short channels
rec["conc_long"], rec["conc_short"] = cedalion.nirs.split_long_short_channels(
    rec["conc"], rec.geo3d, distance_threshold=22.5 * units.mm
)

# define the design matrix
dms = (
    glm.design_matrix.hrf_regressors(
        rec["conc_long"],
        rec.stim,
        glm.Gamma(tau=0 * units.s, sigma=3 * units.s),
    )
    & glm.design_matrix.drift_regressors(rec["conc_long"], drift_order=1)
    & glm.design_matrix.average_short_channel_regressor(rec["conc_short"])
)
dms
```

[46]:
```
DesignMatrix(common=['HRF Motor/Left','HRF Motor/Right','Drift 0','Drift 1','short'],
channel_wise=[])
```

The dataset has 64 trials, with equal numbers of "Motor/Left" and "Motor/Right" conditions. In the following this dataset is split into 4 folds using the function `mlutils.cv.create_cv_splits`.

For each fold, a masked design matrix is created by setting the time segment used for testing to zero.

The GLM parameters are estimated and the signal components explained by nuisance regressors are subtracted.

Finally, the time series is segmented into epochs, yielding for each cross-validation fold an array of samples, to which the trial information required for training is appended as additional coordinates.

[47]:
```
n_splits = 4
before = 5 * cedalion.units.s
after = 25 * cedalion.units.s


cv_folds = []

for i_split, (df_stim_train, df_stim_test) in enumerate(
    mlutils.cv.create_cv_splits(rec.stim, n_splits)
):
    # short-channel regression (SCR):

    # zero-out design matrix in test segment
    dms_masked = mlutils.cv.mask_design_matrix(
        dms,
        df_stim_test,
        before=before,
        after=after,
    )

    # fit long channels with masked design matrix
    result = glm.fit(rec["conc_long"], dms_masked, noise_model="ols")

    # compute component explained by short-channel regressor
    short_component = glm.predict(
        rec["conc_long"],
        result.sm.params.sel(regressor=["short", "Drift 0", "Drift 1"]),
        dms_masked,
    )
    short_component = short_component.pint.quantify(rec["conc_long"].pint.units)

    # subtract short component
    conc_long_scr = rec["conc_long"] - short_component

    # split time series into epochs
    epochs = conc_long_scr.cd.to_epochs(
        rec.stim,
        ["Motor/Left", "Motor/Right"],
        before=before,
        after=after,
    )

    # baseline correction
    baseline = epochs.sel(reltime=(epochs.reltime < 0)).mean("reltime")
    epochs = epochs - baseline
```

```python
    # assign train-/test-set membership...
    is_train = np.zeros(epochs.sizes["epoch"], dtype=bool)
    is_test = np.zeros(epochs.sizes["epoch"], dtype=bool)
    is_train[df_stim_train.index.values] = True
    is_test[df_stim_test.index.values] = True

    # ... and digitized trial labels ...
    label_encoder = LabelEncoder()
    y = label_encoder.fit_transform(epochs.trial_type.values)

    # ... as coordinates to the DataArray
    epochs = epochs.assign_coords(
        {
            "is_train": ("epoch", is_train),
            "is_test": ("epoch", is_test),
            "y" : ("epoch", y)
         }
    )

    cv_folds.append(epochs)
```

For each cross-validation fold the epoched time series looks like this:

[48]:
```python
cv_folds[0]
```

[48]:
```
<xarray.DataArray (epoch: 64, chromo: 2, channel: 44, reltime: 132)> Size: 6MB
[µM] 0.06823 0.04856 0.04232 0.03702 0.03248 … 0.04141 0.0395 0.03601 0.03161
Coordinates:
  * reltime      (reltime) float64 1kB -5.038 -4.809 -4.58 … 24.5 24.73 24.96
    trial_type   (epoch) <U11 3kB 'Motor/Left' 'Motor/Right' … 'Motor/Left'
  * chromo       (chromo) <U3 24B 'HbO' 'HbR'
  * channel      (channel) object 352B 'S1D6' 'S1D8' 'S2D5' … 'S14D25' 'S14D27'
    source       (channel) object 352B 'S1' 'S1' 'S2' 'S2' … 'S13' 'S14' 'S14'
    detector     (channel) object 352B 'D6' 'D8' 'D5' 'D9' … 'D28' 'D25' 'D27'
    is_train     (epoch) bool 64B False False False False … True True True True
    is_test      (epoch) bool 64B True True True True … False False False False
    y            (epoch) int64 512B 0 1 1 0 0 0 1 1 1 0 1 … 0 1 1 0 1 0 0 1 1 0
Dimensions without coordinates: epoch
```

The hemodynamic response to the two motor tasks can be visualized by block-averaging these epochs.

[49]:
```python
blockaverage = cv_folds[0].groupby("trial_type").mean("epoch")

# Plot block averages. Please ignore errors if the plot is too small in the HD case

noPlts2 = int(np.ceil(np.sqrt(len(blockaverage.channel))))
f,ax = plt.subplots(noPlts2,noPlts2, figsize=(12,10))
ax = ax.flatten()
for i_ch, ch in enumerate(blockaverage.channel):
    for ls, trial_type in zip(["-", "--"], blockaverage.trial_type):
        ax[i_ch].plot(blockaverage.reltime, blockaverage.sel(chromo="HbO",␣
↪trial_type=trial_type, channel=ch), "r", lw=2, ls=ls)
```

```
        ax[i_ch].plot(blockaverage.reltime, blockaverage.sel(chromo="HbR", trial_type=trial_type, channel=ch), "b", lw=2, ls=ls)

    ax[i_ch].grid(1)
    ax[i_ch].set_title(ch.values)
    ax[i_ch].set_ylim(-.3, .3)
    ax[i_ch].set_axis_off()
    ax[i_ch].axhline(0, c="k")
    ax[i_ch].axvline(0, c="k")

for i in range(len(blockaverage.channel), len(ax)):
    ax[i].set_axis_off()

plt.suptitle("HbO: r | HbR: b | left: - | right: --")
plt.tight_layout()
```

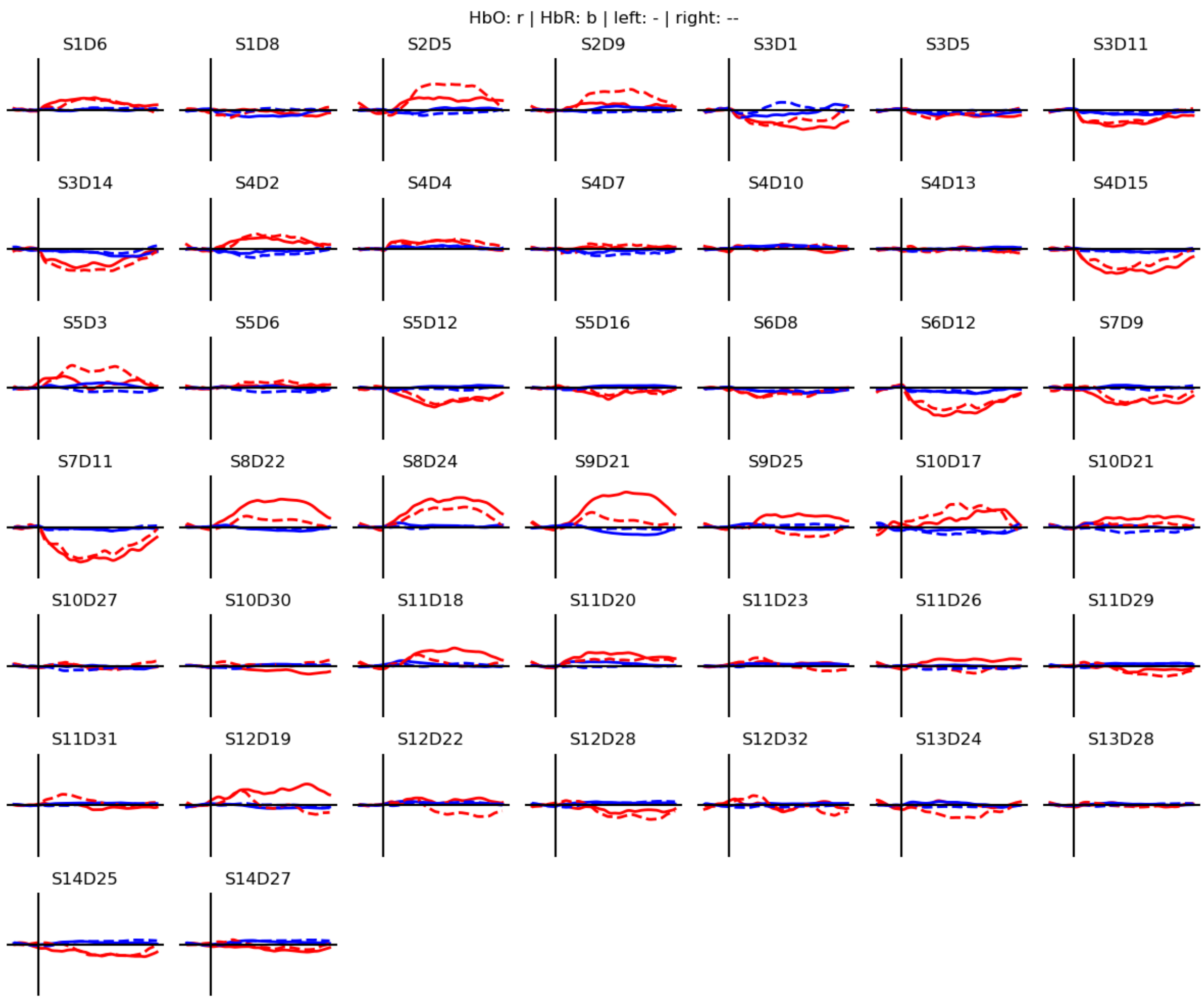

For each of the 64 epochs there are $N_{chromo} \times N_{channel}$ time traces. From these time traces, features must get extracted.

The function `mlutils.features.epoch_features` calculates common features of the hemodynamic response, such as slope, mean, maximum, minimum and area under the curve. For each feature type, a

time range can be specified, over which the feature is calculated. In the present case, this yields features for each channel and chromophore, which are then stacked into a single feature dimensions. The resulting array has the shape expected by scikit-learn, $(N_{samples}, N_{features})$.

[50]:
```
X = mlutils.features.epoch_features(
    cv_folds[0],
    feature_types=["slope", "mean", "max", "min", "auc"],
    reltime_slices={
        "slope": slice(0, 9),
        "mean": slice(3, 10),
        "max": slice(2, 8),
        "min": slice(2, 8),
    },
)
X
```

[50]:
```
<xarray.DataArray (epoch: 64, feature: 440)> Size: 225kB
0.02127 -0.01976 0.009303 -0.007574 -0.01678 … 0.07023 -0.04713 0.1401 0.214
Coordinates:
    trial_type    (epoch) <U11 3kB 'Motor/Left' 'Motor/Right' … 'Motor/Left'
    source        (feature) object 4kB 'S1' 'S1' 'S2' 'S2' … 'S13' 'S14' 'S14'
    detector      (feature) object 4kB 'D6' 'D8' 'D5' 'D9' … 'D28' 'D25' 'D27'
    is_train      (epoch) bool 64B False False False False … True True True
    is_test       (epoch) bool 64B True True True True … False False False
    y             (epoch) int64 512B 0 1 1 0 0 0 1 1 1 0 … 0 1 1 0 1 0 0 1 1 0
  * feature       (feature) object 4kB MultiIndex
  * feature_type  (feature) object 4kB 'slope' 'slope' 'slope' … 'auc' 'auc'
  * chromo        (feature) <U3 5kB 'HbO' 'HbO' 'HbO' … 'HbR' 'HbR' 'HbR'
  * channel       (feature) object 4kB 'S1D6' 'S1D8' … 'S14D25' 'S14D27'
Dimensions without coordinates: epoch
```

During stacking, the coordinates of the `'chromo'` and `'channel'` dimensions don't get lost. They are combined and reassigned to the new `'feature'` dimension. This means that for every feature in `X`, you can trace back which channel and chromophore it originated from.

[51]:
```
X.feature
```

[51]:
```
<xarray.DataArray 'feature' (feature: 440)> Size: 4kB
MultiIndex
Coordinates:
    source        (feature) object 4kB 'S1' 'S1' 'S2' 'S2' … 'S13' 'S14' 'S14'
    detector      (feature) object 4kB 'D6' 'D8' 'D5' 'D9' … 'D28' 'D25' 'D27'
  * feature       (feature) object 4kB MultiIndex
  * feature_type  (feature) object 4kB 'slope' 'slope' 'slope' … 'auc' 'auc'
  * chromo        (feature) <U3 5kB 'HbO' 'HbO' 'HbO' … 'HbR' 'HbR' 'HbR'
  * channel       (feature) object 4kB 'S1D6' 'S1D8' … 'S14D25' 'S14D27'
```

Training and Evaluating a LDA classifier

For each cross-validation fold, extract features, train the classifier and estimate classification accuracy.

[52]:
```
accuracies = []
```

```python
for epochs in cv_folds:
    # extract features
    X = mlutils.features.epoch_features(
        epochs.sel(chromo="HbO"), # HbO only
        feature_types=["slope", "max", "mean"],
        reltime_slices={
            "slope": slice(0, 9),
            "mean": slice(3, 10),
            "max": slice(2, 8),
        },
    )

    # separate train and test sets
    X_train = X[X.is_train]
    y_train = X_train.y
    X_test = X[X.is_test]
    y_test = X_test.y

    # train a LDA classifier
    clf = LinearDiscriminantAnalysis(n_components=1, solver='lsqr', shrinkage="auto")
    clf.fit(X_train, y_train)

    # evaluate perfomance
    accuracy = clf.score(X_test, y_test)
    accuracies.append(accuracy)
    print(f"#train: {len(y_train)} #test: {len(y_test)} #features: {X_train.shape[1]}␣
↪accuracy: {accuracy}")

print()
print(rf"average accuracy over cross-validation splits: {np.mean(accuracies):.3f} ±␣
↪{np.std(accuracies):.3f}")
```

```
#train: 48 #test: 16 #features: 132 accuracy: 0.8125
#train: 48 #test: 16 #features: 132 accuracy: 0.8125

#train: 48 #test: 16 #features: 132 accuracy: 0.6875
#train: 48 #test: 16 #features: 132 accuracy: 0.8125

average accuracy over cross-validation splits: 0.781 ± 0.054
```

What did it learn?

One way of estimating feature importance is the sklearn function `permuatation_importance`. It permutes feature columns and assesses the effect on the classification accuracy.

[53]:
```python
result = permutation_importance(
    clf, X_test, y_test, scoring="accuracy", n_repeats=10, random_state=0
)

importances = result.importances_mean
importance_normalized = importances / importances.sum()
```

The returned metric highlights only a few important features:

[54]:
```
plt.figure(figsize=(10,4))
plt.plot(importance_normalized)
plt.xlabel("feature")
plt.ylabel("normalized permuation importance");
```

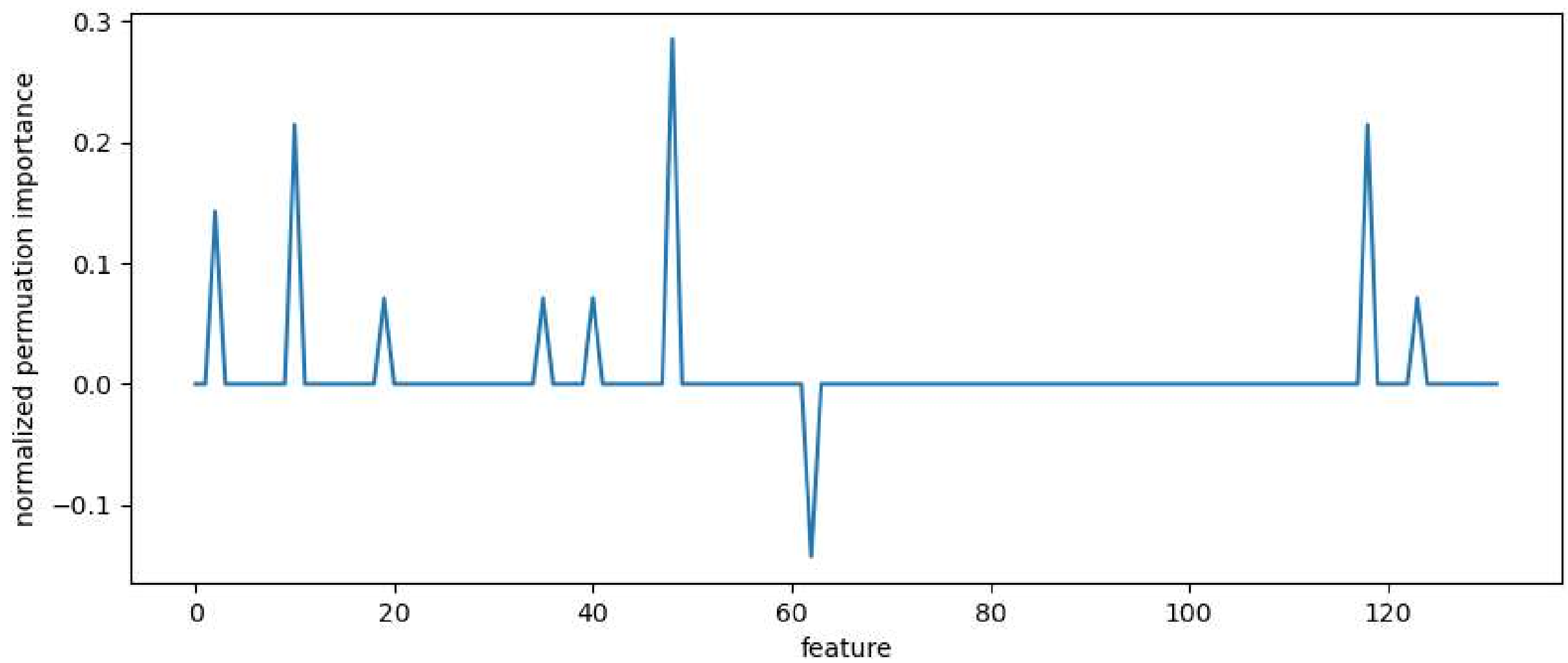

Use the preserved coordinates to identify these features:

[55]:
```
X_train.feature[importance_normalized != 0]
```

[55]:
```
<xarray.DataArray 'feature' (feature: 9)> Size: 72B
MultiIndex
Coordinates:
    chromo        <U3 12B 'HbO'
    source        (feature) object 72B 'S2' 'S4' 'S6' 'S11' … 'S6' 'S11' 'S11'
    detector      (feature) object 72B 'D5' 'D7' 'D12' … 'D8' 'D18' 'D31'
  * feature       (feature) object 72B MultiIndex
  * feature_type  (feature) object 72B 'slope' 'slope' 'slope' … 'mean' 'mean'
  * channel       (feature) object 72B 'S2D5' 'S4D7' … 'S11D18' 'S11D31'
```

By visualizing the feature distributions and the block-averaged epochs of the corresponding channels, we can observe that the HRFs in these channels, as well as the derived features, indeed have discriminative power.

[56]:
```
important_features = X_train.feature[importance_normalized != 0].values

f, ax = plt.subplots(2, len(important_features), figsize=(16,6))

for i, ftr in enumerate(important_features):
    xx_train = X_train.loc[:, ftr]
    xx_test = X_test.loc[:, ftr]
    bins = np.linspace(xx_train.min().item(),xx_train.max().item(), 11)

    ax[0,i].hist(xx_train[xx_train.trial_type == "Motor/Left"], bins,fc="r",␣
 ↪label="Motor/Left", alpha=.5)
```

```
    ax[0,i].hist(xx_train[xx_train.trial_type == "Motor/Right"], bins, fc="g", label="Motor/Right", alpha=.5)

    ax[1,i].hist(xx_test[xx_test.trial_type == "Motor/Left"], bins,fc="r", label="Motor/Left", alpha=.5)
    ax[1,i].hist(xx_test[xx_test.trial_type == "Motor/Right"], bins, fc="g", label="Motor/Right", alpha=.5)

    ax[1,i].set_xlabel(str(ftr))

ax[0,0].set_ylabel("# trials")
ax[1,0].set_ylabel("# trials")
f.suptitle("Top: Train | Bottom: Test | Red: 'Motor/Left' | Green: 'Motor/Right'");
plt.tight_layout()

f, ax = plt.subplots(1, len(important_features), figsize=(16,3))
for i, ftr in enumerate(important_features):
    ax[i].plot(blockaverage.reltime, blockaverage.sel(channel=ftr[1], chromo="HbO", trial_type="Motor/Left"), "r-")
    ax[i].plot(blockaverage.reltime, blockaverage.sel(channel=ftr[1], chromo="HbR", trial_type="Motor/Left"), "b-")
    ax[i].plot(blockaverage.reltime, blockaverage.sel(channel=ftr[1], chromo="HbO", trial_type="Motor/Right"), "r--")
    ax[i].plot(blockaverage.reltime, blockaverage.sel(channel=ftr[1], chromo="HbR", trial_type="Motor/Right"), "b--")
    ax[i].set_ylim(-.2,.2)
    ax[i].grid()
    ax[i].set_title(ftr)
    ax[i].set_xlabel("$t_{rel}$ / s")

ax[0].set_ylabel(r"$\Delta$ concentration / µM")
f.suptitle("Red: HbO | Blue: HbR | - 'Motor/Left' | -- 'Motor/Right'");
plt.tight_layout()
```

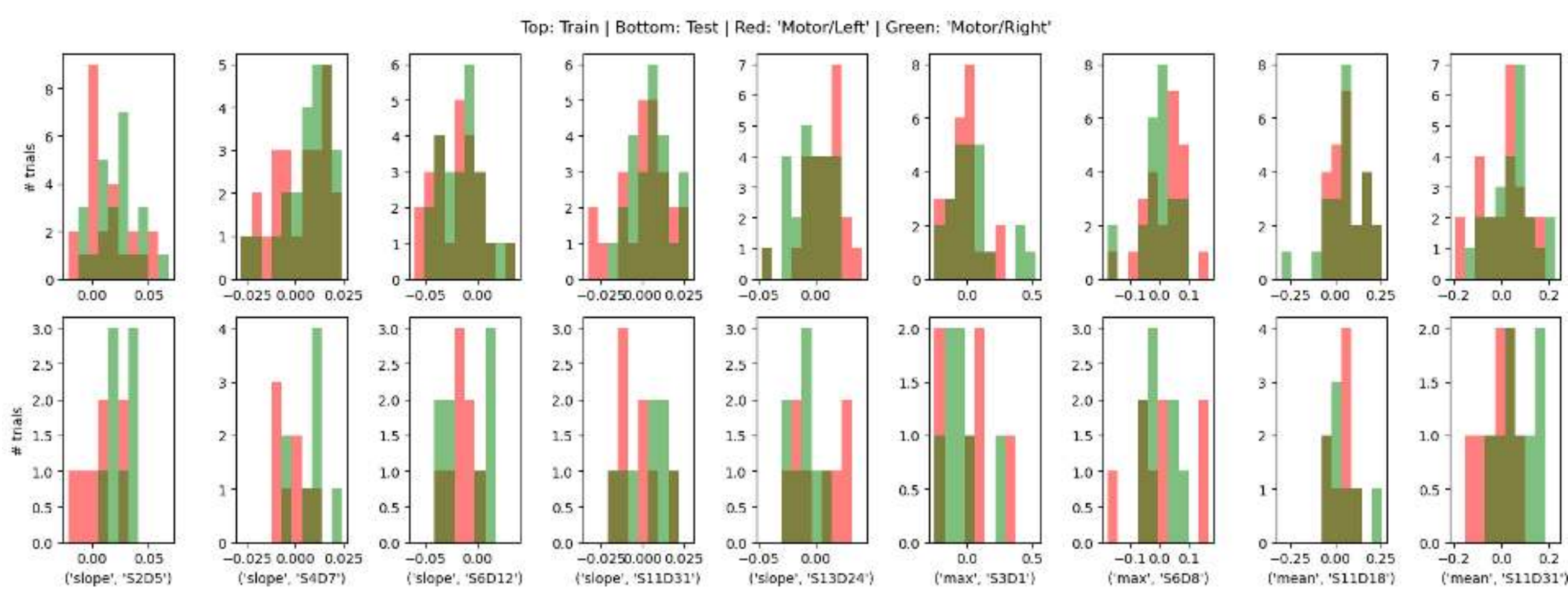

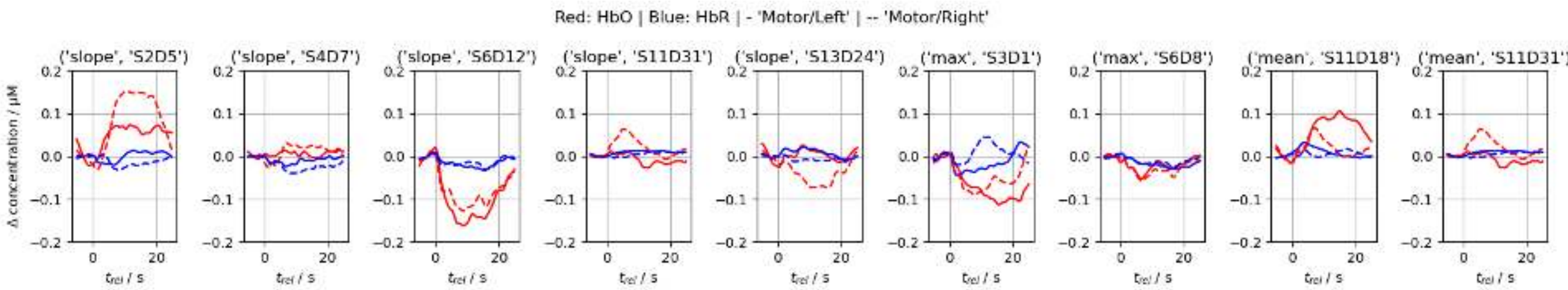

References

[57]:
```
cedalion.bib.dump_to_notebook()
```

<IPython.core.display.HTML object>

Next: 7 — Data Augmentation — generating synthetic data.

S7: Data Augmentation

This example notebook illustrates the functionality in `cedalion.sim.synthetic_hrf` and `cedalion.sim.synthetic_artifact` to create simulated datasets with added activations and motion artifacts.

It has two parts:

1. Adding synthetic activations
2. Adding synthetic motion artifacts

Learning objectives

In this notebook you will learn to:

- Generate synthetic HRF-modulated fNIRS signals with controllable parameters
- Add realistic motion artefacts to clean recordings
- Use augmented data for algorithm benchmarking with known ground truth

[1]:
```
# This cells setups the environment when executed in Google Colab.
try:
    import google.colab
    !curl -s https://raw.githubusercontent.com/ibs-lab/cedalion/dev/scripts/
↪colab_setup.py -o colab_setup.py
    # Select branch with --branch "branch name" (default is "dev")
    %run colab_setup.py
except ImportError:
    pass
```

[2]:
```
# set this flag to True to enable interactive 3D plots
INTERACTIVE_PLOTS = False
```

[3]:
```
import matplotlib.pyplot as plt
import numpy as np
import pyvista as pv
import xarray as xr

import cedalion
import cedalion.dataclasses as cdc
import cedalion.data
import cedalion.geometry.landmarks as cd_landmarks
import cedalion.dot as dot
import cedalion.models.glm as glm
import cedalion.nirs

import cedalion.vis.anatomy
import cedalion.vis.blocks as vbx

import cedalion.sigproc.quality as quality
import cedalion.sim.synthetic_hrf as synhrf
import cedalion.sim.synthetic_artifact as synart
import cedalion.xrutils as xrutils
from cedalion import units
from IPython.display import Image
```

```
xr.set_options(display_expand_data=False)

if INTERACTIVE_PLOTS:
    pv.set_jupyter_backend('server')
else:
    pv.set_jupyter_backend('static')
```

[4]:
```
# helper function to display gifs in rendered notbooks
def display_image(fname : str):
    display(Image(data=open(fname,'rb').read(), format='png'))
```

Adding Synthetic Activations

Loading and preprocessing the dataset

This notebook uses a high-density, whole head resting state dataset recorded with a NinjaNIRS 22.

[5]:
```
rec = cedalion.data.get_nn22_resting_state()

geo3d = rec.geo3d
meas_list = rec._measurement_lists["amp"]

amp = rec["amp"]
amp = amp.pint.dequantify().pint.quantify("V")

display(amp)
```

```
<xarray.DataArray (channel: 567, wavelength: 2, time: 3309)> Size: 30MB
[V] 0.0238 0.02385 0.02375 0.02361 0.02364 … 0.2887 0.2892 0.2897 0.291 0.2923
Coordinates:
  * time         (time) float64 26kB 0.0 0.1112 0.2225 … 367.8 367.9 368.0
    samples      (time) int64 26kB 0 1 2 3 4 5 … 3303 3304 3305 3306 3307 3308
  * channel      (channel) object 5kB 'S10D87' 'S10D94' … 'S47D29' 'S5D137'
    source       (channel) object 5kB 'S10' 'S10' 'S10' … 'S56' 'S47' 'S5'
    detector     (channel) object 5kB 'D87' 'D94' 'D89' … 'D129' 'D29' 'D137'
  * wavelength   (wavelength) float64 16B 760.0 850.0
Attributes:
    data_type_group:  unprocessed raw
```

[6]:
```
cedalion.vis.anatomy.plot_montage3D(rec["amp"], geo3d)
```

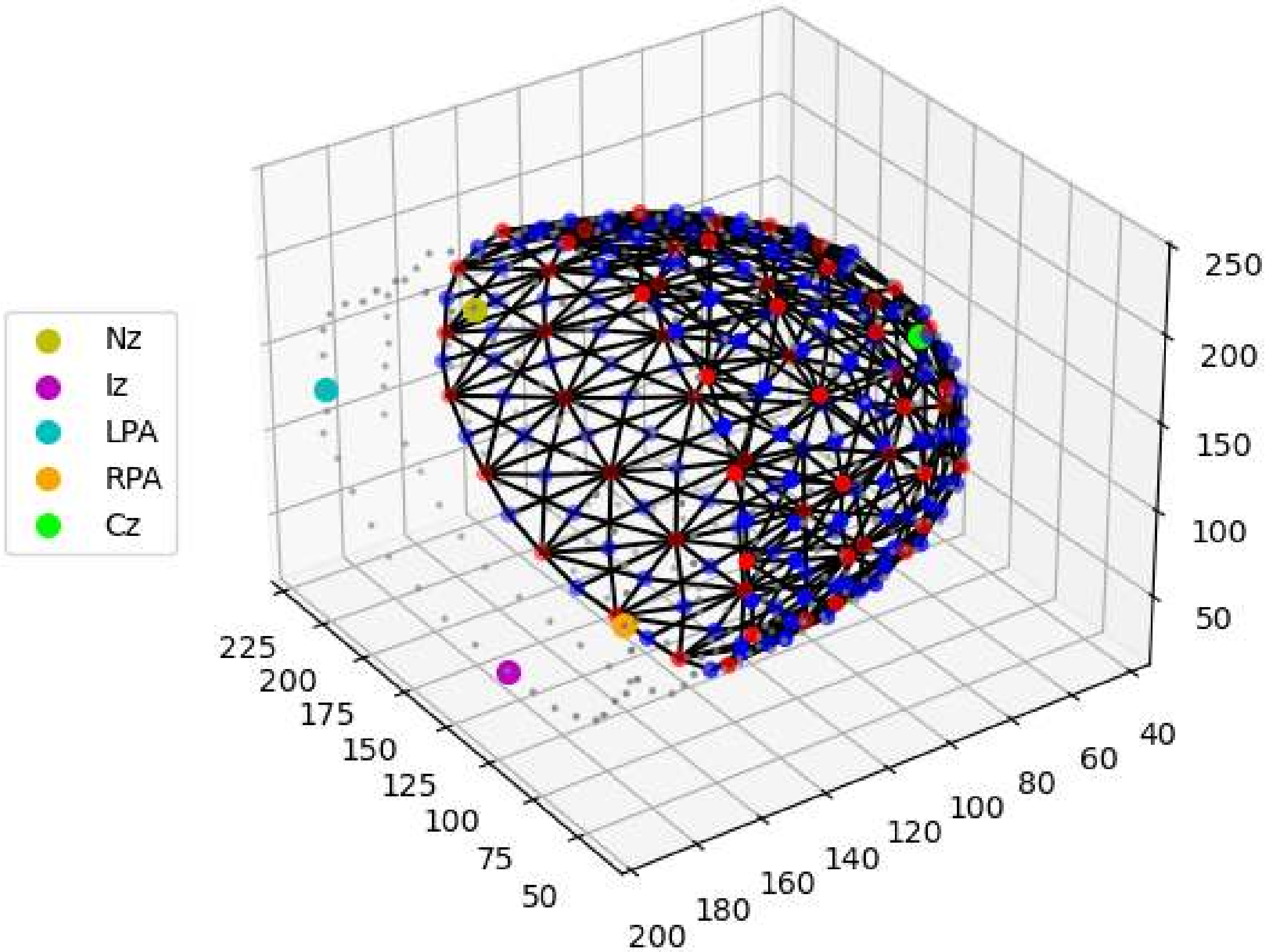

[7]:
```
# Select channels which have at least a signal-to-noise ratio of 10
snr_thresh = 10  # the SNR (std/mean) of a channel.
snr, snr_mask = quality.snr(rec["amp"], snr_thresh)
amp_selected, masked_channels = xrutils.apply_mask(
    rec["amp"], snr_mask, "drop", "channel"
)

print(f"Removed {len(masked_channels)} channels because of low SNR.")
```

```
Removed 22 channels because of low SNR.
```

[8]:
```
# Calculate optical density
od = cedalion.nirs.cw.int2od(amp_selected)
```

Construct headmodel

We load the the Colin27 headmodel, since we need the geometry for image reconstruction.

[9]:
```
head_ijk = dot.get_standard_headmodel("colin27")

# transform coordinates to a RAS coordinate system
head_ras = head_ijk.apply_transform(head_ijk.t_ijk2ras)
```

[10]:
```
display(head_ijk.brain)
display(head_ras.brain)
```

```
TrimeshSurface(faces: 49992 vertices: 25000 crs: ijk units: dimensionless vertex_coords:␣
↪['fsaverage_vertex', 'parcel', 'mni152_r', 'mni152_a', 'mni152_s'])
```

```
TrimeshSurface(faces: 49992 vertices: 25000 crs: mni units: millimeter vertex_coords:␣
↪['fsaverage_vertex', 'parcel', 'mni152_r', 'mni152_a', 'mni152_s'])
```

[11]:
```
head_ras.landmarks
```

[11]:
```
<xarray.DataArray (label: 73, mni: 3)> Size: 2kB
[mm] 0.0045 -118.6 -23.08 -86.08 -19.99 … -100.4 37.02 49.81 -99.34 21.47
Coordinates:
  * label    (label) <U3 876B 'Iz' 'LPA' 'RPA' 'Nz' … 'PO1' 'PO2' 'PO4' 'PO6'
    type     (label) object 584B PointType.LANDMARK … PointType.LANDMARK
Dimensions without coordinates: mni
```

We want to build the synthetic HRFs at C3 and C4 (green dots in the image below)

[12]:
```
plt_pv = pv.Plotter()
vbx.plot_surface(plt_pv, head_ras.brain, color="#d3a6a1")
vbx.plot_surface(plt_pv, head_ras.scalp, opacity=0.1)
vbx.plot_labeled_points(
    plt_pv, head_ras.landmarks.sel(label=["C3", "C4"]), show_labels=True
)
vbx.camera_at_cog(plt_pv, head_ras.brain, (-400,500,400), fit_scene=True)

plt_pv.show()
```

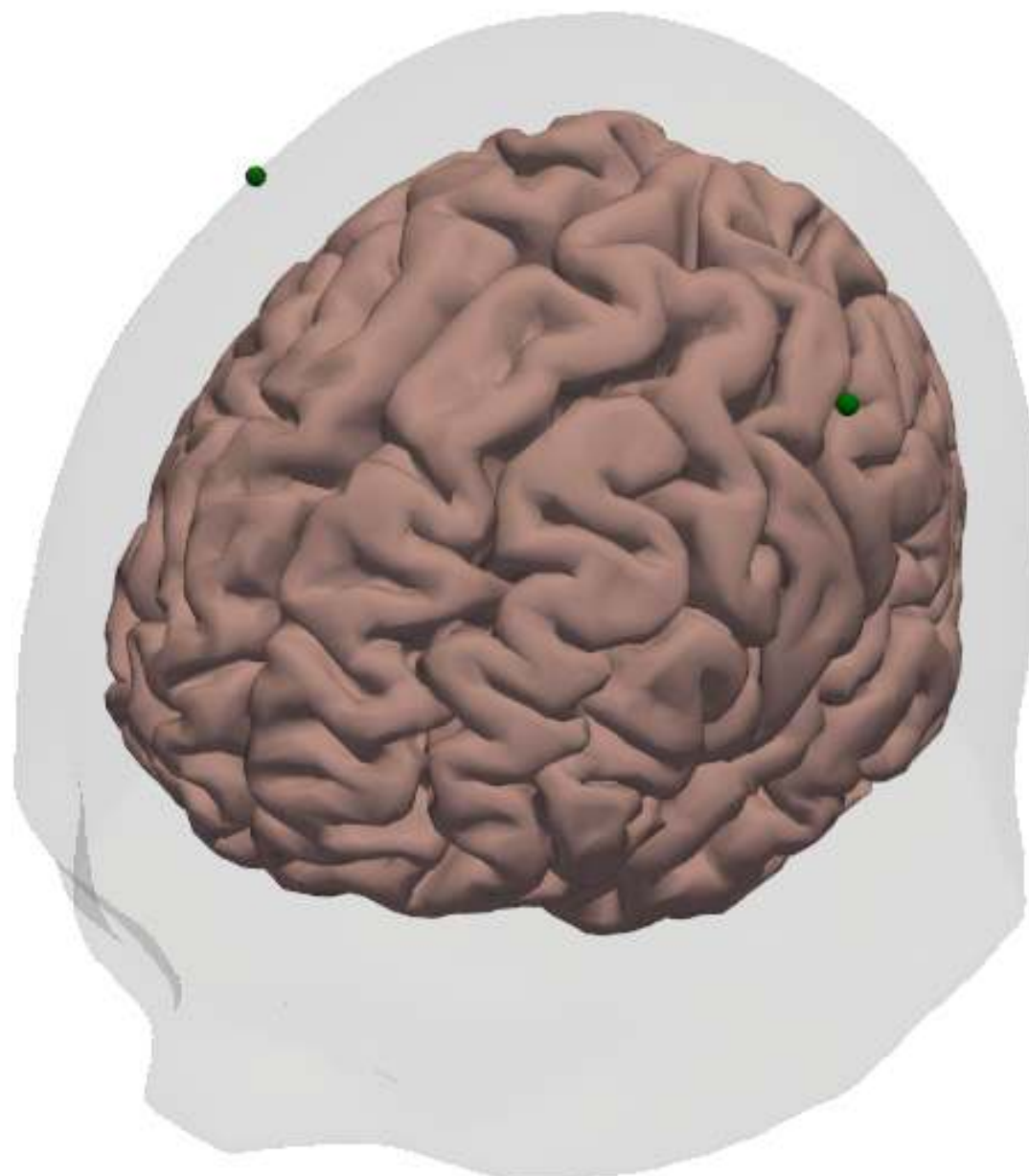

Build spatial activation pattern on brain surface for landmarks C3 and C4

The function `build_spatial_activation` is used to place a spatial activation pattern on the brain surface. The activation pattern is a Gaussian function of the geodesic distance to a seed vertex. Hence, the size of the activation is determined by the standard deviation of this Gaussian, specified by the parameter `spatial_scale`. The peak intensity in HbO is determined by the parameter `intensity_scale`. The intensity of HbR activation is specified relative to the HbO peak intensity. So if the HbO pattern describes an increase in Hbo then providing a negative factor smaller than 1 yields a decrease in HbR with smaller amplitude. The seed vertex (integer) can be selected as the closest vertex to a given landmark:

[13]:
```
# obtain the closest vertices to C3 and C4
_, c3_seed = head_ras.brain.mesh.kdtree.query(
    head_ras.landmarks.sel(label="C3").pint.magnitude
)
_, c4_seed = head_ras.brain.mesh.kdtree.query(
    head_ras.landmarks.sel(label="C4").pint.magnitude
)
```

[14]:
```
# create the spatial activation
spatial_act = synhrf.build_spatial_activation(
    head_ras.brain,
    c3_seed,
    spatial_scale=2 * cedalion.units.cm,
```

```
    intensity_scale=1 * units.micromolar,
    hbr_scale=-0.4,
)
```

The resulting `DataArray` contains an activation value for each vertex and chromophore on the brain surface.

[15]:
```
display(spatial_act)
```

```
<xarray.DataArray (vertex: 25000, chromo: 2)> Size: 400kB
[M] 1.303e-29 -5.211e-30 3.318e-30 -1.327e-30 9.361e-31 … 0.0 -0.0 0.0 -0.0
Coordinates:
  * chromo   (chromo) <U3 24B 'HbO' 'HbR'
Dimensions without coordinates: vertex
```

[16]:
```
f,ax = plt.subplots(1,2,figsize=(10,5))
cedalion.vis.anatomy.plot_brain_in_axes(
        od,
        head_ras.landmarks,
        spatial_act.sel(chromo="HbO").pint.to("uM"),
        head_ras.brain,
        ax[0],
        camera_pos="C3",
        cmap="RdBu_r",
        vmin=-1,
        vmax=+1,
        cb_label=r"$\Delta$ HbO / µM",
        title=None,
    )
ax[0].set_title("HbO")
cedalion.vis.anatomy.plot_brain_in_axes(
        od,
        head_ras.landmarks,
        spatial_act.sel(chromo="HbR").pint.to("uM"),
        head_ras.brain,
        ax[1],
        camera_pos="C3",
        cmap="RdBu_r",
        vmin=-1,
        vmax=+1,
        cb_label=r"$\Delta$ HbR / µM",
        title=None,
    )
ax[1].set_title("HbR");
```

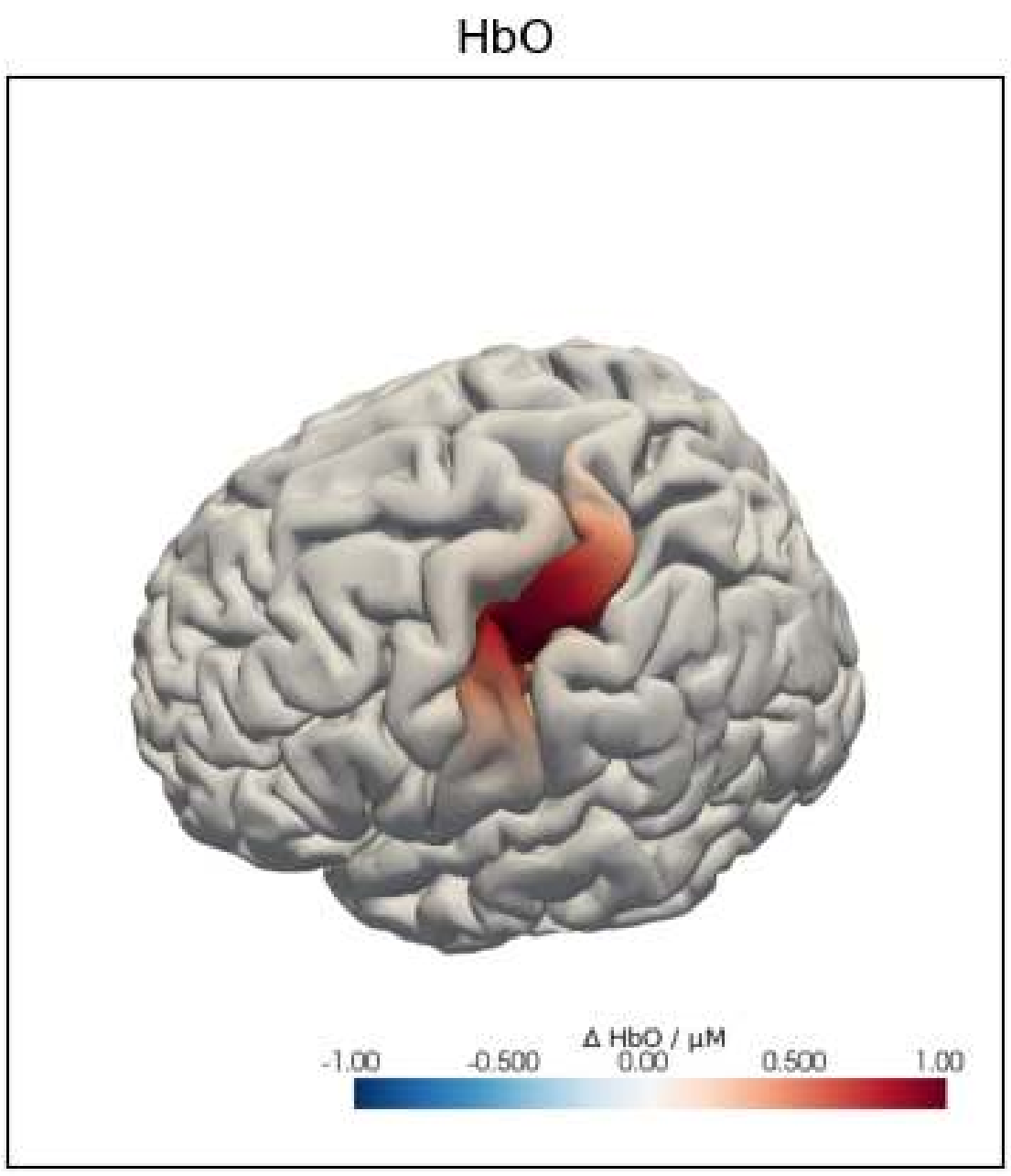

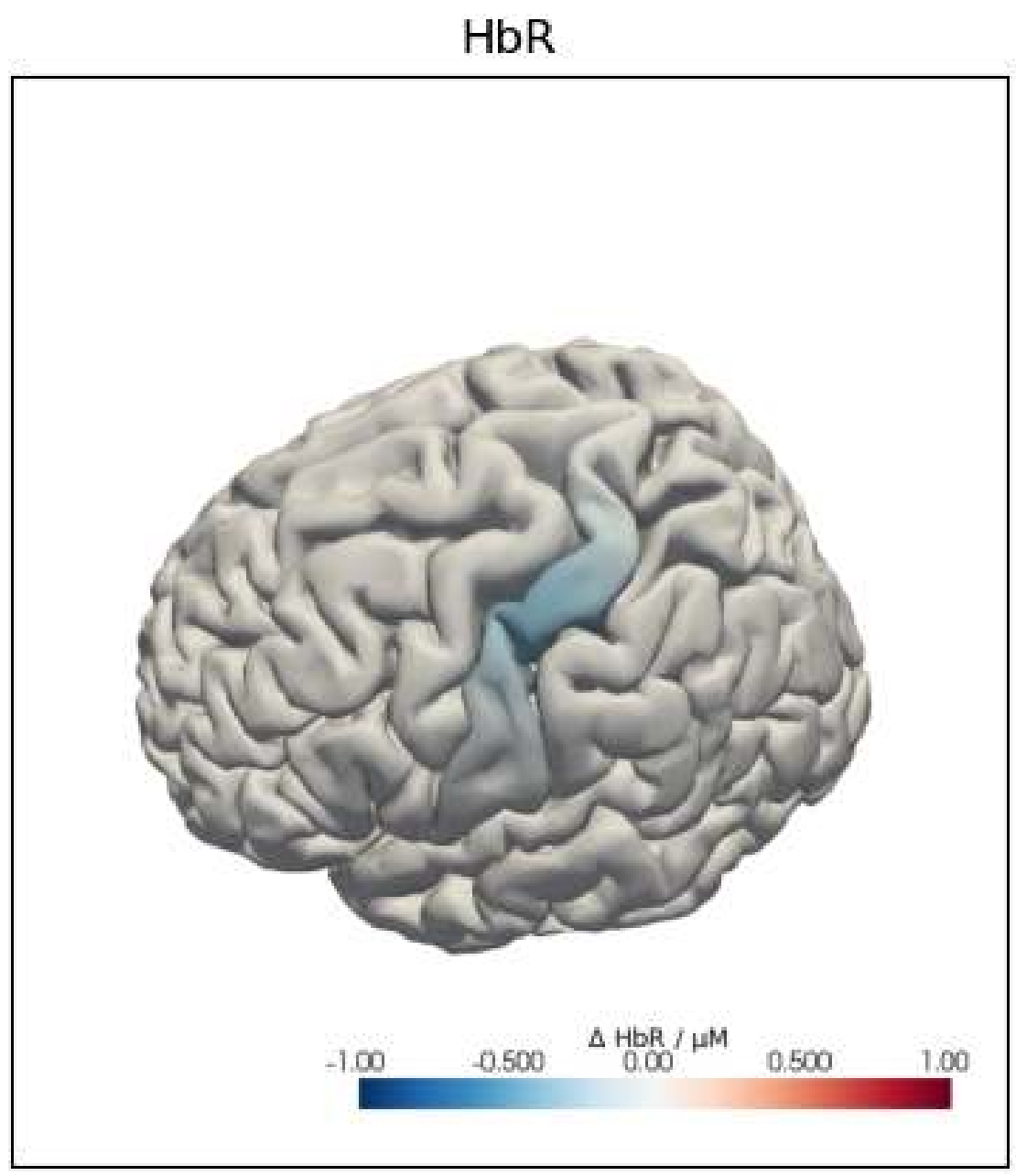

The following plot illustrates the effects of the `spatial_scale` and `intensity_scale` parameters:

[17]:
```
f, ax = plt.subplots(2, 3, figsize=(9, 6))
for i, spatial_scale in enumerate([0.5 * units.cm, 2 * units.cm, 3 * units.cm]):
    spatial_act = synhrf.build_spatial_activation(
        head_ras.brain,
        c3_seed,
        spatial_scale=spatial_scale,
        intensity_scale=1 * units.micromolar,
        hbr_scale=-0.4,
    )

    cedalion.vis.anatomy.plot_brain_in_axes(
        od,
        head_ras.landmarks,
        spatial_act.sel(chromo="HbO").pint.to("uM"),
        head_ras.brain,
        ax[0, i],
        camera_pos="C3",
        cmap="RdBu_r",
        vmin=-1,
        vmax=+1,
        cb_label=r"$\Delta$ HbO / µM",
        title=None,
    )
    ax[0, i].set_title(f"spatial_scale: {spatial_scale.magnitude} cm")

for i, intensity_scale in enumerate(
    [
        0.5 * units.micromolar,
```

```
        1.0 * units.micromolar,
        2.0 * units.micromolar,
    ]
):
    spatial_act = synhrf.build_spatial_activation(
        head_ras.brain,
        c3_seed,
        spatial_scale=2 * units.cm,
        intensity_scale=intensity_scale,
        hbr_scale=-0.4,
    )

    cedalion.vis.anatomy.plot_brain_in_axes(
        od,
        head_ras.landmarks,
        spatial_act.sel(chromo="HbO").pint.to("uM"),
        head_ras.brain,
        ax[1, i],
        camera_pos="C3",
        cmap="RdBu_r",
        vmin=-2,
        vmax=+2,
        cb_label=r"$\Delta$ HbO / µM",
        title=None,
    )
    ax[1, i].set_title(f"intensity_scale: {intensity_scale.magnitude} µM")

f.tight_layout()
```

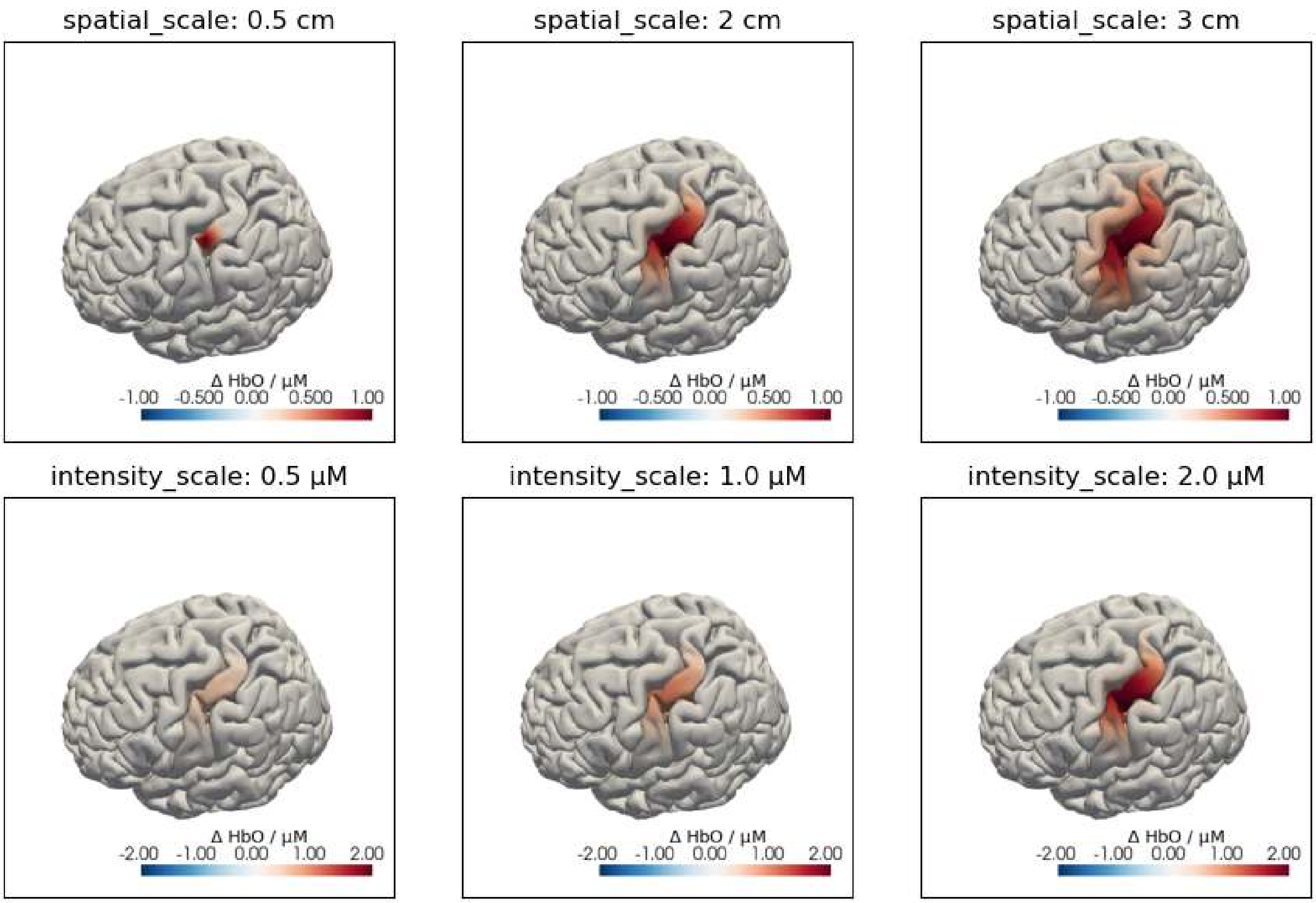

For this example notebook two activations are placed below C3 and C4:

[18]:
```
spatial_act_c3 = synhrf.build_spatial_activation(
    head_ras.brain,
    c3_seed,
    spatial_scale=2 * cedalion.units.cm,
    intensity_scale=1 * units.micromolar,
    hbr_scale=-0.4,
)
spatial_act_c4 = synhrf.build_spatial_activation(
    head_ras.brain,
    c4_seed,
    spatial_scale=2 * cedalion.units.cm,
    intensity_scale=1 * units.micromolar,
    hbr_scale=-0.4,
)
```

We concatenate the two images for C3 and C4 along dimension `trial_type` to get a single `DataArray` with the spatial information for both landmarks.

[19]:
```
# concatenate the two spatial activations along a new dimension
spatial_imgs = xr.concat(
    [spatial_act_c3, spatial_act_c4], dim="trial_type"
).assign_coords(trial_type=["Stim C3", "Stim C4"])
spatial_imgs
```

[19]:
```
<xarray.DataArray (trial_type: 2, vertex: 25000, chromo: 2)> Size: 800kB
[M] 1.303e-29 -5.211e-30 3.318e-30 … -3.621e-22 7.069e-35 -2.828e-35
Coordinates:
  * chromo      (chromo) <U3 24B 'HbO' 'HbR'
  * trial_type  (trial_type) <U7 56B 'Stim C3' 'Stim C4'
Dimensions without coordinates: vertex
```

Plots of spatial patterns

Using the helper function `cedalion.vis.anatomy.plot_brain_in_axes`, the created activations on the brain surface below C3 and C4 are plotted:

[20]:
```
f, ax = plt.subplots(1,2, figsize=(10,5))

cedalion.vis.anatomy.plot_brain_in_axes(
    od,
    head_ras.landmarks,
    spatial_imgs.sel(trial_type="Stim C3", chromo="HbO").pint.to("uM"),
    head_ras.brain,
    ax[0],
    camera_pos="C3",
    cmap="RdBu_r",
    vmin=-1,
    vmax=+1,
    cb_label=r"$\Delta$ HbO / µM",
    title="C3 Activation",
)

cedalion.vis.anatomy.plot_brain_in_axes(
    od,
    head_ras.landmarks,
    spatial_imgs.sel(trial_type="Stim C4", chromo="HbO").pint.to("uM"),
    head_ras.brain,
    ax[1],
    camera_pos="C4",
    cmap="RdBu_r",
    vmin=-1,
    vmax=+1,
    cb_label=r"$\Delta$ HbO / µM",
    title="C4 Activation",
)

f.tight_layout()
```

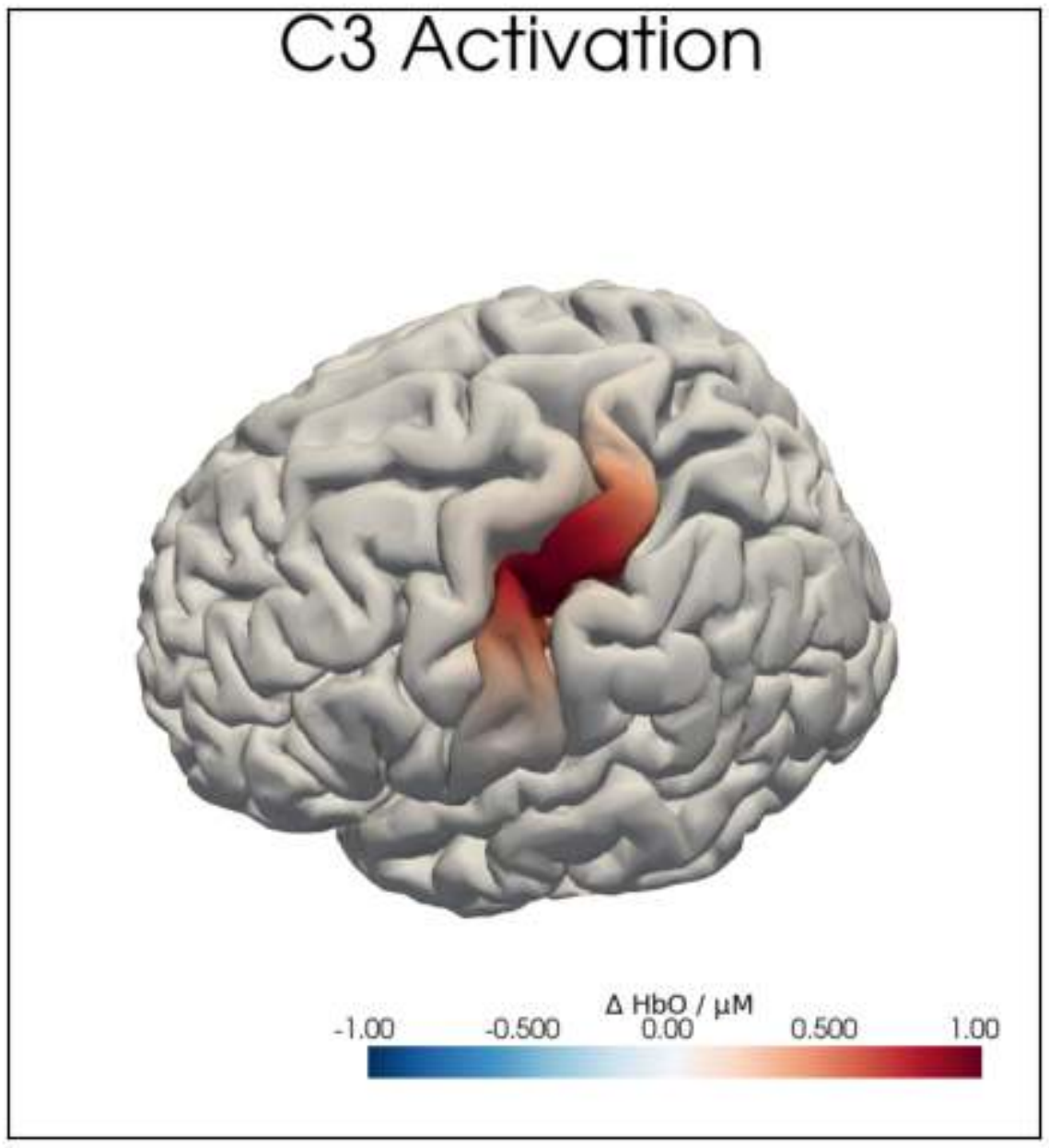

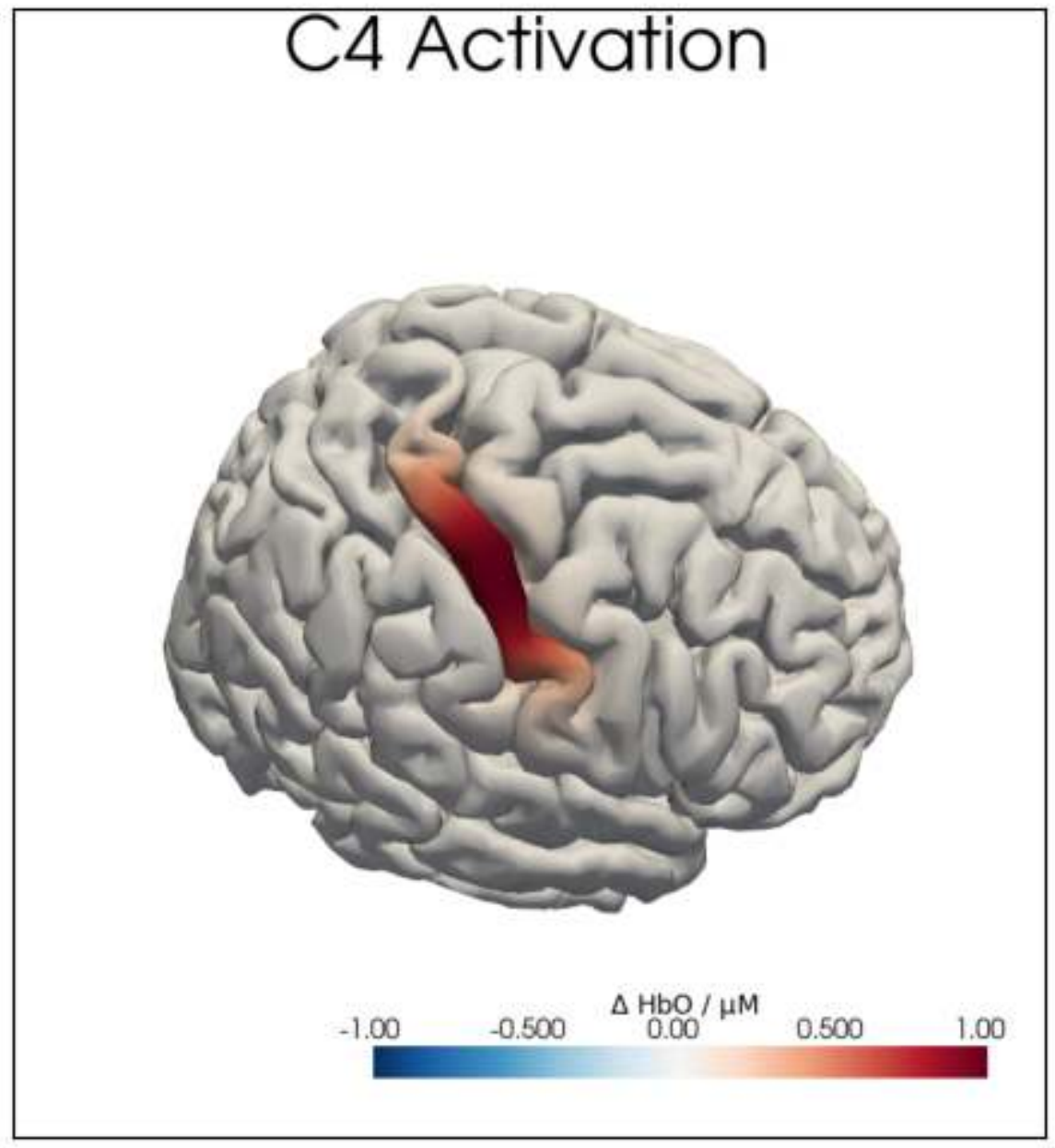

Image Reconstruction

We load the precomputed Adot matrix to be able to map from image to channel space. (For details see S5 image reconstruction tutorial).

[21]:
```
Adot = cedalion.data.get_precomputed_sensitivity("nn22_resting", "colin27")
```

[22]:
```
# we only consider brain vertices, not scalp and drop pruned channels
Adot_brain = Adot.sel(vertex=Adot.is_brain, channel=od.channel)
Adot_brain
```

[22]:
```
<xarray.DataArray (channel: 545, vertex: 25000, wavelength: 2)> Size: 109MB
1.038e-18 1.038e-18 2.69e-18 2.69e-18 … 5.42e-13 5.42e-13 1.069e-22 1.069e-22
Coordinates:
    parcel      (vertex) object 200kB 'VisCent_ExStr_8_LH' … 'VisCent_ExStr…
    is_brain    (vertex) bool 25kB True True True True … True True True True
  * channel     (channel) object 4kB 'S10D87' 'S10D94' … 'S47D29' 'S5D137'
    detector    (channel) object 4kB 'D87' 'D94' 'D89' … 'D129' 'D29' 'D137'
    source      (channel) object 4kB 'S10' 'S10' 'S10' … 'S56' 'S47' 'S5'
  * wavelength  (wavelength) float64 16B 760.0 850.0
Dimensions without coordinates: vertex
Attributes:
    units:    mm
```

The forward model and image reconstruction transform time series in image space into channel space, and vice versa. In the present case, we need to translate between optical-density time series of different wavelengths in channel space and concentration time series of different chromophores in image space. This is facilitated by the function `dot.forward_model.image_to_channel_space`:

[23]:
```
# propagate concentration changes in the brain to optical density
# changes in channel space
```

```
spatial_chan = dot.forward_model.image_to_channel_space(
    Adot_brain, spatial_imgs, spectrum="prahl"
)
spatial_chan
```

[23]:
```
<xarray.DataArray (trial_type: 2, wavelength: 2, channel: 545)> Size: 17kB
[] -6.863e-13 -1.992e-11 -1.644e-11 -5.69e-14 … 1.434e-06 2.526e-08 2.487e-08
Coordinates:
  * wavelength  (wavelength) float64 16B 760.0 850.0
  * channel     (channel) object 4kB 'S10D112' 'S10D133' … 'S9D94' 'S9D96'
  * trial_type  (trial_type) <U7 56B 'Stim C3' 'Stim C4'
    source      (channel) object 4kB 'S10' 'S10' 'S10' 'S10' … 'S9' 'S9' 'S9'
    detector    (channel) object 4kB 'D112' 'D133' 'D135' … 'D92' 'D94' 'D96'
```

Show the spatial activation in channel space with a scalp plot.

[24]:
```
fig, ax = plt.subplots(1, 2)
# adjust plot size
fig.set_size_inches(12, 6)
cedalion.vis.anatomy.scalp_plot(
    od,
    rec.geo3d,
    spatial_chan.sel(trial_type="Stim C3", wavelength=850),
    ax[0],
    cmap="YlOrRd",
    title="850nm, activation under C3",
    vmin=spatial_chan.min().item(),
    vmax=spatial_chan.max().item(),
    cb_label="max peak amplitude",
)
cedalion.vis.anatomy.scalp_plot(
    od,
    rec.geo3d,
    spatial_chan.sel(trial_type="Stim C4", wavelength=850),
    ax[1],
    cmap="YlOrRd",
    title="850nm, activation under C4",
    vmin=spatial_chan.min().item(),
    vmax=spatial_chan.max().item(),
    cb_label="Max peak amplitude",
)
plt.show()
```

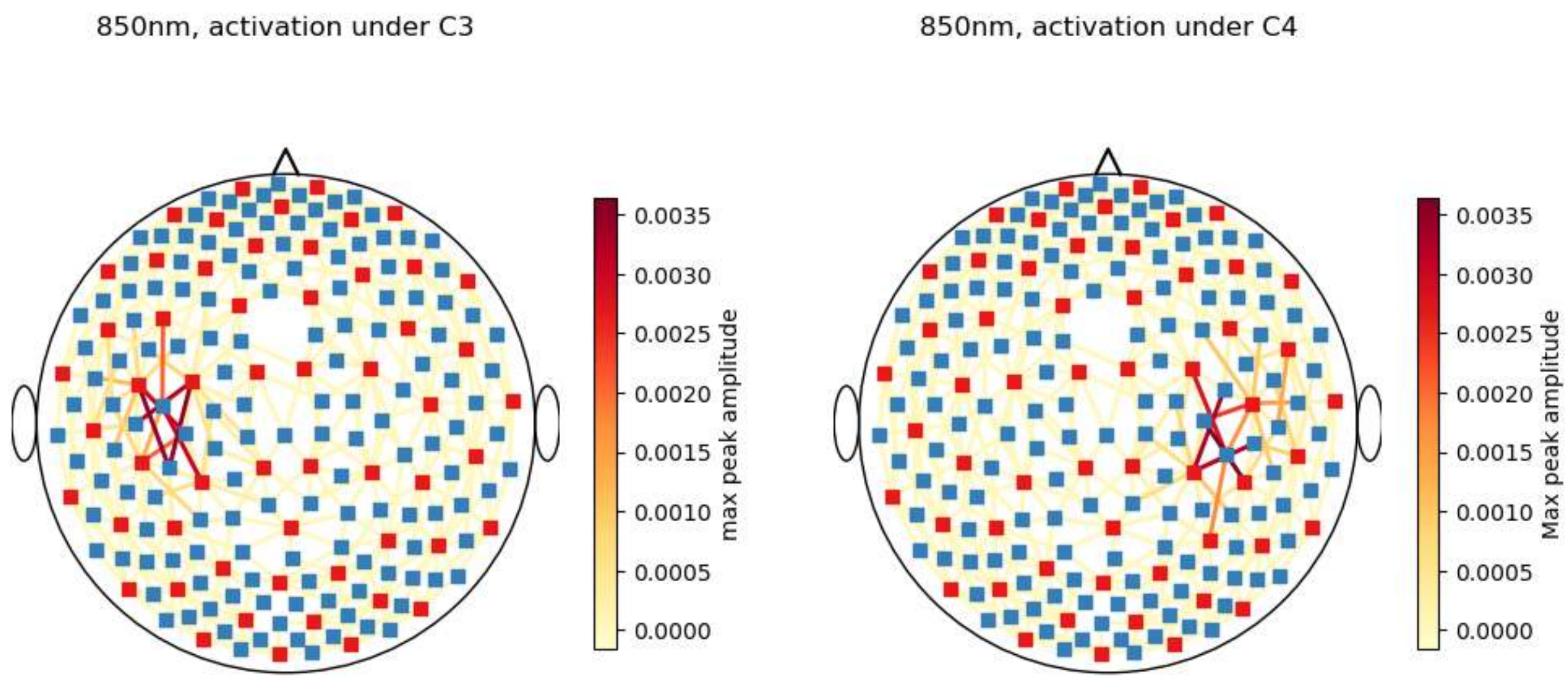

Get top 5 channels for each trial type where synthetic activation is highest

[25]:
```
roi_chans_c3 = spatial_chan.channel[
    spatial_chan.sel(trial_type="Stim C3").max("wavelength").argsort()[-5:].values
].values
roi_chans_c4 = spatial_chan.channel[
    spatial_chan.sel(trial_type="Stim C4").max("wavelength").argsort()[-5:].values
].values
```

Concentration Scale

The activations were simulataed in image space with a peak concentration change of 1 µM in one vertex. The change in optical density in one channel reflects concentration changes in the ensemble of vertices that this channel is sensitive to.

When applying the Beer-Lambert-transformation in channel space, a change in concentration is calculated for each channel. However, the scales of these concentration changes and the concentration changes in single vertices are not the same.

Here, a correction factor is calculated to scale the activation in channel space to 1uM.

[26]:
```
dpf = xr.DataArray(
    [6, 6],
    dims="wavelength",
    coords={"wavelength": rec["amp"].wavelength},
)

# add time axis with one time point so we can convert to conc
spatial_chan_w_time = spatial_chan.expand_dims("time")
spatial_chan_w_time = spatial_chan_w_time.assign_coords(time=[0])
spatial_chan_w_time.time.attrs["units"] = "second"
display(spatial_chan_w_time)
```

```
spatial_chan_conc = cedalion.nirs.cw.od2conc(
    spatial_chan_w_time, geo3d, dpf, spectrum="prahl"
)
```

```
<xarray.DataArray (time: 1, trial_type: 2, wavelength: 2, channel: 545)> Size: 17kB
[] -6.863e-13 -1.992e-11 -1.644e-11 -5.69e-14 … 1.434e-06 2.526e-08 2.487e-08
Coordinates:
  * wavelength  (wavelength) float64 16B 760.0 850.0
  * channel     (channel) object 4kB 'S10D112' 'S10D133' … 'S9D94' 'S9D96'
  * trial_type  (trial_type) <U7 56B 'Stim C3' 'Stim C4'
    source      (channel) object 4kB 'S10' 'S10' 'S10' 'S10' … 'S9' 'S9' 'S9'
    detector    (channel) object 4kB 'D112' 'D133' 'D135' … 'D92' 'D94' 'D96'
  * time        (time) int64 8B 0
```

[27]:
```
# rescale so that synthetic hrfs add 1 micromolar at peak.
rescale_factor = (1* units.micromolar / spatial_chan_conc.max()).item()

display(rescale_factor)
spatial_chan *= rescale_factor
```

) (dimensionless

HRFs in channel space

So far the notebook focused on the spatial extent of the activation. To build the temporal HRF model we use the same functionality that generates hrf regressors for the GLM.

First we select a basis function, which defines the temporal shape of the HRF.

[28]:
```
basis_fct = glm.Gamma(tau=0 * units.s, sigma=3 * units.s, T=3 * units.s)
```

[29]:
```
od.time
```

[29]:
```
<xarray.DataArray 'time' (time: 3309)> Size: 26kB
0.0 0.1112 0.2225 0.3337 0.445 0.5562 … 367.5 367.6 367.7 367.8 367.9 368.0
Coordinates:
  * time     (time) float64 26kB 0.0 0.1112 0.2225 0.3337 … 367.8 367.9 368.0
    samples  (time) int64 26kB 0 1 2 3 4 5 6 … 3303 3304 3305 3306 3307 3308
Attributes:
    units:    second
```

A Stim DataFrame, which contains the onset, duration and amplitude of the synthetic HRFs, is created.

[30]:
```
stim_df = synhrf.build_stim_df(
    max_time=od.time.values[-1] * units.seconds,
    trial_types=["Stim C3", "Stim C4"],
    min_interval=10 * units.seconds,
    max_interval=20 * units.seconds,
    min_stim_dur = 10 * units.seconds,
    max_stim_dur = 10 * units.seconds,
    min_stim_value = 1.0,
    max_stim_value = 1.0,
    order="random",
)
```

[31]:
```python
stim_df.head()
```

[31]:

	onset	duration	value	trial_type
0	10.32	10.0	1.0	Stim C4
1	34.34	10.0	1.0	Stim C4
2	59.80	10.0	1.0	Stim C4
3	82.07	10.0	1.0	Stim C3
4	104.54	10.0	1.0	Stim C3

We can now use our stim dataframe, basis function, and spatial information to create the synthetic HRF timeseries

[32]:
```python
syn_ts = synhrf.build_synthetic_hrf_timeseries(od, stim_df, basis_fct, spatial_chan)
```

We get a synthetic HRF timeseries for each channel, trial_type and chromo / wavelength

[33]:
```python
syn_ts
```

[33]:
```
<xarray.DataArray (trial_type: 2, wavelength: 2, channel: 545, time: 3309)> Size: 58MB
[] -0.0 -0.0 -0.0 -0.0 -0.0 -0.0 -0.0 -0.0 … 0.0 0.0 0.0 0.0 0.0 0.0 0.0 0.0
Coordinates:
  * wavelength  (wavelength) float64 16B 760.0 850.0
  * channel     (channel) object 4kB 'S10D112' 'S10D133' … 'S9D94' 'S9D96'
  * trial_type  (trial_type) <U7 56B 'Stim C3' 'Stim C4'
    source      (channel) object 4kB 'S10' 'S10' 'S10' 'S10' … 'S9' 'S9' 'S9'
    detector    (channel) object 4kB 'D112' 'D133' 'D135' … 'D92' 'D94' 'D96'
  * time        (time) float64 26kB 0.0 0.1112 0.2225 … 367.8 367.9 368.0
```

Sum the synthetic timeseries over trial_type dimension, so it has the same shape as the resting state data

[34]:
```python
syn_ts_sum = syn_ts.sum(dim='trial_type')
syn_ts_sum
```

[34]:
```
<xarray.DataArray (wavelength: 2, channel: 545, time: 3309)> Size: 29MB
[] 0.0 0.0 0.0 0.0 0.0 0.0 0.0 0.0 0.0 … 0.0 0.0 0.0 0.0 0.0 0.0 0.0 0.0 0.0
Coordinates:
  * wavelength  (wavelength) float64 16B 760.0 850.0
  * channel     (channel) object 4kB 'S10D112' 'S10D133' … 'S9D94' 'S9D96'
    source      (channel) object 4kB 'S10' 'S10' 'S10' 'S10' … 'S9' 'S9' 'S9'
    detector    (channel) object 4kB 'D112' 'D133' 'D135' … 'D92' 'D94' 'D96'
  * time        (time) float64 26kB 0.0 0.1112 0.2225 … 367.8 367.9 368.0
```

Adding HRFs to measured data

Here, the simulated activations are combined with physiological noise by adding the synthetic HRFs to the resting state dataset:

[35]:
```python
# set to false to process only the synth. HRF in the following
ADD_RESTING_STATE = True

if ADD_RESTING_STATE:
    od_w_hrf = od + syn_ts_sum
else:
    od_w_hrf = syn_ts_sum
```

```python
    od_w_hrf = od_w_hrf.assign_coords(
        {
            "samples": ("time", od.samples.values),
            "time": ("time", od.time.data),
        }
    )
    od_w_hrf = od_w_hrf.pint.quantify({"time": "s"})
```

Recover the HRFs again

In the following, the added activations should be extracted from the simulated dataset again. To this end, the data is frequency filtered and block averages are calculated.

[36]:
```python
od_w_hrf_filtered = od_w_hrf.cd.freq_filter(fmin=0.02, fmax=0.5, butter_order=4)
```

[37]:
```python
od_w_hrf_filtered
```

[37]:
```
<xarray.DataArray (channel: 545, wavelength: 2, time: 3309)> Size: 29MB
[] 0.002073 0.002634 0.00317 0.003663 … 0.006999 0.006033 0.004973 0.00387
Coordinates:
  * time        (time) float64 26kB 0.0 0.1112 0.2225 … 367.8 367.9 368.0
  * channel     (channel) object 4kB 'S10D87' 'S10D94' … 'S47D29' 'S5D137'
  * wavelength  (wavelength) float64 16B 760.0 850.0
    samples     (time) int64 26kB 0 1 2 3 4 5 … 3303 3304 3305 3306 3307 3308
    source      (channel) object 4kB 'S10' 'S10' 'S10' … 'S56' 'S47' 'S5'
    detector    (channel) object 4kB 'D87' 'D94' 'D89' … 'D129' 'D29' 'D137'
```

[38]:
```python
epochs = od_w_hrf_filtered.cd.to_epochs(
    stim_df,  # stimulus dataframe
    ["Stim C3", "Stim C4"],  # select events
    before=5 * units.seconds,  # seconds before stimulus
    after=20 * units.seconds,  # seconds after stimulus
)

# calculate baseline
baseline = epochs.sel(reltime=(epochs.reltime < 0)).mean("reltime")
# subtract baseline
epochs_blcorrected = epochs - baseline

# group trials by trial_type. For each group individually average the epoch dimension
blockaverage = epochs_blcorrected.groupby("trial_type").mean("epoch")

n_roi = roi_chans_c3.size
# show results
f, ax = plt.subplots(2, n_roi, figsize=(16, 8))
ax = ax.flatten()
for i_ch, ch in enumerate(roi_chans_c3):
    for ls, trial_type in zip(["-", "--"], blockaverage.trial_type):
        ax[i_ch].plot(
            blockaverage.reltime,
            blockaverage.sel(wavelength=760, trial_type=trial_type, channel=ch),
            "r",
```

```python
            lw=2,
            ls=ls,
        )
        ax[i_ch].plot(
            blockaverage.reltime,
            blockaverage.sel(wavelength=850, trial_type=trial_type, channel=ch),
            "b",
            lw=2,
            ls=ls,
        )
    ax[i_ch].grid(1)
    ax[i_ch].set_title(ch)
    ax[i_ch].set_ylim(-0.05, 0.05)

for i_ch, ch in enumerate(roi_chans_c4):
    for ls, trial_type in zip(["-", "--"], blockaverage.trial_type):
        ax[i_ch + n_roi].plot(
            blockaverage.reltime,
            blockaverage.sel(wavelength=760, trial_type=trial_type, channel=ch),
            "r",
            lw=2,
            ls=ls,
        )
        ax[i_ch + n_roi].plot(
            blockaverage.reltime,
            blockaverage.sel(wavelength=850, trial_type=trial_type, channel=ch),
            "b",
            lw=2,
            ls=ls,
        )
    ax[i_ch + n_roi].grid(1)
    ax[i_ch + n_roi].set_title(ch)
    ax[i_ch + n_roi].set_ylim(-0.05, 0.05)

plt.suptitle(
    "Blockaverage for channels most sensitive to C3 (top) and C4 (bottom): 760nm: r | 
 ↪850nm: b | C3: - | C4: --"
)
plt.tight_layout()
plt.show()
```

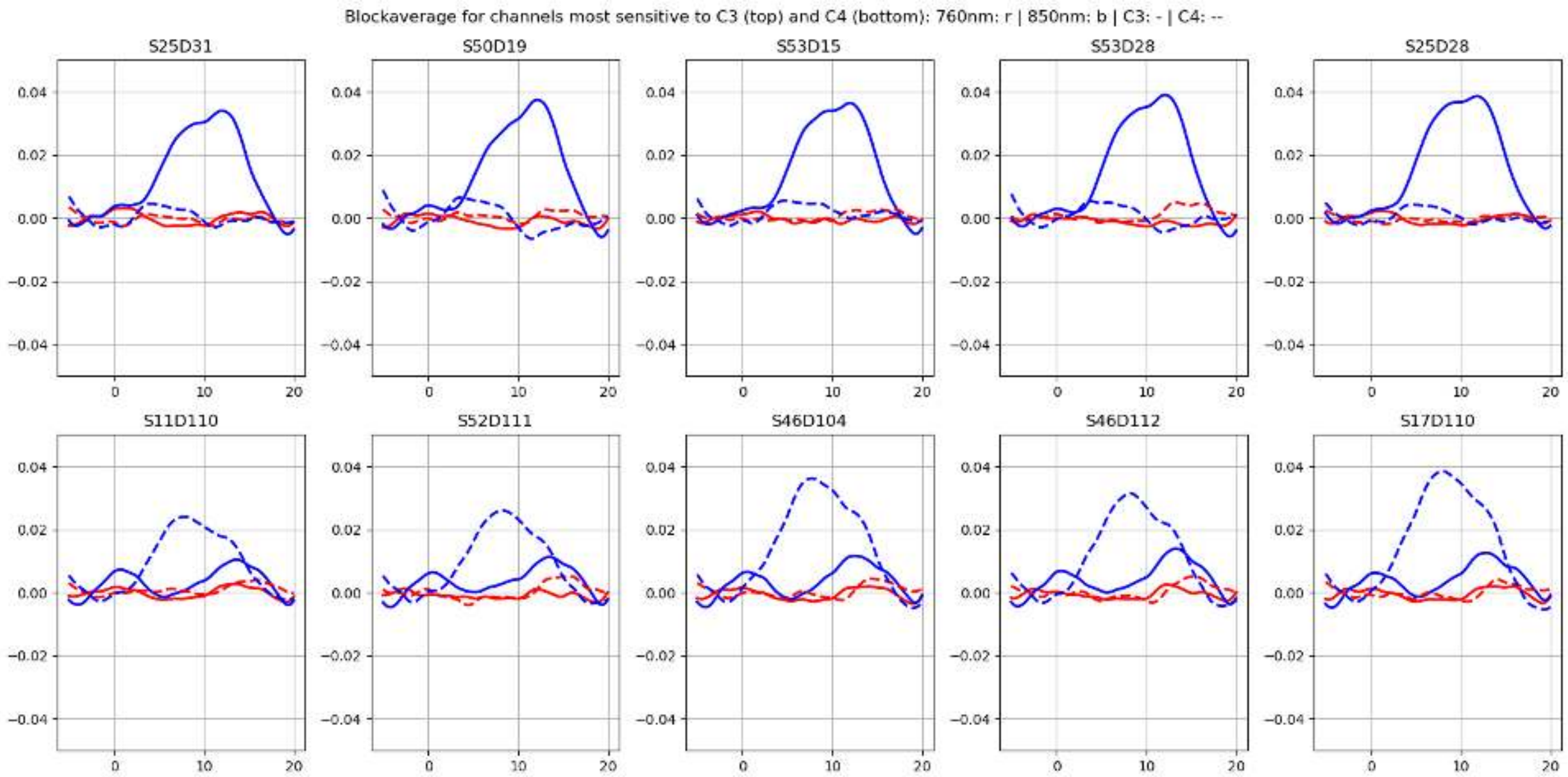

Map block average back to brain surface

We map our extracted block averages back to the brain surface to visualize the recovered HRFs activation for Stim C3. We can compare it to the synthetic HRF image we created earlier.

[39]:
```python
recon = dot.ImageRecon(
    Adot,
    recon_mode="mua2conc",
    alpha_meas=0.5,
    brain_only=True,
    spatial_basis_functions=None
)

blockaverage_img = recon.reconstruct(blockaverage)
blockaverage_img
```

[39]:
```
<xarray.DataArray (chromo: 2, vertex: 25000, trial_type: 2, reltime: 226)> Size: 181MB
[µM] -0.03848 -0.04956 -0.05976 -0.06903 … -0.0005787 -0.0005667 -0.0005552
Coordinates:
  * chromo      (chromo) <U3 24B 'HbO' 'HbR'
    parcel      (vertex) object 200kB 'VisCent_ExStr_8_LH' … 'VisCent_ExStr…
    is_brain    (vertex) bool 25kB True True True True … True True True True
  * reltime     (reltime) float64 2kB -4.995 -4.884 -4.773 … 19.76 19.87 19.98
  * trial_type  (trial_type) object 16B 'Stim C3' 'Stim C4'
Dimensions without coordinates: vertex
```

[40]:
```python
# plot HbO time trace of left and right brain hemisphere during FTapping/Right

for view in ["left_hemi", "right_hemi"]:
    trial_type = "Stim C3"
    gif_fname = "Ftapping-right" + "_HbO_" + view + ".gif"
```

```python
    hbo = blockaverage_img.sel(chromo="HbO", trial_type=trial_type).pint.dequantify()
    hbo_brain = hbo.transpose("vertex", "reltime")

    ntimes = hbo.sizes["reltime"]

    b = cdc.VTKSurface.from_trimeshsurface(head_ras.brain)
    b = pv.wrap(b.mesh)
    b["reco_hbo"] = hbo_brain[:, 0] - hbo_brain[:, 0]

    p = pv.Plotter()

    p.add_mesh(
        b,
        scalars="reco_hbo",
        cmap="seismic",  # 'gist_earth_r',
        clim=(-3.5, 3.5),
        scalar_bar_args={"title": "HbO / µM"},
        smooth_shading=True,
    )

    tl = lambda tt: f"{trial_type} HbO rel. time: {tt:.3f} s"
    time_label = p.add_text(tl(0))

    cog = head_ras.brain.vertices.mean("label")
    cog = cog.pint.dequantify().values
    if view == "left_hemi":
        p.camera.position = cog + [-400, 0, 0]
    else:
        p.camera.position = cog + [400, 0, 0]
    p.camera.focal_point = cog
    p.camera.up = [0, 0, 1]
    p.reset_camera()

    p.open_gif(gif_fname)

    for i in range(0, ntimes, 3):
        b["reco_hbo"] = hbo_brain[:, i] - hbo_brain[:, 0]
        time_label.set_text("upper_left", tl(hbo_brain.reltime[i]))

        p.write_frame()

    p.close()
```

[41]:
```python
display_image("Ftapping-right_HbO_left_hemi.gif")
display_image("Ftapping-right_HbO_right_hemi.gif")
```

Stim C3 HbO rel. time: -1.665 s

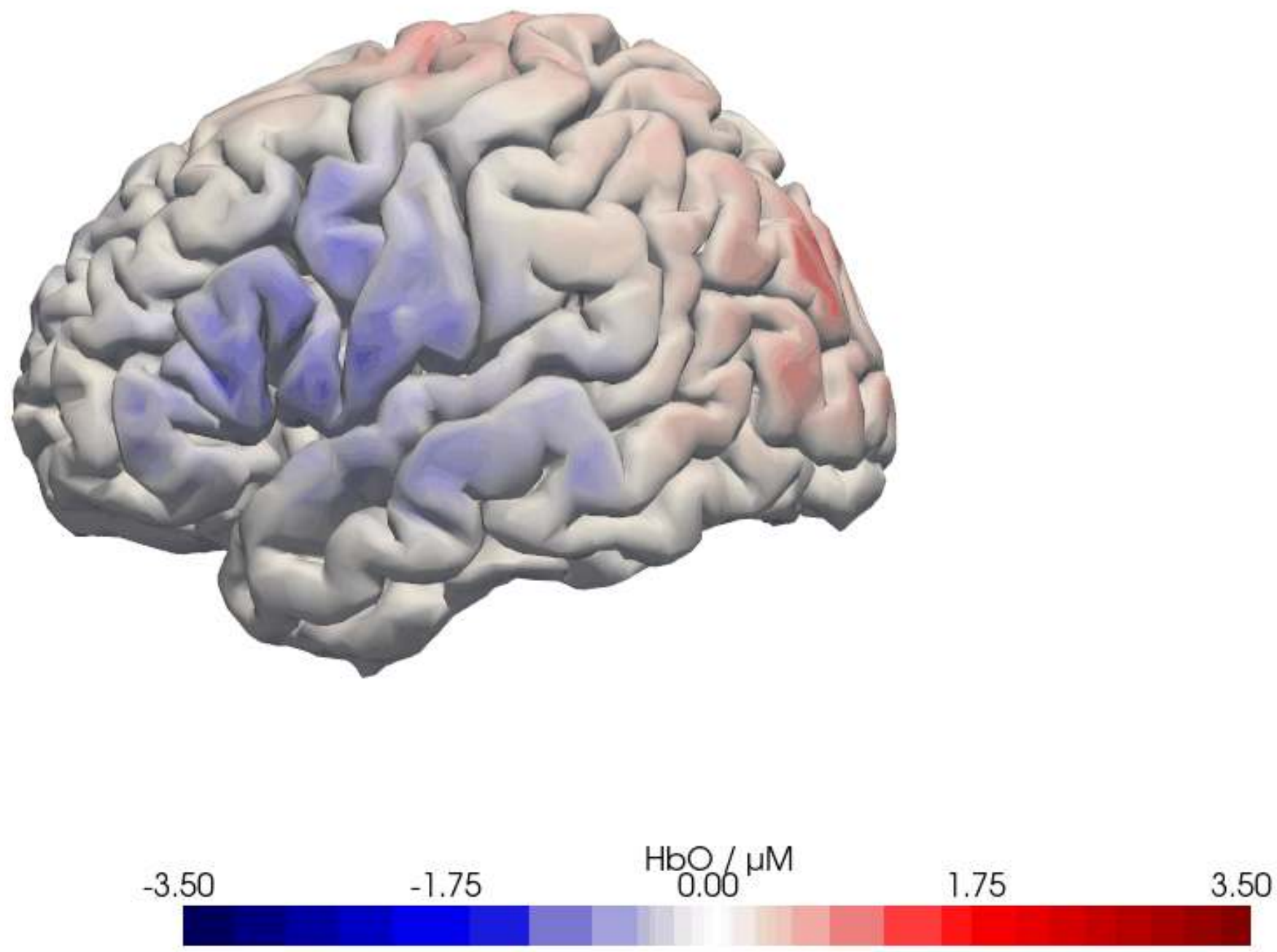

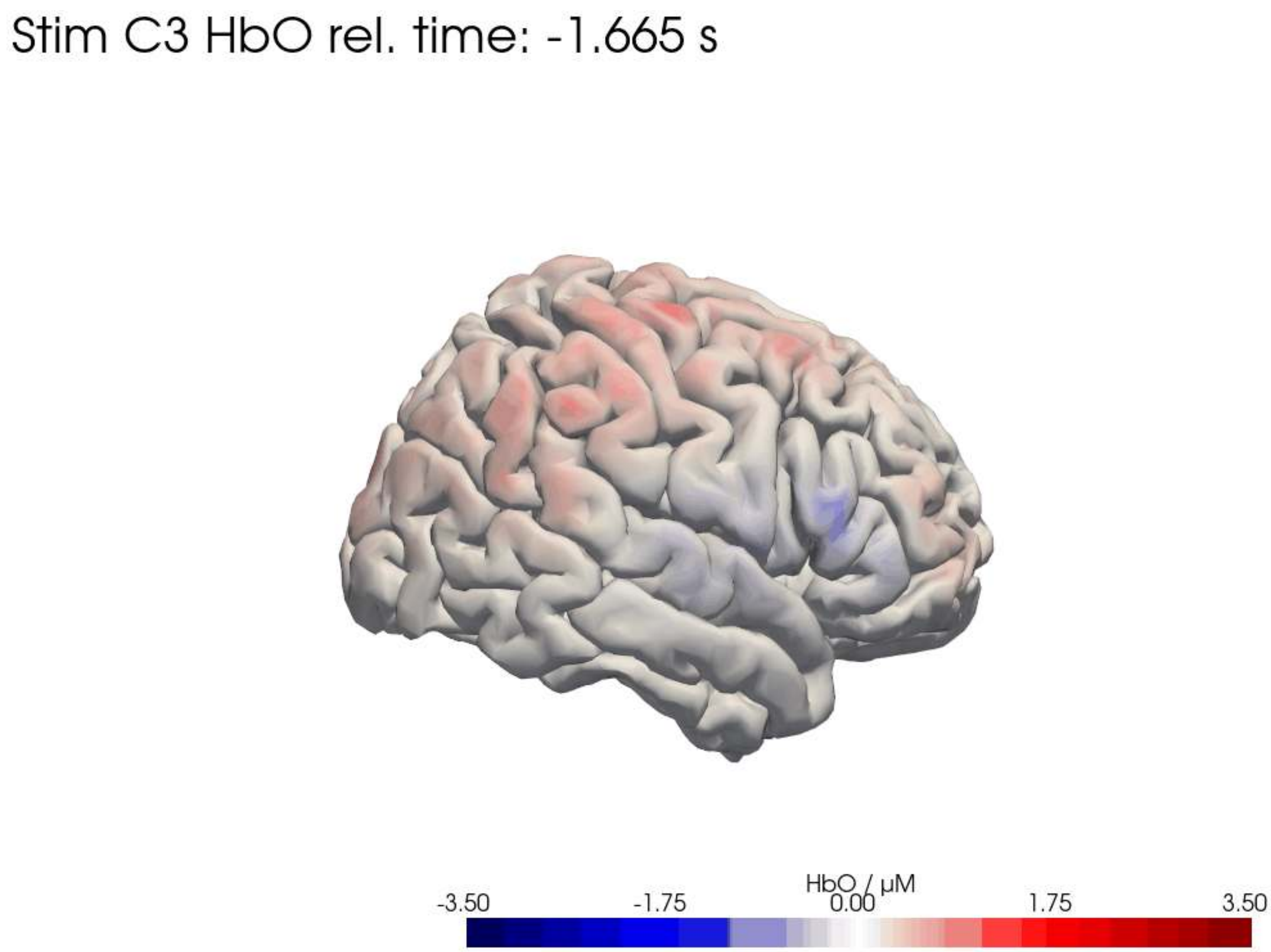

[42]:
```
for c, channel in [("C3", roi_chans_c3[-1]), ("C4", roi_chans_c4[-1])]:
    trial_type = f"Stim {c}"

    f,ax = plt.subplots(2,4, figsize=(14,6))
    vmin,vmax = -2.5,2.5

    cedalion.vis.anatomy.plot_brain_in_axes(
        od,
        head_ras.landmarks,
        spatial_imgs.sel(trial_type=trial_type, chromo="HbO").pint.to("uM"),
        head_ras.brain,
        ax[0,0],
        camera_pos=c,
        cmap="RdBu_r",
        vmin=vmin,
        vmax=vmax,
        cb_label=r"$\Delta$ HbO / µM",
        #title=f"{c} Activation",
    )
    ax[0,0].set_title(f"true activation at {c}")

    trels = [0., 10., 15.]
```

```
    for i_wl, wl in enumerate(blockaverage.wavelength.values):
        tmp = blockaverage.sel(
            trial_type=trial_type, channel=channel, wavelength=wl
        )
        ax[1,0].plot(tmp.reltime, tmp, label=f"{wl} nm")
    for trel in trels:
        ax[1,0].axvline(trel, c="k", ls=":")
    ax[1,0].legend(loc="upper left")
    ax[1,0].set_xlabel("$t_{rel}$")
    ax[1,0].set_ylabel("OD")
    ax[1,0].set_title(channel)

    for i_col, trel in enumerate(trels, start=1):
        for i_row, cam_pos in enumerate(["C3", "C4"]):
            cedalion.vis.anatomy.plot_brain_in_axes(
                od,
                head_ras.landmarks,
                blockaverage_img.sel(chromo="HbO", trial_type=trial_type)
                .sel(reltime=trel, method="nearest"),
                head_ras.brain,
                ax[i_row, i_col],
                camera_pos=cam_pos,
                cmap="RdBu_r",
                vmin=vmin,
                vmax=vmax,
                cb_label=r"$\Delta$ HbO / µM",
                #title="$t_{rel}=" + f"{trel:.1f} s$",
            )
            ax[i_row, i_col].set_title(f"view at {cam_pos} | " + "$t_{rel}=" + f"{trel:
↪.1f} s$")
```

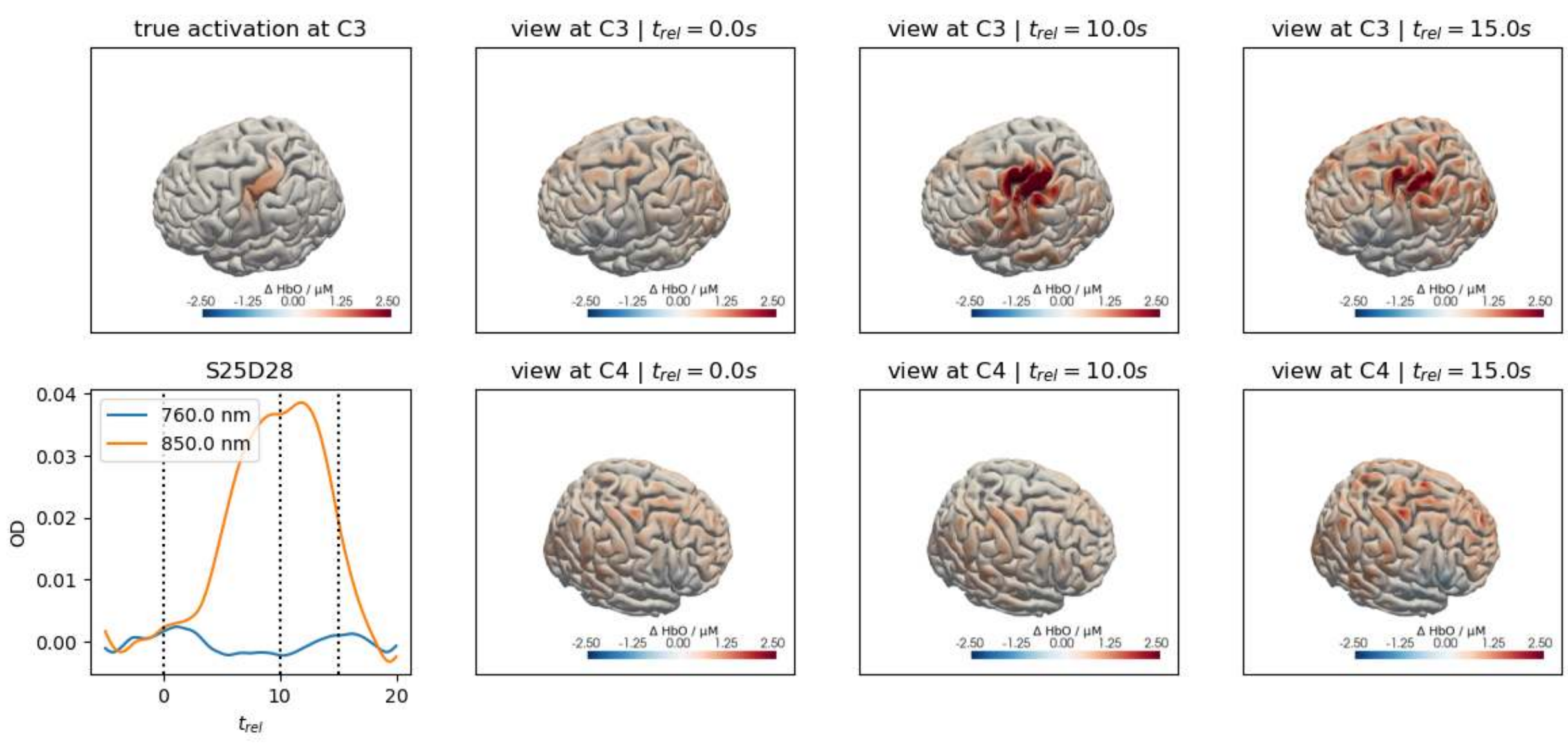

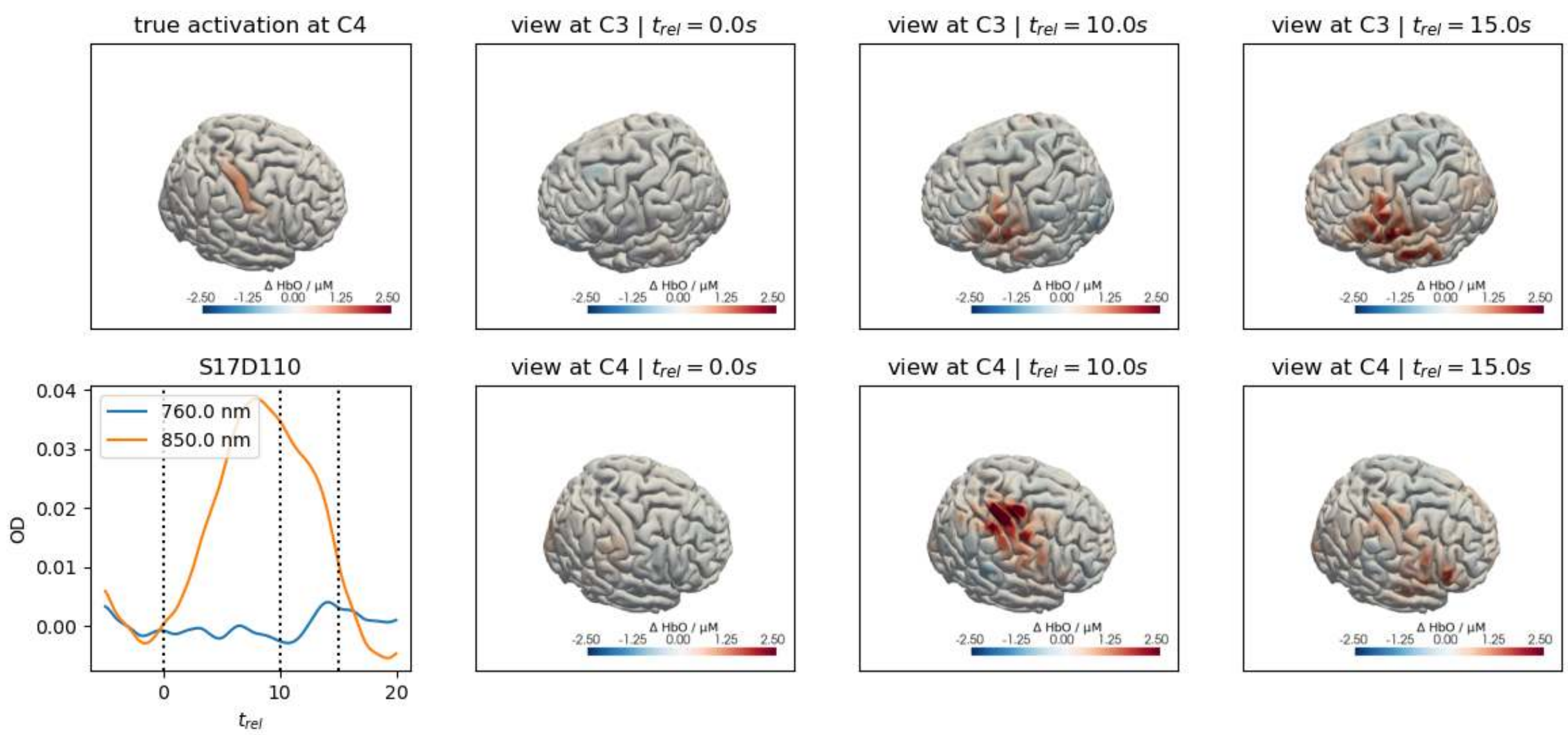

Adding Motion Artifacts

The second part of this notebook demonstrates how to add spike and baseline-shift artifacts to existing time series.

First, we'll load some example data.

[43]:
```python
rec = cedalion.data.get_fingertappingDOT()
rec["od"] = cedalion.nirs.cw.int2od(rec["amp"])

f, ax = plt.subplots(1, 1, figsize=(12, 4))
ax.plot(
    rec["amp"].time,
    rec["amp"].sel(channel="S1D2", wavelength="760"),
    "r-",
    label="760nm",
)
ax.plot(
    rec["amp"].time,
    rec["amp"].sel(channel="S1D2", wavelength="850"),
    "g-",
    label="850nm",
)

plt.legend()
ax.set_xlabel("time / s")
ax.set_ylabel("intensity / V")

display(rec["od"])
```

```
<xarray.DataArray (channel: 100, wavelength: 2, time: 8794)> Size: 14MB
[] 0.02263 0.02322 0.01368 0.005969 0.01692 … 0.01558 0.02343 0.02274 0.02761
Coordinates:
```

```
  * time         (time) float64 70kB 0.0 0.2294 0.4588 … 2.017e+03 2.017e+03
    samples      (time) int64 70kB 0 1 2 3 4 5 … 8788 8789 8790 8791 8792 8793
  * channel      (channel) object 800B 'S1D1' 'S1D2' 'S1D4' … 'S14D31' 'S14D32'
    source       (channel) object 800B 'S1' 'S1' 'S1' 'S1' … 'S14' 'S14' 'S14'
    detector     (channel) object 800B 'D1' 'D2' 'D4' 'D5' … 'D29' 'D31' 'D32'
  * wavelength  (wavelength) float64 16B 760.0 850.0
```

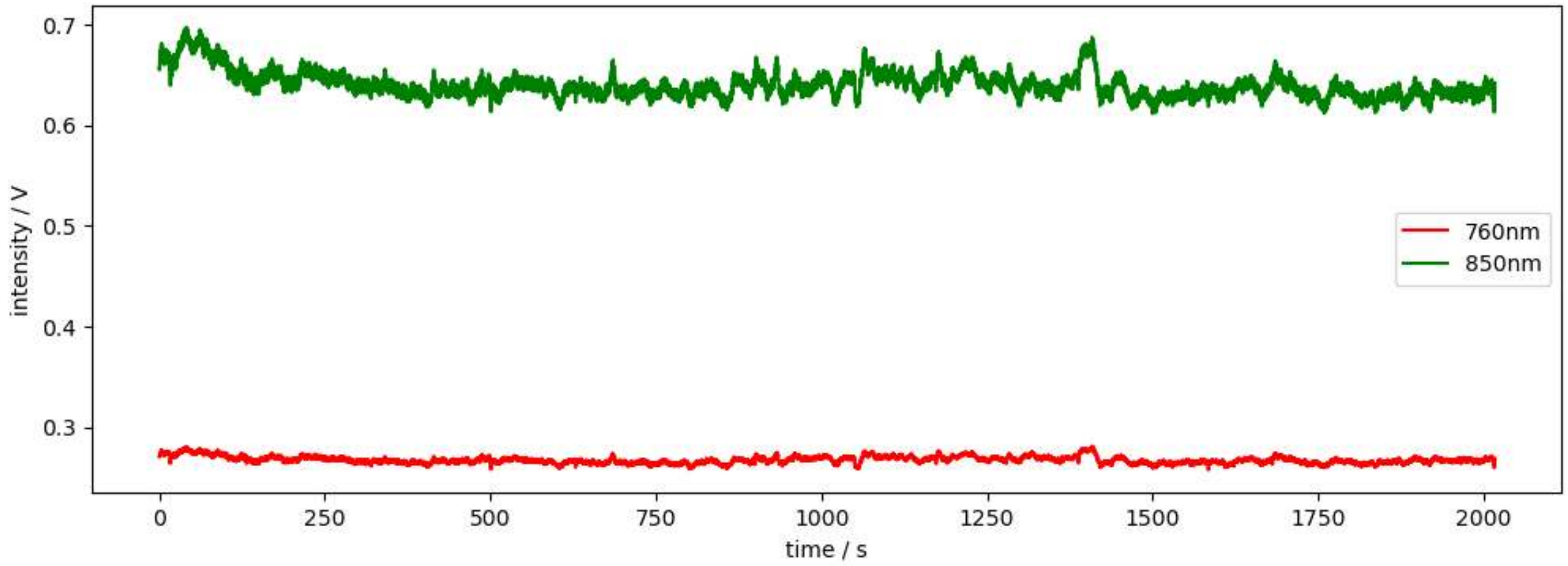

Artifact Generation

Artifacts are generated by the functions `gen_bl_shift` and `gen_spike`.

These function take as arguments: - the time axis of time series - onset time - duration

The amplitude of the generated artifacts is set to 1.

[44]:
```
time = rec["amp"].time

sample_bl_shift = synart.gen_bl_shift(time, 1000) # baseline shift a t = 1000s
sample_spike = synart.gen_spike(time, 1500, 3) # spike artifact at t=1500s for 3s

display(sample_bl_shift)

fig, ax =  plt.subplots(1, 1, figsize=(12,2))
ax.plot(time, sample_bl_shift, "r-", label="bl_shift")
ax.plot(time, sample_spike, "g:", label="spike")
ax.set_xlabel('Time / s')
ax.set_ylabel('Amp')
ax.legend()

plt.tight_layout()
plt.show()
```

```
<xarray.DataArray 'time' (time: 8794)> Size: 70kB
0.0 0.0 0.0 0.0 0.0 0.0 0.0 0.0 0.0 0.0 … 1.0 1.0 1.0 1.0 1.0 1.0 1.0 1.0 1.0
Coordinates:
  * time     (time) float64 70kB 0.0 0.2294 0.4588 … 2.017e+03 2.017e+03
```

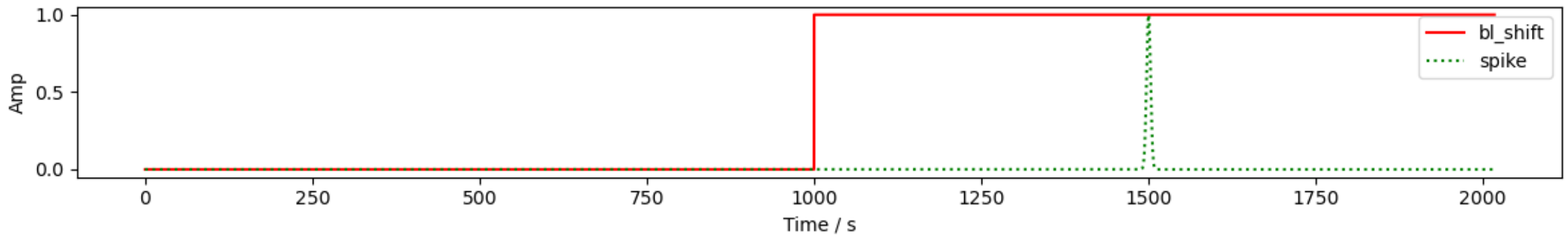

Controlling Artifact Timing

Artifacts can be placed using a timing data frame with columns 'onset', 'duration', 'trial_type', 'value', and 'channel', i.e. it resembles the stimulus data frame with the added 'channel' column.

We can use the function `add_event_timing` to create such timing data frames. The function allows precise control over each event.

The function `sel_chans_by_opt` allows us to select a list of channels by way of a list of optode labels. This reflects the fact that motion artifacts usually stem from the motion of a specific optode or set of optodes, which in turn affects all related channels.

We can also use the functions `random_events_num` and `random_events_perc` to add random events to the data frame, specifying either the number of events or the percentage of the time series duration, respectively.

[45]:
```
# Create a list of events in the format (onset, duration)
events = [(1000, 1), (2000, 1)]

# Creates a new timing dataframe with the specified events.
# Setting channel to None indicates that the artifact applies to all channels.
timing_amp = synart.add_event_timing(events, 'bl_shift', None)

# Select channels by optode
chans = synart.sel_chans_by_opt(["S1"], rec["od"])

# Add random events to the timing dataframe
timing_od = synart.random_events_perc(time, 0.01, ["spike"], chans)

display(timing_amp)
display(timing_od)
```

	onset	duration	trial_type	value	channel
0	1000	1	bl_shift	1	None
1	2000	1	bl_shift	1	None

	onset	duration	trial_type	value	\
0	1483.509973	0.165010	spike	1	
1	207.975880	0.328349	spike	1	
2	662.365745	0.130660	spike	1	
3	1671.834040	0.182054	spike	1	
4	288.476823	0.147720	spike	1	
..	…	…	…	…	
75	566.218925	0.285533	spike	1	
76	1149.287446	0.380106	spike	1	
77	907.677480	0.140890	spike	1	
78	616.329117	0.291620	spike	1	

```
79  1588.501196  0.384842      spike     1

                                     channel
0   [S1D1, S1D2, S1D4, S1D5, S1D6, S1D8]
1   [S1D1, S1D2, S1D4, S1D5, S1D6, S1D8]
2   [S1D1, S1D2, S1D4, S1D5, S1D6, S1D8]
3   [S1D1, S1D2, S1D4, S1D5, S1D6, S1D8]
4   [S1D1, S1D2, S1D4, S1D5, S1D6, S1D8]
..                                   …
75  [S1D1, S1D2, S1D4, S1D5, S1D6, S1D8]
76  [S1D1, S1D2, S1D4, S1D5, S1D6, S1D8]
77  [S1D1, S1D2, S1D4, S1D5, S1D6, S1D8]
78  [S1D1, S1D2, S1D4, S1D5, S1D6, S1D8]
79  [S1D1, S1D2, S1D4, S1D5, S1D6, S1D8]

[80 rows x 5 columns]
```

Adding Artifacts to Data

The function `add_artifacts` scales artifacts and adds them to time-series data.

The artifact generation functions (see above) are passed as a dictionary. Keys correspond to entries in the column 'trial_type' of the timing data frame, i.e. each event specified in the timing data frame is generated using the function `artifacts[trial_type]`. If mode is 'manual', artifacts are scaled directly by the `scale` parameter, otherwise artifacts are automatically scaled by a parameter `alpha` which is calculated using a sliding window approach.

Finally, artifacts to be added to optical-density time series can be automatically scaled to yield specific concentration amplitudes with the function `add_chromo_artifacts_2_od`.

[46]:
```python
artifacts = {"spike": synart.gen_spike, "bl_shift": synart.gen_bl_shift}

# Add baseline shifts to the amp data
rec["amp2"] = synart.add_artifacts(rec["amp"], timing_amp, artifacts)

# Convert the amp data to optical density
rec["od2"] = cedalion.nirs.cw.int2od(rec["amp2"])

dpf = xr.DataArray(
    [6, 6],
    dims="wavelength",
    coords={"wavelength": rec["amp"].wavelength},
)

# add spikes to od based on conc amplitudes
rec["od2"] = synart.add_chromo_artifacts_2_od(
    rec["od2"], timing_od, artifacts, rec.geo3d, dpf, 1.5
)

# Plot the OD data
channels = rec["od"].channel.values[0:6]
fig, axes = plt.subplots(len(channels), 1, figsize=(12, len(channels) * 2))
if len(channels) == 1:
```

```python
    axes = [axes]
for i, channel in enumerate(channels):
    ax = axes[i]
    ax.plot(
        rec["od2"].time,
        rec["od2"].sel(channel=channel, wavelength="850"),
        "g-",
        label="850nm + artifacts",
    )
    ax.plot(
        rec["od"].time,
        rec["od"].sel(channel=channel, wavelength="850"),
        "r-",
        label="850nm - od",
    )
    ax.set_title(f"Channel: {channel}")
    ax.set_xlabel("Time / s")
    ax.set_ylabel("OD")
    ax.legend()
plt.tight_layout()
```

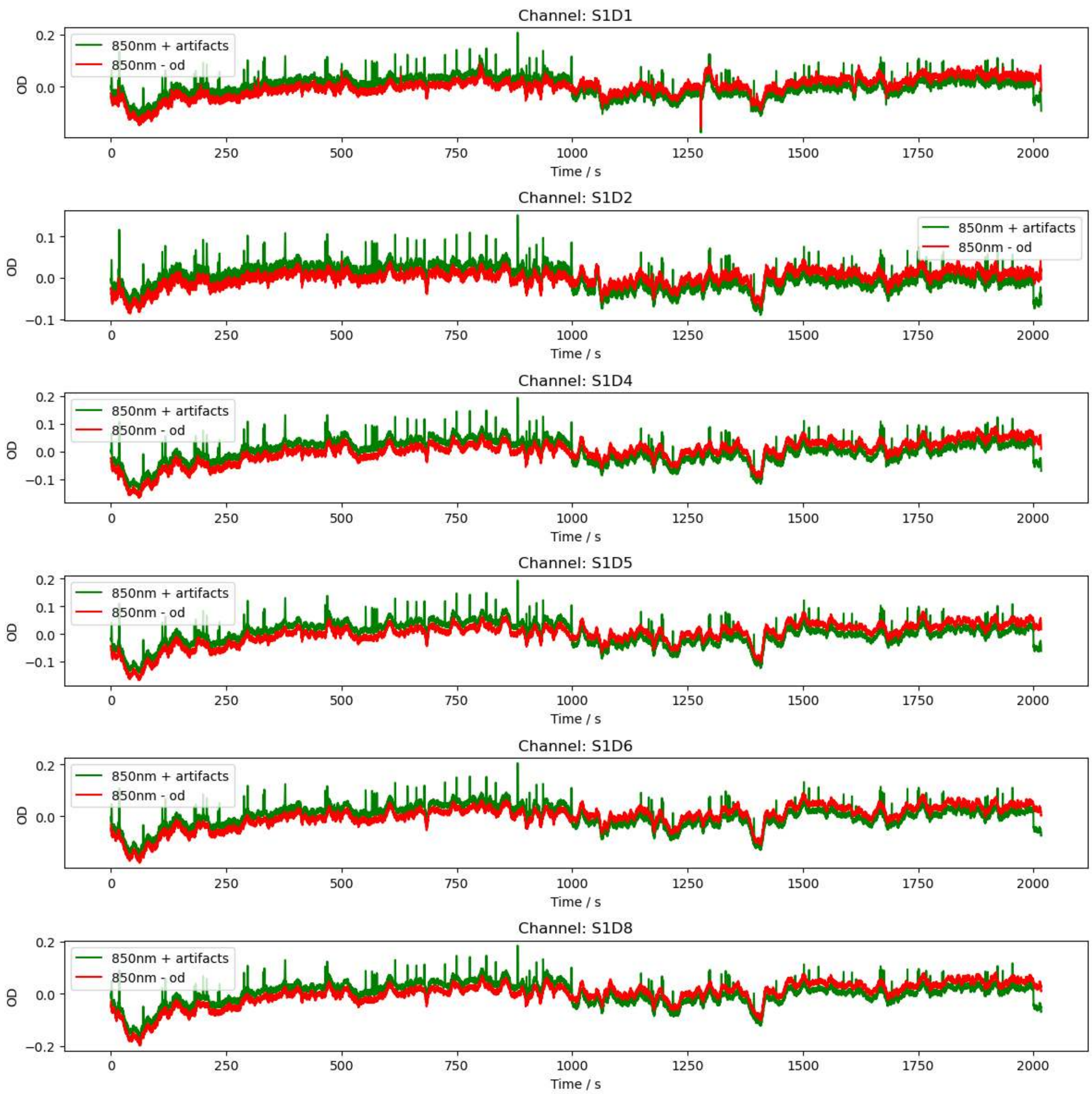

[47]:

```
# Plot the data in conc

rec["conc"] = cedalion.nirs.cw.od2conc(rec["od"], rec.geo3d, dpf)
rec["conc2"] = cedalion.nirs.cw.od2conc(rec["od2"], rec.geo3d, dpf)
channels = rec["od"].channel.values[0:6]
fig, axes = plt.subplots(len(channels), 1, figsize=(12, len(channels) * 2))
if len(channels) == 1:
    axes = [axes]
for i, channel in enumerate(channels):
    ax = axes[i]
    ax.plot(
        rec["conc2"].time,
        rec["conc2"].sel(channel=channel, chromo="HbR"),
        "g-",
```

```
        label="HbR + artifacts",
    )
    ax.plot(
        rec["conc"].time,
        rec["conc"].sel(channel=channel, chromo="HbR"),
        "b-",
        label="HbR",
    )
    ax.set_title(f"Channel: {channel}")
    ax.set_xlabel("Time / s")
    ax.set_ylabel("conc")
    ax.legend()
plt.tight_layout()
plt.show()
```

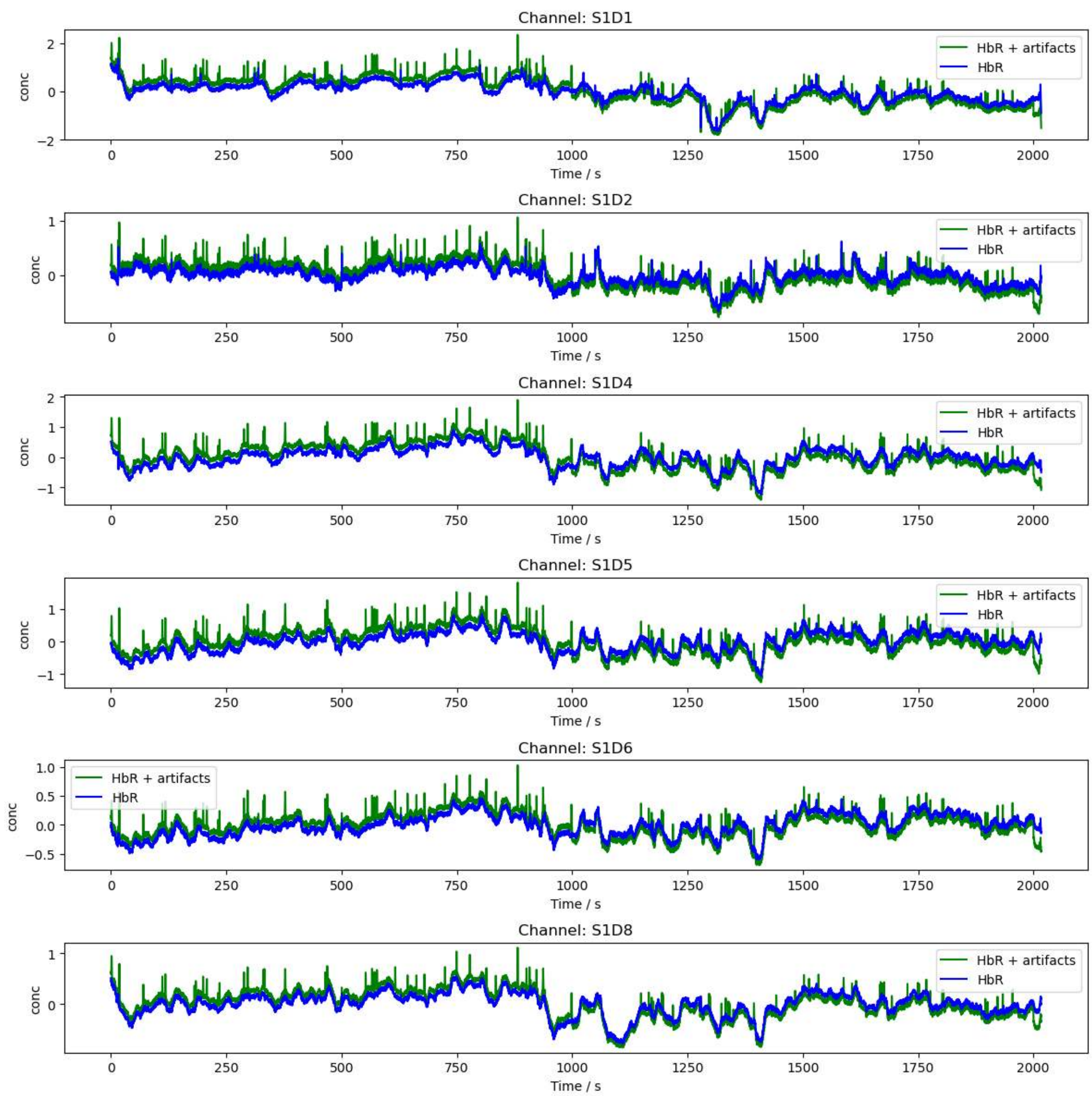

References

```
[48]: cedalion.bib.dump_to_notebook()
```

```
<IPython.core.display.HTML object>
```

You have completed the Cedalion tutorial! Return to the tutorial overview or explore the package feature examples.
